# Four new species of subgenus *Drino* (*sensu stricto*) Robineau-Desvoidy, 1863 from China, with a review of the Chinese species (Diptera, Tachinidae)

**DOI:** 10.3897/BDJ.14.e175030

**Published:** 2026-07-08

**Authors:** Xusheng Liu, Chuntian Zhang

**Affiliations:** 1 Liaoning Key Laboratory of Biological Evolution and Biodiversity, College of Life Science, Shenyang Normal University, Shenyang 110034, China Liaoning Key Laboratory of Biological Evolution and Biodiversity, College of Life Science, Shenyang Normal University Shenyang 110034 China

**Keywords:** tachinids, Eryciini, East Asia, morphology, taxonomy

## Abstract

**Background:**

The subgenus Drino (*sensu stricto*) Robineau-Desvoidy (Diptera, Tachinidae) is a group within the tribe Eryciini, comprising 27 valid species with a wide global distribution, though it is absent from the Australasian region.

**New information:**

The species of the subgenus Drino (*sensu stricto*) Robineau-Desvoidy, 1863 (Diptera, Tachinidae) from China are revised. Fifteen valid species were recognized: *D. adiscalis* (Chao, 1982), *D. angustivitta* Liang & Chao, 1998, *D. argenticeps* (Macquart, 1851), *D. auripollinis* Chao & Liang, 1998, *D. densichaeta* Chao & Liang, 1998, *D. facialis* (Townsend, 1928), *D. flava* Chao & Liang, 1992, *D. interfrons* (Sun & Chao, 1992), *D. laticornis* Chao & Liang, 1998, *D. latifrons* Liu, Zhang & Xi, 2023, *D. longicapilla* Chao & Liang, 1998, *D. longihirta* Chao & Liang, 1992, *D. lota* (Meigen, 1824), *D. minuta* Liang & Chao, 1998, *D. parafacialis* Chao & Liang, 1998, and four species are described as new to science: *D. liaoningensis* **sp. nov**. (Liaoning Province, Northeast China), *D. uniseta* **sp. nov**. (Hubei Province, Central China), *D. meridionalis* **sp. nov**. (Guangxi Autonomous Region, South China) and *D. oharai* **sp. nov**. (Hubei Province, Central China). An identification key to 19 species recorded from China is provided. This is accompanied by 33 figures depicting the head, body, and male terminalia, and a distribution map for the four new species. Additionally, 90 illustrations of the head, body, and terminalia of type specimens of known species are included. The type specimens of the new species are deposited in the Insect Collection of Shenyang Normal University (SYNU), China.

## Introduction

A study of the subgenus Drino (*sensu stricto*) (Diptera: Tachinidae) necessitates a comprehensive review of the taxonomic history of its parent genus. The genus *Drino* (*sensu lato*) Robineau-Desvoidy, 1863 is a relatively diverse group within the tribe Eryciini. According to the classification of O’Hara et al. (2020a), the genus *Drino* (*s. l*.) is subdivided into three subgenera: Drino s.str., Palexorista Townsend, 1921, and Zygobothria Mik, 1891.

Members of the genus *Drino* are medium-sized flies (6.9–12.0 mm) exhibiting highly uniform morphology, particularly in their thoracic and abdominal chaetotaxy. The genus *Drino* is primarily diagnosed by a predominantly red or yellow scutellum (or at least reddish at the apex if mostly black); the presence of four (2+2) katepisternal setae; a single large setula at the base of vein R_4+5_; and the absence of median discal setae on abdominal tergites 3 and 4 according to Tschorsnig and Richter (1998).

Within this framework, the nominate subgenus Drino can be further distinguished by a completely bare parafacial below the lowest frontal seta and the absence of ocellar setae (though these are variable in *D. parafacialis*). O’Hara et al. (2020a) documented that the genus *Drino* (*s. l*.) is widely distributed across all major zoogeographic regions, while the nominate subgenus is subcosmopolitan but notably absent from the Australasian region. Ecologically, these flies serve as significant larval parasitoids, primarily targeting Lepidoptera and Hymenoptera according to Tschorsnig (2017).

The taxonomic definition of the genus *Drino* (*s. l*.) has undergone substantial refinement. The modern concept of the genus *Drino* began with Mesnil (1949b), who redefined the genus *Drino* (*s. l*.) R-D, 1863 from the genus *Sturmia* R-D, 1863, by delineating a group of species characterized by macrotype eggs and distinct morphological traits. Although Mesnil (1949a) proposed a broad classification encompassing seven subgenera, this initial revision lacked comprehensive descriptions of type species. In his subsequent work, Mesnil (1951) provided a key to species and detailed type descriptions; this work remains the most recent comprehensive systematic study of the Palaearctic genus *Drino* (*s. l*.). Of the seven original subgenera, only three—distinguished by a single large setula at the base of vein R_4+5_—are currently retained within the genus; the others have since been elevated to generic status or synonymized.

While Mesnil’s work undeniably laid the essential foundation for the modern taxonomy of the genus *Drino*, his reliance on traditional external morphology has posed persistent challenges for subsequent researchers. A primary limitation is the omission of terminalia characters—an essential diagnostic tool in Tachinidae—in his descriptions of critical Palaearctic type material. Instead, Mesnil’s identification keys relied heavily on highly plastic and subjective external features, such as the subtle patterns of pruinosity on abdominal tergites 3 and 4. Because these characters often exhibit interspecific overlap and intraspecific variation, their utility in species delimitation is limited. Consequently, the lack of genitalic data, coupled with these ambiguous morphological traits, has hindered both precise species identification and the advancement of the group's phylogenetic systematics.

During the late 20th century, research shifted toward regional revisions and the use of genitalic characters. Crosskey (1967) conducted a pivotal systematic revision of the 19 Oriental species of Palexorista (now a subgenus of *Drino*). By utilizing illustrations of male terminalia, Crosskey provided a reliable method to distinguish morphologically similar species, offering a "fresh perspective" on the taxonomy of the *Drino* complex. In his subsequent studies, Crosskey (1976) recorded two Oriental species of the genus *Drino* (*s. str*.), and Crosskey (1977) recorded two Oriental species of the genus *Drino* (*s. str*.), 20 Oriental species of the genus *Palexorista* and three Oriental species of the genus *Zygobothria*. In Crosskey’s research, the taxa now treated as subgenera—namely Drino, Palexorista, and Zygobothria—were treated as independent genera.

In recent decades, significant contributions to the Palaearctic and Oriental faunas have expanded the genus. Herting (1984) catalogued 15 Palaearctic species of the genus *Drino* (*s. l*.) and their synonyms; this catalog synonymized all subgenera into a broadly defined genus *Drino*; subsequent systematic revisions have restored the subgeneric status of Palexorista and Zygobothria. Specifically regarding the Chinese fauna, Chao et al. (1998) reported 32 Chinese species of the genus *Drino* with a key to species and 13 new species of four subgenera Drino, Isochaetina, Palexorista and Zygobothria, which remains the most recent study of the genus and subgenus Drino in China. Morphological standards of genus *Drino* were further solidified by Tschorsnig and Richter (1998), the key to genera provided a strict diagnosis of genus *Drino* in their key to Palaearctic Tachinidae, but like the research of Herting above, this key also synonymized all subgenera under a single genus *Drino*.

The most comprehensive modern treatment is the world checklist by O’Hara et al. (2020a), which recognizes the genus *Drino* as comprising three subgenera (*Drino*, Palexorista, and Zygobothria) and includes 26, 51, and 9 species respectively, alongside 39 unplaced species. Most recently, the discovery of a new species by Liu et al. (2023) has increased the number of recorded species of the genus *Drino* (*s. l*.) in China to 27, reflecting the ongoing taxonomic expansion of this group.

We systematically examined the type specimens of 15 species distributed in China and over 200 additional specimens belonging specifically to the nominate subgenus Drino, as opposed to subgenera Palexorista and Zygobothria, housed in the National Zoological Museum of China, Institute of Zoology, Chinese Academy of Sciences, Beijing (IZCAS), Museum national d'Histoire naturelle, Paris, France (MNHN), National Museum of Natural History, Washington, D.C., United States of America (NMNH) and the Insect Collection of Shenyang Normal University (SYNU). The type materials of the four new species are deposited in SYNU.

Our study recognized 15 known species and identified four new species, which are described and illustrated herein. An identification key to 19 species recorded from China is provided. This is accompanied by a distribution map (Fig. 1) and 32 figures on eight plates (Figs 2–9) depicting the head, body, and male terminalia of the four new species. Additionally, 90 illustrations on 30 plates of the head, body, and terminalia of type specimens of known species are included (Figs 10–39).

## Materials and methods

This study examined over 200 tachinid specimens of the subgenus Drino primarily based on physical material housed in **SYNU** and **IZCAS**, China. All primary specimen data and relevant photographs were derived from the following four collections, categorized by physical examination or digital consultation:

- **IZCAS** Institute of Zoology, Chinese Academy of Sciences, Beijing, China
- **MNHN** Muséum national d’Histoire naturelle, Paris, France
- **NMNH** National Museum of Natural History, Smithsonian Institution, Washington, D.C., USA
- **SYNU** Shenyang Normal University, Shenyang, China

Morphological terminology and measurements follow Tschorsnig and Richter (1998) and Cumming and Wood (2017). Specimens were preliminarily identified using the key in Tschorsnig and Richter (1998), with subsequent confirmations based on examination of type specimens.

Specimens were examined using an OLYMPUS SZX7 stereomicroscope (at SYNU) and a LEICA SAPO stereomicroscope (at IZCAS). For the four new species, digital images of the head and body were captured using a Canon EOS 60D camera equipped with a Canon MP-E 65mm f/2.8 1–5× Macro Lens (Manual Focus, EF Mount). Detailed imaging of the male terminalia utilized an infinity-corrected optical system, incorporating an Olympus UPlanApo 10× microscope objective (NA = 0.25) coupled with a tube lens assembly (based on a Raynox DCR-150 lens with a 208 mm focal length).

All stacking sequences were performed on a WeMacro automatic focus stacking rail under a light source with a color rendering index (CRI) exceeding 97.6%. Successive frames were taken with an approximately 50%–60% overlap and processed using Helicon Focus v.8.1.0. In contrast, images of the type specimens housed in IZCAS were captured as single exposures using the same Canon EOS 60D camera equipped with a Canon EF 100mm f/2.8L Macro IS USM lens, using an on-camera flash for illumination. As a final step for plate preparation, Adobe Photoshop 2020 was utilized for image refinement. This included background normalization, cropping, and the application of a subtle sharpening filter to maximize the clarity of morphological structures without altering the biological features of the specimens.

In addition, type material photographs of *Drino facialis* (*Global Biodiversity Information Facility 2025d*), *D. lota* (*Global Biodiversity Information Facility 2025a*), and *D. argenticeps* (*Global Biodiversity Information Facility 2025b*) were obtained from the Global Biodiversity Information Facility (GBIF) website. These were utilized for comparative morphological analysis, although only representative male specimens providing clear diagnostic features are illustrated in this study: the syntype of *Drino lota* (Meigen) (MNHN ED4773, Muséum national d'Histoire naturelle, Paris), the holotype of *Drino argenticeps* (Macquart, 1851) (MNHN ED9461, same collection), and the holotype of *Drino facialis* (Townsend) (NMNH USNMENT01518434, National Museum of Natural History, Washington, D.C.).

Male terminalia were dissected following the method of O’Hara (2002), and the dissected parts were stored in glycerin within a small plastic microvial pinned beneath the source specimen. Terminalia illustrations were created using Procreate on an iPad, based on focus-stacked digital photographs to ensure morphological accuracy. For the four new species, the illustrations were derived directly from focus-stacked photographs of dissected parts, reflecting their precise morphological structures. For the 15 previously described valid species, illustrations were either newly prepared from dissected specimens examined in this study or modified from Chao et al. (1998) based on current observations.

Abbreviations used in the descriptions: *ac*, acrostichal seta; *dc*, dorsocentral seta; *ia*, intra-alar seta; *sa*, supra-alar seta; *ad*, anterodorsal seta; *av*, anteroventral seta; *d*, dorsal seta; *p*, posterior seta; *pd*, posterodorsal seta; *pv*, posteroventral seta; *v*, ventral seta.

## Taxon treatments

### Drino

Robineau-Desvoidy, 1863

E300F4D8-2C02-57C3-A4A2-DF0165A0F0C9

Drino volucris Robineau-Desvoidy, 1863

#### Diagnosis

Eye bare or with sparse, inconspicuous ommatrichia shorter than two times the facet diameter; however, species like *Drino lota* (Meigen, 1824) or *D. longihirta* Chao & Liang, 1992 possess sparse ommatrichia approximately four times the facet diameter. Ocellar setae vary across subgenera: strong and subequal to reclinate orbital setae in subgenus Zygobothria; absent in *Drino* (*s.str*.) (except in *D. parafacialis*, where they are variable, thin, short, or absent); or thinner and shorter than reclinate orbital setae in subgenus Palexorista. Vibrissae usually inserted well above facial margin in both sexes. First flagellomere rounded apically. Scutellum predominantly red or yellow; if predominantly black, then at least reddish near apex. First postsutural *sa* longer than notopleural seta and first postsutural intra-alar seta. Prosternum with setulae on both sides. Katepimeron bare or with at most several sparse hairs on anterior fourth. Katepisternum with four setae. Base of vein R_4+5_ with a single large setula (sometimes accompanied by a minute supernumerary hair or an additional shorter setula). Abdominal tergites 3 and 4 without median discal setae. Male abdominal tergite 4 usually with secondary sexual hair-patches on the ventral surface.

#### Distribution

Six biogeographic (Afrotropical, Australasian, Nearctic, Neotropical, Oriental, Palaearctic) regions, 127 known species in the world, 39 species in China (O’Hara and Henderson 2020, O’Hara et al. 2020a, O’Hara et al. 2020b, Liu et al. 2023). *Drino* s. str. is subcosmopolitan except the Australasian region (O’Hara et al. 2020a).

#### Subgenus Drino Robineau-Desvoidy, 1863

##### Nomenclature

*Drino* Robineau-Desvoidy, 1863: 250. Type species: *Drino volucris* Robineau-Desvoidy, 1863 (= *Tachina lota* Meigen, 1824), by original designation [France]. – Mesnil 1949a: 2 (revision, the first to classify *Drino* (*s.str*.) as subgenus). – Mesnil 1949b: 87 (revision). – Mesnil 1951: 158 (key and descriptions). – Herting 1974: 8 (notes). – Crosskey 1976: 237 (Catalog). – Crosskey 1977: 675 (Catalog). – Crosskey 1980: 870 (Catalog). – Chao and Shi 1982: 271 (Catalog). – Crosskey 1984: 284 (key). – Herting 1984: 52 (Catalog). – Herting and Dely-Draskovits 1993: 207 (Catalog). – Tschorsnig and Herting 1994: 54, 136 (key and distribution). – Tschorsnig and Richter 1998: 803 (key). – Chao et al. 1998: 1827 (key and descriptions). – Liu et al. 1998: 162 (key and descriptions). – Sabrosky 1999: 118 (family-group names). – Richter 2004: 190 (key). – Xue and Wang 2006: 251 (Catalog). – O’Hara et al. 2009: 66 (Catalog). – Zhang 2016: 242 (key and descriptions). – Tschorsnig 2017: 158 (Host Catalog). – O’Hara et al. 2020a: 314 (World checklist). – O’Hara et al. 2020: 879 (Catalog).

*Phorcida* Robineau-Desvoidy, 1863. – Robineau-Desvoidy 1863: 251. Type species: *Phorcida acronyctae* Robineau-Desvoidy, 1863, by original designation [France]. – Mesnil 1949a: 8 (revision). – Mesnil 1949b: 87 (revision). – Mesnil 1951: 156 (key and descriptions). – Herting 1974: 8 (notes). – Liu et al. 1998: 162 (key and descriptions).

*Zygosturmia* Townsend, 1911. – Townsend 1911: 142, based on female reproductive system [Townsend 1912: 323, adult description]. Type species: *Zygosturmia inca* Townsend, 1911, by monotypy [Peru]. – Mesnil 1949a: 9 (revision). – Mesnil 1949b: 87 (revision). – Mesnil 1951: 156 (key and descriptions). – Sabrosky 1999: 118 (family-group names). – Liu et al. 1998: 162 (key and descriptions). – O’Hara et al. 2020a: 315 (Checklist).

*Laximasicera* Curran, 1927. – Curran 1927: 14. Type species: *Laximasicera sexualis* Curran, 1927 (=*Sturmia bakeri* Coquillett, 1897), by original designation [Canada]. – O’Hara et al. 2020a: 315 (Checklist).

*Anazygosturmia* Townsend, 1927. – Townsend 1927: 271. Type species: *Anazygosturmia analis* Townsend, 1927, by original designation [Brazil]. – Mesnil 1949a: 9 (revision). – Mesnil 1949b: 87 (revision). – Mesnil 1951: 156 (key and descriptions). – Chao et al. 1998: 1827 (key and descriptions). – Liu et al. 1998: 163 (key and descriptions). – O’Hara et al. 2020a: 315 (Checklist).

*Sturmiodoria* Townsend, 1928. – Townsend 1928: 391. Type species: *Sturmiodoria facialis* Townsend, 1928, by original designation [Philippines]. – Mesnil 1949a: 9 (revision). – Mesnil 1949b: 87 (revision). – Mesnil 1951: 156 (key and descriptions). – Crosskey 1976: 237 (Catalog). – Crosskey 1977: 675 (Catalog). – Crosskey 1980: 870 (Catalog). – Chao et al. 1998: 1827 (key and descriptions). – Liu et al. 1998: 163 (key and descriptions). – O’Hara et al. 2009: 66 (Catalog). – Zhang 2016: 242 (key and descriptions). – O’Hara et al. 2020a: 315 (Checklist).

*Cubaemyia* Townsend, 1931. – Townsend 1931: 179. Type species: *Tachina cubaecola* Jaennicke, 1867, by original designation [Cuba]. – O’Hara et al. 2020a: 315 (Checklist).

*Gyrovaga* Townsend, 1933. – Townsend 1933: 473 (junior homonym of *Gyrovaga* Gistel, 1848). Type species: *Tachina vicina* Zetterstedt, 1849, by original designation [Denmark]. – Mesnil 1949a: 9 (revision). – Mesnil 1949b: 88 (revision). – Mesnil 1951: 156 (key and descriptions). – Herting 1984: 52 (Catalog). – Herting and Dely-Draskovits 1993: 207 (Catalog). – Chao et al. 1998: 1827 (key and descriptions). – Liu et al. 1998: 163 (key and descriptions). – O’Hara et al. 2020a: 315 (Checklist).

##### Diagnosis

Parafacial completely bare. Ocellar setae absent (*D. parafacialis* exceptional and its ocellar setae are variable, thin, short or absent).

### Drino liaoningensis

Zhang & Liu sp. nov.

96F42649-9543-5244-8A51-3F16622DC6FB

B6F9B473-4130-4C03-B26E-C7BD8B54C2BA

#### Materials

- **Type status:** Holotype. **Occurrence:** recordedBy: Henan Li; individualCount: 1; sex: male; lifeStage: adult; occurrenceID: 8099BC46-2FD8-569E-A3EA-DCA635D51940; **Taxon:** scientificName: *Drino liaoningensis*; **Location:** country: China; stateProvince: Liaoning; locality: Huangdaigou, Qingyuan County; verbatimElevation: 600–800 m; verbatimCoordinates: 41.83°N, 124.92°E; decimalLatitude: 41.83; decimalLongitude: 124.92; **Identification:** identifiedBy: Chuntian Zhang; dateIdentified: 2015; **Event:** samplingProtocol: sweeping; eventDate: 26–31.Ⅴ.2015; **Record Level:** collectionCode: Insects
- **Type status:** Paratype. **Occurrence:** recordedBy: Chuntian Zhang; individualCount: 1; sex: male; lifeStage: adult; occurrenceID: A5132677-30B3-52E8-B2C0-A31CCCC78634; **Taxon:** scientificName: *Drino liaoningensis*; **Location:** country: China; stateProvince: Liaoning; locality: Huangdaigou, Qingyuan County; verbatimElevation: 600–800 m; verbatimCoordinates: 41.83°N, 124.92°E; decimalLatitude: 41.83; decimalLongitude: 124.92; **Identification:** identifiedBy: Chuntian Zhang; dateIdentified: 2015; **Event:** samplingProtocol: sweeping; eventDate: 26–31.Ⅴ.2015; **Record Level:** collectionCode: Insects

#### Description

Body length 11.1–12.0 mm (Fig. 2a, b).

**Male**. Head black (Fig. 2c, d) in ground color. Eye nearly bare, with sparse ommatrichia about 2 facets in length. Frontal vitta dark brown; fronto-orbital plate with dingy pale yellowish pruinosity especially on upper half; parafacial, face, gena and occiput covered with grayish white pruinosity; lunule brown. Antenna covered with pale yellowish pruinosity, postpedicel pale brown at base in ground color, palpus dark brown and gradually darkened at base, prementum gleaming black. Frons 1.2–1.3 times longer than facial length, about 0.3 times of head width or 0.8–0.9 times eye width, frontal vitta parallel or slightly widened posteriorly, 1.7 times narrower than fronto-orbital plate at middle; parafacial at narrowest point about 1.5 times postpedicel width and bare below lowest frontal seta; genal height about 0.2 times eye height; 6–10 frontal setae, 3–5 frontal setae below antennal insertion, descending to parafacial and lowest one descending to mid or lowest level of pedicel, fronto-orbital plate with a row of 3–4 reclinate orbital setae and shorter setae on outer surface, ocellar seta absent, outer vertical seta distinctly longer than postocular setula, inner vertical seta about 0.5–0.6 times eye height; postocular setae short and straight. Face appreciably sunken with a low middle facial ridge, lower margin of face not protruding forward, facial ridge with setae only on lower 1/5, vibrissa slightly inserted above lower facial margin, a row of 9–12 strong subvibrissae about 0.4 times as long as vibrissa. Occiput on upper half mostly with white hairs and 1–2 rows of black setulae behind postocular row. Antenna with postpedicel about 2.5 times as long as pedicel, arista thickened at basal 2/5–1/2, first aristomere short, second aristomere 1.5 times longer than diameter. Palpus about 1.1 times as long as postpedicel. Prementum 2.2–2.3 times longer than width, labellum large.

**Thorax** black in ground color, covered with grayish white pruinosity on both sides, and grayish white with sparse yellow pruinosity on dorsal side, 5 dark longitudinal vittae on scutum. Scutellum reddish yellow except for base black, 3+3 *ac*, 3+4 *dc*, 1+3 *ia*, 1+3 *sa*, first one slightly longer than notopleural seta and distinctly longer than postsutural intra-alar seta, postpronotal lobe with 4 setae, anterior one short, 3 of them arranged in a straight line and anterior one located between inner two setae, scutellum with 5 pairs of marginal setae, apical scutellar setae crossed and about as long as scutellum, 2 lateral scutellar setae about 0.7–0.9 times as long as subapical setae, a pair of discal scutellar setae. Prosternum with setulae on both sides, proepisternum bare, anepisternum with 1 seta anteriorly and a row of 7 to 8 setae posteriorly, anepimeron with 1–2 seta(e), one of them strong, katepisternum with 4 setae, katepimeron at most with a few hairs on anterior quarter, anatergite bare.

**Wing** wholly hyaline, tegula black, basicosta dark brown. Calypters white, margins wholly yellowish, costal spine short and indistinct, 2nd and 3rd costal sectors bare ventrally, relative lengths of 2nd, 3rd and 4th costal sectors approximately in ratio of 14:20:11, base of R_4+5_ with one setula dorsally and ventrally, about same length as crossvein r-m, vein M curved at bending, vein M from bend to crossvein dm-cu longer than distance between bend and wing hind margin (5:3). Halter yellow on basal half and dark brown on distal half.

**Legs** dark brown, pulvilli yellowish, fore claw longer than 5th tarsomere. Fore tibia with a row of sparse short *ad*, 2 *p*, 1 short preapical *ad* and 1 long preapical *d*, 1 long preapical *pv*. Mid tibia with 1 *ad*, 2 *p* and 1 *v*. Hind tibia with a row of irregular long *ad* in which one is slightly stronger, 2–3 *v* (only lowest one always strong), a row of irregular *pd*, 1 preapical dorsal and *ad*, 1 preapical *av*, preapical *pv* absent.

**Abdomen** long ovate, black in ground color dorsally, covered with yellowish gray pruinosity, a narrow black median vitta on tergites 3 (distinctly) to 4, reddish yellow on both sides of syntergite 1+2 to tergite 4. Mid-dorsal depression of syntergite 1+2 extending to its posterior margin, syntergite 1+2 and tergite 3 each with 2 short median marginal setae and a pair of lateral marginal setae, setae on syntergite 1+2 shorter than that on tergite 3, median discal and lateral discal setae absent on tergites 3 and 4, and a complete row of marginal setae present on tergite 4. Tergites 4 with large sexual hair patches of appressed hairs ventrally. Sternite 5 square, lateral lobe with three or more distinctly long and strong setae and some weaker setae, bluntly round at apex, median U shape cleft about 2/5 length of sternite 5.

**Male terminalia** (Fig. 3). Cerci evenly narrowed, slightly pointed at apex, narrowly separated on apical 3/5 in caudal view, cerci nearly straight and as wide as surstyli at middle in lateral view, narrowed to apex. Surstylus about same length as cercus or slightly longer, bluntly round at apex in caudal view, blunt in lateral view. Pregonite with several setae on dorsal surface; postgonite about 2 times epiphallus length, about 1/2 pregonite width at middle; epiphallus short and hook-like, shorter than postgonite; basiphallus and distiphallus distinctly sclerotized; distiphallus weakly expanded in lateral view; acrophallus round at apex.

**Female**. Unknown.

#### Diagnosis

Eye nearly bare, with sparse ommatrichia about 2 facets in length, frons 1.2–1.3 times longer than facial length, about 0.3 times head width, parafacial at narrowest point about 1.5 times antenna width and bare below lowest frontal seta, genal height about 0.2 times eye height, 3–5 frontal setae descending to parafacial, ocellar seta absent, outer vertical seta distinctly longer than postocular setula. Cerci narrowly separated on apical 3/5 in caudal view, about as wide as surstyli at middle in lateral view; surstylus about same length as cerci, bluntly rounded at apex in caudal view, blunt in lateral view. Postgonite about 2 times epiphallus length, about 1/2 pregonite width at middle.

#### Etymology

The epithet is from the type locality, Liaoning, China.

#### Distribution

China (Province Liaoning).

#### Remarks

*Drino parafacialis* Chao & Liang, 1998 from China (Zhejiang) is closely similar to this new species, but is distinguished from parafacial at narrowest point only slightly wider than postpedicel width, outer vertical seta almost as long as postocular setulae, genal height about 0.1 times eye height, cercus about 1/2 surstylus width at middle in lateral view (as in Fig. 38b).

### Drino meridionalis

Zhang & Liu sp. nov.

DC0F38F1-A8CE-5416-BF49-9D695EF8C666

6A679127-F596-4489-8532-4BBA695B5891

#### Materials

- **Type status:** Holotype. **Occurrence:** recordedBy: Qiang Wang; individualCount: 1; sex: male; lifeStage: adult; occurrenceID: CF24B0DA-DAEB-59BB-8AED-627E401604E7; **Taxon:** scientificName: *Drino meridionalis*; **Location:** country: China; stateProvince: Guangxi Autonomous Region; locality: Pingmeng, Napo County; verbatimElevation: 400–1000 m; **Identification:** identifiedBy: Chuntian Zhang; dateIdentified: 2011; **Event:** samplingProtocol: sweeping; eventDate: 7–10.V.2011; **Record Level:** collectionCode: Insects
- **Type status:** Paratype. **Occurrence:** recordedBy: Qiang Wang; individualCount: 10; sex: male; lifeStage: adult; occurrenceID: 359CD990-3C3D-53B6-A727-6075E6B9C668; **Taxon:** scientificName: *Drino meridionalis*; **Location:** country: China; stateProvince: Guangxi Autonomous Region; locality: Pingmeng, Napo County; verbatimElevation: 400–1000 m; **Identification:** identifiedBy: Chuntian Zhang; dateIdentified: 2011; **Event:** samplingProtocol: sweeping; eventDate: 7–10.V.2011; **Record Level:** collectionCode: Insects
- **Type status:** Paratype. **Occurrence:** recordedBy: Qiang Wang; individualCount: 1; sex: male; lifeStage: adult; occurrenceID: 0B42CFBC-830B-53D3-A0FB-2B4E218EBD62; **Taxon:** scientificName: *Drino meridionalis*; **Location:** country: China; stateProvince: Guangxi Autonomous Region; locality: Nonggang, Longzhou County; verbatimElevation: 300–450 m; **Identification:** identifiedBy: Chuntian Zhang; dateIdentified: 2011; **Event:** samplingProtocol: sweeping; eventDate: 1–2, 4.V.2011; **Record Level:** collectionCode: Insects
- **Type status:** Paratype. **Occurrence:** recordedBy: Qiang Wang; individualCount: 1; sex: female; lifeStage: adult; occurrenceID: 319C02B5-5CDC-5FB8-B2DC-E4F0330DDD4B; **Taxon:** scientificName: *Drino meridionalis*; **Location:** country: China; stateProvince: Guangxi Autonomous Region; locality: Nonggang, Longzhou County; verbatimElevation: 300–450 m; **Identification:** identifiedBy: Chuntian Zhang; dateIdentified: 2011; **Event:** samplingProtocol: sweeping; eventDate: 1–2.V.2011; **Record Level:** collectionCode: Insects
- **Type status:** Paratype. **Occurrence:** recordedBy: Haoxin Lin; individualCount: 1; sex: female; lifeStage: adult; occurrenceID: 1A561EFA-6574-5880-8F2E-D3C3A237FBE2; **Taxon:** scientificName: *Drino meridionalis*; **Location:** country: China; stateProvince: Fujian; locality: Yanping, Nanping County; **Identification:** identifiedBy: Chuntian Zhang; dateIdentified: 2015; **Event:** samplingProtocol: sweeping; eventDate: Ⅵ.2015; **Record Level:** collectionCode: Insects

#### Description

Body length 9.3–10.5 mm (Fig. 4a, b).

**Male**. Head black (Fig. 4c, d) in ground color. Eye nearly bare, with sparse ommatrichia shorter than 2 facets in length. Frontal vitta dark brown; fronto-orbital plate, parafacial, face, gena and occiput covered with grayish white pruinosity; lunule brown. Antenna covered with pale yellow pruinosity, postpedicel pale brown at base in ground color, palpus yellow and gradually turning dark brown at base, prementum gleaming black. Frons 1.2–1.3 times longer than facial length, about 0.2–0.3 times head width or 0.7 times eye width, frontal vitta parallel or slightly narrowing posteriorly, 0.55 times the width of fronto-orbital plate at middle; parafacial at narrowest point about as wide as postpedicel or slightly wider and bare below lowest frontal seta; genal height about 0.1 times eye height; 5–7 frontal setae, two frontal setae below antennal insertion, descending to parafacial and lowest seta descending to lowest level of pedicel, fronto-orbital plate with a row of 3–4 reclinate orbital setae and shorter setae on outer surface, ocellar setae absent, outer vertical seta hair-like and not distinctly longer than postocular setula, inner vertical seta about 0.3 times eye height; postocular setae short and straight. Face appreciably sunken with a low middle facial ridge, lower margin of face not protruding forward, facial ridge with short setae only on lower 1/5, vibrissa slightly inserted above lower facial margin, a row of 8–12 strong subvibrissae about 0.4 times as long as vibrissa. Occiput mostly with white hairs without a row of black setulae behind postocular row. Antenna with postpedicel about 3 times as long as pedicel, arista thickened on basal 2/5–1/2, first aristomere short, second aristomere same length as diameter. Palpus almost as long as postpedicel. Prementum 2.5–3.0 times longer than width, labellum large.

**Thorax** black in ground color, covered with grayish white pruinosity on both sides, and grayish white with sparse yellow pruinosity on dorsal side, 5 dark longitudinal vittae on scutum. Scutellum brown on apical half and black on basal half, 3+3 *ac*, 3+4 *dc*, 1+3 *ia*, 1+3 *sa*, first one about as long as notopleural seta and longer than postsutural intra-alar seta, postpronotal lobe with 3–4 setae (anterior one short and sometimes hard to separate with hairs around), 3 of them arranged in a straight line and anterior one located between inner two setae, scutellum with 5 pairs of marginal setae, apical scutellar setae crossed and about 2/3 as long as scutellum, 2 lateral scutellar setae about 0.7 times as long as subapical setae, with a pair of discal scutellar setae. Prosternum with setulae on both sides, proepisternum bare, anepisternum with 1 seta anteriorly and a row of 7 to 8 setae posteriorly, anepimeron with 2–3 setae, one of them strong, katepisternum with 4 setae, katepimeron at most with a few hairs on anterior fourth, anatergite bare.

**Wing** wholly hyaline and brownish, tegula black, basicosta dark brown. Calypters white, margins wholly yellowish, costal spine short and indistinct, 2nd and 3rd costal sectors bare ventrally, relative lengths of 2nd, 3rd and 4th costal sectors approximately in ratio of 57:84:50, base of R_4+5_ with one setula dorsally and ventrally, about same length as crossvein r-m, vein M curved at bending, vein M from bend to crossvein dm-cu longer than distance between bend and wing hind margin (23:15). Halter yellow on basal half and dark brown on distal half.

**Legs** dark brown, pulvilli yellowish, fore claw longer than 5th tarsomere. Fore tibia with a row of sparse short *ad*, 2 *p*, 1 short preapical *ad* and 1 preapical *d*, 1 long preapical *pv*. Mid tibia with 1 *ad*, 2 posterior (upper one about half the length of the lowest one) and 1 *v*. Hind tibia with a row of irregular long *ad* in which one is stronger, 1–3 *v* (only lowest one always strong), a row of irregular *pd*, 1 preapical dorsal and *ad*, 1 preapical *av*, preapical *pv* absent.

**Abdomen** long ovate, black in ground color dorsally, covered with yellowish gray pruinosity, a narrow black median vitta on tergites 3 (distinctly) to 5, reddish yellow on both sides of tergite 3. Mid-dorsal depression of syntergite 1+2 extending to its posterior margin, syntergite 1+2 and tergite 3 each with 2 short median marginal setae and a pair of lateral marginal setae, setae on syntergite 1+2 shorter than that on tergite 3, sometimes difficult to separate lateral marginal setae from hairs on syntergite, median discal and lateral discal setae absent on tergites 3 and 4, and a complete row of marginal setae present on tergite 4. Tergites 4 and 5 of male each with two large sexual hair patches of appressed hairs ventrally. Sternite 5 nearly square, lateral lobe with several setae without a long strong seta, bluntly rounded at apex, median U shape cleft about 1/2 length of sternite 5.

**Male terminalia** (Fig. 5). Cerci slender, evenly narrowed, slightly pointed at apex, narrowly separated on apical 1/2 in caudal view, nearly straight in lateral view, narrowed to apex. Surstylus about same length as cerci, bluntly rounded at apex in caudal view, slightly blunt in lateral view. Cerci about 2/3 surstyli width and with an obvious notch near middle in lateral view. Pregonite with several setae on dorsal surface; postgonite about 2.5 times epiphallus length, 2/3 pregonite width at middle; basiphallus and distiphallus distinctly sclerotized; distiphallus weakly expanded in lateral view; acrophallus slightly flattened at apex.

**Female**. Frons about 0.8–0.9 times eye width, parafacial at narrowest point about 0.8–0.9 times postpedicel width. Anepisternum with 4 setae anteriorly, one of them strong, and a row of 7 to 8 setae posteriorly. Fore claw distinctly shorter than 5th tarsomere. Tergites 4 and 5 without large sexual hair patches of appressed hairs ventrally. Other characteristics are the same as those of the male.

#### Diagnosis

Eye nearly bare, with sparse ommatrichia shorter than 2 facets in length, frons 1.2–1.3 times longer than facial length, about 0.7 times eye width, parafacial at narrowest point about as wide as postpedicel or slightly wider and bare below lowest frontal seta, genal height about 0.1 times eye height, two frontal setae descending to parafacial, ocellar seta absent, occiput without black setulae behind postocular row. Cerci narrowly separated on apical 1/2 in caudal view, about 2/3 surstyli width and with an obvious notch near middle in lateral view; surstylus about same length as cerci, bluntly rounded at apex in caudal view, slightly blunt in lateral view. Postgonite about 2.5 times epiphallus length, 2/3 pregonite width at middle.

#### Etymology

The epithet is from the type locality, Guangxi and Fujian in southern China. Latin *meridionalis* (= southern).

#### Distribution

China (Provinces Fujian, Guangxi).

#### Remarks

*Drino parafacialis* Chao & Liang, 1998 from China (Zhejiang) is closely similar to this new species, but is distinguished from the latter by having frons about 0.8 times eye width, occiput with sparse black setulae behind postocular row, cerci about 1/2 surstyli width and without an obvious notch near middle in lateral view (as in Fig. 38b).

### Drino oharai

Zhang & Liu sp. nov.

B645CD24-E51A-5A33-A4CD-6ABAEE204BFA

66EF3F80-370A-4355-A4EE-1C39B67446BB

#### Materials

- **Type status:** Holotype. **Occurrence:** recordedBy: Chuntian Zhang; individualCount: 1; sex: male; lifeStage: adult; occurrenceID: 4E63A8D3-68F4-575B-ACED-820ED533CABC; **Taxon:** scientificName: *Drino oharai*; **Location:** country: China; stateProvince: Hubei; locality: Dabie Mountains, Yingshan County; verbatimElevation: 400–900 m; verbatimCoordinates: 31°N, 116°E; decimalLatitude: 31; decimalLongitude: 116; **Identification:** identifiedBy: Chuntian Zhang; dateIdentified: 2014; **Event:** samplingProtocol: sweeping; eventDate: 29.Ⅵ.2014; **Record Level:** collectionCode: Insects
- **Type status:** Paratype. **Occurrence:** recordedBy: Shidi Wang & Zhe Zhao; individualCount: 1; sex: male; lifeStage: adult; occurrenceID: 5383A10B-22E2-5ACF-900E-7177129EC627; **Taxon:** scientificName: *Drino oharai*; **Location:** country: China; stateProvince: Shaanxi ; locality: Taibai Mountains, Baoji City; verbatimElevation: 600–2800 m; **Identification:** identifiedBy: Chuntian Zhang; dateIdentified: 2010; **Event:** samplingProtocol: sweeping; eventDate: 17–19.Ⅶ.2010; **Record Level:** collectionCode: Insects
- **Type status:** Paratype. **Occurrence:** recordedBy: Chuntian Zhang; individualCount: 1; sex: male; lifeStage: adult; occurrenceID: CBD9B7C6-98DE-5422-A651-3A95CC560F78; **Taxon:** scientificName: *Drino oharai*; **Location:** country: China; stateProvince: Zhejiang; locality: Shangliaokeng, Suichang County; verbatimElevation: 500–900m; **Identification:** identifiedBy: Chuntian Zhang; dateIdentified: 2013; **Event:** samplingProtocol: sweeping; eventDate: 4.Ⅶ.2013; **Record Level:** collectionCode: Insects
- **Type status:** Paratype. **Occurrence:** recordedBy: Qiang Wang; individualCount: 1; sex: male; lifeStage: adult; occurrenceID: 73AB4449-FF92-5E90-B449-61514E062D30; **Taxon:** scientificName: *Drino oharai*; **Location:** country: China; stateProvince: Shaanxi ; locality: Mt. Canglong, Shanyang County; verbatimElevation: 718 m; **Identification:** identifiedBy: Chuntian Zhang; dateIdentified: 2013; **Event:** samplingProtocol: sweeping; eventDate: 23.Ⅶ.2013; **Record Level:** collectionCode: Insects
- **Type status:** Paratype. **Occurrence:** recordedBy: Chuntian Zhang; individualCount: 1; sex: male; lifeStage: adult; occurrenceID: EBD2B80A-4D6E-52FE-BC2E-397CCF2A370C; **Taxon:** scientificName: *Drino oharai*; **Location:** country: China; stateProvince: Hubei; locality: Qingtaiguan, Luotian County; verbatimElevation: 600–950 m; verbatimCoordinates: 31°N, 115°E; decimalLatitude: 31; decimalLongitude: 115; **Identification:** identifiedBy: Chuntian Zhang; dateIdentified: 2014; **Event:** samplingProtocol: sweeping; eventDate: 3.Ⅶ.2014; **Record Level:** collectionCode: Insects
- **Type status:** Paratype. **Occurrence:** recordedBy: Chuntian Zhang; individualCount: 1; sex: male; lifeStage: adult; occurrenceID: AF3708B4-4048-56C1-8554-36B243D4AE81; **Taxon:** scientificName: *Drino oharai*; **Location:** country: China; stateProvince: Hubei; locality: Xiaoqiling, Yingshan County; verbatimElevation: 1050–1182 m; verbatimCoordinates: 30°N, 116°E; decimalLatitude: 30; decimalLongitude: 116; **Identification:** identifiedBy: Chuntian Zhang; dateIdentified: 2014; **Event:** samplingProtocol: sweeping; eventDate: 28.Ⅵ.2014; **Record Level:** collectionCode: Insects
- **Type status:** Paratype. **Occurrence:** recordedBy: Qiang Wang; individualCount: 1; sex: male; lifeStage: adult; occurrenceID: 5CDC6439-E5F6-589C-8624-5721DE2E84FA; **Taxon:** scientificName: *Drino oharai*; **Location:** country: China; stateProvince: Chengdu; locality: Mt. Qingcheng, Dujiangyan; verbatimElevation: 900–1100 m; verbatimCoordinates: 30°55'N 103°29'E; decimalLatitude: 30.92; decimalLongitude: 103.48; **Identification:** identifiedBy: Chuntian Zhang; dateIdentified: 2015; **Event:** samplingProtocol: sweeping; eventDate: 27–28.Ⅶ.2015; **Record Level:** collectionCode: Insects
- **Type status:** Paratype. **Occurrence:** recordedBy: Chuntian Zhang & Xinyi Li; individualCount: 1; sex: male; lifeStage: adult; occurrenceID: B853DC1F-3C20-5CE5-861A-A57DFF206C0C; **Taxon:** scientificName: *Drino oharai*; **Location:** country: China; stateProvince: Fujian; locality: Wuyi Mountains, Nanping City; verbatimElevation: 580–712 m; verbatimCoordinates: 27°42'N 117°43'E; decimalLatitude: 27.7; decimalLongitude: 117.7; **Identification:** identifiedBy: Chuntian Zhang; dateIdentified: 2022; **Event:** samplingProtocol: sweeping; eventDate: 29.Ⅵ.2022; **Record Level:** collectionCode: Insects
- **Type status:** Paratype. **Occurrence:** recordedBy: Chuntian Zhang & Xinyi Li; individualCount: 1; sex: male; lifeStage: adult; occurrenceID: 6741CC79-8574-5241-9B97-B734FD987C60; **Taxon:** scientificName: *Drino oharai*; **Location:** country: China; stateProvince: Fujian; locality: Jianyang, Nanping; verbatimElevation: 536–950 m; verbatimCoordinates: 27°40'N 117°36'E; decimalLatitude: 27.7; decimalLongitude: 117.6; **Identification:** identifiedBy: Chuntian Zhang; dateIdentified: 2022; **Event:** samplingProtocol: sweeping; eventDate: 1.Ⅶ.2022; **Record Level:** collectionCode: Insects
- **Type status:** Paratype. **Occurrence:** recordedBy: Chuntian Zhang & Xinyi Li; individualCount: 1; sex: male; lifeStage: adult; occurrenceID: 6E8FCFA7-0881-5DED-9EEB-CFFB301DB556; **Taxon:** scientificName: *Drino oharai*; **Location:** country: China; stateProvince: Fujian; locality: Wuyi Mountains, Nanping City; verbatimElevation: 630–680 m; verbatimCoordinates: 27°46'N 117°41'E; decimalLatitude: 27.8; decimalLongitude: 117.7; **Identification:** identifiedBy: Chuntian Zhang; dateIdentified: 2022; **Event:** samplingProtocol: sweeping; eventDate: 12.Ⅶ.2022; **Record Level:** collectionCode: Insects

#### Description

Body length 6.9–9.6 mm (Fig. 6a, b).

**Male**. Head black (Fig. 6c, d) in ground color. Eye nearly bare, with sparse ommatrichia shorter than 2 facets in length. Frontal vitta dark brown; fronto-orbital plate, parafacial, face, gena and occiput covered with grayish white pruinosity; lunule brown. Antenna covered with pale yellow pruinosity, postpedicel pale brown at base in ground color, palpus yellow and gradually turning dark brown at base, prementum gleaming black. Ocellar triangle about 5/9 length of frons width. Frons 1.3 times longer than facial length, about 0.2 times head width or 0.5 times eye width, frontal vitta parallel, 0.77 times the width of fronto-orbital plate at middle; parafacial at narrowest point about 0.7 times postpedicel width and bare below lowest frontal seta; genal height 0.1–0.2 times eye height; 9–11 frontal setae, 4–6 frontal setae below antennal insertion, descend to parafacial and lowest one descending to lowest level of pedicel, fronto-orbital plate with a row of 2–3 reclinate orbital setae and shorter setae on outer surface, ocellar seta absent, outer vertical seta hair-like and not distinctly longer than postocular setula, inner vertical seta about 0.5 eye height; postocular setae short and straight. Face appreciably sunken with a low middle facial ridge, lower margin of face not protruding forward, facial ridge with setae only on lower 1/5, vibrissa slightly inserted above lower facial margin, a row of 8–12 strong subvibrissae about 0.4 times as long as vibrissa. Occiput mostly with white hairs without a row of black setulae behind postocular row. Antenna with postpedicel about 3 times as long as pedicel, arista thickened on basal 1/3, first aristomere short, second aristomere same length as diameter. Palpus almost as long as postpedicel. Prementum 3.3 times longer than width, labellum large.

**Thorax** black in ground color, covered with grayish white pruinosity, 5 dark longitudinal vittae on scutum. Scutellum reddish yellow except for base black, 3+3 *ac*, 3+4 *dc*, 1+3 *ia*, 1+3 *sa*, first one about as long as notopleural seta and longer than postsutural intra-alar seta, postpronotal lobe with 3–4 setae, 3 of them arranged in a straight line and anterior one located between inner two setae, scutellum with 5 pairs of marginal setae, apical scutellar setae crossed and about 4/5 as long as scutellum, 2–3 lateral scutellar setae about 0.4–0.6 times as long as subapical setae, with a pair of discal scutellar setae. Prosternum with setulae on both sides, proepisternum bare, anepisternum almost with only hair anteriorly and a row of 7 to 8 setae posteriorly, anepimeron with 2–3 setae, one of them strong, katepisternum with 4 setae, katepimeron at most with a few hairs on anterior fourth, anatergite bare.

**Wing** wholly hyaline, tegula black, basicosta dark brown. Calypters white, margins wholly yellowish, costal spine short and indistinct, 2nd and 3rd costal sectors bare ventrally, relative lengths of 2nd, 3rd and 4th costal sectors approximately in ratio of 12:25:15, base of R_4+5_ with one setula dorsally and ventrally (sometimes with an additional short setula), about same length as crossvein r-m, vein M curved at bending, vein M from bend to crossvein dm-cu longer than distance between bend and wing hind margin (20:9). Halter yellow on basal half and dark brown on distal half.

**Legs** dark brown, pulvilli yellowish, fore claw longer than 5th tarsomere. Fore tibia with a row of sparse short *ad*, 2 *p*, 1 short preapical *ad* and 1 preapical *d*, 2 long preapical *pv*. Mid tibia with 1 *ad*, 2 posterior (upper one almost ½ length of lowest one) and 1 *v*. Hind tibia with a row of regular long *ad*, a line of *v* gradually strong on preapical side, a row of irregular *pd*, 1 preapical *d* and *ad*, 1 preapical *av*, preapical *pv* absent.

**Abdomen** long ovate, black in ground color dorsally, covered with yellowish gray pruinosity, a narrow black median vitta on tergites 3 (distinctly) to 5, reddish yellow on both sides of tergite 3. Mid-dorsal depression of syntergite 1+2 extending to its posterior margin, syntergite 1+2 and tergite 3 each with 2 short median marginal setae and a pair of lateral marginal setae, setae on syntergite 1+2 shorter than that on tergite 3, median discal and lateral discal setae absent on tergites 3 and 4, and a complete row of marginal setae present on tergite 4. Tergite 4 of male with two large sexual long hair patches of appressed hairs ventrally. Sternite 5 nearly square, lateral lobe with several setae about same length, bluntly rounded at apex, median U shape cleft about 2/5 length of sternite 5.

**Male terminalia** (Fig. 7). Cerci slender, evenly narrowed, slightly pointed at apex, narrowly separated on apical 2/5 in caudal view, about 5/4 surstyli width at middle in lateral view. Surstylus about 2/3 cerci length, bluntly rounded at apex in caudal view, slightly blunt in lateral view. Pregonite with several setae on dorsal surface; postgonite about 1.5 times epiphallus length, as wide as pregonite at middle; epiphallus long and thin; basiphallus and distiphallus distinctly sclerotized; distiphallus weakly expanded in lateral view; acrophallus round at apex.

**Female**. Unknown.

#### Diagnosis

Eye nearly bare, with sparse ommatrichia shorter than 2 facets in length, ocellar triangle about 5/9 frons width, frons 1.3 times longer than facial length, about 0.5 times eye width, parafacial at narrowest point about 0.7 times postpedicel width and bare below lowest frontal seta, genal height about 0.1–0.2 times eye height, 4–6 frontal setae descending to parafacial, ocellar seta absent. Cerci narrowly separated on apical 2/5 in caudal view, about 1.25 times surstyli width at middle in lateral view; surstylus about 2/3 cerci length, bluntly rounded at apex in caudal view, slightly blunt in lateral view. Postgonite about 1.5 times epiphallus length, as wide as pregonite at middle.

#### Etymology

The specific name is dedicated to Dr. James E. O’Hara, for his great contributions to the systematics of Tachinidae of the world.

#### Distribution

China (Provinces Fujian, Hubei, Shaanxi, Sichuan, Zhejiang).

#### Remarks

*Drino minuta* Chao & Liang, 1998 from China (Guangdong) is closely similar to this new species, but is distinguished from the latter by having frons 0.8–0.9 times eye width, ocellar triangle about 2/3 frons width, parafacial at narrowest point slightly wider than antenna.

### Drino uniseta

Zhang & Liu sp. nov.

A93B683F-C224-5360-884E-465EEC2F0331

6057F028-8C7E-455D-9435-AC7FBD98B1BC

#### Materials

- **Type status:** Holotype. **Occurrence:** recordedBy: Peng Hou; individualCount: 1; sex: male; lifeStage: adult; occurrenceID: 133591B0-CF8D-5BFB-B51C-EB9F9D5CC3DA; **Taxon:** scientificName: *Drino uniseta*; **Location:** country: China; stateProvince: Hubei; locality: Taohuachong, Yingshan County, Huanggang City; verbatimElevation: 600–700 m; **Identification:** identifiedBy: Chuntian Zhang; dateIdentified: 2014; **Event:** samplingProtocol: sweeping; eventDate: 23.Ⅵ.2014; **Record Level:** collectionCode: Insects
- **Type status:** Paratype. **Occurrence:** recordedBy: Qi Sun; individualCount: 1; sex: male; lifeStage: adult; occurrenceID: D4CA1ED5-D95F-56C7-8AF4-7AB9B72C9BEB; **Taxon:** scientificName: *Drino uniseta*; **Location:** country: China; stateProvince: Zhejiang; locality: Nianbadu, Jiangshan County, Quzhou City; verbatimElevation: 450–517 m; verbatimCoordinates: 28.19°N, 118.36°E; decimalLatitude: 28.19; decimalLongitude: 118.36; **Identification:** identifiedBy: Chuntian Zhang; dateIdentified: 2016; **Event:** samplingProtocol: sweeping; eventDate: 8–9.Ⅷ.2016; **Record Level:** collectionCode: Insects
- **Type status:** Paratype. **Occurrence:** recordedBy: Chuntian Zhang; individualCount: 1; sex: male; lifeStage: adult; occurrenceID: 8EB97EF9-086F-5038-BE94-BC1D2827737A; **Taxon:** scientificName: Drino *un*iseta; **Location:** country: China; stateProvince: Fujian; locality: Zhupai, Wuyi mountains; verbatimElevation: 631 m; **Identification:** identifiedBy: Chuntian Zhang; dateIdentified: 2021; **Event:** samplingProtocol: sweeping; eventDate: 27.V.2021; **Record Level:** collectionCode: Insects

#### Description

Body length 11.7–12.0 mm (Fig. 8a, b).

**Male**. Head black (Fig. 8c, d) in ground color. Eye nearly bare, with sparse ommatrichia shorter than 2 facets in length. Frontal vitta dark brown; fronto-orbital plate with dingy pale yellowish pruinosity especially on upper half; parafacial, face, gena and occiput covered with grayish white pruinosity; lunule brown. Antenna covered with pale yellowish pruinosity, postpedicel pale brown at base in ground color, palpus dark brown and gradually darkened at base, prementum gleaming black. Frons 1.4–1.6 times longer than facial length, about 0.3 times head width or 0.8–0.9 times eye width, frontal vitta slightly narrowed posteriorly, about 0.83 times the width of fronto-orbital plate at middle; parafacial at narrowest point about 1.6–1.7 times postpedicel width and bare below lowest frontal seta; genal height 0.1–0.2 times eye height; 6–8 frontal setae, three frontal setae below antennal insertion, descending to parafacial and lowest one descending to lowest level of pedicel, fronto-orbital plate with a row of 3 reclinate orbital setae and shorter setae on outer surface, ocellar seta absent, outer vertical seta hair-like and not distinctly longer than postocular setula, inner vertical setae about 0.5 times eye height; postocular setae short and straight. Face appreciably sunken with a low middle facial ridge, lower margin of face not protruding forward, facial ridge with setae only on lower 1/5, vibrissa slightly inserted above lower facial margin, a row of 9–12 strong subvibrissae about 0.4 times as long as vibrissa. Occiput on upper half mostly with white hairs with 3–7 sparse black setulae behind postocular row. Antenna with postpedicel about 3.2 times as long as pedicel, arista thickened on basal 2/5, first aristomere short, second aristomere 1.5 times longer than diameter. Palpus about 1.1 times as long as postpedicel. Prementum 3.5 times longer than width, labellum large.

**Thorax** black in ground color, covered with grayish white pruinosity on both sides, and grayish white with sparse yellow pruinosity on dorsal side, 5 dark longitudinal vittae on scutum. Scutellum light yellow except for base black, 3+3 *ac*, 3+4 *dc*, 1+3 *ia*, 1+3 *sa*, first one about as long as notopleural seta and longer than postsutural intra-alar seta, postpronotal lobe with 4 setae, anterior one short, 3 of them arranged in a straight line and anterior one located between inner two setae, scutellum with 5 pairs of marginal setae, apical scutellar setae crossed and about 0.8–1.1 times as long as scutellum, 2 lateral scutellar setae about 0.6–0.9 times as long as subapical setae, with a pair of discal scutellar setae. Prosternum with setulae on both sides, proepisternum bare, anepisternum with 1 seta anteriorly and a row of 7 to 8 setae posteriorly, anepimeron with 3 setae, one of them strong, katepisternum with 4 setae, katepimeron at most with a few hairs on anterior half, anatergite bare.

**Wing** wholly hyaline, tegula black, basicosta dark brown. Calypters white, margins wholly yellowish, costal spine short and indistinct, 2nd and 3rd costal sectors bare ventrally, relative lengths of 2nd, 3rd and 4th costal sectors approximately in ratio of 7:10:6, base of R_4+5_ with one setula dorsally and ventrally, about same length as crossvein r-m, vein M curved at bending, vein M from bend to crossvein dm-cu longer than distance between bend and wing hind margin (5:3). Halter yellow on basal half and dark brown on distal half.

**Legs** dark brown, pulvilli yellowish, fore claw longer than 5th tarsomere. Fore tibia with a row of sparse short *ad*, 2 *p*, 1 short preapical *ad* and 1 preapical *d*, 1 long preapical *pv*. Mid tibia with 1 *ad*, 2 posterior (basal one almost 1/2 length of distal one) and 1 *v*. Hind tibia with a row of irregular long *ad* in which one is stronger, 2–3 *v* (only distal one always strong), a row of irregular *pd*, 1 preapical dorsal and *ad*, 1 preapical *av*, preapical *pv* absent.

**Abdomen** long ovate, black in ground color, covered with yellowish gray pruinosity, a narrow black median vitta on tergites 3 (distinctly) to 5, reddish yellow on both sides of syntergite 1+2 to tergite 4. Mid-dorsal depression of syntergite 1+2 extending to its posterior margin, syntergite 1+2 and tergite 3 each with 2 short median marginal setae and a pair of lateral marginal setae, setae on syntergite 1+2 shorter than that on tergite 3, median discal and lateral discal setae absent on tergites 3 and 4, and a complete row of marginal setae present on tergite 4. Tergites 4 and 5 of male each with two large sexual hair patches of appressed hairs ventrally. Sternite 5 square, lateral lobe with one distinctly long and strong seta and some weaker setae, bluntly rounded at apex, median U-shaped cleft about 3/5 length of sternite 5.

**Male terminalia** (Fig. 9). Cerci evenly narrowed, slightly pointed at apex, narrowly separated on apical 2/5 in caudal view, about 2/3 surstyli width at middle in lateral view, nearly straight and both sides are almost parallel, then narrowed sharply just before the tip in lateral view, narrowed to apex. Surstylus about same length as cerci, bluntly rounded at apex in caudal view, slightly blunt in lateral view. Pregonite with several setae on dorsal surface; postgonite about 2 times epiphallus length, 2/5 pregonite width at middle; pregonite and postgonite pointing in almost same direction; epiphallus short and hook-like, shorter than postgonite; basiphallus and distiphallus distinctly sclerotized; distiphallus elongate in lateral view; acrophallus round at apex. Lateral lobe of 5th sternite distinctly with only a long strong seta at apex.

**Female**. Unknown.

#### Diagnosis

Eye nearly bare, with sparse ommatrichia shorter than 2 facets in length, frons 1.4–1.6 times longer than facial length, about 0.3 times head width, parafacial at narrowest point about 1.6–1.7 times postpedicel width and bare below lowest frontal seta, parafacial width at scape level longer than gena height in lateral view, genal height 0.1–0.2 times eye height, 3 frontal setae descending to parafacial, ocellar seta absent, pregonite and postgonite pointing in almost the same direction, lateral lobe of 5th sternite distinctly with only a long strong seta at apex. Cerci narrowly separated on apical 2/5 in caudal view, about 2/3 surstyli width at middle in lateral view; surstylus about as long as cerci, bluntly rounded at apex in caudal view, slightly blunt in lateral view. Postgonite about 2 times epiphallus length, 2/5 pregonite width at middle. Distiphallus elongate in lateral view.

#### Etymology

The specific name is derived from a diagnostic character of this species, lateral lobe of 5th sternite distinctly with only a long strong seta at apex. Latin *unus* (one) + *seta* (bristle) (*uniseta* = a single seta).

#### Distribution

China (Provinces Fujian, Hubei, Zhejiang).

#### Remarks

*Drino parafacialis* Chao & Liang, 1998 from China (Zhejiang) is closely similar to this new species, but is distinguished from the latter by having parafacial at narrowest point only slightly wider than postpedicel, pregonite and postgonite pointing in different directions at an angle of about 60°, cerci about 1/2 surstyli width at middle in lateral view (as in Fig. 38b).

### Drino adiscalis

(Chao, 1982)

85DFD2AD-8DA2-5BB3-B164-F4A3F368D103

Lydella adiscalis Chao in Chao and Shi, 1982. – Chao and Shi 1982: 272. Type material deposited in IZCAS, examined.
Drino adiscalis : Chao et al. 1998: 1830. – Xue and Wang 2006: 251 (Catalog). – O’Hara et al. 2009: 66 (Catalog). – Zhang 2016: 243, 244 (key and descriptions). – O’Hara et al. 2020a: 315 (Catalog). – O’Hara et al. 2020: 879 (Catalog).

#### Materials

- **Type status:** Holotype. **Occurrence:** recordedBy: Xuezhong Zhang; individualCount: 1; sex: male; lifeStage: adult; occurrenceID: 9449CD6C-8ACC-5109-8C19-064B44E4F9AF; **Taxon:** scientificName: *Drino adiscalis*; **Location:** country: China; stateProvince: Xizang Autonomous Region; locality: Gyamda, Xizang; verbatimElevation: 3400m; **Identification:** identifiedBy: Jianming Zhao; dateIdentified: 1976; **Event:** eventDate: 23.Ⅶ.1976; **Record Level:** collectionCode: Insects
- **Type status:** Allotype. **Occurrence:** recordedBy: Xuezhong Zhang; individualCount: 1; sex: male; lifeStage: adult; occurrenceID: 26343820-395E-5421-A497-710AB0285B31; **Taxon:** scientificName: *Drino adiscalis*; **Location:** country: China; stateProvince: Xizang Autonomous Region; locality: Gyamda, Xizang; verbatimElevation: 3400m; **Identification:** identifiedBy: Jianming Zhao; dateIdentified: 1976; **Event:** eventDate: 23.Ⅶ.1976; **Record Level:** collectionCode: Insects
- **Type status:** Paratype. **Occurrence:** recordedBy: Xuezhong Zhang et Yinheng Han leg; individualCount: 18; sex: male; lifeStage: adult; occurrenceID: 45056A04-B65F-53A0-9C7B-45BA0E23C665; **Taxon:** scientificName: *Drino adiscalis*; **Location:** country: China; stateProvince: Xizang Autonomous Region; locality: Gyamda, Chagyab; verbatimElevation: 3400–3600m; **Identification:** identifiedBy: Jianming Zhao; dateIdentified: 1976; **Event:** eventDate: 5–29.Ⅶ.1976; **Record Level:** collectionCode: Insects
- **Type status:** Paratype. **Occurrence:** recordedBy: Xuezhong Zhang et Yinheng Han leg; individualCount: 15; sex: female; lifeStage: adult; occurrenceID: 3D64F4E9-1B3E-5B1A-95B9-1BD457FCFF81; **Taxon:** scientificName: *Drino adiscalis*; **Location:** country: China; stateProvince: Xizang Autonomous Region; locality: Gyamda, Chagyab; verbatimElevation: 3400–3600m; **Identification:** identifiedBy: Jianming Zhao; dateIdentified: 1976; **Event:** eventDate: 5–29.Ⅶ.1976; **Record Level:** collectionCode: Insects
- **Type status:** Other material. **Occurrence:** recordedBy: Chuntian Zhang & Xinyi Li; individualCount: 1; sex: female; lifeStage: adult; occurrenceID: 2BEAD29C-6205-5EF6-A3DF-1DF140166A8F; **Taxon:** scientificName: *Drino adiscalis*; **Location:** country: China; stateProvince: Qinghai; locality: Binggou, Babaozhen, Qilian County; verbatimElevation: 3059 m; verbatimCoordinates: 38°08'N 100°10'E; decimalLatitude: 38.1; decimalLongitude: 100.1; **Identification:** identifiedBy: Chuntian Zhang; dateIdentified: 2020; **Event:** samplingProtocol: sweeping; eventDate: 4.Ⅷ.2020; **Record Level:** collectionCode: Insects

#### Diagnosis

Body length (Fig. 10a, b) 7.0–10.0 mm. Frons (Fig. 10c, d) 1.5 times eye width, frontal vitta 1.2 times wider than fronto-orbital plate at middle, genal height 0.27 times eye height, antenna with postpedicel about 2.7 times as long as pedicel, ocellar triangle about 1/2 frons width, 3–4 reclinate orbital setae, outer vertical seta about 1/2 as long as inner vertical seta, occiput on upper half mostly with white hairs and a dense row of black setulae behind postocular row. Scutellum reddish yellow except for base black, with 2 lateral scutellar setae. Relative lengths of 2nd, 3rd and 4th costal sectors approximately in ratio of 5:7:4, vein M from bend to crossvein dm-cu shorter than distance between bend and wing hind margin (18:29), halter yellow on basal half and dark brown on distal half. Mid tibia with 4–5 *ad*, 2 *p* and 2 *v*. Tergites 4 and 5 with large sexual hair patches of appressed hairs ventrally. Cerci (Fig. 11a) slender, evenly narrowed, slightly pointed at apex, narrowly separated on apical 2/5 in caudal view, in lateral view cerci nearly straight, narrowed to apex. Surstylus about same length as cerci, slightly pointed at apex in caudal view, slightly blunt in lateral view. Pregonite (Fig. 11b) longer than distiphallus and slightly bent; postgonite slender; basiphallus and distiphallus distinctly sclerotized; distiphallus weakly expanded in lateral view.

#### Distribution

China (Provinces Liaoning, Neimenggu, Sichuan, Xinjiang, Xizang).

#### Remarks

We have examined the holotype deposited in IZCAS. *D. latifrons* Liu, Zhang & Xi, 2023 is similar to this species but differs in having fronto-orbital plate with a row of 5–7 reclinate orbital setae, mid tibia with 3–4 *ad*, 3 *p* and 2 *v*, surstyli shorter than cerci in lateral view.

### Drino angustivitta

Liang & Chao, 1998

C3022024-2D1B-53A9-93DF-C319FEADAF93

Drino angustivitta Liang & Chao in Chao et al., 1998. – Chao et al. 1998: 1830. – O’Hara et al. 2009: 66 (Catalog). – O’Hara et al. 2020a: 315 (Catalog). – O’Hara et al. 2020: 879 (Catalog). Type material deposited in IZCAS, examined.

#### Materials

- **Type status:** Holotype. **Occurrence:** recordedBy: Zhaoke Liang; individualCount: 1; sex: male; lifeStage: adult; occurrenceID: C537E711-62F3-5BFD-BE3D-E18F156FEB1A; **Taxon:** scientificName: *Drino angustibitta*; **Location:** country: China; stateProvince: Hainan; locality: Wuzhi mountains; **Identification:** identifiedBy: Jianming Zhao; dateIdentified: 1982; **Event:** eventDate: 12.Ⅵ.1982; **Record Level:** collectionCode: Insects
- **Type status:** Paratype. **Occurrence:** recordedBy: Zhaoke Liang; individualCount: 1; sex: male; lifeStage: adult; occurrenceID: 5843D6E6-15AC-52F5-B5C5-D54C22469CFC; **Taxon:** scientificName: *Drino angustibitta*; **Location:** country: China; stateProvince: Hainan; locality: Wuzhi mountains; **Identification:** identifiedBy: Jianming Zhao; dateIdentified: 1982; **Event:** eventDate: 12.Ⅵ.1982; **Record Level:** collectionCode: Insects
- **Type status:** Paratype. **Occurrence:** recordedBy: Baolin Zhang; individualCount: 1; sex: male; lifeStage: adult; occurrenceID: B20DFEBF-05A7-5179-A70F-DFEF57F5AF0C; **Taxon:** scientificName: *Drino angustibitta*; **Location:** country: China; stateProvince: Hainan; locality: Xinglong County; **Identification:** identifiedBy: Jianming Zhao; dateIdentified: 1963; **Event:** eventDate: 6.Ⅴ.1963; **Record Level:** collectionCode: Insects
- **Type status:** Paratype. **Occurrence:** recordedBy: Zide Fan; individualCount: 1; sex: male; lifeStage: adult; occurrenceID: 04730FDE-ED79-5C8D-86B6-1752E290AB1E; **Taxon:** scientificName: *Drino angustibitta*; **Location:** country: China; stateProvince: Hunan; locality: Jianyang City; **Identification:** identifiedBy: Jianming Zhao; dateIdentified: 1957; **Event:** eventDate: 4–5.Ⅷ.1957; **Record Level:** collectionCode: Insects
- **Type status:** Other material. **Occurrence:** recordedBy: Chuntian Zhang & Xinyi Li; individualCount: 1; sex: female; lifeStage: adult; occurrenceID: 54DA0573-57AF-5061-88C4-457C7794082E; **Taxon:** scientificName: *Drino angustibitta*; **Location:** country: China; stateProvince: Fujian; locality: Tongmucun Village, Wuyi mountains; verbatimElevation: 510–630 m; verbatimCoordinates: 27°46'N 117°41'E; decimalLatitude: 27.7; decimalLongitude: 117.7; **Identification:** identifiedBy: Chuntian Zhang; dateIdentified: 2022; **Event:** samplingProtocol: sweeping; eventDate: 26.Ⅵ.2022; **Record Level:** collectionCode: Insects
- **Type status:** Other material. **Occurrence:** recordedBy: Chuntian Zhang & Xinyi Li; individualCount: 1; sex: female; lifeStage: adult; occurrenceID: 79EDD806-AAC9-5B34-AC8B-78E1A64ECC84; **Taxon:** scientificName: *Drino angustibitta*; **Location:** country: China; stateProvince: Hunan; locality: Dupangling Nature Reserve, Yongzhou City; verbatimElevation: 380–460 m; verbatimCoordinates: 25°27'N 111°22'E; decimalLatitude: 25.45; decimalLongitude: 111.37; **Identification:** identifiedBy: Chuntian Zhang; dateIdentified: 2020; **Event:** samplingProtocol: sweeping; eventDate: 1–4.Ⅸ.2020; **Record Level:** collectionCode: Insects
- **Type status:** Other material. **Occurrence:** recordedBy: Shidie Liu; individualCount: 3; sex: female; lifeStage: adult; occurrenceID: 1F13D831-FCF3-5246-81BE-BB8728C8A5B5; **Taxon:** scientificName: *Drino angustibitta*; **Location:** country: China; stateProvince: Hunan; locality: Longzitou, Longnan, Ganzhou City; verbatimElevation: 660 m; verbatimCoordinates: 24°43'N 114°44'E; decimalLatitude: 24.7; decimalLongitude: 114.7; **Identification:** identifiedBy: Chuntian Zhang; dateIdentified: 2021; **Event:** samplingProtocol: sweeping; eventDate: 7.Ⅵ.2021; **Record Level:** collectionCode: Insects

#### Diagnosis

Body length (Fig. 12a, b) 8.0–11.0 mm. Antenna covered with pale yellowish pruinosity, postpedicel pale brown in ground color. Frons (Fig. 12c, d) of male 0.6 times eye width, frontal vitta about 1/2 as wide as fronto-orbital plate at middle or less, parafacial at narrowest point about 1.4 times postpedicel width, occiput on upper half mostly with white hairs and without black setulae behind postocular row, arista thickened at basal 4/9. Relative lengths of 2nd, 3rd and 4th costal sectors approximately in ratio of 10:13:8. Mid tibia with 1 *ad*, 3 *p* and 1 *v*. Tergite 5 with dense pruinosity on basal 3/5, tergites 4 and 5 without sexual hair patches of appressed hairs ventrally. Cerci (Fig. 13a) slender, evenly narrowed, apparently pointed at apex, narrowly separated on apical 1/2 in caudal view, nearly straight in lateral view, pointed to apex. Surstylus slightly shorter than cerci, slightly pointed at apex in caudal view, slightly blunt in lateral view. Pregonite (Fig. 13b) with a distinct bend; basiphallus and distiphallus distinctly sclerotized; distiphallus weakly expanded in lateral view and distinctly longer than pregonite.

#### Distribution

China (Provinces Fujian, Hainan, Hunan).

#### Remarks

We have examined the holotype deposited in IZCAS. *D. argenticeps* (Macquart, 1851) is similar to this species but differs in having frons of male less than half the eye width, tergite 5 covered with dense pruinosity on basal half, and cerci rounded at apex.

### Drino argenticeps

(Macquart, 1851)

C6AD6FDD-7FF5-5C28-8244-322D1E059ED9

Masicera argenticeps Macquart, 1851. – Macquart 1851: 166 [also 1851: 193]. – Crosskey 1971: 273 (notes). Holotype ♂ in MNHN, external morphology examined via photographs.
Sturmia (Sturmia) vicinella Baranov, 1932. – Baranov 1932: 79.
Drino argenticeps : Mesnil 1949a: 11 (revision). – Mesnil 1951: 158, 162 (key and descriptions). – Crosskey 1976: 237 (Catalog). – Crosskey 1977: 675 (Catalog). – Herting 1984: 53 (Catalog). – Herting and Dely-Draskovits 1993: 207 (Catalog). – Chao et al. 1998: 1830 (key and descriptions). – O’Hara et al. 2009: 66 (Catalog). – O’Hara et al. 2020a: 315 (Catalog). – O’Hara et al. 2020: 879 (Catalog).

#### Materials

- **Type status:** Holotype. **Occurrence:** individualCount: 1; sex: male; lifeStage: adult; occurrenceID: 8A9AF6BC-C000-54B4-B6A3-7B28D53EC89D; **Taxon:** scientificName: *Masicera argenticeps*; **Location:** country: South-East Asia; **Identification:** identifiedBy: Macquart; **Record Level:** collectionCode: Insects
- **Type status:** Other material. **Occurrence:** recordedBy: Chuntian Zhang; individualCount: 1; sex: male; lifeStage: adult; occurrenceID: E931AB52-7FE6-5CAE-A9AB-BCAE593F5B2A; **Taxon:** scientificName: *Drino argenticeps*; **Location:** country: China; stateProvince: Fujian; locality: Zhupai, Wuyi Mountains; verbatimElevation: 631 m; **Identification:** identifiedBy: Chuntian Zhang; dateIdentified: 2021; **Event:** samplingProtocol: sweeping; eventDate: 27.Ⅴ.2021; **Record Level:** collectionCode: Insects
- **Type status:** Other material. **Occurrence:** recordedBy: Chuntian Zhang & Xinyi Li; individualCount: 1; sex: male; lifeStage: adult; occurrenceID: 3FF54769-D642-56ED-AF75-15BB3753DCB5; **Taxon:** scientificName: *Drino argenticeps*; **Location:** country: China; stateProvince: Fujian ; locality: Wuyi Mountains; verbatimElevation: 580–712 m; verbatimCoordinates: 27°42'N 117°43'E; decimalLatitude: 27.7; decimalLongitude: 117.71; **Identification:** identifiedBy: Chuntian Zhang; dateIdentified: 2022; **Event:** samplingProtocol: sweeping; eventDate: 29.Ⅵ.2022; **Record Level:** collectionCode: Insects
- **Type status:** Other material. **Occurrence:** recordedBy: Min Wang; individualCount: 1; sex: male; lifeStage: adult; occurrenceID: A6828F16-25EC-5DE3-B702-6793CC622F29; **Taxon:** scientificName: *Drino argenticeps*; **Location:** country: China; stateProvince: Guangdong; locality: Yuenanling Nature Reserve; **Identification:** identifiedBy: Chuntian Zhang; dateIdentified: 2011; **Event:** samplingProtocol: sweeping; eventDate: 18–21.Ⅴ.2011; **Record Level:** collectionCode: Insects
- **Type status:** Other material. **Occurrence:** recordedBy: Shidie Liu; individualCount: 2; sex: male; lifeStage: adult; occurrenceID: F433AB49-8F47-5270-96FE-9869075C866D; **Taxon:** scientificName: *Drino argenticeps*; **Location:** country: China; stateProvince: Guangdong; locality: Chebaling Nature Reserve, Shixing, Shaoguan City; verbatimElevation: 495–570 m; verbatimCoordinates: 24°42'N 114°10'E; decimalLatitude: 24.70; decimalLongitude: 114.16; **Identification:** identifiedBy: Chuntian Zhang; dateIdentified: 2021; **Event:** samplingProtocol: sweeping; eventDate: 1–3.Ⅵ.2021; **Record Level:** collectionCode: Insects
- **Type status:** Other material. **Occurrence:** recordedBy: Chuntian Zhang; individualCount: 1; sex: male; lifeStage: adult; occurrenceID: 73FF6865-F99B-5E47-BA22-FE0B64DF25F7; **Taxon:** scientificName: *Drino argenticeps*; **Location:** country: China; stateProvince: Guagnxi; locality: Mt. Maoer, Guilin City; verbatimElevation: 350–1180–2100 m; **Identification:** identifiedBy: Chuntian Zhang; dateIdentified: 2004; **Event:** samplingProtocol: sweeping; eventDate: 4.Ⅴ–29.Ⅳ.2004; **Record Level:** collectionCode: Insects
- **Type status:** Other material. **Occurrence:** recordedBy: Bo Hao; individualCount: 2; sex: male; lifeStage: adult; occurrenceID: 31E854AA-1181-5018-B3E5-3B0B5B74823F; **Taxon:** scientificName: *Drino argenticeps*; **Location:** country: China; stateProvince: Jiangxi; locality: Jiulian Mountains Nature Reserve; verbatimElevation: 490–587 m; verbatimCoordinates: 24°57'N 114°43'E; decimalLatitude: 24.95; decimalLongitude: 114.71; **Identification:** identifiedBy: Chuntian Zhang; dateIdentified: 2020; **Event:** samplingProtocol: sweeping; eventDate: 20–22.Ⅷ.2020; **Record Level:** collectionCode: Insects
- **Type status:** Other material. **Occurrence:** recordedBy: Ying Zhao; individualCount: 1; sex: male; lifeStage: adult; occurrenceID: 911DEC61-F1F7-51C9-B9BC-CFE8F2E3CB29; **Taxon:** scientificName: *Drino argenticeps*; **Location:** country: China; stateProvince: Liaoning; locality: Mt. Huangyi, Kuandian County, Dandong City; verbatimElevation: 395–533 m; verbatimCoordinates: 40°43'N 124°45'E; decimalLatitude: 40.71; decimalLongitude: 124.75; **Identification:** identifiedBy: Chuntian Zhang; dateIdentified: 2018; **Event:** samplingProtocol: sweeping; eventDate: 23–24.Ⅴ.2018; **Record Level:** collectionCode: Insects
- **Type status:** Other material. **Occurrence:** recordedBy: Chuntian Zhang; individualCount: 1; sex: female; lifeStage: adult; occurrenceID: 0F16F1FF-C3F0-5FD1-9195-5F208693630C; **Taxon:** scientificName: *Drino argenticeps*; **Location:** country: China; stateProvince: Sichuan; locality: Daochengkasi; verbatimElevation: 2750–3000 m; **Identification:** identifiedBy: Chuntian Zhang; dateIdentified: 2006; **Event:** samplingProtocol: sweeping; eventDate: 2.Ⅶ.2006; **Record Level:** collectionCode: Insects
- **Type status:** Other material. **Occurrence:** recordedBy: Bo Hao; individualCount: 1; sex: male; lifeStage: adult; occurrenceID: E775B500-E2EC-5402-865B-49452635B2CC; **Taxon:** scientificName: *Drino argenticeps*; **Location:** country: China; stateProvince: Yunnan; locality: Gaoligong Mountains, Gongshan County; verbatimElevation: 1353–3013 m; verbatimCoordinates: 27°80'N 98°50'E; decimalLatitude: 28.33; decimalLongitude: 98.83; **Identification:** identifiedBy: Chuntian Zhang; dateIdentified: 2015; **Event:** samplingProtocol: sweeping; eventDate: 12–15.Ⅴ.2015; **Record Level:** collectionCode: Insects
- **Type status:** Other material. **Occurrence:** recordedBy: Chuntian Zhang; individualCount: 1; sex: female; lifeStage: adult; occurrenceID: 88CABF65-B218-5538-B307-86FFDC81852D; **Taxon:** scientificName: *Drino argenticeps*; **Location:** country: China; stateProvince: Yunnan; locality: Shuidiping, Lanping County, Nujiang Autonomous Prefecture; verbatimElevation: 1441 m; verbatimCoordinates: 26°29'N 99°13'E; decimalLatitude: 26.48; decimalLongitude: 99.21; **Identification:** identifiedBy: Chuntian Zhang; dateIdentified: 2015; **Event:** samplingProtocol: sweeping; eventDate: 18.Ⅴ.2015; **Record Level:** collectionCode: Insects

#### Diagnosis

Body length (Fig. 14a, b) 11.0 mm. Frons (Fig. 14c) of male less than 0.5 times eye width, occiput on upper half mostly with white hairs and without a row of black setulae behind postocular row, antenna with postpedicel about 3 times pedicel length, arista slightly thickened on basal 1/3. Scutellum totally reddish yellow. Lateral scutellar setae about 0.6 times subapical setae length. Vein M bluntly bent, vein M from bend to crossvein dm-cu 2 times the distance from bend to hind margin of wing. Halter brown on basal half and black on apical half. Mid tibia with 1 *ad*, 2 *p* and 1 *v*. Tergite 3 with a black longitudinal stripe at base about 1/2 transverse width on its middle, tergite 4 with pruinose band 1/2 its length, without large sexual hair patches of appressed hairs ventrally. (Photos of holotype can be found in Global Biodiversity Information Facility (2025b). Postabdomen of male as in Fig. 15a and Fig. 15b.

#### Distribution

China (Provinces Fujian, Guangdong, Guangxi, Guizhou, Hainan, Jiangxi, Liaoning, Sichuan, Taiwan, Xizang, Yunnan, Zhejiang).

#### Remarks

We examined the outlook photos of the holotype of *Masicera argenticeps* Macquart deposited in MNHN. *D. angustivitta* Liang & Chao, 1998 is similar to this species but differs in having frons of male less than 0.6 times eye width, tergite 5 covered with dense pruinosity on basal 3/5, cercus apparently pointed at apex in lateral and caudal views.

### Drino auripollinis

Chao & Liang, 1998

080CF80C-1B16-547D-81E9-689D1B6BA042

Drino auripollinis Chao & Liang in Chao et al., 1998. – Chao et al. 1998: 1835. – Liu et al. 1998: 171 (key and descriptions). – Xue and Wang 2006: 251 (Catalog). – O’Hara et al. 2009: 66 (Catalog). – O’Hara et al. 2020a: 315 (Catalog). – O’Hara et al. 2020: 879 (Catalog). Type material deposited in IZCAS, examined.

#### Materials

- **Type status:** Holotype. **Occurrence:** recordedBy: Xuezhong Zhang; individualCount: 1; sex: male; lifeStage: adult; occurrenceID: BF44422E-2635-5213-9B51-8EE68B7FE44C; **Taxon:** scientificName: *Drino auripollinis*; **Location:** country: China; stateProvince: Yunnan; locality: Lushui City; verbatimElevation: 2100 m; **Identification:** identifiedBy: Jianming Zhao; dateIdentified: 1981; **Event:** eventDate: 19.Ⅴ.1981; **Record Level:** collectionCode: Insects
- **Type status:** Paratype. **Occurrence:** recordedBy: Xuekui Sun & Hong Liu; individualCount: 2; sex: male; lifeStage: adult; occurrenceID: F2280858-F990-5A8F-9A8B-4CA19F057659; **Taxon:** scientificName: *Drino auripollinis*; **Location:** country: China; stateProvince: Hunan; locality: Mt. Tianping, Sangzhi County; verbatimElevation: 1350–1400 m; **Identification:** identifiedBy: Jianming Zhao; dateIdentified: 1988; **Event:** eventDate: 12.Ⅷ.1988; **Record Level:** collectionCode: Insects
- **Type status:** Paratype. **Occurrence:** recordedBy: Xuekui Sun; individualCount: 1; sex: female; lifeStage: adult; occurrenceID: 8EF2B92E-FEC8-53A5-9B80-3EE466012797; **Taxon:** scientificName: *Drino auripollinis*; **Location:** country: China; stateProvince: Hunan; locality: Sangzhi; verbatimElevation: 1400 m; **Identification:** identifiedBy: Jianming Zhao; dateIdentified: 1988; **Event:** eventDate: 12.Ⅷ.1988; **Record Level:** collectionCode: Insects
- **Type status:** Paratype. **Occurrence:** individualCount: 1; sex: male; lifeStage: adult; occurrenceID: 3D76329B-C82A-5E55-A586-6C8EC8556FB3; **Taxon:** scientificName: *Drino auripollinis*; **Location:** country: China; stateProvince: Guizhou; locality: Jiangkou; verbatimElevation: 560 m; **Identification:** identifiedBy: Jianming Zhao; dateIdentified: 1988; **Event:** eventDate: 5.Ⅶ.1988; **Record Level:** collectionCode: Insects
- **Type status:** Other material. **Occurrence:** recordedBy: Chuntian Zhang; individualCount: 1; sex: male; lifeStage: adult; occurrenceID: A4359D3D-C685-5D61-81FB-F19C39451F89; **Taxon:** scientificName: *Drino auripollinis*; **Location:** country: China; stateProvince: Guangxi; locality: Jiuwan Mountains, Huanjiang County; verbatimElevation: 1000–1400 m; verbatimCoordinates: 25°21'N 108°58'E; decimalLatitude: 25.35; decimalLongitude: 108.97; **Identification:** identifiedBy: Chuntian Zhang; dateIdentified: 2015; **Event:** samplingProtocol: sweeping; eventDate: 18–22.Ⅶ.2015; **Record Level:** collectionCode: Insects

#### Diagnosis

Body length (Fig. 16a, b) 11.0 mm. Frons (Fig. 16c, d) 2/3 eye width, parafacial at narrowest point slightly wider than postpedicel (9:8), genal height about 0.15 times eye height. Relative lengths of 2nd, 3rd and 4th costal sectors approximately in ratio of 29:53:37, vein M from bend to crossvein dm-cu 2 times longer than distance from bend to wing hind margin. Mid tibia with 1 *ad*, 2 *p* and 1 *v*. Tergite 4 with a dense pruinose band 2/5 its length, without large sexual hair patches of appressed hairs ventrally, tergite 5 gleaming black. Cerci (Fig. 17a) slender, evenly narrowed, slightly rounded at apex, broadly separated on apical 1/2 in caudal view; in lateral view, cerci nearly straight, narrowed to apex. Surstylus slightly longer than cerci, bluntly rounded at apex in lateral view. Pregonite (Fig. 17b) distinctly longer than distiphallus; postgonite slender; epiphallus short and hook-like at apex, shorter than postgonite; basiphallus and distiphallus distinctly sclerotized; distiphallus weakly expanded in lateral view.

#### Distribution

China (Provinces Chongqing, Fujian, Gansu, Guangxi, Guizhou, Hubei, Hunan, Shanxi, Sichuan, Yunnan).

#### Remarks

We have examined the holotype deposited in IZCAS. *D. longicapilla* Chao & Liang, 1998 is similar to this species but differs in having fronto-orbital plate convex, with dense and long black hairs, parafacial at narrowest point about 1.7 times postpedicel width, tergite 4 with dense pruinose band 2/3 its length.

### Drino densichaeta

Chao & Liang, 1998

7457020E-E8FB-5D3D-9760-52CFBCAEC566

Drino densichaeta Chao & Liang in Chao et al., 1998. – Chao et al. 1998: 1838. – Xue and Wang 2006: 251 (Catalog). – O’Hara et al. 2009: 66 (Catalog). – O’Hara et al. 2020a: 315 (Catalog). – O’Hara et al. 2020: 880 (Catalog). Type material deposited in IZCAS, examined.

#### Materials

- **Type status:** Holotype. **Occurrence:** recordedBy: Xuezhong Zhang; individualCount: 1; sex: male; lifeStage: adult; occurrenceID: B3EFC4E5-A66A-5AEE-9567-1015DB647FCD; **Taxon:** scientificName: *Drino densichaeta*; **Location:** country: China; stateProvince: Yunnan; locality: Lushui City; **Identification:** identifiedBy: Jianming Zhao; dateIdentified: 1981; **Event:** eventDate: 27.Ⅴ.1981; **Record Level:** collectionCode: Insects

#### Diagnosis

Body length (Fig. 18a, b) 12.5 mm. Frons (Fig. 18c, d) 0.86 times eye width, parafacial at narrowest point about 2.5 times postpedicel width, genal height 0.2 times eye height, occiput on upper half mostly with white hairs and a complete row of sparse black setulae behind postocular row, arista thickened at basal 3/10. Two lateral scutellar setae, apical scutellar setae crossed and about 0.7 times scutellum length. Relative lengths of 2nd, 3rd and 4th costal sectors approximately in ratio of 20:20:13, vein M from bend to crossvein dm-cu longer than distance from bend to wing hind margin (25:18). Mid tibia with 1 *ad*, 2 *p* and 1 *v*. Abdomen with thin and dense hairs, tergite 4 with 15 rows of hairs dorsally, tergite 4 with large and tergite 5 with small sexual hair patches of appressed hairs ventrally. Cerci (Fig. 19a) broad, evenly narrowed, slightly pointed at apex, broadly separated on apical 1/4 in caudal view; in lateral view, cerci nearly straight, narrowed in middle and blunt at apex. Surstylus about same length as cerci, bluntly rounded at apex in caudal view, slightly blunt at apex in lateral view. Pregonite (Fig. 19b) apparently wider than postgonite; epiphallus short and hook-like at apex, shorter than postgonite; basiphallus and distiphallus distinctly sclerotized; distiphallus weakly expanded in lateral view.

#### Distribution

China (Province Yunnan).

#### Remarks

We have examined the holotype deposited in IZCAS. *D. oharai* is similar to this species but differs in frons about 0.50 times eye width, occiput mostly with white hairs without a row of black setulae behind postocular row, tergite 4 with sparse hairs, not as dense as in *D. densichaeta*.

### Drino facialis

(Townsend, 1928)

8759A812-4CEB-5E4B-B167-1CB0BC877C56

Sturmiodoria facialis Townsend, 1928. – Townsend 1928: 392. Holotype ♀ in NMNH, examined via photographs.
Sturmia (Sturmia) latistylata Baranov, 1932. – Baranov 1932: 79. – Sabrosky and Crosskey 1969: 50 (des. male lectotype).
Drino facialis : Mesnil 1949a: 10. – Mesnil 1951: 158, 163 (key and description). – Crosskey 1976: 237. – Crosskey 1977: 675 (Catalog). – Chao and Shi 1982: 271 (Catalog). – Chao et al. 1998: 1838 (key and notes). – Liu et al. 1998: 170 (key and descriptions). – Xue and Wang 2006: 251 (Catalog). – O’Hara et al. 2009: 66 (Catalog). – Zhang 2016: 243, 245 (key and descriptions). – Tschorsnig 2017: 151 (Host Catalog). – O’Hara et al. 2020a: 315 (Catalog). – O’Hara et al. 2020: 880 (Catalog).
Drino albifacies Townsend in Mesnil, 1951. – Mesnil 1951: 163 (Manuscript name cited as a synonym, unavailable).

#### Materials

- **Type status:** Holotype. **Occurrence:** individualCount: 1; sex: female; lifeStage: adult; occurrenceID: 1451C0C1-91C0-5714-83A4-1A7F2415AED3; **Taxon:** scientificName: *Drino facialis*; **Location:** locality: Philippines, Island of Basilan Baker, Basilan; **Record Level:** collectionCode: Insects
- **Type status:** Other material. **Occurrence:** recordedBy: Qiang Wang; individualCount: 1; sex: female; lifeStage: adult; occurrenceID: 5C7D366E-BE48-5343-90F2-108B46BD858E; **Taxon:** scientificName: *Drino facialis*; **Location:** country: China; stateProvince: Fujian; locality: Tongmu Village, Nanping City; verbatimElevation: 720–990 m; verbatimCoordinates: 27°30'N 117°40'E; decimalLatitude: 27.5; decimalLongitude: 117.7; **Identification:** identifiedBy: Chuntian Zhang; dateIdentified: 2015; **Event:** samplingProtocol: sweeping; eventDate: 27–30.Ⅳ.2015; **Record Level:** collectionCode: Insects

#### Diagnosis

Body length (Fig. 20a, b) 9.0–11.0 mm. Frons (Fig. 20c) about 0.3 times head width or 0.6 times eye width, genal height less than 1/8 eye height, parafacial at narrowest point narrower than postpedicel, occiput on upper half mostly with white hairs and without a row of black setulae behind postocular row. Apical scutellar setae crossed and about 2/3 scutellum length. Scutellum brown on apical half. Vein M from bend to crossvein dm-cu 1.5 times longer than distance from bend to wing hind margin. Mid tibia with 1 *ad*, 2 *p* and 1 *v*. Abdomen covered with dense pale yellowish pruinosity, tergite 3 with pruinosity band 2/3 its length, tergite 4 with pruinosity band 3/4 its length, with large sexual hair patches of appressed hairs ventrally, tergite 5 gleaming black with pruinosity band 1/2 its length. (Photos of holotype could be found in Global Biodiversity Information Facility (2025d); postabdomen of male as in Fig. 21a and Fig. 21b.)

#### Distribution

China (Provinces and Municipalities Anhui, Beijing, Chongqing, Fujian, Guangdong, Guizhou, Hainan, Hebei, Henan, Hubei, Hunan, Jiangsu, Jiangxi, Liaoning, Inner Mongolia, Ningxia, Shandong, Shanghai, Shanxi, Sichuan, Taiwan, Tianjin, Xizang, Yunnan, Zhejiang).

#### Remarks

We examined the outlook photos of the holotype of *Sturmiodoria facialis* Townsend deposited in the NMNH. *D. parafacialis* Chao & Liang is similar to this species but differs in having parafacial at its narrowest point only slightly wider than postpedicel width, frons of male about 0.8–0.9 times eye width, occiput with sparse black setulae behind postocular row.

### Drino flava

Chao & Liang, 1992

B5D67E8B-660C-5E9A-9959-EA9AAD05E9D7

Drino flava Chao & Liang in Sun et al., 1992. – Sun et al. 1992: 1177. – Chao et al. 1998: 1840 (key and notes). – Liu et al. 1998: 169 (key and descriptions). – O’Hara et al. 2009: 66 (Catalog). – O’Hara et al. 2020a: 315 (Catalog). – O’Hara et al. 2020: 880 (Catalog). Type material deposited in IZCAS, examined.

#### Materials

- **Type status:** Holotype. **Occurrence:** recordedBy: Huaicheng Chai; individualCount: 1; sex: male; lifeStage: adult; occurrenceID: 7280E7D1-1544-5EDB-BCE2-B45456734C92; **Taxon:** scientificName: *Drino flava*; **Location:** country: China; stateProvince: Sichuan; locality: Wolong, Wenchuan County; verbatimElevation: 1600 m; **Identification:** identifiedBy: Jianming Zhao; dateIdentified: 1983; **Event:** eventDate: 26.Ⅶ.1983; **Record Level:** collectionCode: Insects
- **Type status:** Allotype. **Occurrence:** recordedBy: Xuekui Su; individualCount: 1; sex: female; lifeStage: adult; occurrenceID: 44BBA75A-E8B4-52A1-BA02-94A471FA8CB8; **Taxon:** scientificName: *Drino flava*; **Location:** country: China; stateProvince: Hunan; locality: Mt. Tianping, Sangzhi County; verbatimElevation: 1400 m; **Identification:** identifiedBy: Jianming Zhao; dateIdentified: 1988; **Event:** eventDate: 12.Ⅷ.1988; **Record Level:** collectionCode: Insects
- **Type status:** Paratype. **Occurrence:** recordedBy: Huaicheng Chai & Xuezhong Zhang; individualCount: 4; sex: male; lifeStage: adult; occurrenceID: 3E6276B2-1868-5F9E-AD36-0C3543FA0A3A; **Taxon:** scientificName: *Drino flava*; **Location:** country: China; stateProvince: Sichuan; locality: Wolong, Wenchuan; verbatimElevation: 1400–1600 m; **Identification:** identifiedBy: Jianming Zhao; dateIdentified: 1983; **Event:** eventDate: 26.Ⅶ.–3.Ⅷ. 1983; **Record Level:** collectionCode: Insects
- **Type status:** Paratype. **Occurrence:** recordedBy: Huaicheng Chai & Xuezhong Zhang; individualCount: 4; sex: female; lifeStage: adult; occurrenceID: 206DD3FD-702A-51F5-964B-7B6515D50616; **Taxon:** scientificName: *Drino flava*; **Location:** country: China; stateProvince: Sichuan; locality: Wolong, Wenchuan; verbatimElevation: 1400–1600 m; **Identification:** identifiedBy: Jianming Zhao; dateIdentified: 1983; **Event:** eventDate: 26.Ⅶ.–3.Ⅷ. 1983; **Record Level:** collectionCode: Insects
- **Type status:** Paratype. **Occurrence:** recordedBy: Hong Liu; individualCount: 1; sex: male; lifeStage: adult; occurrenceID: F5881BDD-655E-5503-834F-AB3057870E99; **Taxon:** scientificName: *Drino flava*; **Location:** country: China; stateProvince: Hunan; locality: Mt. Tianping, Sangzhi; **Identification:** identifiedBy: Jianming Zhao; dateIdentified: 1988; **Event:** eventDate: 12.Ⅷ.1988; **Record Level:** collectionCode: Insects
- **Type status:** Paratype. **Occurrence:** recordedBy: Hong Liu; individualCount: 8; sex: female; lifeStage: adult; occurrenceID: 25DBB08F-7911-51A4-B6C6-1EB4B1A9DC06; **Taxon:** scientificName: *Drino flava*; **Location:** country: China; stateProvince: Hunan; locality: Mt. Tianping, Sangzhi; verbatimElevation: 1300 m; **Identification:** identifiedBy: Jianming Zhao; dateIdentified: 1988; **Event:** eventDate: 12–15.Ⅷ.1988; **Record Level:** collectionCode: Insects
- **Type status:** Paratype. **Occurrence:** recordedBy: Xuekui Sun; individualCount: 1; sex: female; lifeStage: adult; occurrenceID: 759133BC-12AE-5CB4-83A3-8E5DAF65B99E; **Taxon:** scientificName: *Drino flava*; **Location:** country: China; stateProvince: Hunan; locality: Yongshun; verbatimElevation: 500 m; **Identification:** identifiedBy: Jianming Zhao; dateIdentified: 1988; **Event:** eventDate: 9.Ⅷ.1988; **Record Level:** collectionCode: Insects
- **Type status:** Paratype. **Occurrence:** recordedBy: Xuekui Sun; individualCount: 1; sex: female; lifeStage: adult; occurrenceID: 432DD225-4FBD-5135-9968-796C717A19B6; **Taxon:** scientificName: *Drino flava*; **Location:** country: China; stateProvince: Hunan; locality: Cili; **Identification:** identifiedBy: Jianming Zhao; dateIdentified: 1988; **Event:** eventDate: 3.Ⅸ.1988; **Record Level:** collectionCode: Insects
- **Type status:** Paratype. **Occurrence:** recordedBy: Xuekui Sun; individualCount: 2; sex: male; lifeStage: adult; occurrenceID: BADB7B7F-2723-5CA4-83C7-98FBE56984F5; **Taxon:** scientificName: *Drino flava*; **Location:** country: China; stateProvince: Zhejiang; locality: Tianmu Mountains; **Identification:** identifiedBy: Jianming Zhao; dateIdentified: 1987; **Event:** eventDate: 7.Ⅷ.1987; **Record Level:** collectionCode: Insects
- **Type status:** Paratype. **Occurrence:** recordedBy: Xuekui Sun; individualCount: 1; sex: female; lifeStage: adult; occurrenceID: 418D1309-EF97-518A-B4C2-80488C2FF615; **Taxon:** scientificName: *Drino flava*; **Location:** country: China; stateProvince: Hubei; locality: Hefeng; verbatimElevation: 400 m; **Identification:** identifiedBy: Jianming Zhao; dateIdentified: 1988; **Event:** eventDate: 1.Ⅷ.1988; **Record Level:** collectionCode: Insects
- **Type status:** Paratype. **Occurrence:** recordedBy: Xuekui Sun; individualCount: 1; sex: female; lifeStage: adult; occurrenceID: 971716AF-0982-53F2-9EB2-D38C9B18E390; **Taxon:** scientificName: *Drino flava*; **Location:** country: China; stateProvince: Guizhou; locality: Jiangkou; verbatimElevation: 500 m; **Identification:** identifiedBy: Jianming Zhao; dateIdentified: 1988; **Event:** eventDate: 15.Ⅶ.1988; **Record Level:** collectionCode: Insects
- **Type status:** Paratype. **Occurrence:** recordedBy: Xuezhong Zhang; individualCount: 1; sex: female; lifeStage: adult; occurrenceID: 09A3C947-FC7D-5512-A986-0F5A6659816F; **Taxon:** scientificName: *Drino flava*; **Location:** country: China; stateProvince: Yunnan; locality: Longzhiben Mountains; verbatimElevation: 2500 m; **Identification:** identifiedBy: Jianming Zhao; dateIdentified: 1981; **Event:** eventDate: 21.Ⅶ.1981; **Record Level:** collectionCode: Insects
- **Type status:** Paratype. **Occurrence:** recordedBy: Xuezhong Zhang; individualCount: 1; sex: male; lifeStage: adult; occurrenceID: FE22A311-846C-5C08-8DFF-2D5C88D4C5FF; **Taxon:** scientificName: *Drino flava*; **Location:** country: China; stateProvince: Yunnan; locality: Lushui; verbatimElevation: 2100 m; **Identification:** identifiedBy: Jianming Zhao; dateIdentified: 1981; **Event:** eventDate: 22.Ⅴ.1981; **Record Level:** collectionCode: Insects
- **Type status:** Other material. **Occurrence:** recordedBy: Yue Li; individualCount: 1; sex: female; lifeStage: adult; occurrenceID: 0B7FBC5E-2BEE-566A-A529-8B8A9AA1DEDE; **Taxon:** scientificName: *Drino flava*; **Location:** country: China; stateProvince: Chongqing; locality: Guimenguan, Yintiaoling, Wuxi City; verbatimElevation: 1460 m; verbatimCoordinates: 31°48'N 109°91'E; decimalLatitude: 31.8; decimalLongitude: 109.9; **Identification:** identifiedBy: Chuntian Zhang; dateIdentified: 2022; **Event:** samplingProtocol: sweeping; eventDate: 16–18.Ⅷ.2022; **Record Level:** collectionCode: Insects
- **Type status:** Other material. **Occurrence:** recordedBy: Chuntian Zhang; individualCount: 1; sex: female; lifeStage: adult; occurrenceID: E6333D37-70C7-5D17-9BE8-C9C8B9005AA1; **Taxon:** scientificName: *Drino flava*; **Location:** country: China; stateProvince: Fujian; locality: Tongmu Village, Wuyi Mountains; verbatimElevation: 755 m; verbatimCoordinates: 27°74'N 117°64'E; decimalLatitude: 28.23; decimalLongitude: 118.06; **Identification:** identifiedBy: Chuntian Zhang; dateIdentified: 2021; **Event:** samplingProtocol: sweeping; eventDate: 28.Ⅴ.2021; **Record Level:** collectionCode: Insects
- **Type status:** Other material. **Occurrence:** recordedBy: Chuntian Zhang & Xinyi Li; individualCount: 1; sex: female; lifeStage: adult; occurrenceID: 51D9551C-3613-548F-A8EA-B44119F1234D; **Taxon:** scientificName: *Drino flava*; **Location:** country: China; stateProvince: Fujian; locality: Tongmu Village, Wuyi Mountains; verbatimElevation: 650–802m; verbatimCoordinates: 27°43'N 117°41'E; decimalLatitude: 27.71; decimalLongitude: 117.68; **Identification:** identifiedBy: Chuntian Zhang; dateIdentified: 2022; **Event:** samplingProtocol: sweeping; eventDate: 10.Ⅶ.2022; **Record Level:** collectionCode: Insects
- **Type status:** Other material. **Occurrence:** recordedBy: Ruiqing Dong; individualCount: 2; sex: female; lifeStage: adult; occurrenceID: 0D1638A6-3221-5715-AE94-EB05D3D005FA; **Taxon:** scientificName: *Drino flava*; **Location:** country: China; stateProvince: Gansu; locality: Qinghe Forest Farm, Kang County, Longnan; verbatimElevation: 1529.1m; verbatimCoordinates: 33.2068°N 105.8750°E; decimalLatitude: 33.2068; decimalLongitude: 105.875; **Identification:** identifiedBy: Chuntian Zhang; dateIdentified: 2023; **Event:** samplingProtocol: sweeping; eventDate: 4.Ⅶ.2023; **Record Level:** collectionCode: Insects
- **Type status:** Other material. **Occurrence:** recordedBy: Chuntian Zhang; individualCount: 1; sex: female; lifeStage: adult; occurrenceID: EA6B608D-1C6C-506F-9C2B-7BC5C0FE57DE; **Taxon:** scientificName: *Drino flava*; **Location:** country: China; stateProvince: Liaoning; locality: Hunheyuan Nature Reserve, Huangdaigou, Qingyuan City; verbatimElevation: 500–800m; **Identification:** identifiedBy: Chuntian Zhang; dateIdentified: 2016; **Event:** samplingProtocol: sweeping; eventDate: 12–16.Ⅶ.2016; **Record Level:** collectionCode: Insects
- **Type status:** Other material. **Occurrence:** recordedBy: Chuntian Zhang & Xinyi Li; individualCount: 1; sex: female; lifeStage: adult; occurrenceID: 98E50693-930B-5939-9315-7BA539F6139F; **Taxon:** scientificName: *Drino flava*; **Location:** country: China; stateProvince: Liaoning; locality: Huangdaigou, Qingyuan; verbatimElevation: 600–800m; **Identification:** identifiedBy: Chuntian Zhang; dateIdentified: 2016; **Event:** samplingProtocol: sweeping; eventDate: 5.Ⅷ.2016; **Record Level:** collectionCode: Insects
- **Type status:** Other material. **Occurrence:** recordedBy: Bo Hao & Shidie Liu; individualCount: 1; sex: female; lifeStage: adult; occurrenceID: 8ECEB9BD-A26B-5FF5-AC06-EEF8CB493522; **Taxon:** scientificName: *Drino flava*; **Location:** country: China; stateProvince: Liaoning; locality: Heshangmaozi, Tanggou, Benxi City ; verbatimElevation: 588–750m; verbatimCoordinates: 41°70'N, 124°13'E; decimalLatitude: 42.17; decimalLongitude: 124.22; **Identification:** identifiedBy: Chuntian Zhang; dateIdentified: 2020; **Event:** samplingProtocol: sweeping; eventDate: 24–26.Ⅶ.2020; **Record Level:** collectionCode: Insects
- **Type status:** Other material. **Occurrence:** recordedBy: Yuzhuo Wang; individualCount: 1; sex: female; lifeStage: adult; occurrenceID: EC27391C-F7E6-595E-A9FF-12C1D72B1A75; **Taxon:** scientificName: *Drino flava*; **Location:** country: China; stateProvince: Liaoning; locality: Heshangmaozi, Tanggou, Benxi City ; verbatimElevation: 588–750 m; verbatimCoordinates: 41°70'N, 124°13'E; decimalLatitude: 42.17; decimalLongitude: 124.22; **Identification:** identifiedBy: Chuntian Zhang; dateIdentified: 2020; **Event:** samplingProtocol: sweeping; eventDate: 24–26.Ⅶ.2020; **Record Level:** collectionCode: Insects
- **Type status:** Other material. **Occurrence:** recordedBy: Qiang Wang; individualCount: 2; sex: female; lifeStage: adult; occurrenceID: DB290626-19A5-56E4-B9FD-6219E5DFEE9C; **Taxon:** scientificName: *Drino flava*; **Location:** country: China; stateProvince: Sichuan; locality: Qingcheng mountains, Chengdu City; verbatimElevation: 900–1100 m; verbatimCoordinates: 30°55'N, 103°29'E; decimalLatitude: 30.92; decimalLongitude: 103.48; **Identification:** identifiedBy: Chuntian Zhang; dateIdentified: 2015; **Event:** samplingProtocol: sweeping; eventDate: 27–28.Ⅶ.2015; **Record Level:** collectionCode: Insects

#### Diagnosis

Body length (Fig. 22a, b) 9.0–11.0 mm. Frons (Fig. 22c, d) 1.3 times longer than facial length, parafacial at narrowest point about 1.3 times postpedicel width, genal height about 0.1 times eye height, antenna with postpedicel about 2.8 times as long as pedicel, arista thickened at basal 2/5. Relative lengths of 2nd, 3rd and 4th costal sectors approximately in ratio of 11:20:12, vein M from bend to crossvein dm-cu longer than distance from bend to wing hind margin (42:23). Mid tibia with 1 *ad*, 2 *p* and 1 *v*. Abdomen yellow, tergite 3 without black band at posterior margin, tergites 3 and 4 with 1/4–1/3 square or trapezoidal dorsal black spot at middle, tergite 4 on apical 1/4 and tergite 5 gleaming black, abdomen with pale gray pruinosity. Cerci (Fig. 23a) slender, evenly narrowed, slightly pointed at apex, broadly separated on apical 2/5 in caudal view, in lateral view cerci nearly straight, narrowed to apex. Surstylus about same length as cerci, bluntly rounded at apex in caudal view, slightly blunt in lateral view. Pregonite (Fig. 23b) slender; postgonite about as wide as pregonite; epiphallus short and hook-like at apex, shorter than postgonite; basiphallus and distiphallus distinctly sclerotized; distiphallus weakly expanded in lateral view.

#### Distribution

China (Provinces Guizhou, Hubei, Hunan, Shanxi, Sichuan, Yunnan, Zheijang).

#### Remarks

We have examined the holotype deposited in IZCAS. *D. facialis* (Townsend) is similar to this species but differs in abdomen mainly black, tergites 2–4 with more or less reddish yellow on both sides but always obscure, tergites 3, 4 with black band on apical margin, parafacial at its narrowest point narrower than antenna width.

### Drino interfrons

(Sun & Chao, 1992)

C23E17C5-6CC0-5C32-9D43-2468876CD760

Thecocarcelia interfrons Sun & Chao in Sun et al., 1992. – Sun et al. 1992: 1189. – Chao et al. 1998: 1821 (key and notes). Type material deposited in IZCAS, examined.
Drino interfrons : O’Hara et al. 2009: 66 (Catalog). – O’Hara et al. 2020a: 316 (Catalog). – O’Hara et al. 2020: 880 (Catalog).

#### Materials

- **Type status:** Holotype. **Occurrence:** recordedBy: Xuekui Sun; individualCount: 1; sex: male; lifeStage: adult; occurrenceID: 84F0421B-CA63-5076-B171-A65B4F5D7152; **Taxon:** scientificName: *Drino interfrons*; **Location:** country: China; stateProvince: Hunan; locality: Zhushitou, Dayong City; verbatimElevation: 450 m; **Identification:** identifiedBy: Jianming Zhao; dateIdentified: 1988; **Event:** eventDate: 21.Ⅷ.1988; **Record Level:** collectionCode: Insects
- **Type status:** Paratype. **Occurrence:** recordedBy: Xuekui Sun; individualCount: 1; sex: female; lifeStage: adult; occurrenceID: 9608E9D5-AA8E-5C11-BFA3-F8094D8C303F; **Taxon:** scientificName: *Drino interfrons*; **Location:** country: China; stateProvince: Hunan; locality: Zhushitou, Dayong City; verbatimElevation: 450 m; **Identification:** identifiedBy: Jianming Zhao; dateIdentified: 1988; **Event:** eventDate: 21.Ⅷ.1988; **Record Level:** collectionCode: Insects
- **Type status:** Paratype. **Occurrence:** recordedBy: Xuekui Sun; individualCount: 1; sex: female; lifeStage: adult; occurrenceID: A6AEB643-065E-5952-AF98-A9FAB11452D9; **Taxon:** scientificName: *Drino interfrons*; **Location:** country: China; stateProvince: Guizhou; locality: Jiangkou, Guizhou; verbatimElevation: 560 m; **Identification:** identifiedBy: Jianming Zhao; dateIdentified: 1988; **Event:** eventDate: 5.Ⅶ.1988; **Record Level:** collectionCode: Insects
- **Type status:** Paratype. **Occurrence:** recordedBy: Xuekui Sun; individualCount: 1; sex: female; lifeStage: adult; occurrenceID: DB352529-5F9C-5049-8027-A2D5DA264CC8; **Taxon:** scientificName: *Drino interfrons*; **Location:** country: China; stateProvince: Guizhou; locality: Mt. Fanjing, Jiangkou; verbatimElevation: 560 m; **Identification:** identifiedBy: Jianming Zhao; dateIdentified: 1988; **Event:** eventDate: 15.Ⅶ.1988; **Record Level:** collectionCode: Insects
- **Type status:** Paratype. **Occurrence:** recordedBy: Xuekui Sun; individualCount: 1; sex: female; lifeStage: adult; occurrenceID: A18D3E30-06BA-58C6-A7EF-D099F4B95904; **Taxon:** scientificName: *Drino interfrons*; **Location:** country: China; stateProvince: Hunan; locality: Shanmuhe Forest Farm, Yongshun County; verbatimElevation: 500 m; **Identification:** identifiedBy: Jianming Zhao; dateIdentified: 1988; **Event:** eventDate: 9.Ⅷ.1988; **Record Level:** collectionCode: Insects
- **Type status:** Paratype. **Occurrence:** recordedBy: Xuekui Sun; individualCount: 1; sex: female; lifeStage: adult; occurrenceID: F3D7C41F-771F-55FD-A738-828C5225151C; **Taxon:** scientificName: *Drino interfrons*; **Location:** country: China; stateProvince: Hunan; locality: Gaowangjie, Guzhang County; verbatimElevation: 550 m; **Identification:** identifiedBy: Jianming Zhao; dateIdentified: 1988; **Event:** eventDate: 4.Ⅶ.1988; **Record Level:** collectionCode: Insects
- **Type status:** Other material. **Occurrence:** recordedBy: Shidie Liu; individualCount: 1; sex: male; lifeStage: adult; occurrenceID: BE093289-7133-56D6-A486-B332007D3E26; **Taxon:** scientificName: *Drino interfrons*; **Location:** country: China; stateProvince: Guangdong; locality: Lianping, Huangjiang County; verbatimElevation: 543.3 m; verbatimCoordinates: 24°27'N, 114°25'E; decimalLatitude: 24.45; decimalLongitude: 114.41; **Identification:** identifiedBy: Chuntian Zhang; dateIdentified: 2021; **Event:** samplingProtocol: sweeping; eventDate: 5.Ⅵ.2021; **Record Level:** collectionCode: Insects

#### Diagnosis

Body length (Fig. 24a, b) 9.0 mm. Frons (Fig. 24c, d) of male about 1/3 eye width, frontal vitta 1.4 times wider than fronto-orbital plate at middle, parafacial at narrowest point about 0.7 times postpedicel width, genal height 0.025 times eye height, 4 reclinate orbital setae, occiput on upper half mostly with white hairs and without black setula behind postocular row. Antenna with postpedicel about 2.6 times as long as pedicel, arista thickened at basal 2/5, palpus dark brown. Relative lengths of 2nd, 3rd and 4th costal sectors approximately in ratio of 34:50:25, vein M from bend to crossvein dm-cu longer than distance between bend and wing hind margin (40:23). Mid tibia with 1 *ad*. Tergite 4 without large sexual hair patches of appressed hairs ventrally. Cerci (Fig. 25a) wide at base, sharply narrowed, slightly pointed at apex, narrowly separated on apical 1/2 in caudal view, in lateral view cerci nearly straight, narrowed to apex. Surstylus slightly shorter than cerci, bluntly round at apex in caudal view, slightly blunt in lateral view. Pregonite (Fig. 25b) longer than distiphallus; postgonite slender; basiphallus distinctly sclerotized; distiphallus short and weakly expanded in lateral view.

#### Distribution

China (Provinces Fujian, Guangdong, Guizhou, Hunan).

#### Remarks

We have examined the holotype deposited in IZCAS. *D. longicapilla* Chao & Liang is similar to this species but differs in frons of male about 0.5 times eye width, parafacial at narrowest point about 1.7 times postpedicel width, genal height 0.11 times eye height.

### Drino laticornis

Chao & Liang, 1998

73E9A303-7264-54EF-9641-6B0EE98F47FB

Drino laticornis Chao & Liang in Chao et al., 1998. – Chao et al. 1998: 1845. – O’Hara et al. 2009: 67 (Catalog). – O’Hara et al. 2020a: 316 (Catalog). – O’Hara et al. 2020: 880 (Catalog). Type material deposited in IZCAS, examined.

#### Materials

- **Type status:** Holotype. **Occurrence:** recordedBy: Enyi Liang; individualCount: 1; sex: male; lifeStage: adult; occurrenceID: FBF9F78B-7786-5A15-9741-EBD447355FDF; **Taxon:** scientificName: *Drino laticornis*; **Location:** country: China; stateProvince: Beijing; locality: Qinglong Bridge; **Identification:** identifiedBy: Jianming Zhao; dateIdentified: 1980; **Event:** eventDate: 6.Ⅶ.1980; **Record Level:** collectionCode: Insects

#### Diagnosis

Body length (Fig. 26a, b) 7.5 mm. Frons (Fig. 26c, d) of male about 1.2 times eye width, parafacial at narrowest point about 0.9 times postpedicel width, 5–7 reclinate orbital setae, genal height about 0.1 times eye height, occiput on upper half mostly with white hairs and a complete row of black setulae behind postocular row. Relative lengths of 2nd, 3rd and 4th costal sectors approximately in ratio of 4:6:3, vein M from bend to crossvein dm-cu shorter than distance between bend and wing hind margin (3:4). Tergites 4 and 5 with large hair patches of appressed hairs ventrally. Cerci (Fig. 27a) slender, evenly narrowed, slightly pointed at apex, narrowly separated on apical 1/2 in caudal view, in lateral view cerci nearly straight, narrowed to apex. Surstylus shorter than cerci, bluntly round at apex in caudal view, slightly blunt in lateral view. Pregonite (Fig. 27b) slightly curved and long; postgonite slender and distinctly shorter than pregonite; epiphallus short and hook-like at apex, about as long as postgonite; basiphallus and distiphallus distinctly sclerotized; distiphallus weakly expanded in lateral view.

#### Distribution

China (Beijing (Municipality), Province Hebei).

#### Remarks

We have examined the holotype deposited in IZCAS. *D. interfrons* (Sun & Chao) is similar to this species but differs in frons of male about 1/3 eye width, parafacial at narrowest point about 0.7 times postpedicel width, genal height 0.025 times eye height.

### Drino latifrons

Liu, Zhang & Xi, 2023

B3CF5FCD-2A04-5A66-838F-CB6464ADEC88

Drino latifrons Liu, Zhang & Xi, 2023. – Liu et al. 2023: 185. Type material deposited in SYNU, examined.

#### Materials

- **Type status:** Holotype. **Occurrence:** recordedBy: Zixuan Huang; individualCount: 1; sex: male; lifeStage: adult; occurrenceID: FC880639-C2E2-50DC-9A88-E1BDABC093B0; **Taxon:** scientificName: *Drino latifrons*; **Location:** country: China; stateProvince: Sichuan; locality: No. 2 experimental field, Waqie, Hongyuan, Aba; verbatimCoordinates: 32°48'N, 102°33'E; decimalLatitude: 32.8; decimalLongitude: 102.55; **Identification:** identifiedBy: Chuntian Zhang; dateIdentified: 2021; **Event:** eventDate: 5.Ⅶ.2021; **Record Level:** collectionCode: Insects
- **Type status:** Paratype. **Occurrence:** recordedBy: Zixuan Huang; individualCount: 11; sex: male; lifeStage: adult; occurrenceID: 8F84E21B-0ABB-5C55-B840-3F0CDED3CAAE; **Taxon:** scientificName: *Drino latifrons*; **Location:** country: China; stateProvince: Sichuan; locality: No. 2 experimental field, Waqie, Hongyuan, Aba; verbatimCoordinates: 32°48'N, 102°33'E; decimalLatitude: 32.8; decimalLongitude: 102.55; **Identification:** identifiedBy: Chuntian Zhang; dateIdentified: 2021; **Event:** eventDate: 5.Ⅶ.2021; **Record Level:** collectionCode: Insects
- **Type status:** Other material. **Occurrence:** recordedBy: Chuntian Zhang & Xinyi Li ; individualCount: 11; sex: male; lifeStage: adult; occurrenceID: CB272642-A9DF-5FD0-BC2B-00AB5D9E5363; **Taxon:** scientificName: *Drino latifrons*; **Location:** country: China; stateProvince: Qinghai; locality: Maixiu Forest Farm, Zeku County; verbatimElevation: 2850–2950 m; verbatimCoordinates: 35°16'N, 101°55'E; decimalLatitude: 35.27; decimalLongitude: 101.92; **Identification:** identifiedBy: Chuntian Zhang; dateIdentified: 2020; **Event:** samplingProtocol: sweeping; eventDate: 21–22.Ⅶ.2020; **Record Level:** collectionCode: Insects

#### Diagnosis

Body length (Fig. 28a, b) 7.2–9.6 mm. Eye (Fig. 28c, d) bare. Frons about 0.4 times head width or 1.3–1.4 times eye width, fronto-orbital plate with a row of 5–7 reclinate orbital setae and short black hairs on outer surface, arista thickened on basal 2/3 to 3/4. Scutellum reddish yellow except for base black, 3 presutural and 4 postsutural dorsocentral setae. Mid tibia with 3–4 *ad*, 3 *p* and 2 *v*. Abdominal tergites 4 and 5 of male each with large sexual hair patches of appressed hairs ventrally. Cerci (Fig. 29a) slender, evenly narrowed, slightly pointed at apex, narrowly separated on apical 1/3 in caudal view, in lateral view cerci nearly straight, narrowed at apex. Surstylus about same length as cerci, bluntly round at apex in caudal view, widely blunt in lateral view. Pregonite (Fig. 29b) with several setae on dorsal surface; postgonite slender and slightly hook-like; epiphallus straight at base and hook-like at apex, longer than postgonite; basiphallus and distiphallus distinctly sclerotized; distiphallus weakly expanded in lateral view; acrophallus sharp at apex and outer rim not exceed by posterior part of distiphallus.

#### Distribution

China (Provinces Qinghai, Sichuan).

#### Remarks

We have examined the holotype deposited in IZCAS. *D. adiscalis* (Chao & Shi) is similar to this species but differs in fronto-orbital plate with a row of 3–4 reclinate orbital setae, Mid tibia with 4–5 *ad*, 2 *p* and 2 *v*, surstyli about same length as cerci in lateral view.

### Drino longicapilla

Chao & Liang, 1998

3A08D622-BC12-517E-B79B-CE75FFD59D6D

Drino longicapilla Liang & Chao in Chao et al., 1998. – Chao et al. 1998: 1847. – O’Hara et al. 2009: 67 (Catalog). – O’Hara et al. 2020a: 316 (Catalog). – O’Hara et al. 2020: 880 (Catalog). Type material deposited in IZCAS, examined.

#### Materials

- **Type status:** Holotype. **Occurrence:** recordedBy: Xuezhong Zhang ; individualCount: 1; sex: male; lifeStage: adult; occurrenceID: 5B69D3B8-E130-5DC5-890E-C017383EAE78; **Taxon:** scientificName: *Drino longicapilla*; **Location:** country: China; stateProvince: Yunnan; locality: Yunlong; verbatimElevation: 2500m; **Identification:** identifiedBy: Jianming Zhao; dateIdentified: 1981; **Event:** eventDate: 22.Ⅵ.1981; **Record Level:** collectionCode: Insects

#### Diagnosis

Body length (Fig. 30a, b) 11.5 mm. Frons (Fig. 30c, d) 0.5 times eye width, fronto-orbital plate convex, parafacial at narrowest point about 1.7 times postpedicel width, occiput on upper half mostly with white hairs and 1–2 rows of black setulae behind postocular row. Relative lengths of 2nd, 3rd and 4th costal sectors approximately in ratio of 73:100:60, vein M from bend to crossvein dm-cu longer than distance between bend and wing hind margin (48:25). Mid tibia with 1 strong *ad*. Tergite 4 on apical 2/3 and tergite 5 gleaming black, tergite 4 without large sexual hair patches of appressed hairs ventrally. Cerci (Fig. 31a) slender, evenly narrowed, slightly pointed at apex, narrowly separated on apical 1/3 in caudal view, in lateral view cerci nearly straight, blunt at apex. Surstylus about slightly shorter than cerci, sharp on apical half in caudal view, slightly blunt in lateral view. Pregonite (Fig. 31b) about as long as distiphallus; epiphallus short and hook-like at apex; basiphallus and distiphallus distinctly sclerotized; distiphallus weakly expanded in lateral view.

#### Distribution

China (Provinces Liaoning, Neimenggu, Yunnan).

#### Remarks

We have examined the holotype deposited in IZCAS. D. *auripollinis* Chao & Liang is similar to this species but differs in having fronto-orbital plate flat, with sparse and short black hairs, parafacial at narrowest point slightly wider than antenna (27:24), tergite 4 with dense pruinosity band 2/5 of its length.

### Drino longihirta

Chao & Liang, 1992

631250DD-7264-5342-A1C1-1DBF75B06182

Drino longihirta Chao & Liang in Sun et al., 1992. – Sun et al. 1992: 1180. – Chao et al. 1998: 1849 (key and note). – O’Hara et al. 2009: 67 (Catalog). – Zhang 2016: 243, 245 (key and descriptions). – O’Hara et al. 2020a: 316 (Catalog). – O’Hara et al. 2020: 880 (Catalog). Type material deposited in IZCAS, examined.

#### Materials

- **Type status:** Holotype. **Occurrence:** recordedBy: Xuekui Sun; individualCount: 1; sex: male; lifeStage: adult; occurrenceID: B2D63AAA-19BB-586A-AE0C-50414D47C1AC; **Taxon:** scientificName: *Drino longihirta*; **Location:** country: China; stateProvince: Hunan; locality: Mt. Tianping, Sangzhi County; verbatimElevation: 1200m; **Identification:** identifiedBy: Jianming Zhao; dateIdentified: 1988; **Event:** eventDate: 15.Ⅷ.1988; **Record Level:** collectionCode: Insects
- **Type status:** Paratype. **Occurrence:** recordedBy: Xuezhong Zhang ; individualCount: 1; sex: male; lifeStage: adult; occurrenceID: 82C1DFD3-3857-56B0-A7F2-31489C57C603; **Taxon:** scientificName: *Drino longihirta*; **Location:** country: China; stateProvince: Yunan; locality: Zhiben Mountains, Yunlong County; verbatimElevation: 2500m; **Identification:** identifiedBy: Jianming Zhao; dateIdentified: 1981; **Event:** eventDate: 24.Ⅶ.1981; **Record Level:** collectionCode: Insects
- **Type status:** Other material. **Occurrence:** recordedBy: Qiang Wang; individualCount: 1; sex: male; lifeStage: adult; occurrenceID: 3F567823-5BC3-5BC0-BA84-C9F227F48ECC; **Taxon:** scientificName: *Drino longihirta*; **Location:** country: China; stateProvince: Anhui; locality: Huang mountains, Huangjiang County; verbatimElevation: 100–550m; verbatimCoordinates: 30°20'N, 117°30'E; decimalLatitude: 30.3; decimalLongitude: 117.5; **Identification:** identifiedBy: Chuntian Zhang; dateIdentified: 2015; **Event:** samplingProtocol: sweeping; eventDate: 7–16.Ⅴ.2015; **Record Level:** collectionCode: Insects

#### Diagnosis

Body length (Fig. 32a, b) 11.0 mm. Eye (Fig. 32c, d) with ommatrichia about 4 facets in length. Frons of male about 0.7 times eye width, parafacial at narrowest point about 1.5 times postpedicel width and bare below lowest frontal seta, genal height about 0.13 times eye height, antenna with postpedicel about 3 times as long as pedicel, occiput on upper half mostly with white hairs and without black setulae behind postocular row, palpus dark brown and gradually darkened at base. Scutellum reddish yellow on apical 7/10. Relative lengths of 2nd, 3rd and 4th costal sectors approximately in ratio of 60:100:51, vein M curved at bending, vein M from bend to crossvein dm-cu longer than distance between bend and wing hind margin (20:13). Mid tibia with 2 *ad*. Tergites 4 and 5 with sexual hair patches of appressed hairs ventrally. Cerci (Fig. 33a) slender, evenly narrowed, slightly pointed at apex, narrowly separated on apical 3/7 in caudal view, cerci nearly straight, narrowed to apex in lateral view. Surstylus about same length as cerci, bluntly rounded at apex in caudal view, slightly pointed in lateral view. Pregonite (Fig. 33b) straight and wide; epiphallus short and hook-like at apex; basiphallus and distiphallus distinctly sclerotized; distiphallus weakly expanded in lateral view.

#### Distribution

China (Provinces and Municipalities Anhui, Beijing, Heilongjiang, Hunan, Jilin, Liaoning, Shanxi, Sichuan, Yunnan).

#### Remarks

We have examined the holotype deposited in IZCAS. *D. lota* Chao & Liang is similar to this species but differs in having frons of male about 0.6 times eye width, antenna with postpedicel about 2 times as long as pedicel, mid tibia with 1 *ad*, surstylus bluntly rounded at apex, slightly longer than cerci in lateral view.

### Drino lota

(Meigen, 1824)

1242788D-3F60-553B-BC62-ED17DD9815B6

Tachina lota Meigen, 1824. – Meigen 1824: 326. – Herting 1972: 9 (des. male lectotype).
Tachina rapida Meigen, 1824. – Meigen 1824: 326. – Mesnil 1951: 164.
Exorista lota Meigen, 1838. – Meigen 1838: 255. Syntypes (♂ & ♀) in MNHN, external morphology examined via photographs.
Exorista rapida Meigen, 1838. – Meigen 1838: 255.
Exorista fugax Rondani, 1859. – Rondani 1859: 127, It 9 Parma (Italy), des. Herting 1969: 194.
Drino volucris Robineau-Desvoidy, 1863. – Robineau-Desvoidy 1863: 250. – Mesnil 1951: 164.
Exorista immunita Pandellé, 1896. – Pandellé 1896: 28. – Mesnil 1951: 164.
Sturmia quadrimaculata Girschner, 1899. – Girschner 1899: 182, d Thurin–gen (DDR).
Drino lota : Mesnil 1949a: 10 (revision). – Mesnil 1951: 158, 164 (key and descriptions). – Herting 1984: 53 (Catalog). – Herting and Dely-Draskovits 1993: 208 (Catalog). – Tschorsnig and Herting 1994: 54, 136 (key and distribution). – Chao et al. 1998: 1850 (key and notes). – Xue and Wang 2006: 252 (Catalog). – O’Hara et al. 2009: 67 (Catalog). – Tschorsnig 2017: 158 (Host Catalog). – O’Hara et al. 2020a: 316 (Catalog). – O’Hara et al. 2020: 880 (Catalog).

#### Materials

- **Type status:** Holotype. **Occurrence:** individualCount: 1; sex: female; lifeStage: adult; occurrenceID: F642A244-07C8-5D9F-817C-0CD26F129826; **Taxon:** scientificName: *Exorista rapida*; **Identification:** identifiedBy: Meigen; dateIdentified: 1838; **Record Level:** collectionCode: Insects
- **Type status:** Syntype. **Occurrence:** individualCount: 1; sex: male; lifeStage: adult; occurrenceID: B57A8EFA-F4EA-5DC7-81BD-B51B1EFE82E5; **Taxon:** scientificName: *Exorista lota*; **Identification:** identifiedBy: Meigen; dateIdentified: 1838; **Record Level:** collectionCode: Insects
- **Type status:** Other material. **Occurrence:** recordedBy: Min Wang; individualCount: 1; sex: male; lifeStage: adult; occurrenceID: 3B06A8A1-778B-5E36-9664-70355E3EC47C; **Taxon:** scientificName: *Drino lota*; **Location:** country: China; stateProvince: Guangdong; locality: Yuenanling Nature Reserve; **Identification:** identifiedBy: Chuntian Zhang; dateIdentified: 2011; **Event:** samplingProtocol: sweeping; eventDate: 18–21.Ⅴ.2011; **Record Level:** collectionCode: Insects
- **Type status:** Other material. **Occurrence:** recordedBy: Chuntian Zhang; individualCount: 1; sex: male; lifeStage: adult; occurrenceID: EB8D1931-95AC-5E90-B465-05777C138497; **Taxon:** scientificName: *Drino lota*; **Location:** country: China; stateProvince: Guagnxi; locality: Mt. Maoer, Guilin City; verbatimElevation: 350–1180–2100 m; **Identification:** identifiedBy: Chuntian Zhang; dateIdentified: 2004; **Event:** samplingProtocol: sweeping; eventDate: 4.Ⅴ–29.Ⅳ.2004; **Record Level:** collectionCode: Insects
- **Type status:** Other material. **Occurrence:** recordedBy: Chuntian Zhang; individualCount: 1; sex: male; lifeStage: adult; occurrenceID: 322982D4-4A4E-5818-9955-D3F52BA68823; **Taxon:** scientificName: *Drino lota*; **Location:** country: China; stateProvince: Liaoning; locality: Mt. Tiecha, Benxi City; verbatimElevation: 500–950 m; **Identification:** identifiedBy: Chuntian Zhang; dateIdentified: 2006; **Event:** samplingProtocol: sweeping; eventDate: 28.Ⅴ.2006; **Record Level:** collectionCode: Insects
- **Type status:** Other material. **Occurrence:** recordedBy: Jing Hao; individualCount: 1; sex: male; lifeStage: adult; occurrenceID: C7993D56-2E96-5704-B7AD-4A0EB226CCC2; **Taxon:** scientificName: *Drino lota*; **Location:** country: China; stateProvince: Liaoning; locality: Sunligou, Tianshifu, Benxi City; verbatimElevation: 380–580 m; **Identification:** identifiedBy: Chuntian Zhang; dateIdentified: 2006; **Event:** samplingProtocol: sweeping; eventDate: 29.Ⅴ.2006; **Record Level:** collectionCode: Insects
- **Type status:** Other material. **Occurrence:** recordedBy: Chuntian Zhang; individualCount: 1; sex: female; lifeStage: adult; occurrenceID: C7AB3EC4-D0B2-5F21-BAB6-E40A12979304; **Taxon:** scientificName: *Drino lota*; **Location:** country: China; stateProvince: Liaoning; locality: Laotuding, Huanren County, Benxi City; verbatimElevation: 500–650 m; **Identification:** identifiedBy: Chuntian Zhang; dateIdentified: 2006; **Event:** samplingProtocol: sweeping; eventDate: 30.Ⅴ.2006; **Record Level:** collectionCode: Insects
- **Type status:** Other material. **Occurrence:** recordedBy: Jing Lian & Jing Hao; individualCount: 1; sex: female; lifeStage: adult; occurrenceID: F0187C19-8993-59C3-8CAD-1BCFF4D0E57E; **Taxon:** scientificName: *Drino lota*; **Location:** country: China; stateProvince: Liaoning; locality: Laotuding, Huanren County, Benxi City; verbatimElevation: 500–650 m; **Identification:** identifiedBy: Chuntian Zhang; dateIdentified: 2006; **Event:** samplingProtocol: sweeping; eventDate: 10.Ⅶ.2006; **Record Level:** collectionCode: Insects
- **Type status:** Other material. **Occurrence:** recordedBy: Hai Lin; individualCount: 2; sex: male; lifeStage: adult; occurrenceID: 24CBBD13-6333-5A0B-BE2A-4822D2B5676C; **Taxon:** scientificName: *Drino lota*; **Location:** country: China; stateProvince: Liaoning; locality: Qingshangou, Kuandian County; **Identification:** identifiedBy: Chuntian Zhang; dateIdentified: 2011; **Event:** samplingProtocol: sweeping; eventDate: 17.Ⅶ.2011; **Record Level:** collectionCode: Insects
- **Type status:** Other material. **Occurrence:** recordedBy: Qiang Wang; individualCount: 12; sex: male; lifeStage: adult; occurrenceID: A754094A-CE61-5115-AB43-DC35A3CF6094; **Taxon:** scientificName: *Drino lota*; **Location:** country: China; stateProvince: Liaoning; locality: Laotuding, Huanren County, Benxi City; verbatimElevation: 550–1300 m; **Identification:** identifiedBy: Chuntian Zhang; dateIdentified: 2011; **Event:** samplingProtocol: sweeping; eventDate: 1.Ⅶ.–30.Ⅵ.2011; **Record Level:** collectionCode: Insects
- **Type status:** Other material. **Occurrence:** recordedBy: Qiang Wang; individualCount: 1; sex: female; lifeStage: adult; occurrenceID: C763C58C-1C47-576E-A247-ACBB5E773B02; **Taxon:** scientificName: *Drino lota*; **Location:** country: China; stateProvince: Liaoning; locality: Laotuding, Huanren County, Benxi City; verbatimElevation: 550–1300 m; **Identification:** identifiedBy: Chuntian Zhang; dateIdentified: 2011; **Event:** samplingProtocol: sweeping; eventDate: 1.Ⅶ.–30.Ⅵ.2011; **Record Level:** collectionCode: Insects
- **Type status:** Other material. **Occurrence:** recordedBy: Peng Hou; individualCount: 1; sex: female; lifeStage: adult; occurrenceID: 5C77BDB2-C2F5-5FD9-AD17-041A9C0ACB3E; **Taxon:** scientificName: *Drino lota*; **Location:** country: China; stateProvince: Liaoning; locality: Laotuding, Huanren County, Benxi City; **Identification:** identifiedBy: Chuntian Zhang; dateIdentified: 2013; **Event:** samplingProtocol: sweeping; eventDate: 29.Ⅴ.2013; **Record Level:** collectionCode: Insects
- **Type status:** Other material. **Occurrence:** recordedBy: Yansen Zhang; individualCount: 1; sex: female; lifeStage: adult; occurrenceID: 8D7E36DD-D11D-5F32-B92C-5C741A4C53F6; **Taxon:** scientificName: *Drino lota*; **Location:** country: China; stateProvince: Liaoning; locality: Huangdaigou, Qingyuan County; verbatimElevation: 600–800 m; verbatimCoordinates: 41°83'N, 124°92'E; decimalLatitude: 42.38; decimalLongitude: 125.53; **Identification:** identifiedBy: Chuntian Zhang; dateIdentified: 2015; **Event:** samplingProtocol: sweeping; eventDate: 2.Ⅶ.–28.Ⅵ.2015; **Record Level:** collectionCode: Insects
- **Type status:** Other material. **Occurrence:** recordedBy: Xin L; individualCount: 1; sex: female; lifeStage: adult; occurrenceID: 5E214ECD-14EE-5694-807E-C00D11E98168; **Taxon:** scientificName: *Drino lota*; **Location:** country: China; stateProvince: Liaoning; locality: Huangdaigou, Qingyuan County; verbatimElevation: 600–800 m; verbatimCoordinates: 41°83'N, 124°92'E; decimalLatitude: 42.38; decimalLongitude: 125.53; **Identification:** identifiedBy: Chuntian Zhang; dateIdentified: 2015; **Event:** samplingProtocol: sweeping; eventDate: 2.Ⅶ.–28.Ⅵ.2015; **Record Level:** collectionCode: Insects
- **Type status:** Other material. **Occurrence:** recordedBy: Bing Li & Xu Chen; individualCount: 1; sex: male; lifeStage: adult; occurrenceID: F241D6D0-E182-5EDF-9AD5-0309A3B3EB65; **Taxon:** scientificName: *Drino lota*; **Location:** country: China; stateProvince: Liaoning; locality: Huangdaigou, Qingyuan County; verbatimElevation: 500–700 m; **Identification:** identifiedBy: Chuntian Zhang; dateIdentified: 2016; **Event:** samplingProtocol: sweeping; eventDate: 9–21.Ⅷ.2016; **Record Level:** collectionCode: Insects
- **Type status:** Other material. **Occurrence:** recordedBy: Houcan Liang; individualCount: 4; sex: female; lifeStage: adult; occurrenceID: 0D27DB5D-1519-53E8-92D8-87199C17C288; **Taxon:** scientificName: *Drino lota*; **Location:** country: China; stateProvince: Liaoning; locality: Baishilazi, Kuandian County, Dandong City; verbatimElevation: 435–740 m; verbatimCoordinates: 40°53'N, 124°52'E; decimalLatitude: 40.88; decimalLongitude: 124.87; **Identification:** identifiedBy: Chuntian Zhang; dateIdentified: 2017; **Event:** samplingProtocol: sweeping; eventDate: 18–22.Ⅵ.2017; **Record Level:** collectionCode: Insects
- **Type status:** Other material. **Occurrence:** recordedBy: Chuntian Zhang; individualCount: 1; sex: female; lifeStage: adult; occurrenceID: C3E6B823-8394-5296-80EF-8ACE31E453B1; **Taxon:** scientificName: *Drino lota*; **Location:** country: China; stateProvince: Liaoning; locality: Baishilazi, Kuandian County, Dandong City; verbatimElevation: 435–740 m; verbatimCoordinates: 40°53'N, 124°52'E; decimalLatitude: 40.88; decimalLongitude: 124.87; **Identification:** identifiedBy: Chuntian Zhang; dateIdentified: 2017; **Event:** samplingProtocol: sweeping; eventDate: 18–22.Ⅵ.2017; **Record Level:** collectionCode: Insects
- **Type status:** Other material. **Occurrence:** recordedBy: Ying Zhao; individualCount: 1; sex: male; lifeStage: adult; occurrenceID: C212AAC6-3CA0-5272-82CB-741DAB63222F; **Taxon:** scientificName: *Drino lota*; **Location:** country: China; stateProvince: Liaoning; locality: Mt. Huangyi, Kuandian County, Dandong City; verbatimElevation: 395–533 m; verbatimCoordinates: 40°43'N, 124°45'E; decimalLatitude: 40.72; decimalLongitude: 124.75; **Identification:** identifiedBy: Chuntian Zhang; dateIdentified: 2018; **Event:** samplingProtocol: sweeping; eventDate: 23–24.Ⅴ.2018; **Record Level:** collectionCode: Insects
- **Type status:** Other material. **Occurrence:** recordedBy: Chuntian Zhang; individualCount: 1; sex: female; lifeStage: adult; occurrenceID: F967B5D7-5948-552C-8F8B-BBC5BB2C95CF; **Taxon:** scientificName: *Drino lota*; **Location:** country: China; stateProvince: Sichuan; locality: Daochengkasi; verbatimElevation: 2750–3000 m; **Identification:** identifiedBy: Chuntian Zhang; dateIdentified: 2006; **Event:** samplingProtocol: sweeping; eventDate: 12.Ⅶ.2006; **Record Level:** collectionCode: Insects
- **Type status:** Other material. **Occurrence:** recordedBy: Bo Hao; individualCount: 1; sex: male; lifeStage: adult; occurrenceID: 08B92496-8664-5D52-8798-0D9C8566CBCD; **Taxon:** scientificName: *Drino lota*; **Location:** country: China; stateProvince: Yunnan; locality: Gaoligong Mountains, Gongshan; verbatimElevation: 1353–3013 m; verbatimCoordinates: 27°80'N, 98°50'E; decimalLatitude: 28.33; decimalLongitude: 98.83; **Identification:** identifiedBy: Chuntian Zhang; dateIdentified: 2015; **Event:** samplingProtocol: sweeping; eventDate: 12–15.Ⅴ.2015; **Record Level:** collectionCode: Insects
- **Type status:** Other material. **Occurrence:** recordedBy: Chuntian Zhang; individualCount: 1; sex: female; lifeStage: adult; occurrenceID: E4A309BC-BF20-5633-8A6B-B6F7DF59F18E; **Taxon:** scientificName: *Drino lota*; **Location:** country: China; stateProvince: Yunnan; locality: Shuidiping, Lanping, Nujiang Autonomous Prefecture; verbatimElevation: 1441 m; verbatimCoordinates: 26°29'N, 99°13'E; decimalLatitude: 26.48; decimalLongitude: 99.22; **Identification:** identifiedBy: Chuntian Zhang; dateIdentified: 2015; **Event:** samplingProtocol: sweeping; eventDate: 18.Ⅴ.2015; **Record Level:** collectionCode: Insects

#### Diagnosis

Body length (Fig. 34a, b) 8.0 mm. Eye (Fig. 34c) with ommatrichia about 4 facets in length. Frons of male about 0.72–0.79 times eye width, genal height 0.17–0.20 times eye height, antenna with postpedicel about 2 times as long as pedicel, occiput on upper half mostly with white hairs and without or sometimes with 1–2 black setulae behind postocular row, palpus yellow and gradually turning brown at base. Scutellum reddish yellow on apical 2/3. Vein M from bend to crossvein dm-cu longer than distance between bend and wing hind margin (2:1). Mid tibia with 1 *ad*. Tergite 5 with pruinosity at most on basal half, tergites 4 (larger) and 5 with sexual hair patches of appressed hairs ventrally. Surstylus bluntly rounded at apex and slightly longer than cerci in lateral view. (Photos of the holotype and syntype can be found in Global Biodiversity Information Facility 2025a, Global Biodiversity Information Facility 2025c. Postabdomen of male as in Fig. 35a and Fig. 35b)

#### Distribution

China (Provinces Gaungdong, Guangxi, Liaoning, Ningxia, Shanghai, Sichuan, Xizang, Yunnan, Zhejiang).

#### Remarks

We have examined the holotype deposited in IZCAS. *D. longihirta* Chao & Liang is similar to this species but differs in having frons of male about 0.7 times eye width, antenna with postpedicel about 3 times as long as pedicel, mid tibia with 2 *ad*, surstylus slightly pointed at apex, slightly shorter than cerci in lateral view.

### Drino minuta

Liang & Chao, 1998

CB123380-8529-5D37-AC2F-2C4C97AE725D

Drino minuta Liang & Chao in Chao et al., 1998. – Chao et al. 1998: 1850. – O’Hara et al. 2009: 67 (Catalog). – O’Hara et al. 2020a: 316 (Catalog). – O’Hara et al. 2020: 880 (Catalog). Type material deposited in IZCAS, examined.

#### Materials

- **Type status:** Holotype. **Occurrence:** recordedBy: Jielian Tang; individualCount: 1; sex: male; lifeStage: adult; occurrenceID: 287131A0-5A5D-569D-A755-6EF98092368D; **Taxon:** scientificName: *Drino minuta*; **Location:** country: China; stateProvince: Guangdong; locality: Zhanjiang City; **Identification:** identifiedBy: Jianming Zhao; dateIdentified: 1981; **Event:** eventDate: 29.Ⅴ.1981; **Record Level:** collectionCode: Insects
- **Type status:** Paratype. **Occurrence:** recordedBy: Xuelian Wu; individualCount: 1; sex: female; lifeStage: adult; occurrenceID: C947095C-033B-5309-B0ED-34E58CD55D0F; **Taxon:** scientificName: *Drino minuta*; **Location:** country: China; stateProvince: Guangdong; locality: Zhanjiang City; **Identification:** identifiedBy: Jianming Zhao; dateIdentified: 1981; **Event:** eventDate: Ⅶ.1981; **Record Level:** collectionCode: Insects
- **Type status:** Other material. **Occurrence:** recordedBy: Chao Fu; individualCount: 1; sex: female; lifeStage: adult; occurrenceID: FD86DAAB-6938-55CE-B3BF-DA147AC28CA8; **Taxon:** scientificName: *Drino minuta*; **Location:** country: China; stateProvince: Hebei; locality: Tangjiachang, Mt. Xiaowutai; verbatimElevation: 1200 m; **Identification:** identifiedBy: Chuntian Zhang; dateIdentified: 2009; **Event:** eventDate: 25.Ⅵ.2009; **Record Level:** collectionCode: Insects

#### Diagnosis

Body length (Fig. 36a, b) 6.0–7.5 mm. Frons (Fig. 36c, d) 0.8–0.9 times eye width, frontal vitta wider than fronto-orbital plate, ocellar triangle about 2/3 frons width, parafacial at its narrowest point slightly wider than postpedicel, genal height about 0.1 times eye heights, 2 reclinate orbital setae, occiput on upper half without black setulae behind postocular row, arista thickened on basal 3/5. Two lateral scutellar setae. Relative lengths of 2nd, 3rd and 4th costal sectors approximately in ratio of 5:8:5, vein M from bend to crossvein dm-cu longer than distance between bend and wing hind margin (23:17). Mid tibia with 1 *ad*, 2 posterior and 1 *v*. Tergites 4 and 5 with hair patches of appressed hairs ventrally. (Postabdomen of female as in Fig. 37a and Fig. 37b)

#### Distribution

China (Provinces Guangdong, Hebei, Liaoning, Sichuan, Yunnan).

#### Remarks

We have examined the holotype deposited in IZCAS. *D. densichaeta* Chao & Liang is similar to this species but differs in having frons about 0.86 times eye width, parafacial at narrowest point about 2.5 times postpedicel width, and genal height 0.2 times eye height.

### Drino parafacialis

Chao & Liang, 1998

D2A61243-0423-5F26-9BD2-3584A01FB72B

Drino parafacialis Liang & Chao in Chao et al., 1998. – Chao et al. 1998: 1852. – O’Hara et al. 2009: 67 (Catalog). – Zhang 2016: 243, 246 (key and descriptions). – O’Hara et al. 2020a: 316 (Catalog). – O’Hara et al. 2020: 880 (Catalog). Type material deposited in IZCAS, examined.

#### Materials

- **Type status:** Holotype. **Occurrence:** recordedBy: Xuekui Sun; individualCount: 1; sex: male; lifeStage: adult; occurrenceID: 6A5E9772-3347-54A8-9A5B-A6D88C379CFD; **Taxon:** scientificName: *Drino parafacialis*; **Location:** country: China; stateProvince: Zhejiang; locality: Tianmu mountains; **Identification:** identifiedBy: Jianming Zhao; dateIdentified: 1987; **Event:** eventDate: 11.Ⅵ.1987; **Record Level:** collectionCode: Insects
- **Type status:** Paratype. **Occurrence:** recordedBy: Keren Huang; individualCount: 1; sex: male; lifeStage: adult; occurrenceID: 4F9EAFAE-F3F7-58BA-A817-C9DE7C1BAA5D; **Taxon:** scientificName: *Drino parafacialis*; **Location:** country: China; stateProvince: Zhejiang; locality: Tianmu mountains; **Identification:** identifiedBy: Jianming Zhao; dateIdentified: 1957; **Event:** eventDate: 26.Ⅵ.1957; **Record Level:** collectionCode: Insects
- **Type status:** Paratype. **Occurrence:** recordedBy: Keren Huang; individualCount: 1; sex: male; lifeStage: adult; occurrenceID: 45C077E8-14F1-5523-A75B-3BB3D10643FE; **Taxon:** scientificName: *Drino parafacialis*; **Location:** country: China; stateProvince: Sichuan; locality: Emei mountains; **Identification:** identifiedBy: Jianming Zhao; dateIdentified: 1957; **Event:** eventDate: 4.Ⅵ.1957; **Record Level:** collectionCode: Insects
- **Type status:** Other material. **Occurrence:** recordedBy: Chuntian Zhang; individualCount: 1; sex: male; lifeStage: adult; occurrenceID: D62C90C3-509F-5ABE-8CDB-949B51BEB24D; **Taxon:** scientificName: *Drino parafacialis*; **Location:** country: China; stateProvince: Guangdong; locality: Ruyuan, Nanling; verbatimElevation: 720–1020 m; verbatimCoordinates: 24°55'43"N, 113°00'59"E; decimalLatitude: 24.92; decimalLongitude: 113.02; **Identification:** identifiedBy: Chuntian Zhang; dateIdentified: 2024; **Event:** eventDate: 22.Ⅴ.2024; **Record Level:** collectionCode: Insects
- **Type status:** Other material. **Occurrence:** recordedBy: Bo Hao; individualCount: 1; sex: male; lifeStage: adult; occurrenceID: 47041B39-0501-5EF9-8720-E3A0406AB4EC; **Taxon:** scientificName: *Drino parafacialis*; **Location:** country: China; stateProvince: Guangdong; locality: Ruyuan, Shaoguan City; verbatimElevation: 480–1010 m; verbatimCoordinates: 24°52'N, 113°01'E; decimalLatitude: 24.87; decimalLongitude: 113.02; **Identification:** identifiedBy: Chuntian Zhang; dateIdentified: 2020; **Event:** eventDate: 26–28.Ⅶ.2020; **Record Level:** collectionCode: Insects
- **Type status:** Other material. **Occurrence:** recordedBy: Qiang Wang; individualCount: 1; sex: male; lifeStage: adult; occurrenceID: 62C0A439-79F9-51AB-A41A-951952AB3B50; **Taxon:** scientificName: *Drino parafacialis*; **Location:** country: China; stateProvince: Liaoning; locality: Qingliang Mountains, Xiuyan County; verbatimElevation: 300–600 m; **Identification:** identifiedBy: Chuntian Zhang; dateIdentified: 2013; **Event:** eventDate: 10.Ⅵ.2013; **Record Level:** collectionCode: Insects
- **Type status:** Other material. **Occurrence:** recordedBy: Bing Li; individualCount: 1; sex: male; lifeStage: adult; occurrenceID: 6D9C2FEC-3B91-5FF0-8A2C-5A3C73B5F7B6; **Taxon:** scientificName: *Drino parafacialis*; **Location:** country: China; stateProvince: Liaoning; locality: Huangdaigou, Qingyuan County; verbatimElevation: 600–800 m; verbatimCoordinates: 41°83'N, 124°92'E; decimalLatitude: 42.38; decimalLongitude: 125.53; **Identification:** identifiedBy: Chuntian Zhang; dateIdentified: 2015; **Event:** eventDate: 26–31.Ⅴ.2015; **Record Level:** collectionCode: Insects
- **Type status:** Other material. **Occurrence:** recordedBy: Chuntian Zhang; individualCount: 3; sex: male; lifeStage: adult; occurrenceID: 43A3003B-23C3-5A8E-A6B0-FB23A1B45FE5; **Taxon:** scientificName: *Drino parafacialis*; **Location:** country: China; stateProvince: Liaoning; locality: Mt. Huangyi; verbatimElevation: 450–550 m; verbatimCoordinates: 40°73'N, 124°75'E; decimalLatitude: 41.22; decimalLongitude: 125.25; **Identification:** identifiedBy: Chuntian Zhang; dateIdentified: 2020; **Event:** eventDate: 14.Ⅵ.2020; **Record Level:** collectionCode: Insects
- **Type status:** Other material. **Occurrence:** recordedBy: Shidi Wang; individualCount: 1; sex: female; lifeStage: adult; occurrenceID: A76233D2-2A1A-5B77-8476-ADB05D6312CC; **Taxon:** scientificName: *Drino parafacialis*; **Location:** country: China; stateProvince: Shaanxi; locality: Mt. Taibai, Baoji; verbatimElevation: 600–2800 m; **Identification:** identifiedBy: Chuntian Zhang; dateIdentified: 2010; **Event:** eventDate: 17–19.Ⅶ.2010; **Record Level:** collectionCode: Insects

#### Diagnosis

Body length (Fig. 38a, b) 7.5–12.0 mm. Frons (Fig. 38c, d) of male about 0.8 times eye width, parafacial at narrowest point only slightly wider than postpedicel width, genal height about 1/10 eye height, outer vertical seta almost as long as postocular setula, 2–3 frontal setae descending to parafacial and lower level of pedicel, occiput with sparse black setulae behind postocular row. Relative lengths of 2nd, 3rd and 4th costal sectors approximately in ratio of 37:50:28, vein M from bend to crossvein dm-cu longer than distance between bend and wing hind margin (29:18). Mid tibia with 1 *ad*, 2 posterior and 1 *v*. Tergites 4 and 5 with large patches of appressed sexual hairs ventrally. Cerci (Fig. 39a) evenly narrowed, blunt at apex, not separated apically in caudal view, in lateral view cerci nearly straight, narrowed to apex. Surstylus slightly longer than cerci, bluntly rounded at apex in caudal view, slightly blunt in lateral view. Cerci about 1/2 of surstyli width and without an obvious notch near middle in lateral view. Pregonite (Fig. 39b) long and slightly curved; postgonite slender; epiphallus short and hook-like at apex, slightly shorter than postgonite; basiphallus and distiphallus distinctly sclerotized; distiphallus weakly expanded in lateral view.

#### Distribution

China (Provinces Guangdong, Liaoning, Shaanxi, Sichuan, Zhejiang).

#### Remarks

We have examined the holotype deposited in IZCAS. *D. densichaeta* Chao & Liang is similar to this species but differs in having parafacial at narrowest point narrower than postpedicel width, frons of male about 0.6 times eye width, and occiput without sparse black setulae behind postocular row.

## Identification Keys

### Key to three subgenera of *Drino* Robineau-Desvoidy, 1863 (Revised after Crosskey 1967)

**Table:** 

1	Ocellar setae strong, equal or subequal in size to reclinate orbital setae, when present inserted at all forwards, or all backwards, or form a straight line with the anterior ocellus. Eye bare. Parafacial bare.	Subgenus **Zygobothria Mik, 1891**
–	Ocellar setae absent, or if present thinner or shorter than reclinate orbital setae and inserted behind of the anterior ocellus. Eye bare, or with ommatrichia about two or four facet length. Parafacial bare or finely haired on upper parts (below lowest frontal seta).	2
2	Parafacial finely haired on upper part, hair sometimes extending on to lower part, occasionally only a very few minute hairs immediately below lowest frontal setae but parafacial never entirely bare. Ocellar setae present, short and thin (*D. laetifica* exceptional with ocellar setae absent).	Subgenus **Palexorista Townsend, 1921**
–	Parafacial completely bare. Ocellar setae absent (*D. parafacialis* exceptional with ocellar setae is variable, thin, short or absent).	Subgenus ***Drino* Robineau-Desvoidy, 1863**

### Key to species of subgenus *Drino* Robineau-Desvoidy from China

**Table:** 

1	Male with 2 reclinate orbital setae.	2
–	Male with 3–4 or more reclinate orbital setae.	4
2	Frons at most 0.9 times eye width.	3
–	Frons as long as or longer than eye width, ocellar triangle length about 2/3 of frons width; 2 lateral scutellar setae.	***D. minuta* Liang & Chao**
3	Frons about 0.86 eye width, occiput with 1–2 rows of black setulae behind postocular row, tergite 4 with 15 rows of hairs dorsally (as in Fig. 18a).	***D. densichaeta* Chao & Liang**
–	Frons about 0.50 of eye width, occiput mostly with white hairs without a row of black setulae behind postocular row, tergite 4 with sparse hairs dorsally, not dense as above.	***D. oharai* sp.nov**.
4	Tergites 4 and 5 with patches of appressed hairs ventrally.	5
–	Tergites 4 and 5 without patches of appressed hairs ventrally.	14
5	Eye with ommatrichia about 4 facets in length.	6
–	Eye bare, or with short ommatrichia about 2 facets in length.	7
6	Frons of male about 0.6 times of eye width, antenna with postpedicel about 2 times as long as pedicel, mid tibia with 1 *ad*, surstylus blunt round at apex, slightly longer than cerci in lateral view.	***D. lota* (Meigen)**
–	Frons of male about 0.7 times of eye width, antenna with postpedicel about 3 times as long as pedicel, mid tibia with 2 *ad*, surstylus slightly pointed at apex, slightly shorter than cerci in lateral view.	***D. longihirta* Chao & Liang**
7	Abdomen yellow on both sides of syntergite 1+2 to tergite 4, tergites 3 and 4 with 1/4–1/3 square or trapezoidal dorsal black spot at middle (Fig. 22a), tergite 3 without black band at posterior margin, tergite 4 on posterior 1/4 and tergite 5 gleaming black, abdomen with pale gray pruinosity.	***D. flava* Chao & Liang**
–	Abdomen mainly black, syntergite 1+2 to tergite 4 more or less reddish yellow on both sides, tergites 3 and 4 each with black band on posterior margin, abdomen with dark gray or brown gray pruinosity.	8
8	Frons of male wider than eye width.	9
–	Frons of male narrower than eye width.	10
9	Fronto-orbital plate with a row of 5–7 reclinate orbital setae, mid tibia with 3–4 *ad*, 3 *p* and 2 *v*, srustyli shorter than cerci in lateral view.	***D. latifrons* Liu, Zhang & Xi**
–	Fronto-orbital plate with a row of 3–4 reclinate orbital setae, Mid tibia with 4–5 *ad*, 2 *p* and 2 *v*, srustyli about same length as cerci in lateral view.	***D. adiscalis* (Chao)**
10	Parafacial at narrowest point narrower than postpedicel width, frons of male about 3/5 of eye width, scutellum brown on apical half, abdomen covered with dense pale yellowish pruinosity.	***D. facialis* (Townsend)**
–	Parafacial at narrowest point wider than postpedicel width, frons of male about 7/10–9/10 of eye width, scutellum brown or light yellow on apical half, abdomen covered with silvery pruinosity, varying from dense to sparse.	11
11	Genal height 1/6 of eye height or less.	12
–	Genal height distinctly more than 1/6 of eye height.	13
12	Frons about 0.8 times of eye width, occiput with sparse black setulae behind postocular row, cerci about 1/2 of surstyli width and without an obvious notch near middle in lateral view.	***D. parafacialis* Chao & Liang**
–	Frons about 0.7 times of eye width, occiput without black setulae behind postocular row, cerci about about 2/3 of surstyli width and with an obvious notch near middle in lateral view.	***D. meridionalis* sp. nov**.
13	Outer vertical seta hair-like and not distinctly longer than postocular setulae, parafacial at narrowest point 1.6–1.7 times antenna width, genal height about 1/5 eye height, occiput with 5–7 black setulae behind postocular row.	***D. uniseta* sp. nov**.
–	Outer vertical seta about 1/2 as long as inner vertical seta, parafacial at narrowest point 1.5 times antenna width, genal height about 1/4 eye height, occiput with 2 rows of black setulae behind postocular row.	***D. liaoningensis* sp. nov**.
14	Frons of male about 1.2 times of eye width, palpus black, occiput with almost a completely row of black setulae behind postocular row, parafacial at narrowest point shorter than antenna width, tergite 4 with dense and thin hairs but without hair patches.	***D. laticornis* Chao & Liang**
–	Frons of male at most 0.6 times of eye width, palpus yellow or brown, occiput mostly without a completely row of black setulae behind postocualr row except *D. longicapilla*, parafacial at narrowest point shorter or longer than antenna width, tergite 4 without hair patches.	15
15	Frons of male about 1/3 of eye width, frontal vitta 1.4 times wider than fronto-orbital plate at middle, 4 reclinate orbital setae, antenna with postpedicel about 2.6 times as long as pedicel, arista thickened on basal 2/5, palpus dark brown.	***D. interfrons* (Sun & Chao)**
–	Frons of male wider than 1/3 of eye width, frontal vitta narrower than or subequal to fronto-orbital plate, 2–4 reclinate orbital setae, postpedicel 2.7–3.2 times as long as pedicel, arista thickened on basal 1/6–4/9, and palpus pale brown to yellow.	16
16	Head, thorax, abodomen covered with dense dark golden yellow pruinosity dorsally. Eye with sparse ommatrichia about 4 facets in length.	17
–	Head, thorax, abodomen covered with gray pruinosity dorsally. Eye with sparse and inconspicuous ommatrichia shorter than 2 facets in length.	18
17	Fronto-orbital plate convex, with dense and long black hairs, parafacial at narrowest point about 1.7 times postpedicel width, pruinosity thin and shiny on abdomen, tergites 3 and 4 with pruinosity on basal 1/3, tergite 4 on posterior 2/3 and tergite 5 gleaming black.	***D. longicapilla* Chao & Liang**
–	Fronto-orbital plate flat, with sparse and short black hairs, parafacial at narrowest point slightly wider than postpedicel width, pruinosity dense on abdomen, tergite 3 covered with pruinosity on basal 1/2 and tergite 4 on basal 2/5, tergite 4 on posterior 3/5 and tergite 5 gleaming black.	***D. auripollinis* Chao & Liang**
18	Frons of male narrower than 1/2 eye width, postpedicel dark brown in ground color (as in Fig. 12c), mid tibia with 2 *p*, tergite 5 covered with dense pruinosity on basal half.	***D. angustivitta* Liang & Chao**
–	Frons of male 3/5 eye width, postpedicel pale brown in ground color, mid tibia with 3 *p*, tergite 5 covered with dense pruinosity on basal 3/5.	***D. argenticeps* (Macquart, 1851)**

Key to males

## Supplementary Material

XML Treatment for Drino

XML Treatment for Drino liaoningensis

XML Treatment for Drino meridionalis

XML Treatment for Drino oharai

XML Treatment for Drino uniseta

XML Treatment for Drino adiscalis

XML Treatment for Drino angustivitta

XML Treatment for Drino argenticeps

XML Treatment for Drino auripollinis

XML Treatment for Drino densichaeta

XML Treatment for Drino facialis

XML Treatment for Drino flava

XML Treatment for Drino interfrons

XML Treatment for Drino laticornis

XML Treatment for Drino latifrons

XML Treatment for Drino longicapilla

XML Treatment for Drino longihirta

XML Treatment for Drino lota

XML Treatment for Drino minuta

XML Treatment for Drino parafacialis

## Figures and Tables

**Figure 1.**
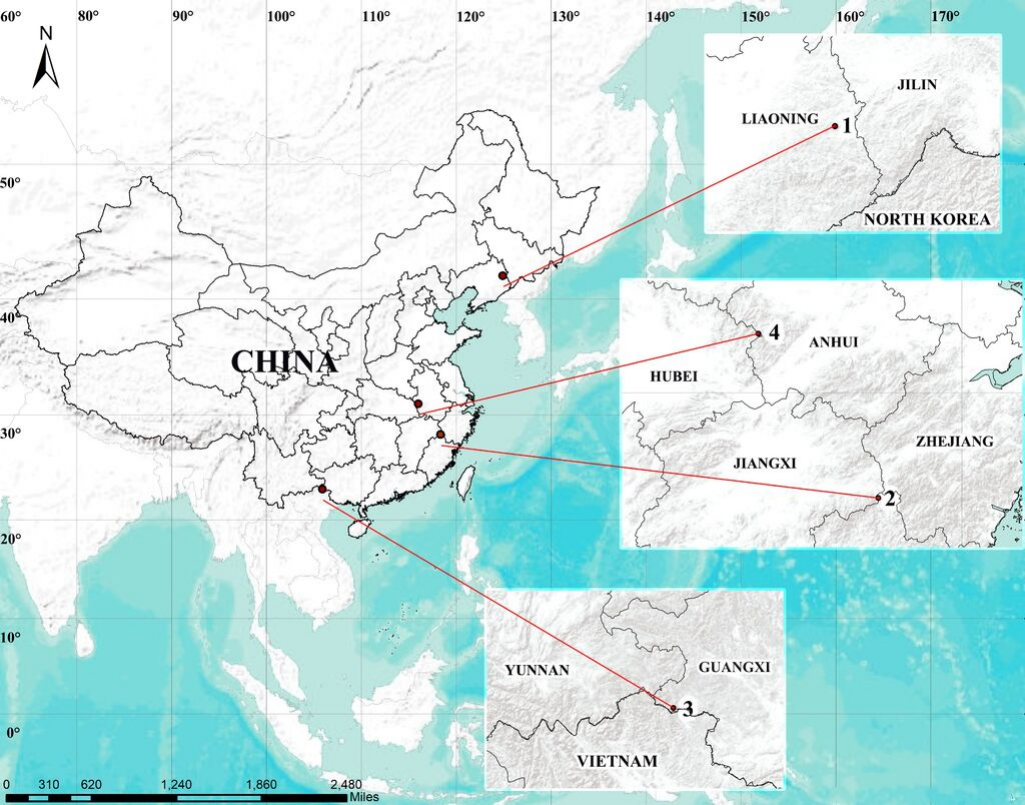
Geographic distributions of four *Drino* new species from China. **1** - *Drino liaoningensis* sp. nov. (holotype from Liaoning Province, China); **2** - *Drino uniseta* sp. nov. (paratype from Zhejiang Province, China); **3** - *Drino meridionalis* sp. nov. (holotype from Guangxi Autonomous Region, China); **4** - *Drino oharai* sp. nov. (holotype from Hubei Province, China).

**Figure 2a.**
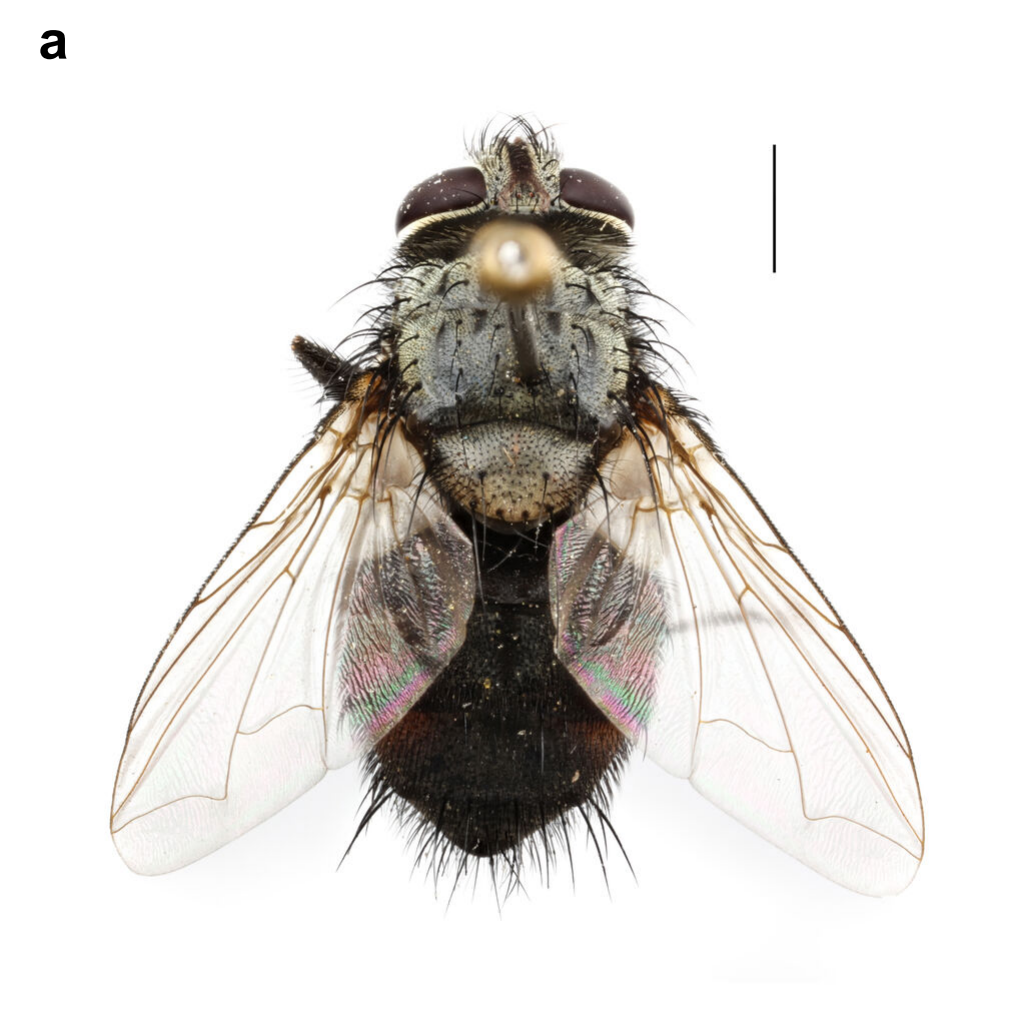
male body in dorsal view.

**Figure 2b.**
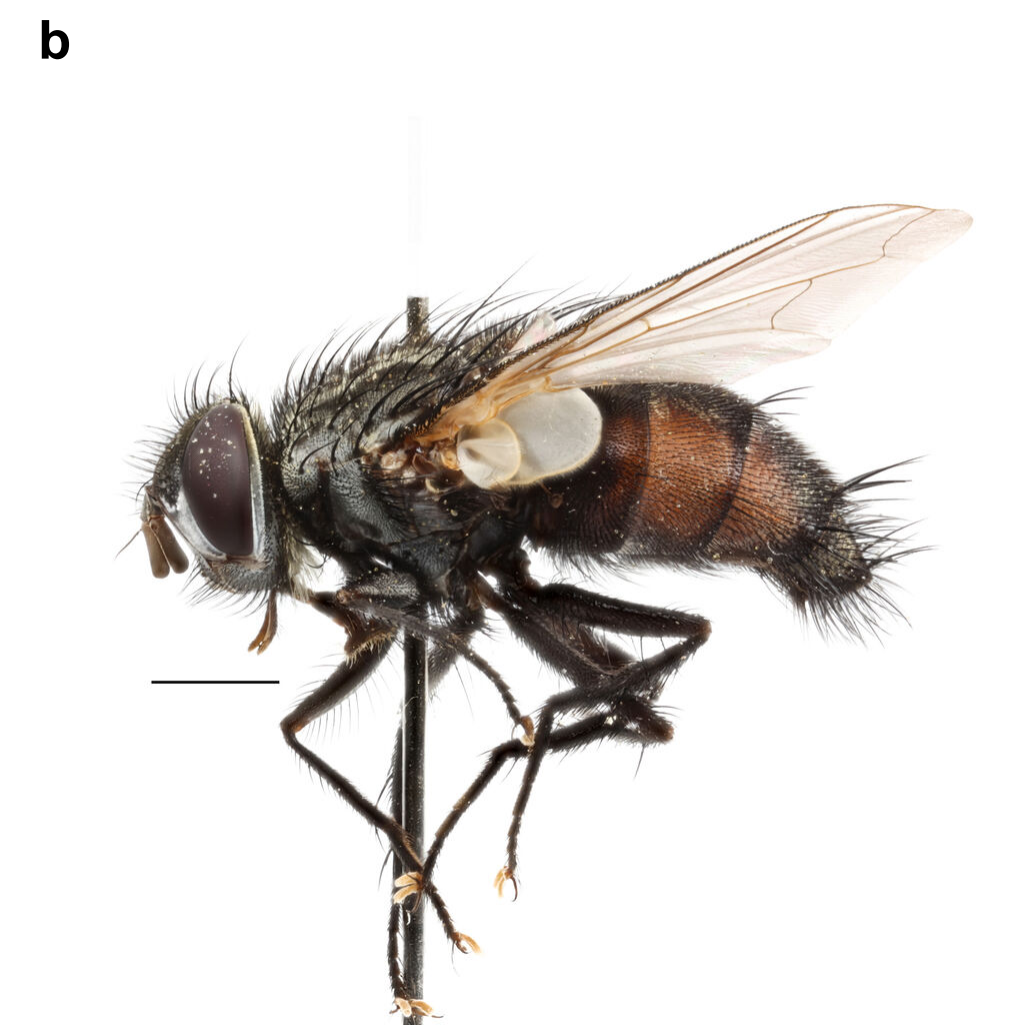
male body in lateral view.

**Figure 2c.**
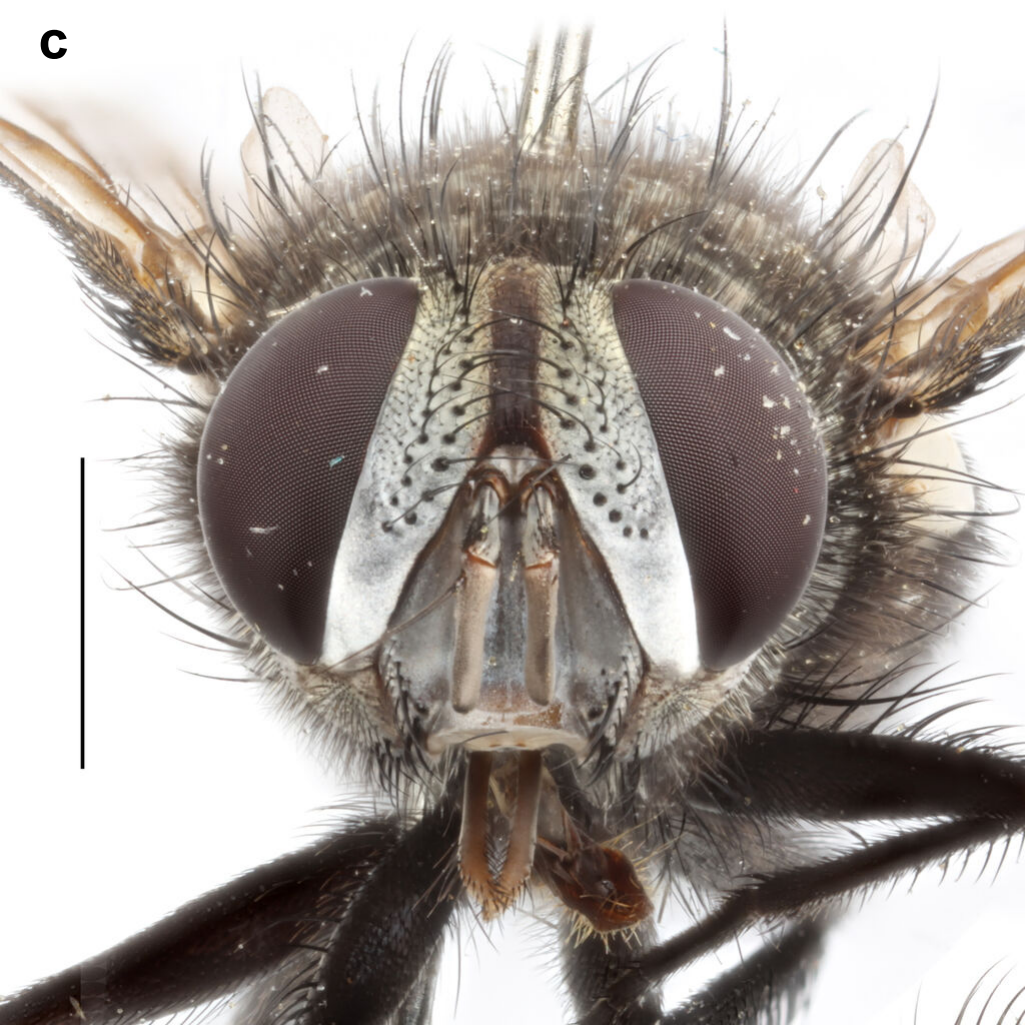
male head in anterior view.

**Figure 2d.**
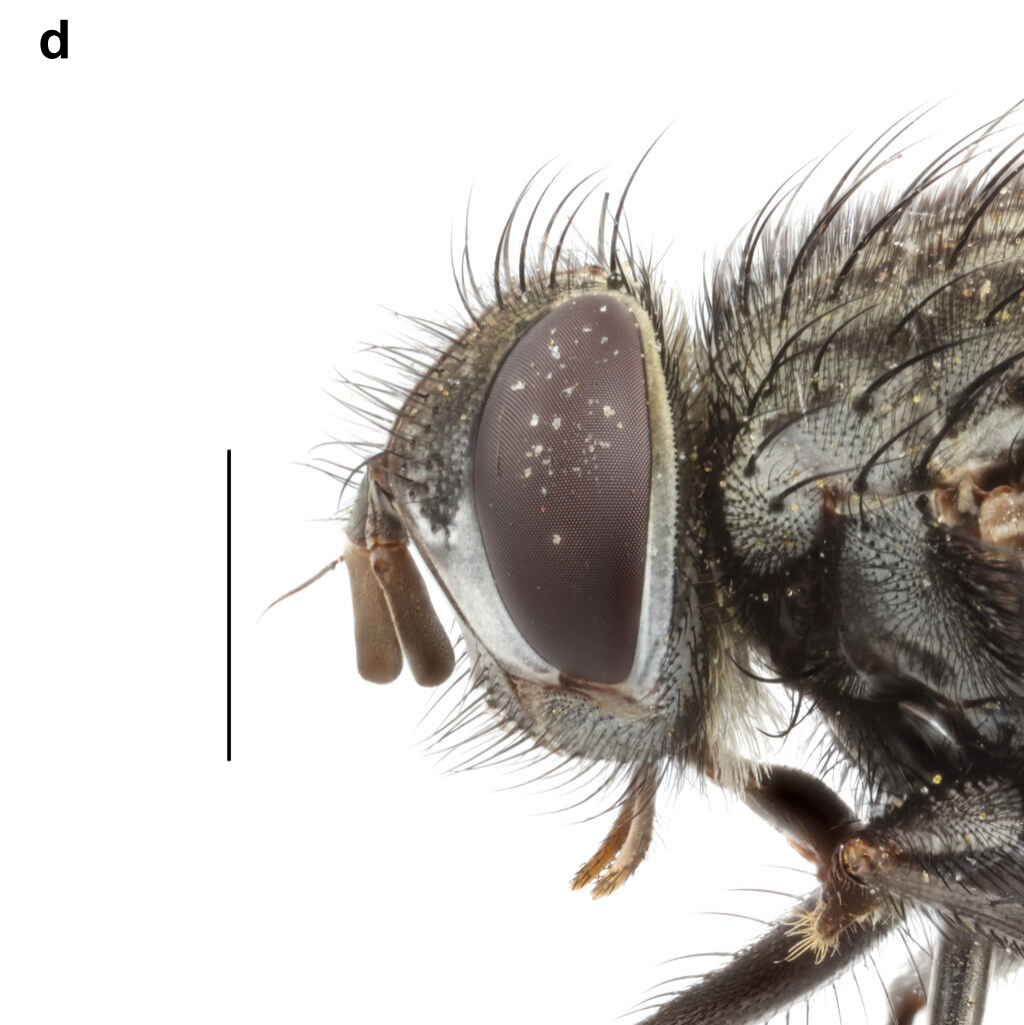
male head in lateral view.

**Figure 3a.**
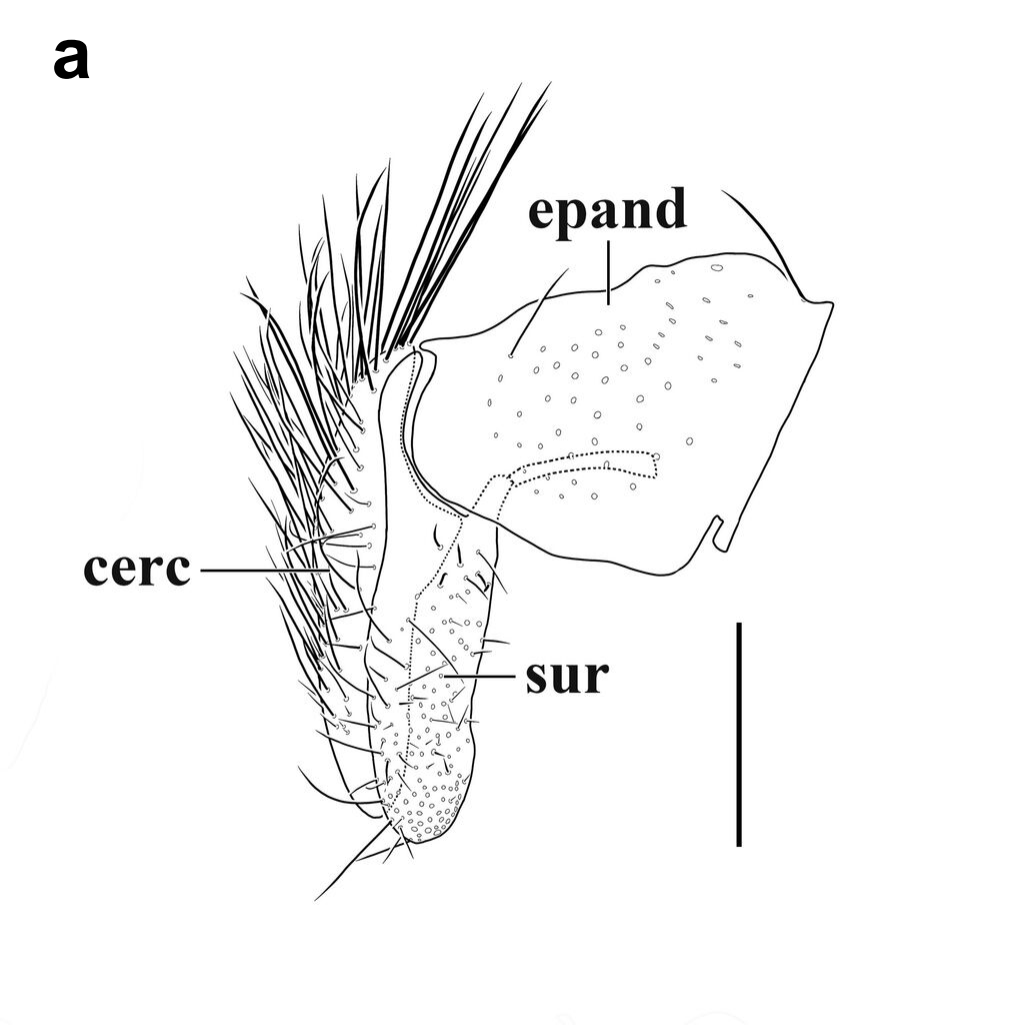
male cercus, surstylus and epandrium in lateral view.

**Figure 3b.**
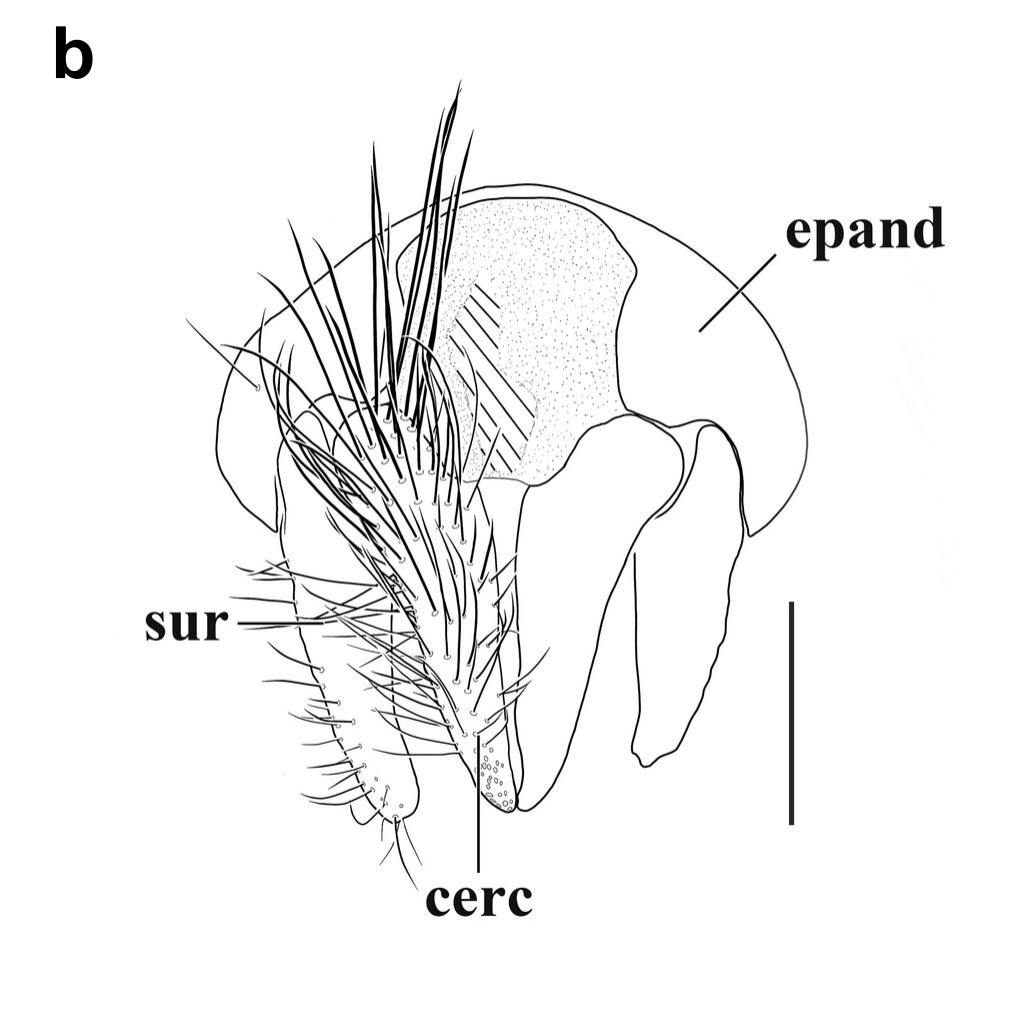
male cerci, surstyli and epandrium in caudal view.

**Figure 3c.**
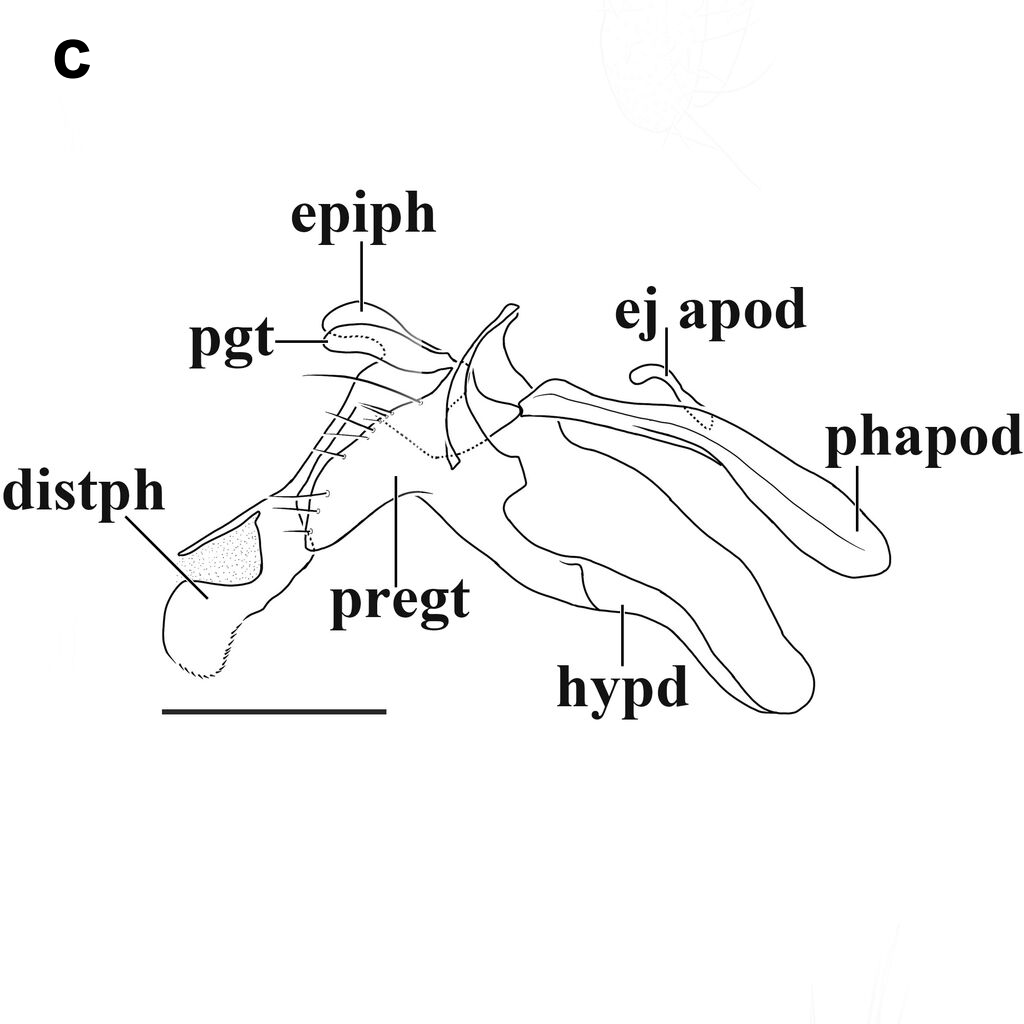
male phallus (basiphallus, distiphallus, ejaculatory apodeme, epiphallus, hypandrium, phallapodeme, postgonite, pregonite) in lateral view.

**Figure 3d.**
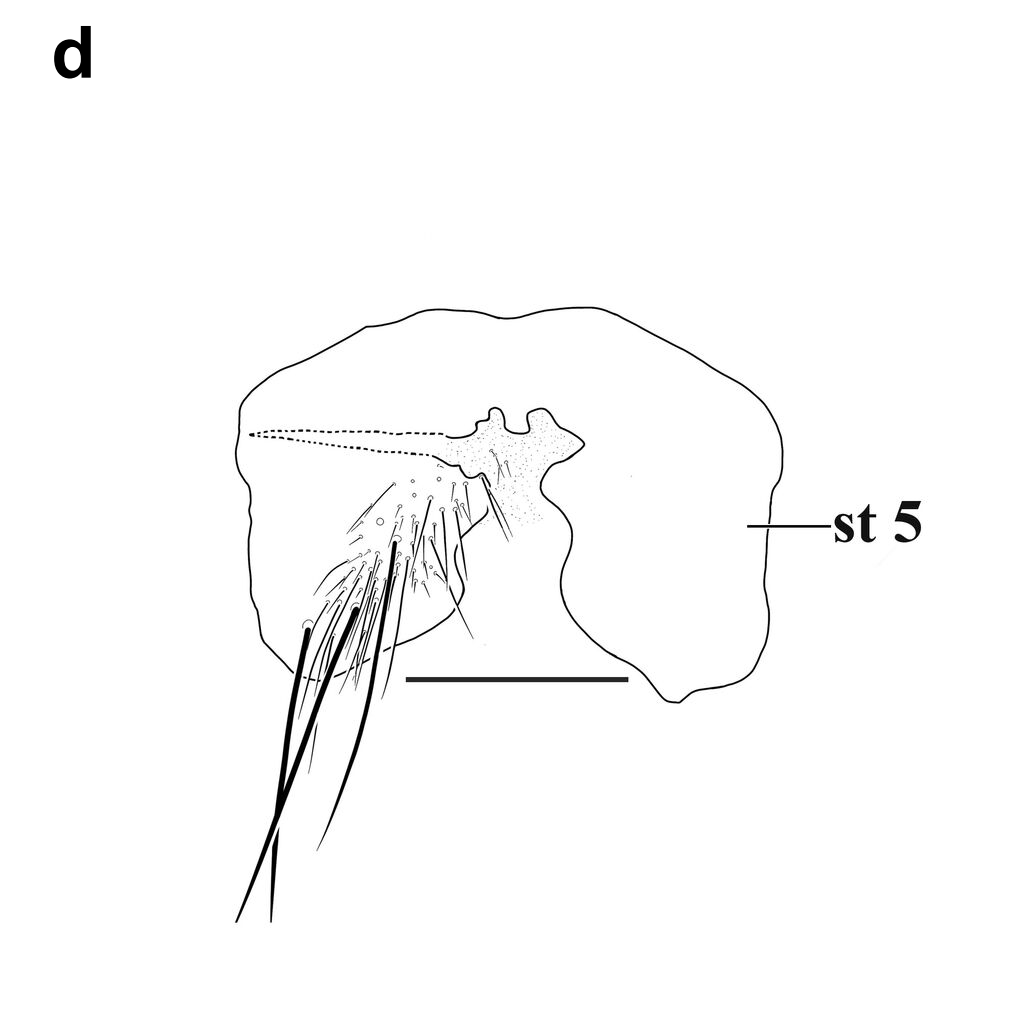
male sternite 5 in ventral view.

**Figure 4a.**
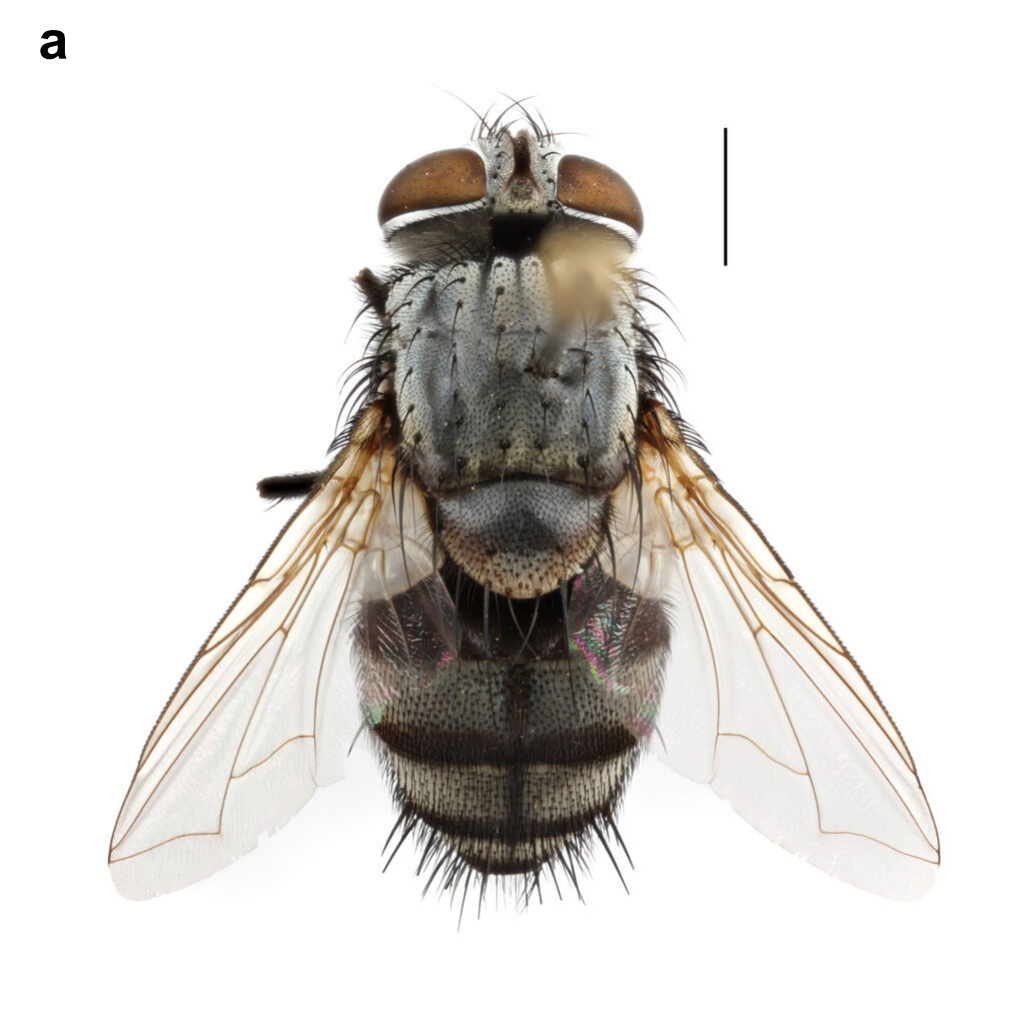
male body in dorsal view.

**Figure 4b.**
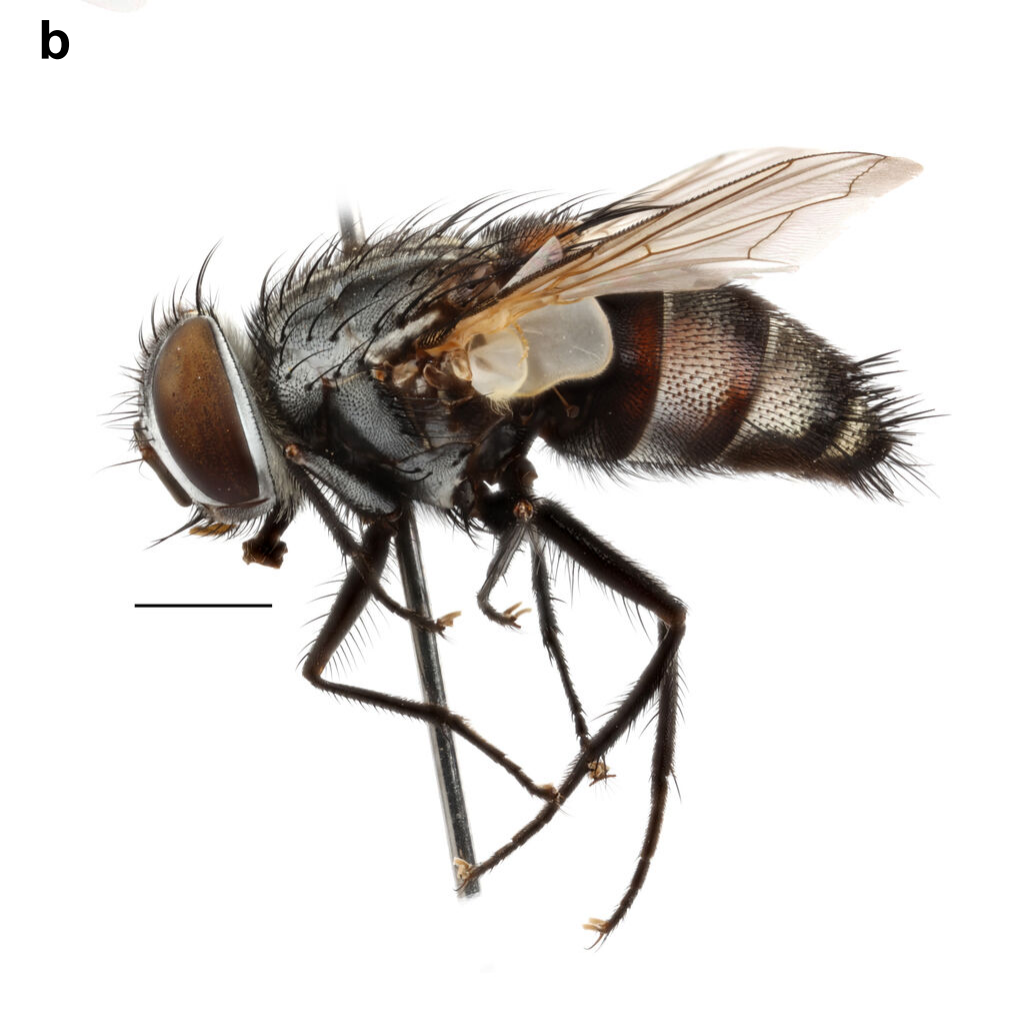
male body in lateral view.

**Figure 4c.**
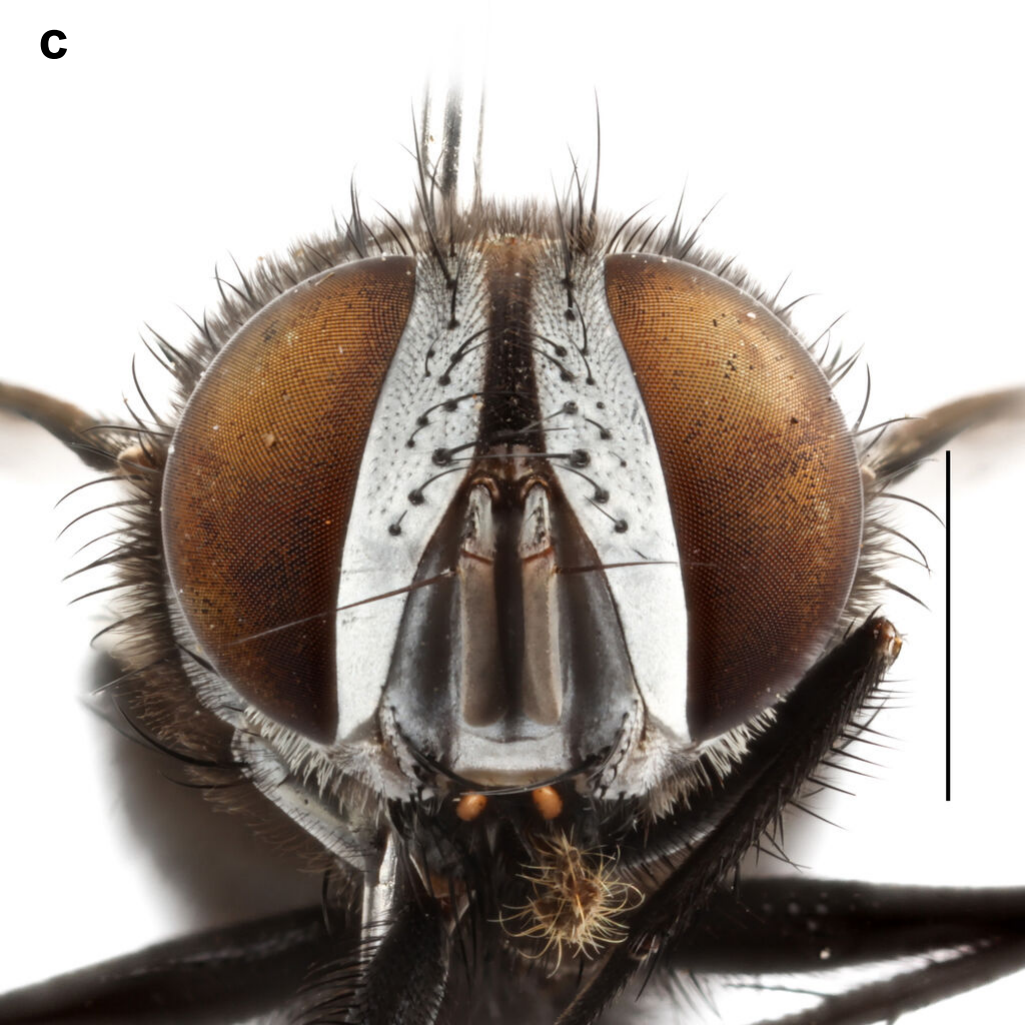
male head in anterior view.

**Figure 4d.**
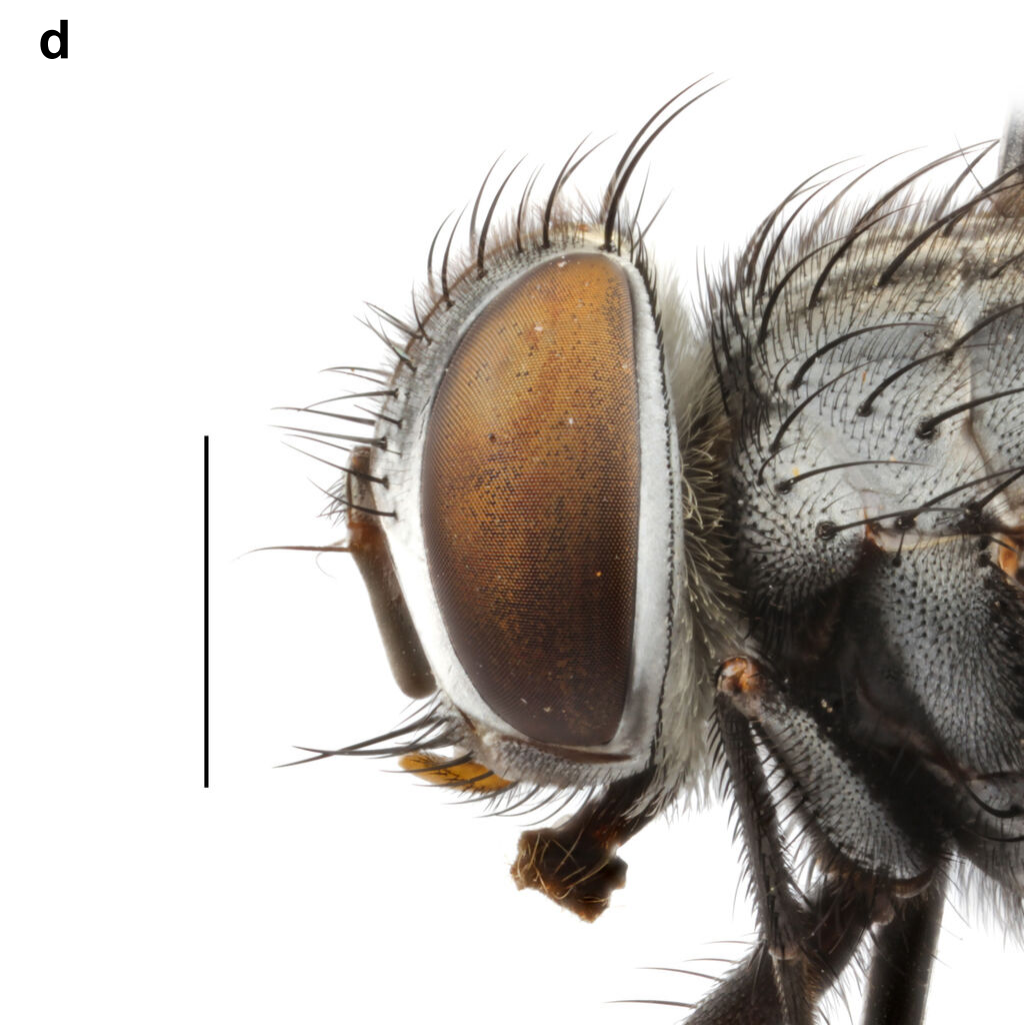
male head in lateral view.

**Figure 5a.**
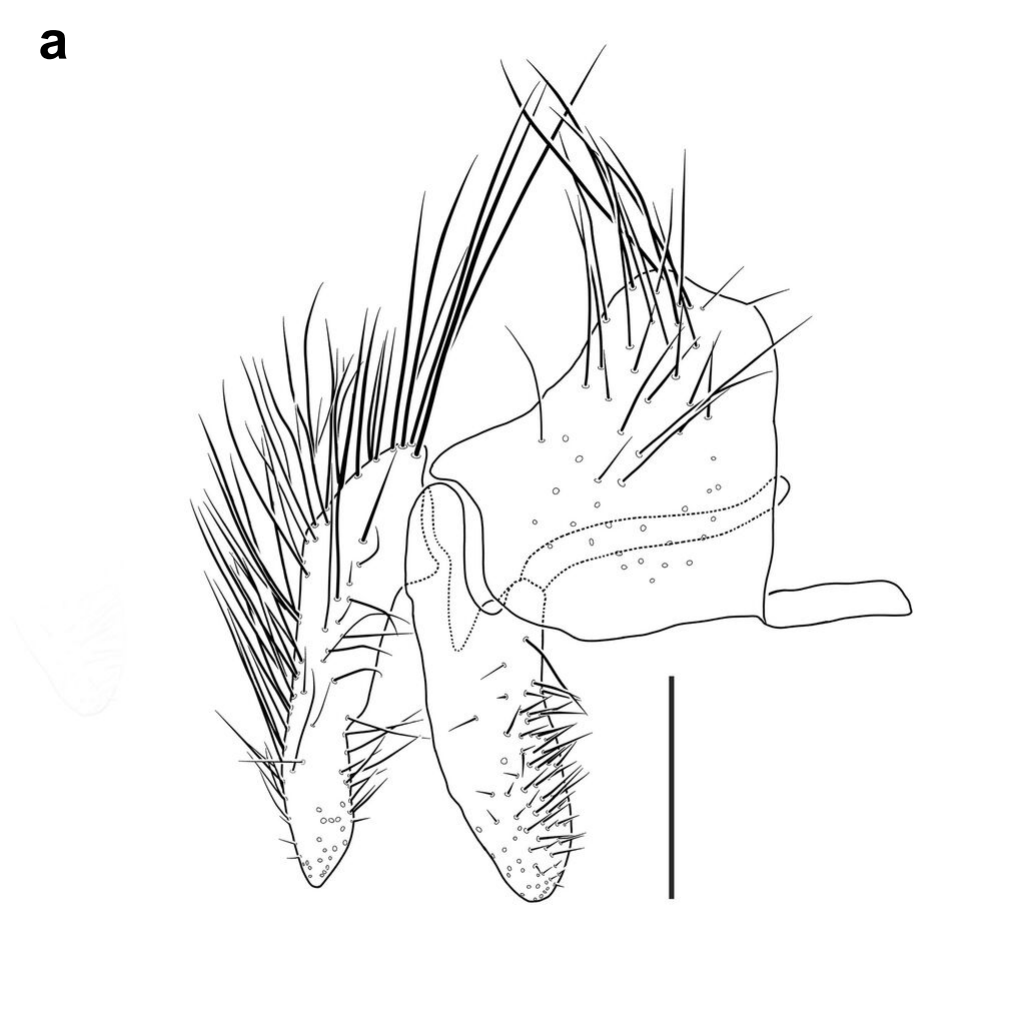
male cercus, surstylus and epandrium in lateral view.

**Figure 5b.**
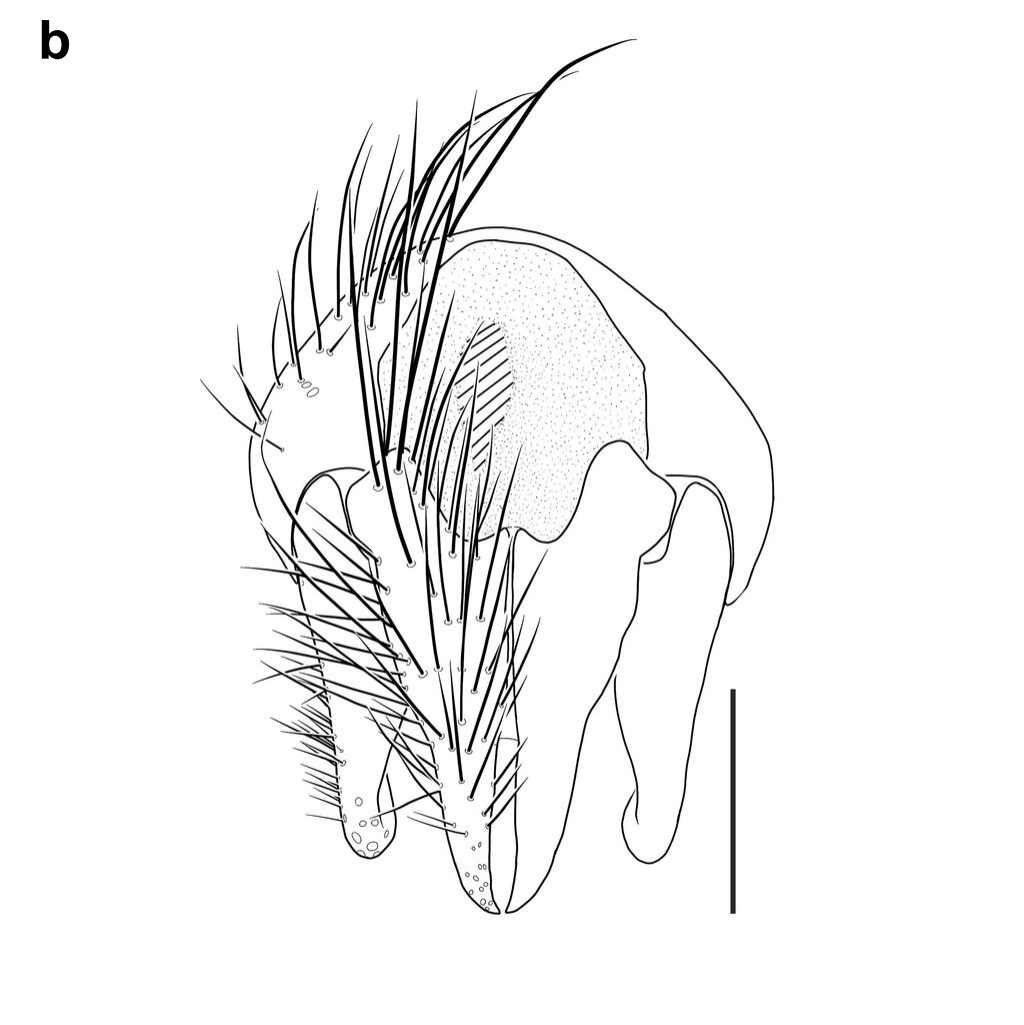
male cerci, surstyli and epandrium in caudal view.

**Figure 5c.**
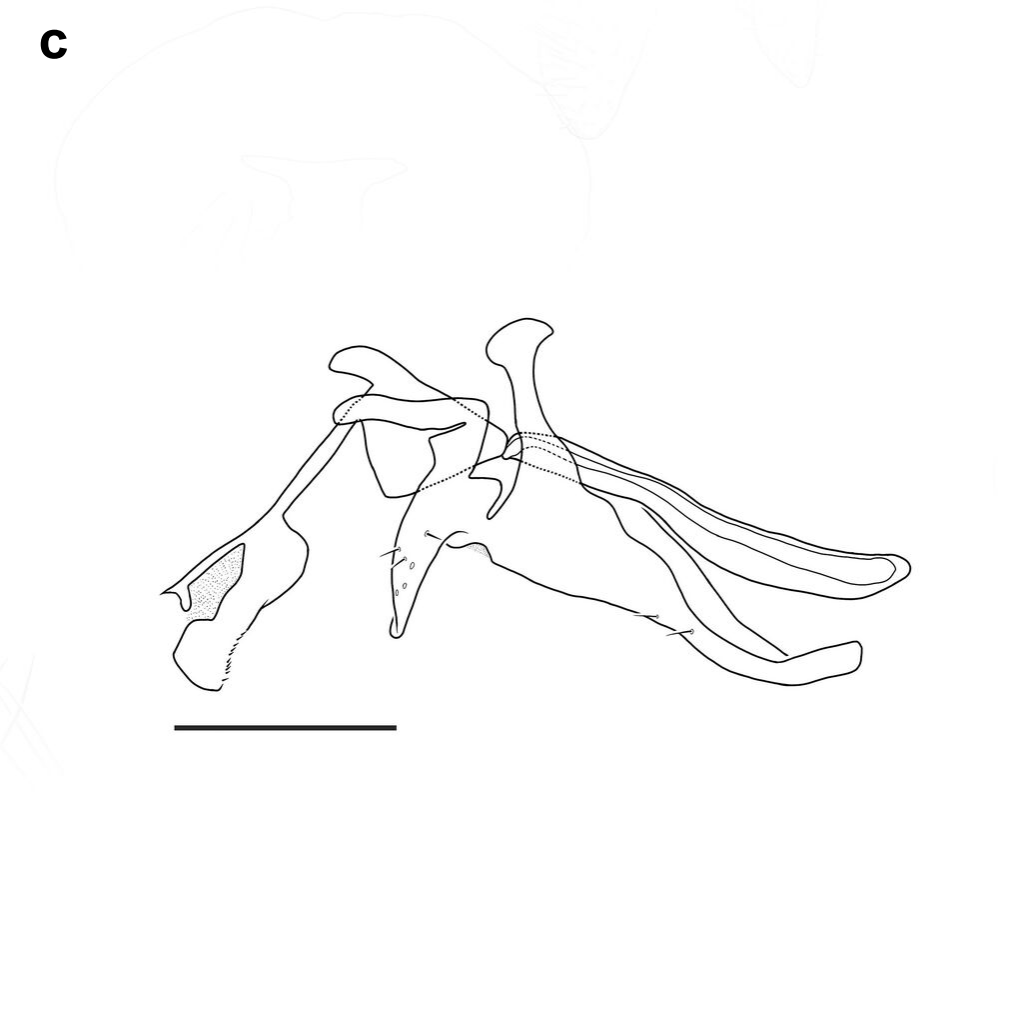
male phallus (basiphallus, distiphallus, ejaculatory apodeme, epiphallus, hypandrium, phallapodeme, postgonite, pregonite) in lateral view.

**Figure 5d.**
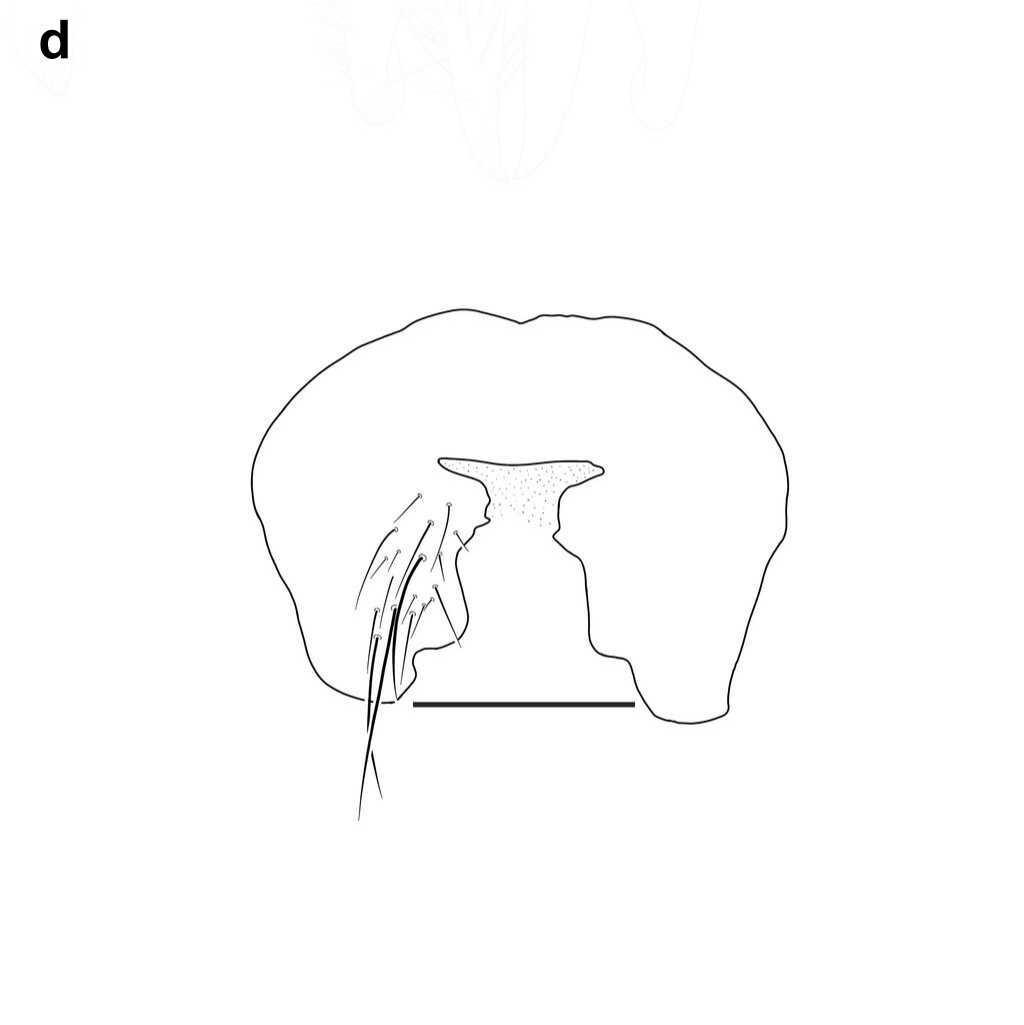
male sternite 5 in ventral view.

**Figure 6a.**
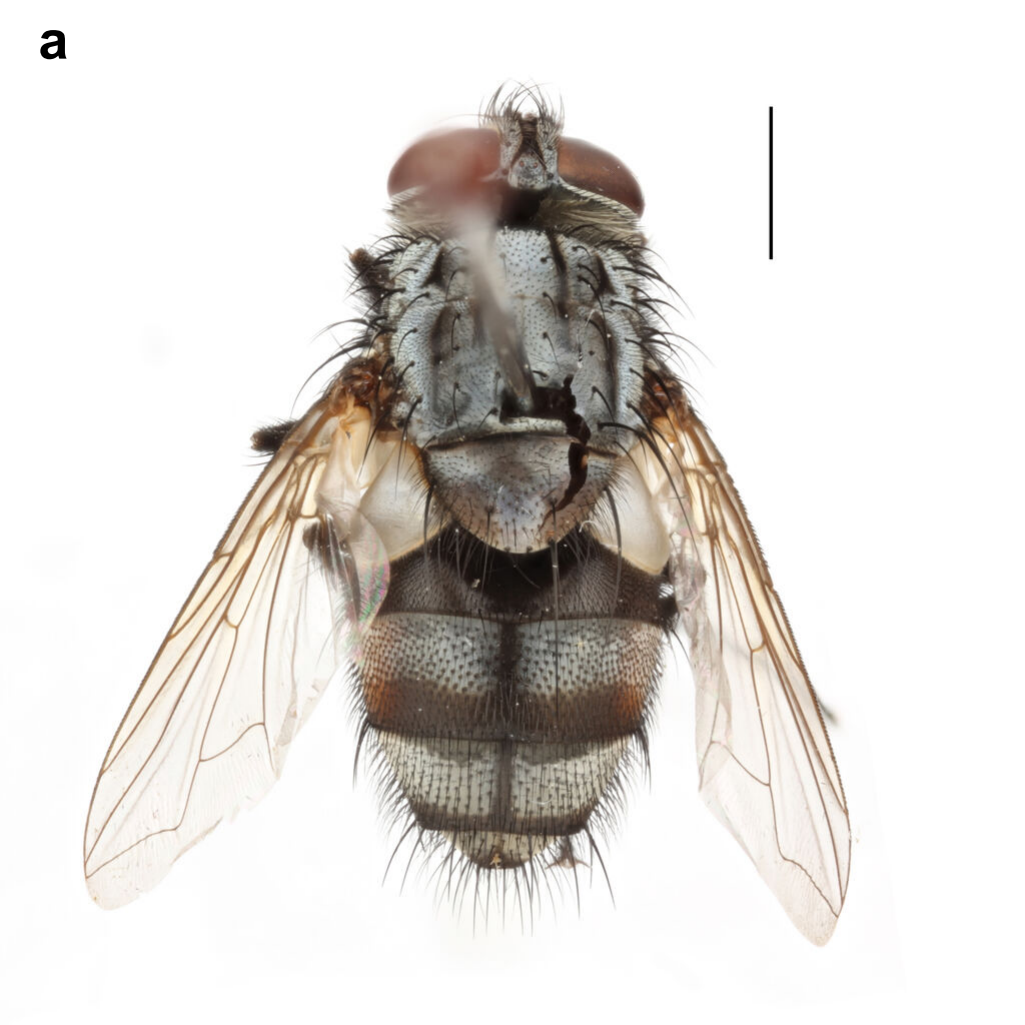
male body in dorsal view.

**Figure 6b.**
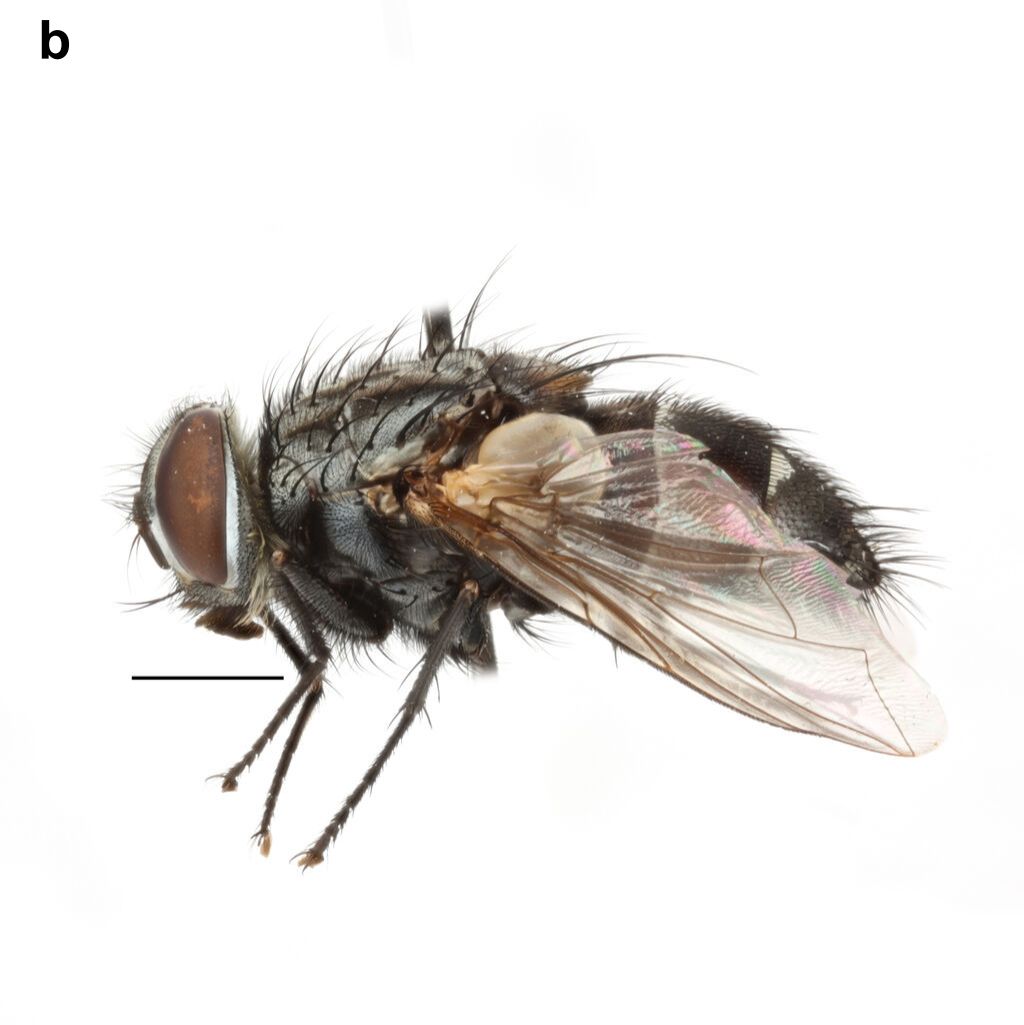
male body in lateral view.

**Figure 6c.**
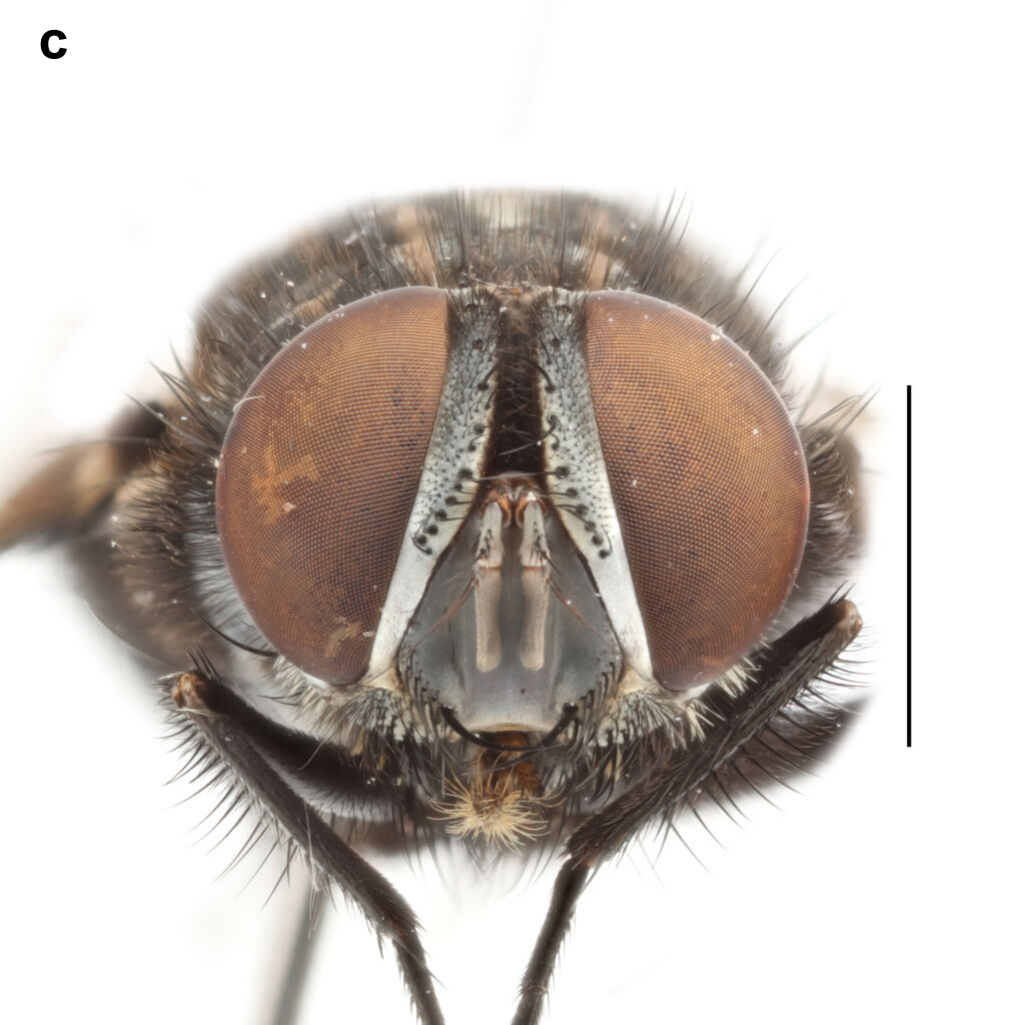
male head in anterior view.

**Figure 6d.**
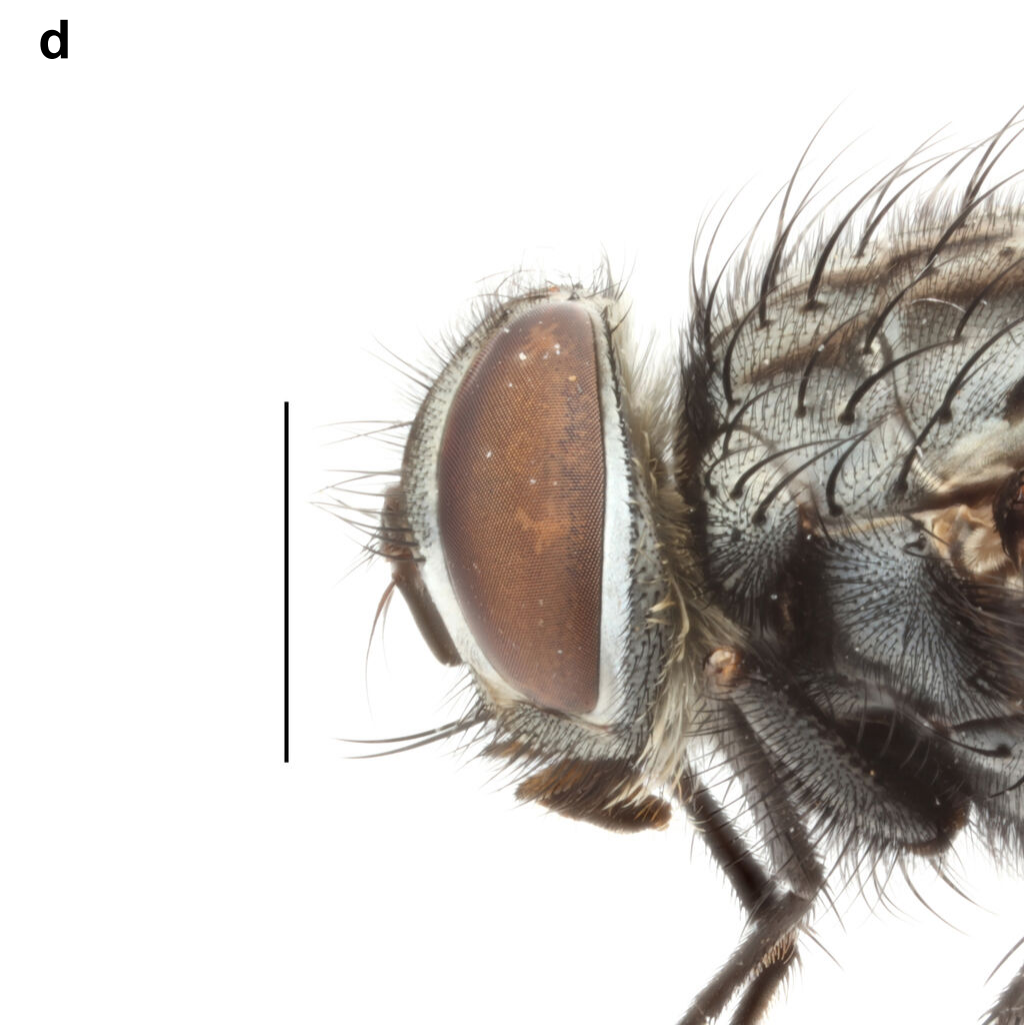
male head in lateral view.

**Figure 7a.**
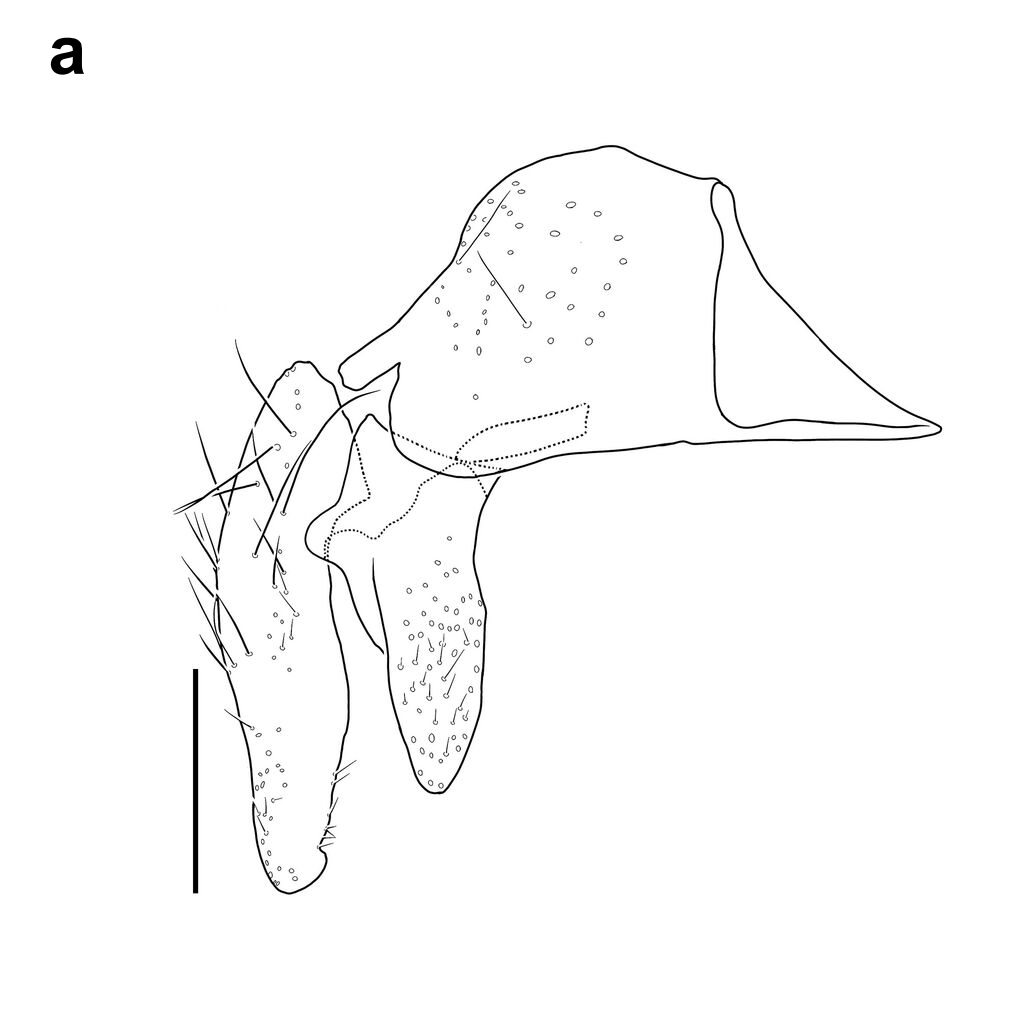
male cercus, surstylus and epandrium in lateral view.

**Figure 7b.**
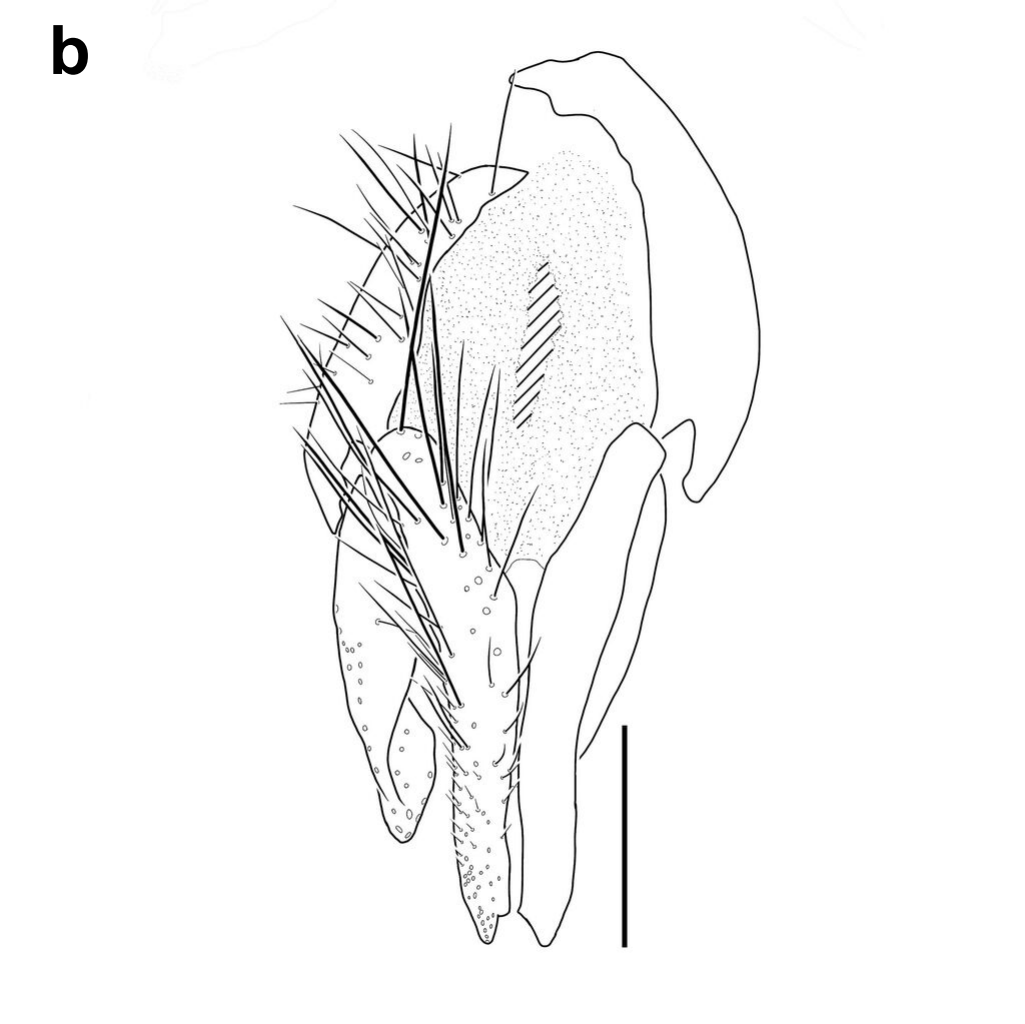
male cerci, surstyli and epandrium in caudal view.

**Figure 7c.**
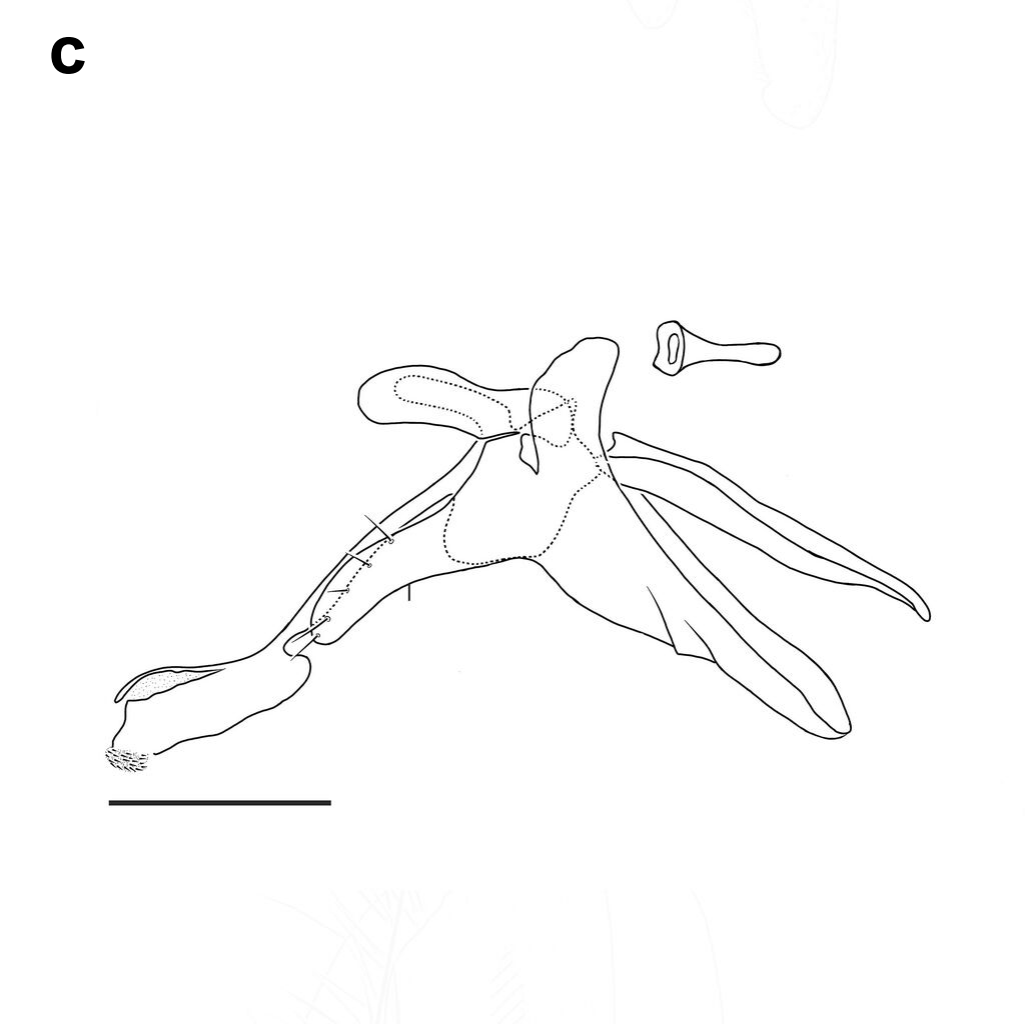
male phallus (basiphallus, distiphallus, ejaculatory apodeme, epiphallus, hypandrium, phallapodeme, postgonite, pregonite) in lateral view.

**Figure 7d.**
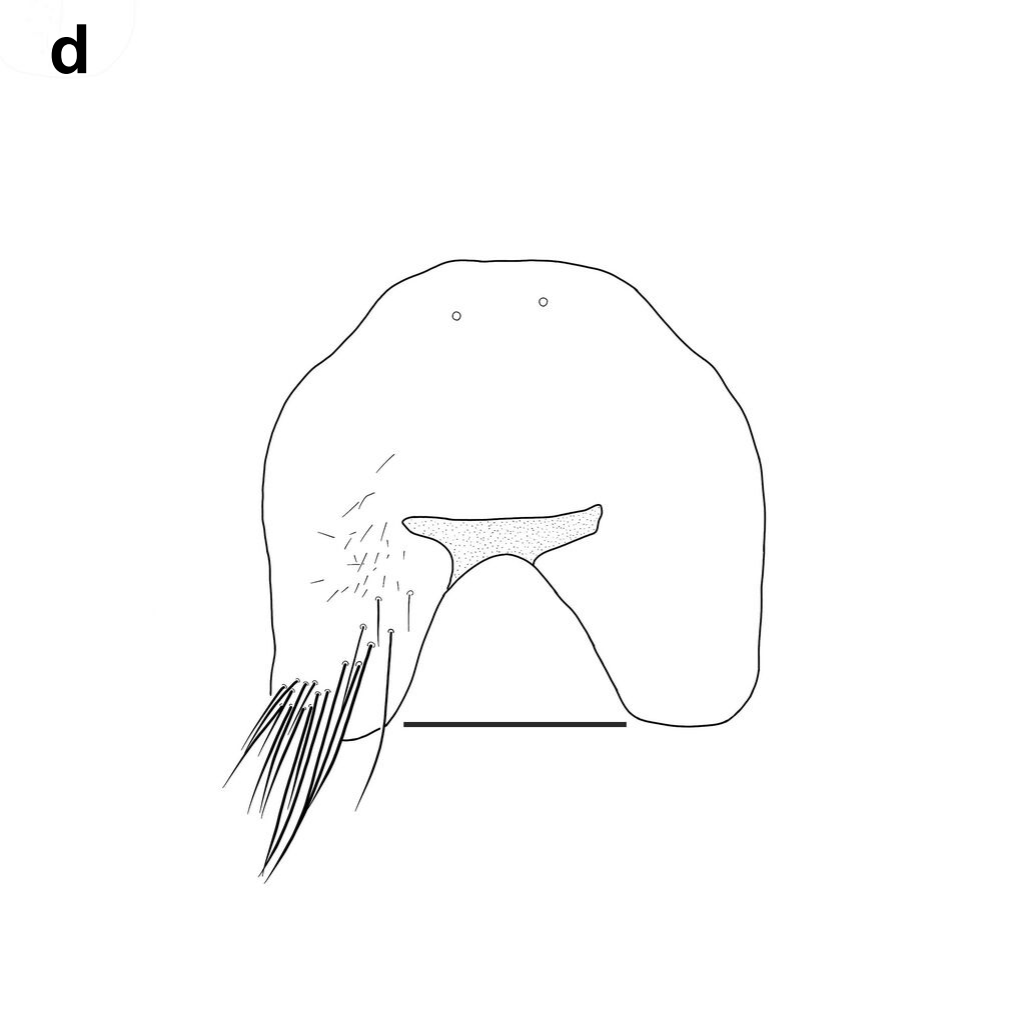
male sternite 5 in ventral view.

**Figure 8a.**
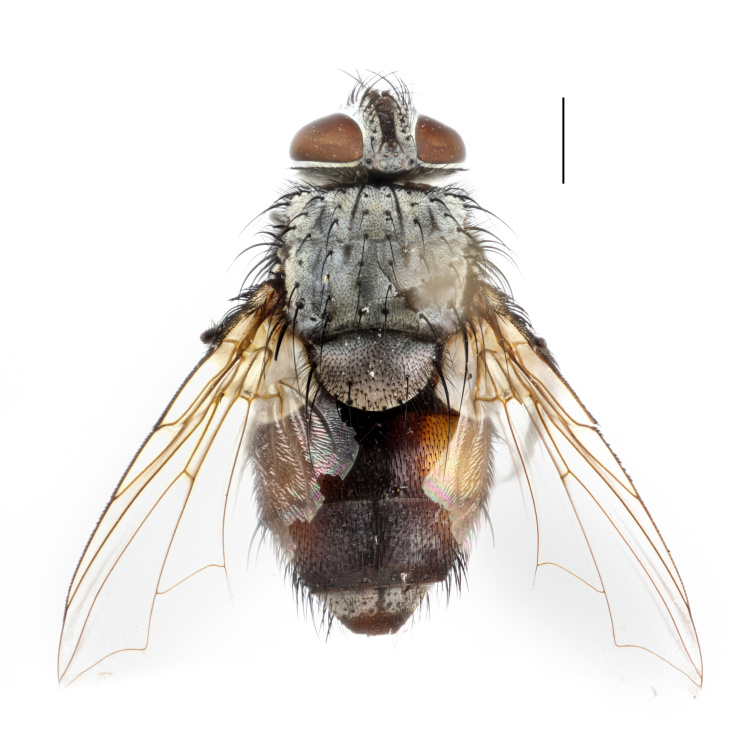
male body in dorsal view.

**Figure 8b.**
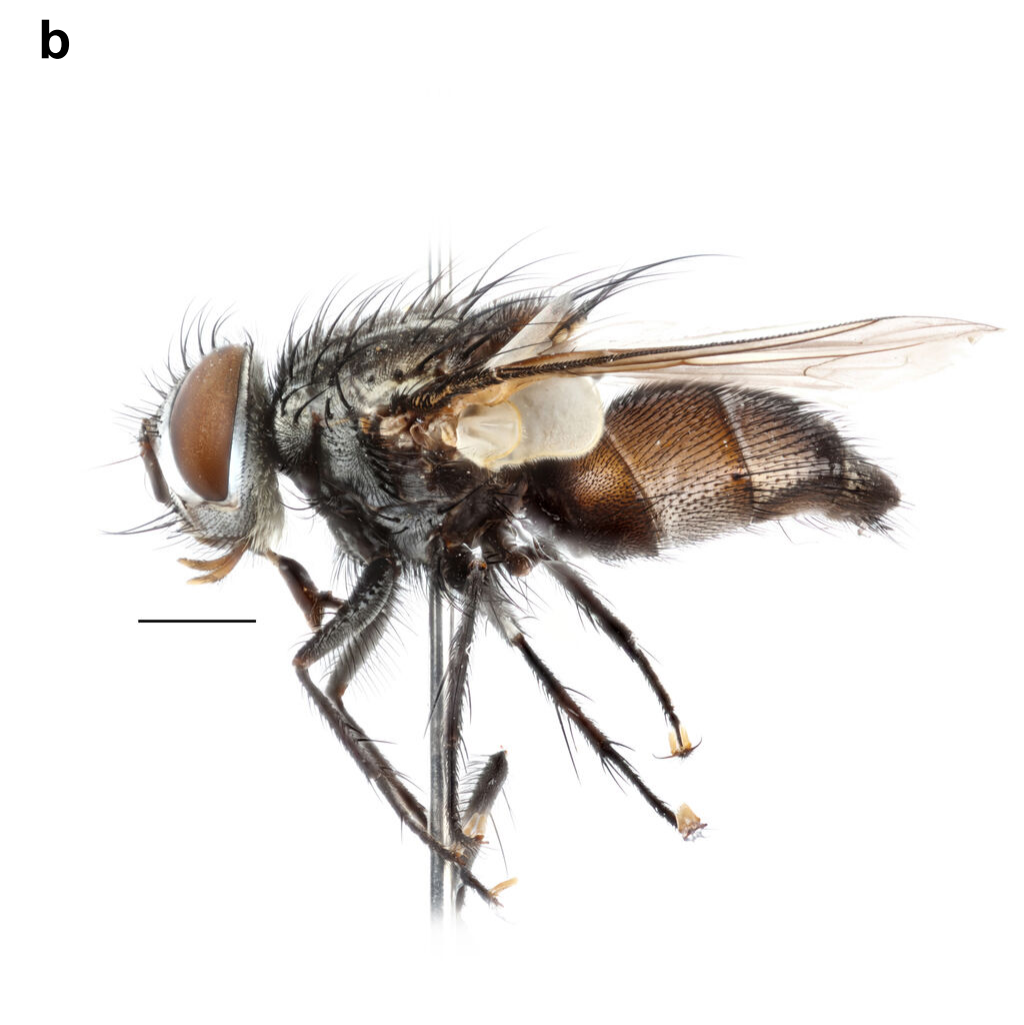
male body in lateral view.

**Figure 8c.**
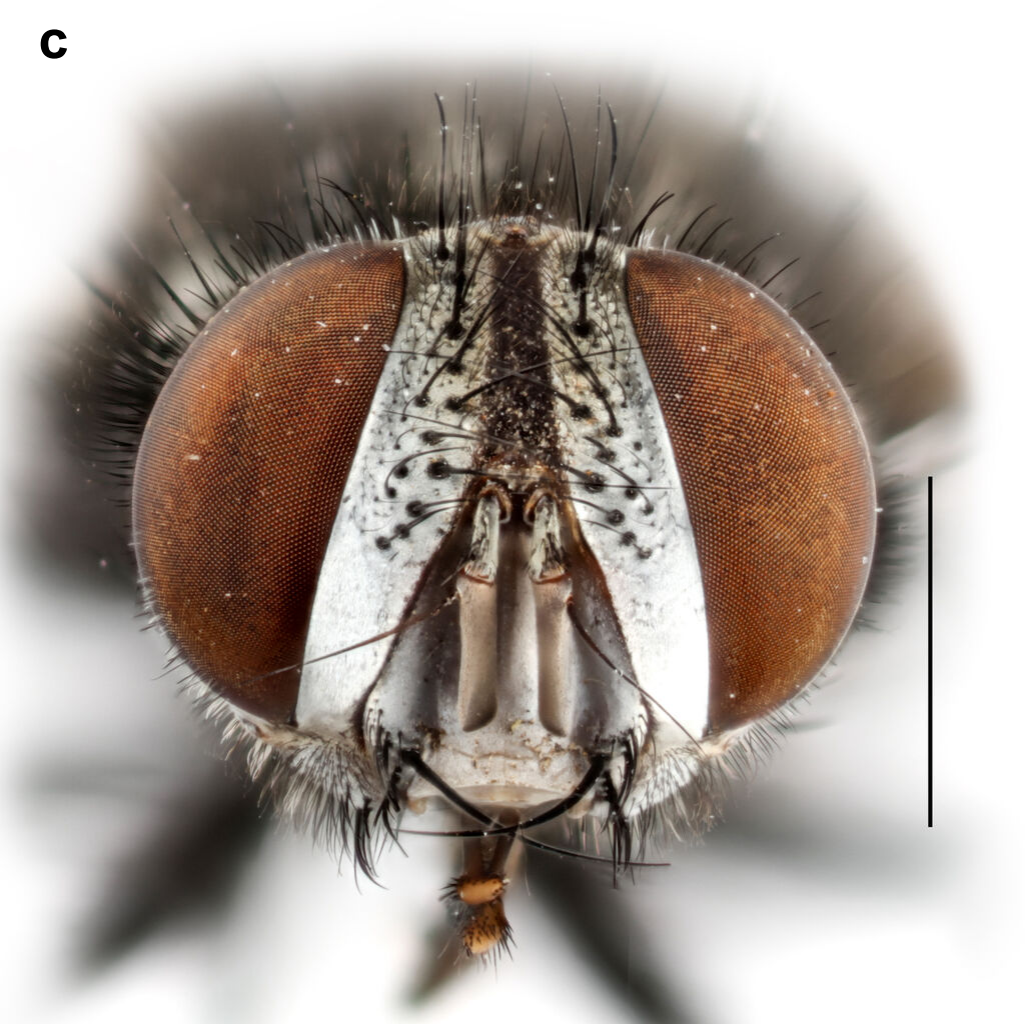
male head in anterior view.

**Figure 8d.**
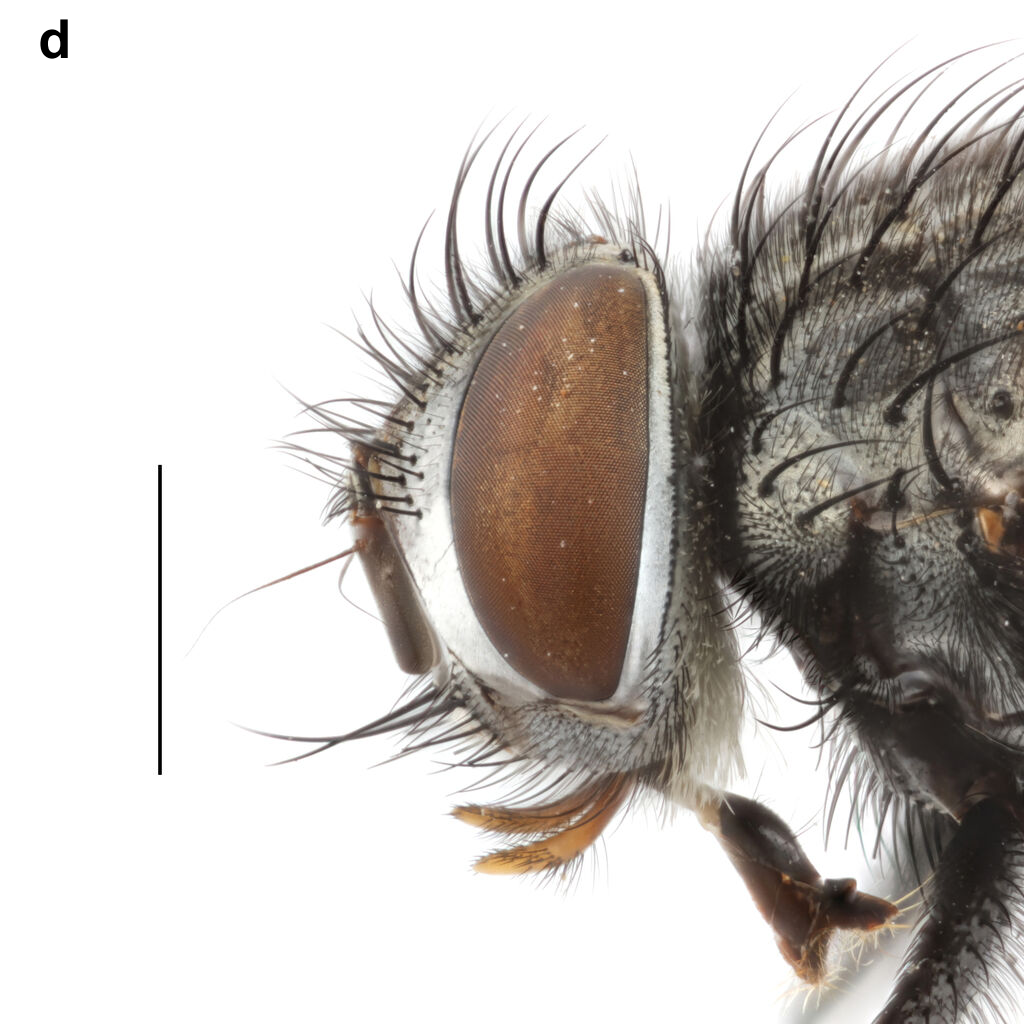
male head in lateral view.

**Figure 9a.**
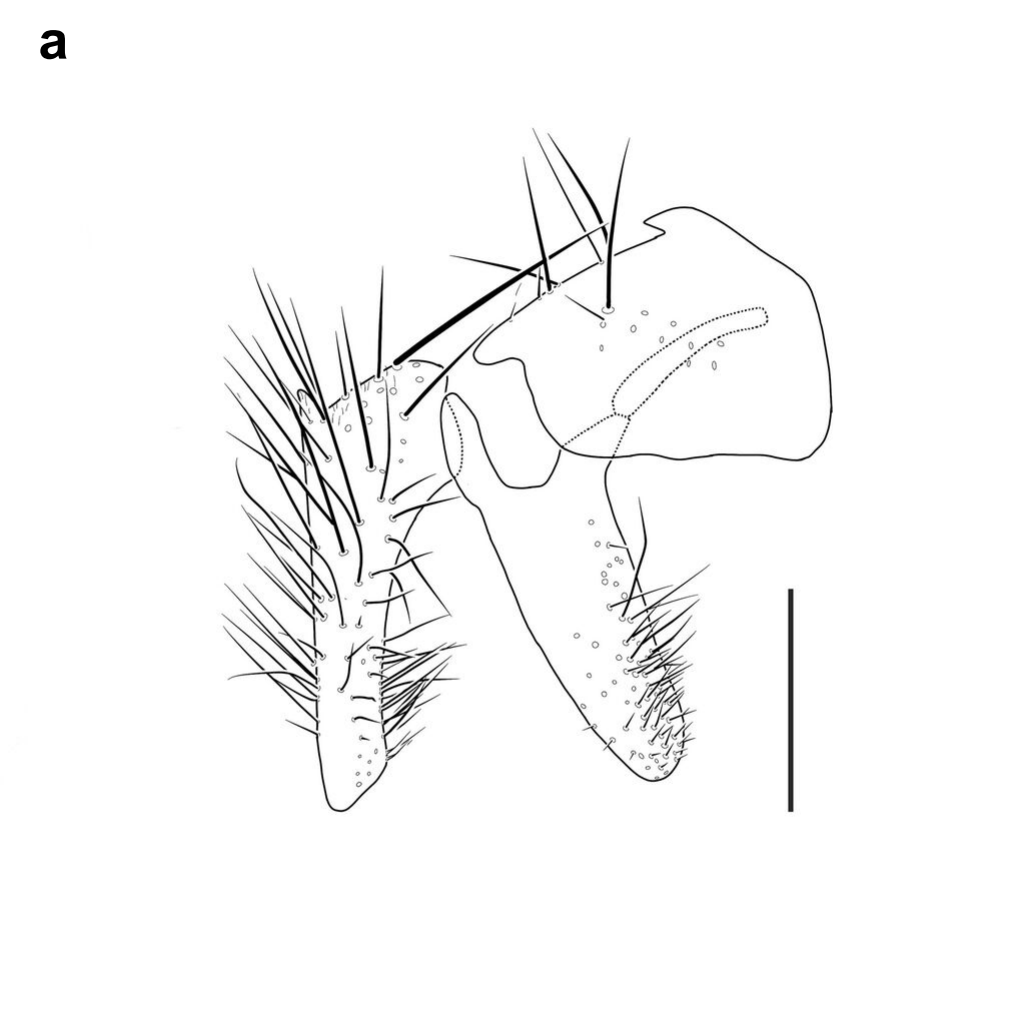


**Figure 9b.**
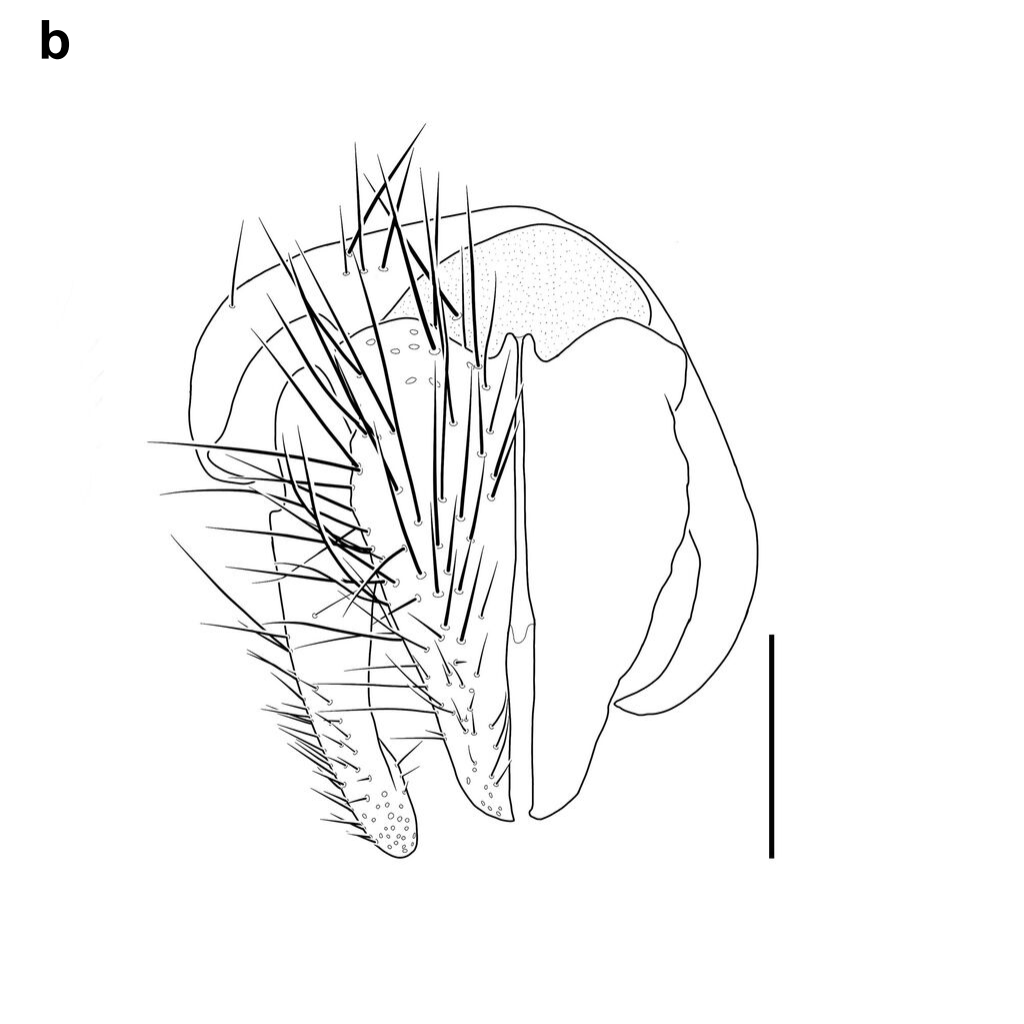
male cerci, surstyli and epandrium in caudal view.

**Figure 9c.**
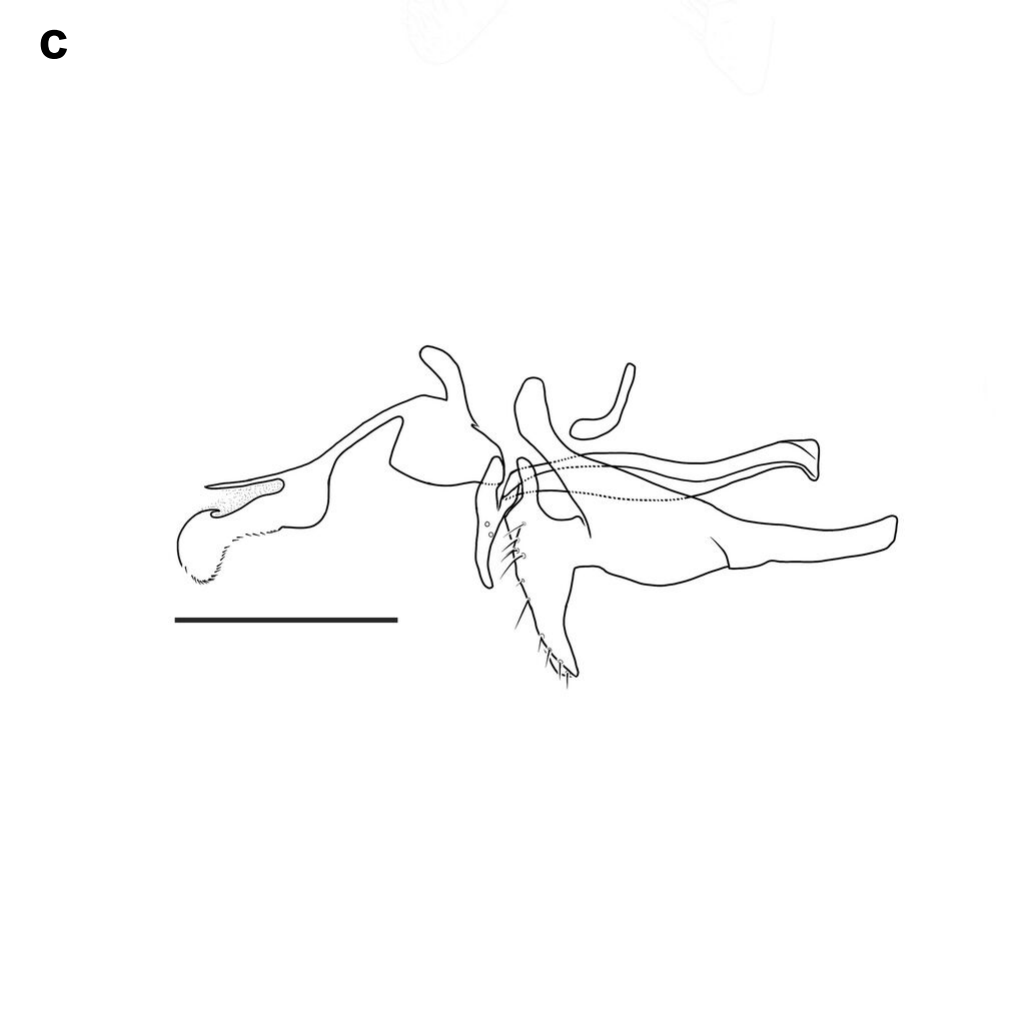
male phallus (basiphallus, distiphallus, ejaculatory apodeme, epiphallus, hypandrium, phallapodeme, postgonite, pregonite) in lateral view.

**Figure 9d.**
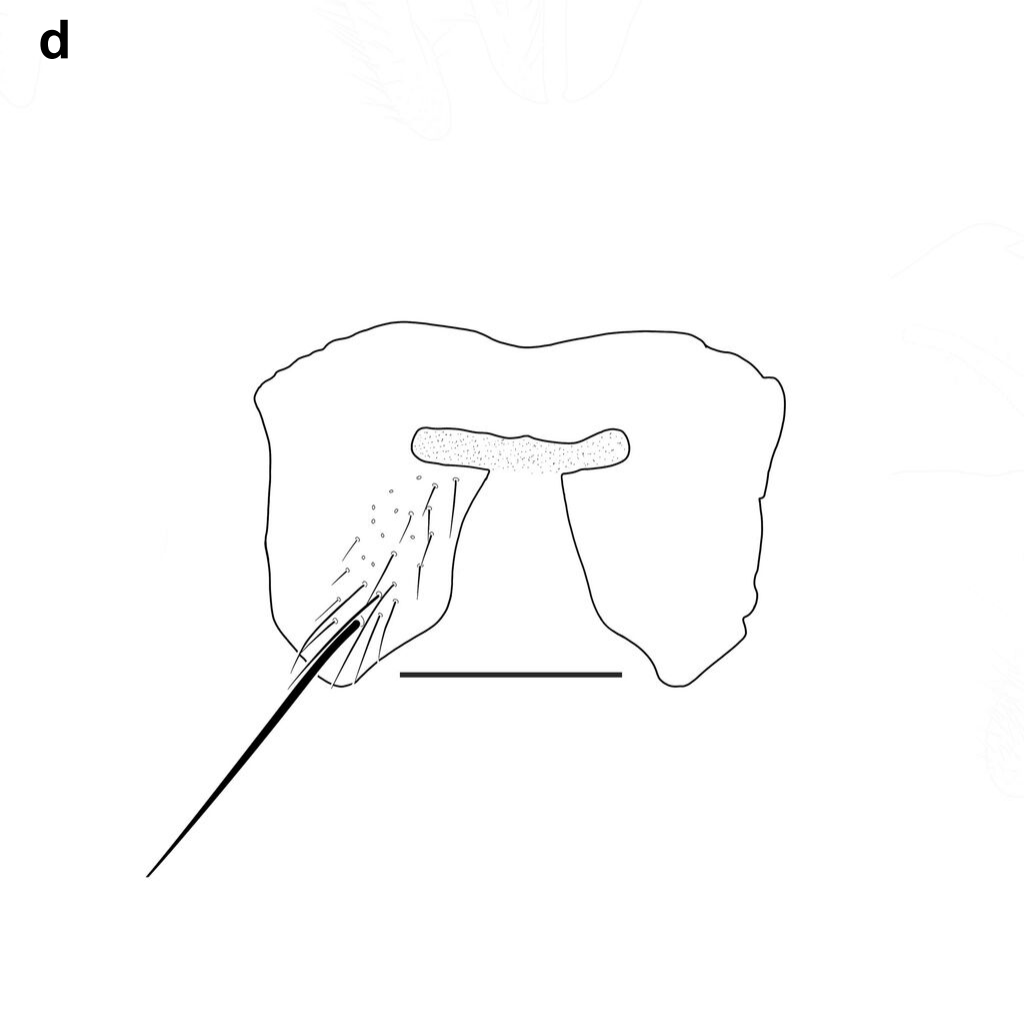
male sternite 5 in ventral view.

**Figure 10a.**
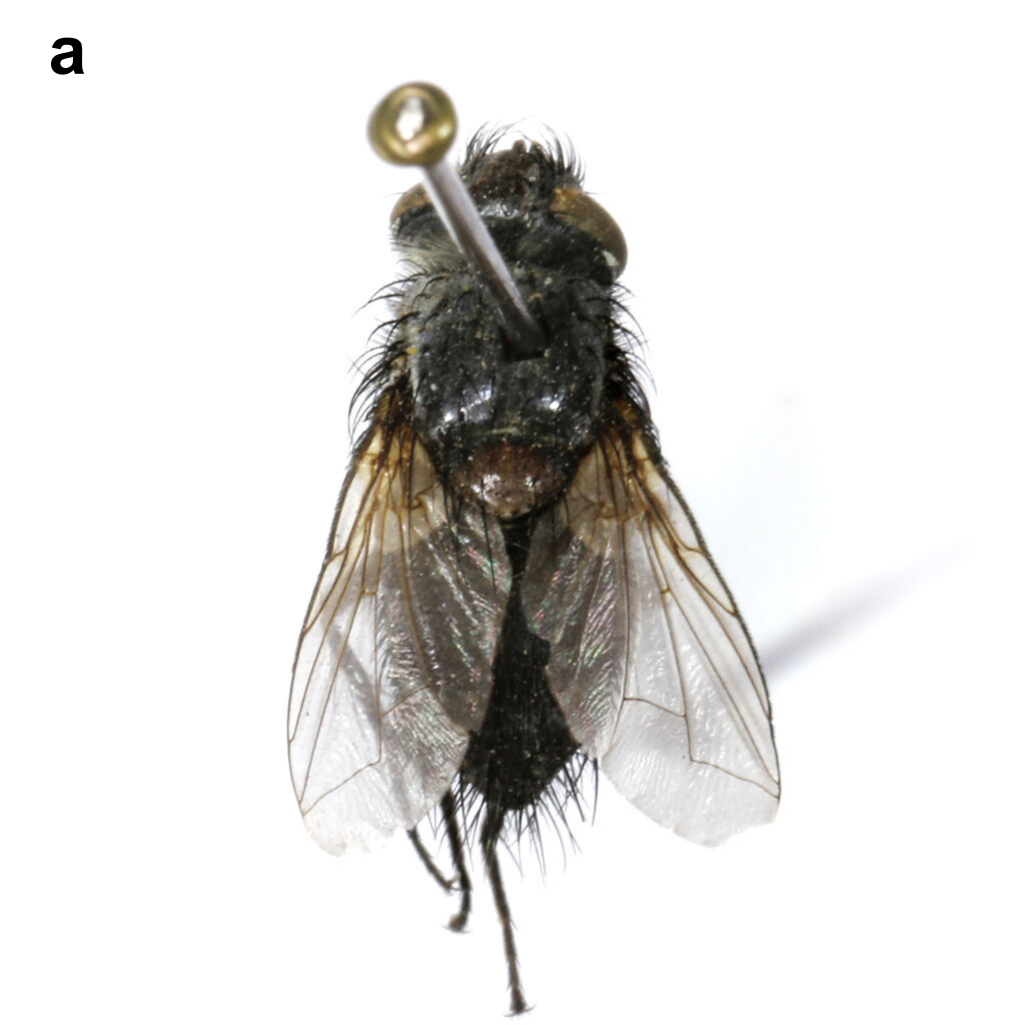
male body in dorsal view

**Figure 10b.**
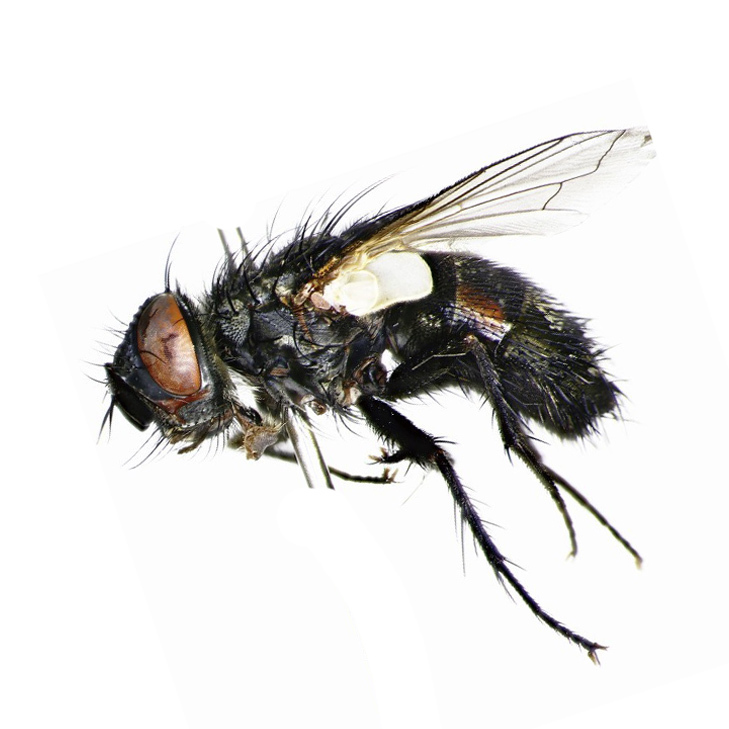
male body in lateral view

**Figure 10c.**
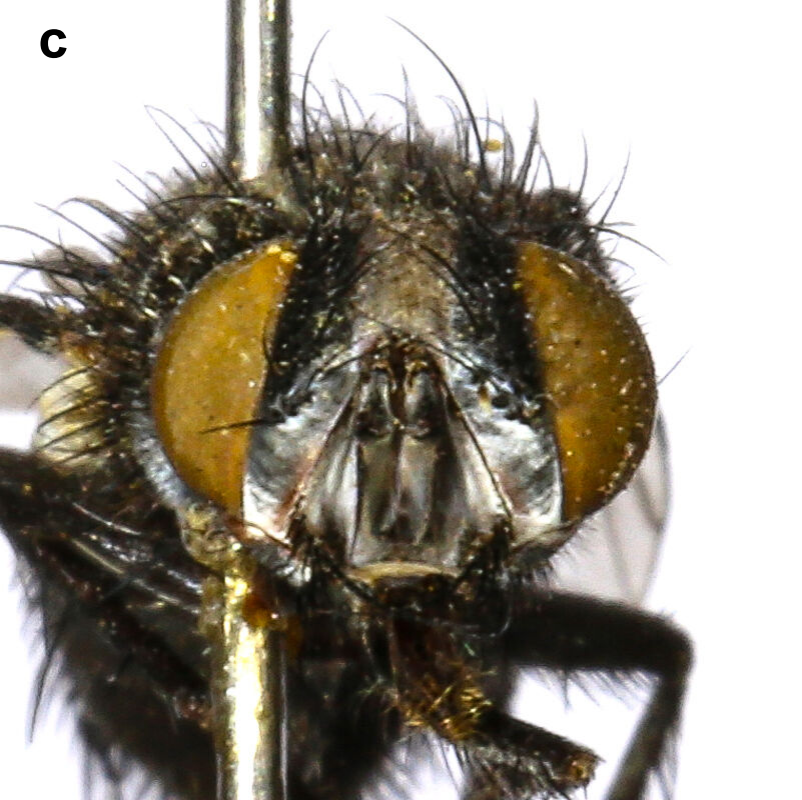
male head in anterior view

**Figure 10d.**
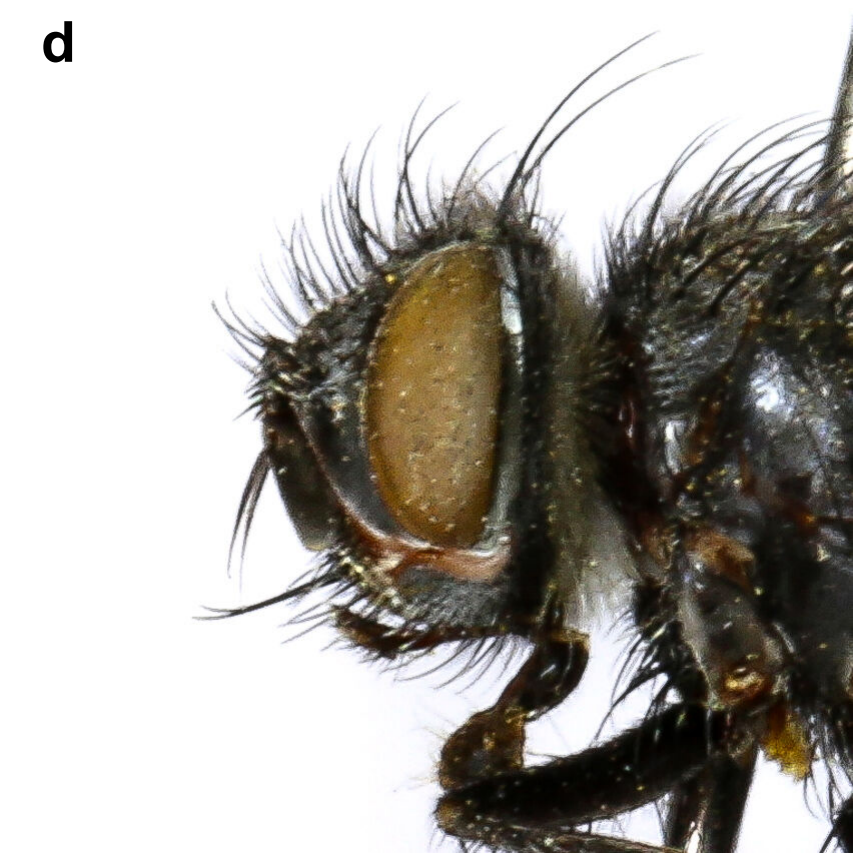
male head in latera view

**Figure 11a.**
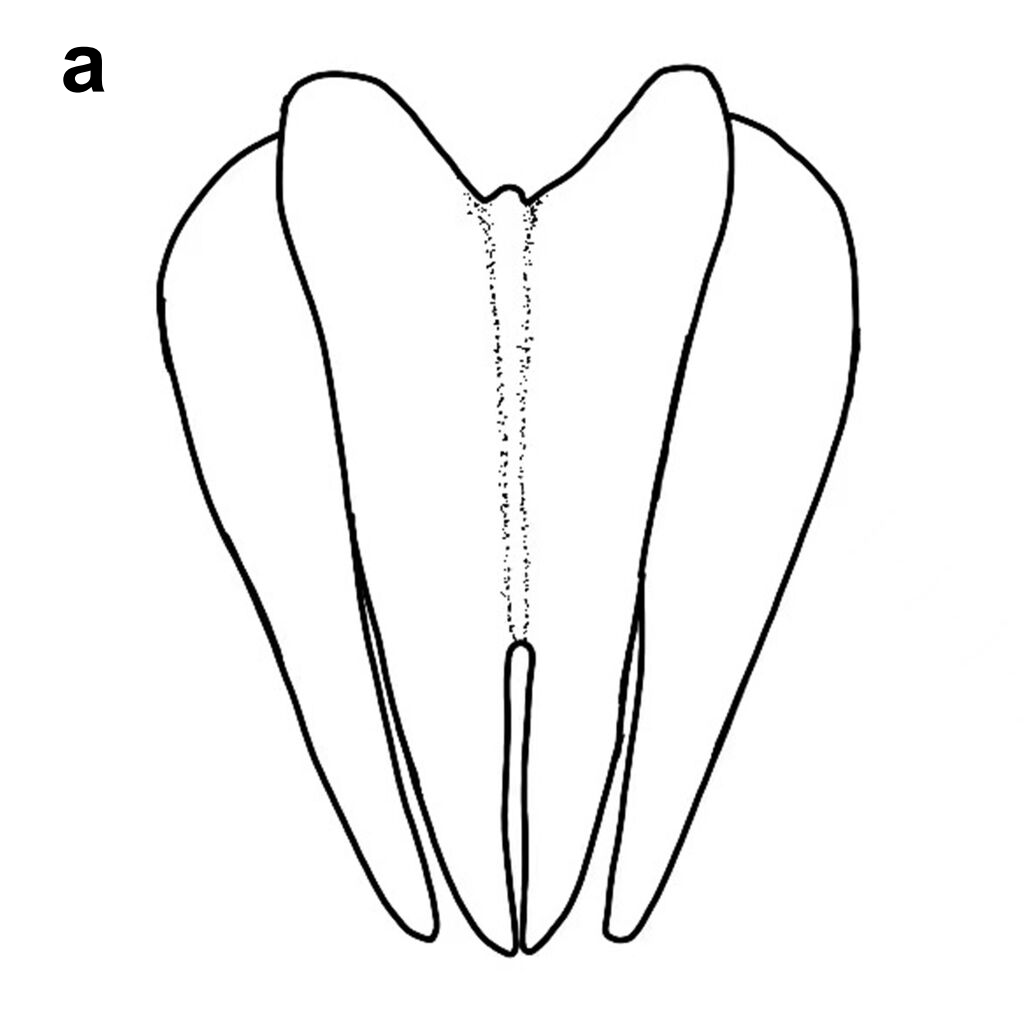
male cerci, surstyli in caudal view.

**Figure 11b.**
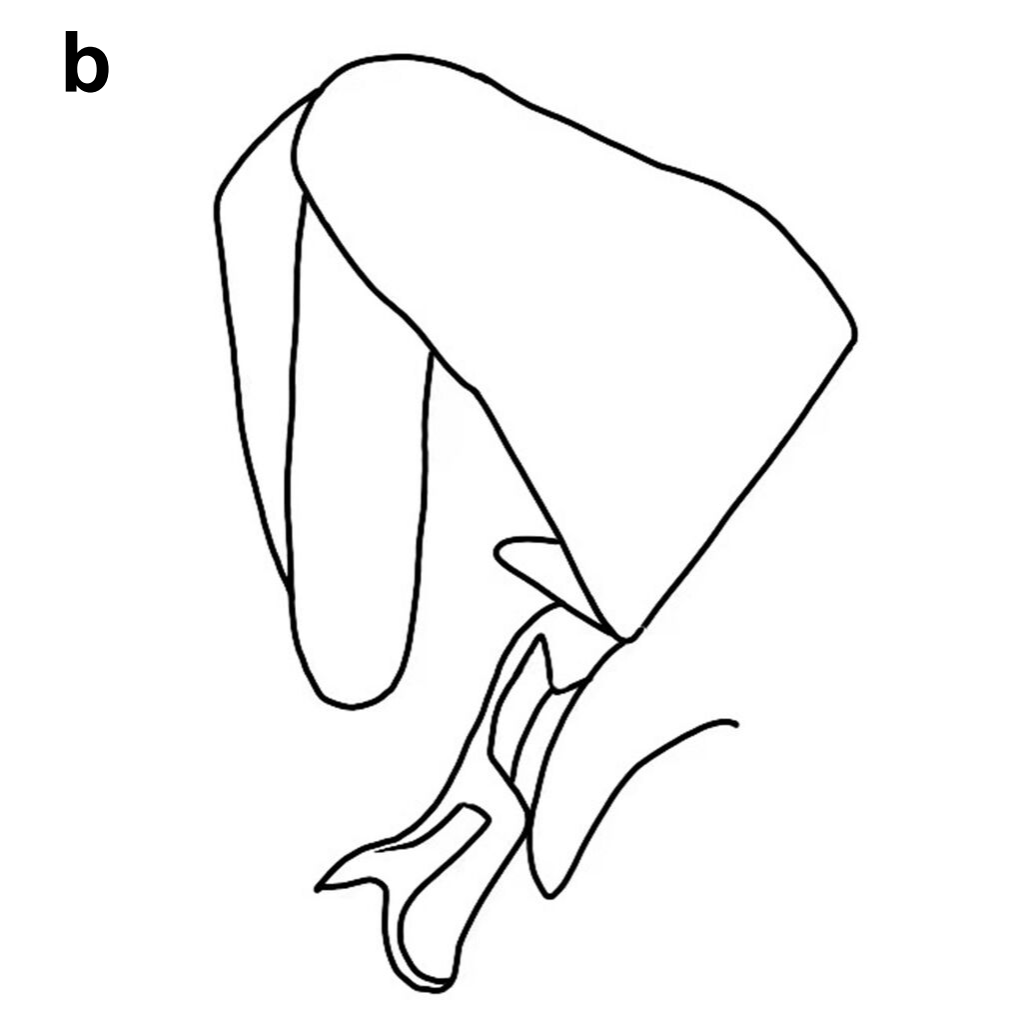
male cercus, surstylus, epandrium and phallus (part) in lateral view.

**Figure 12a.**
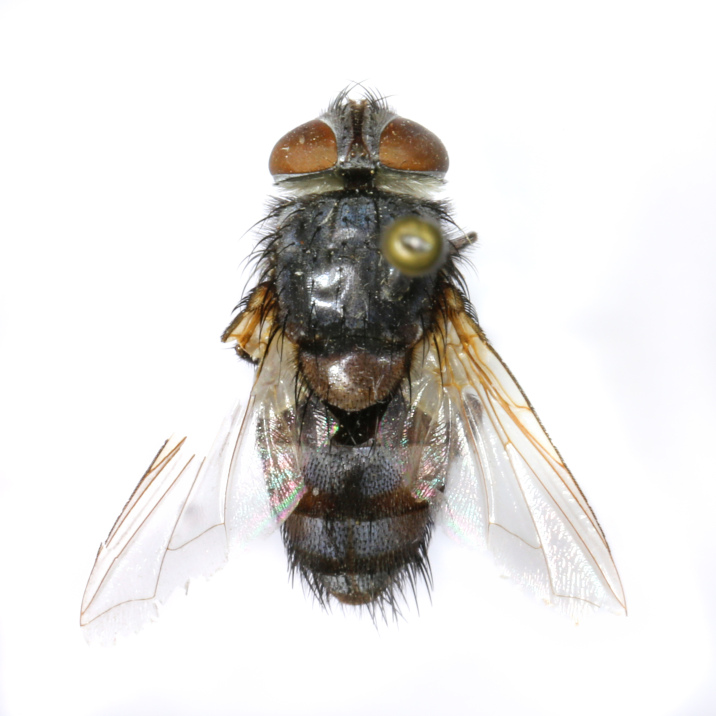
male body in dorsal view

**Figure 12b.**
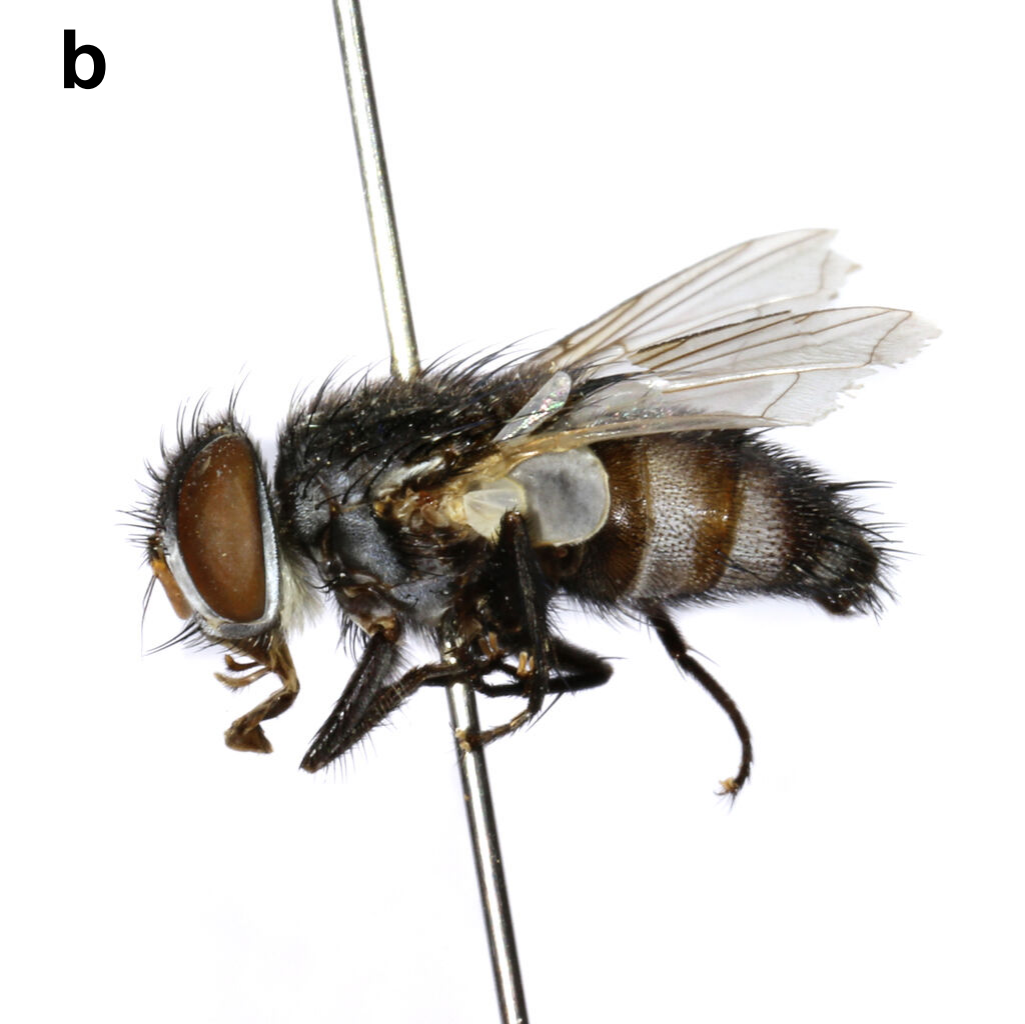
male body in lateral view

**Figure 12c.**
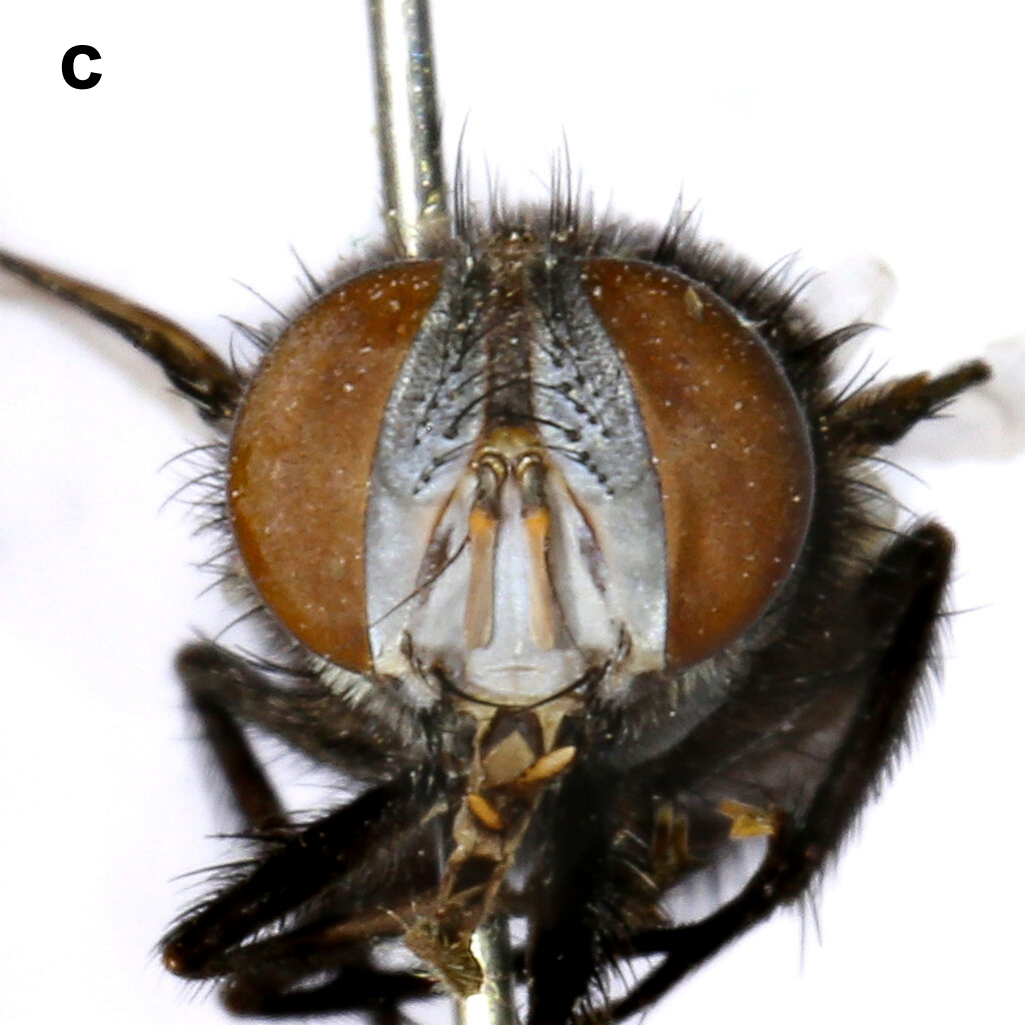
male head in anterior view

**Figure 12d.**
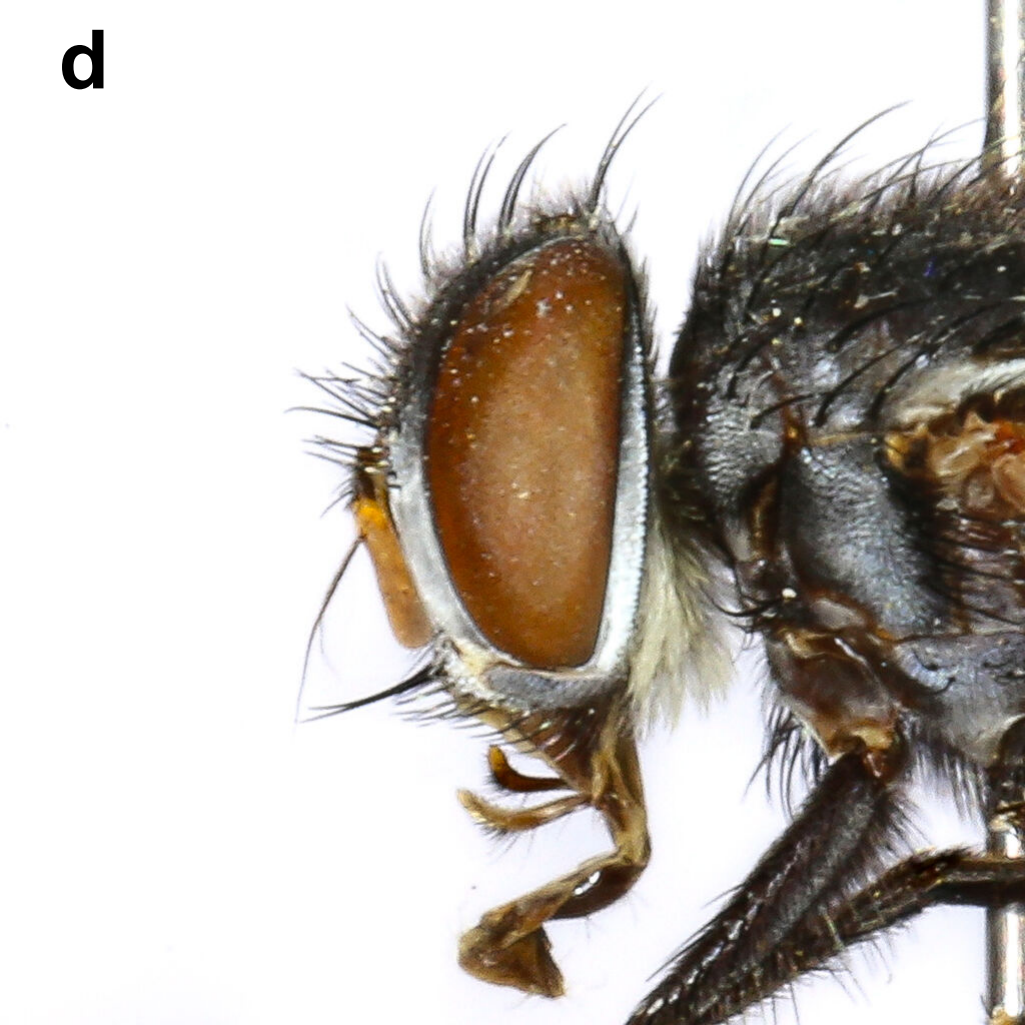
male heas in lateral view

**Figure 13a.**
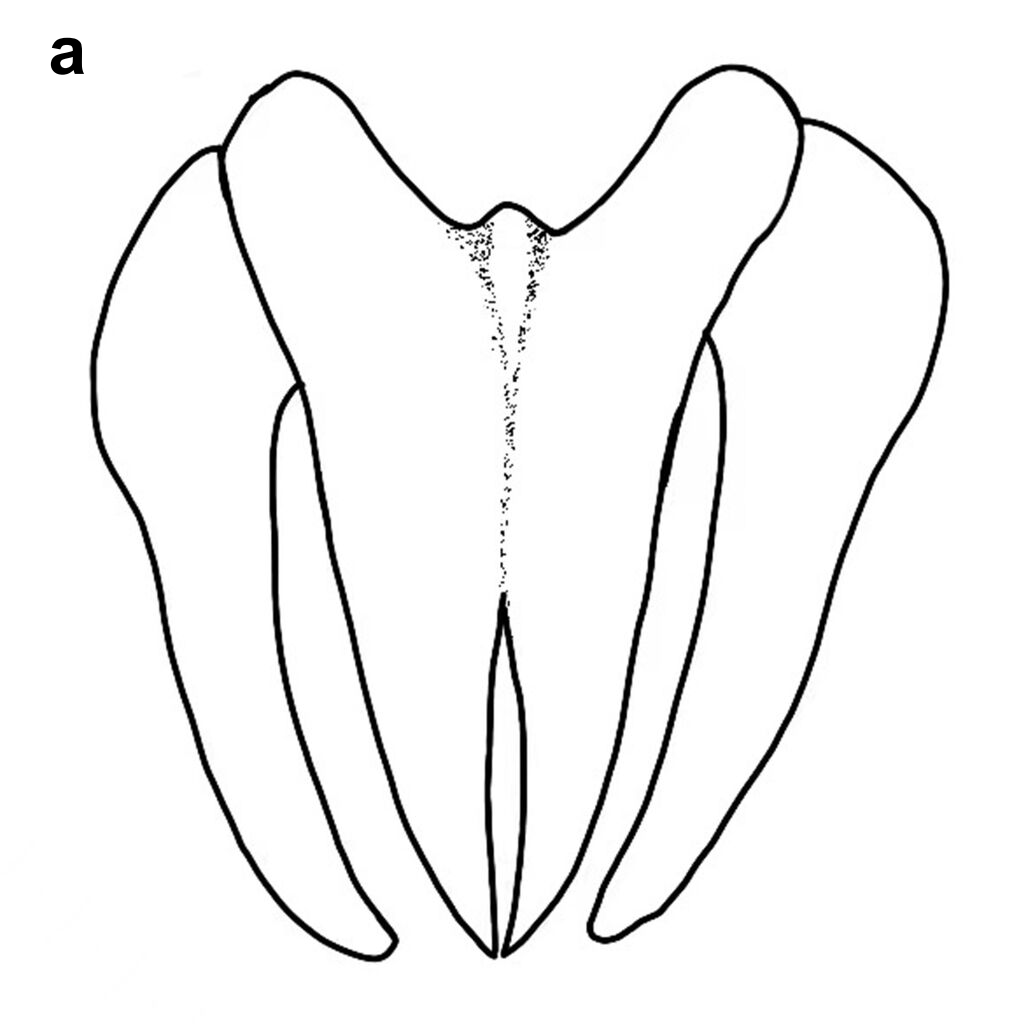
male cerci, surstyli in caudal view.

**Figure 13b.**
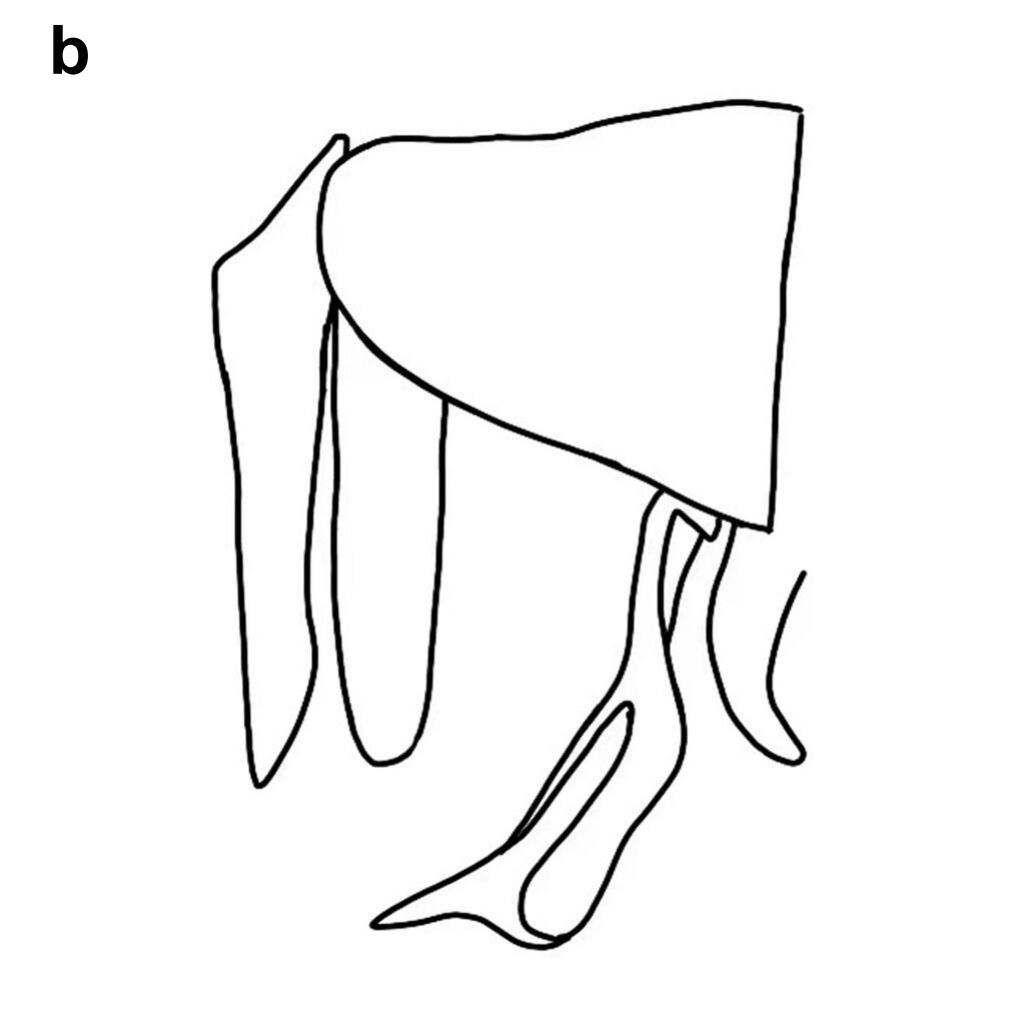
male cercus, surstylus, epandrium and phallus (part) in lateral view.

**Figure 14a.**
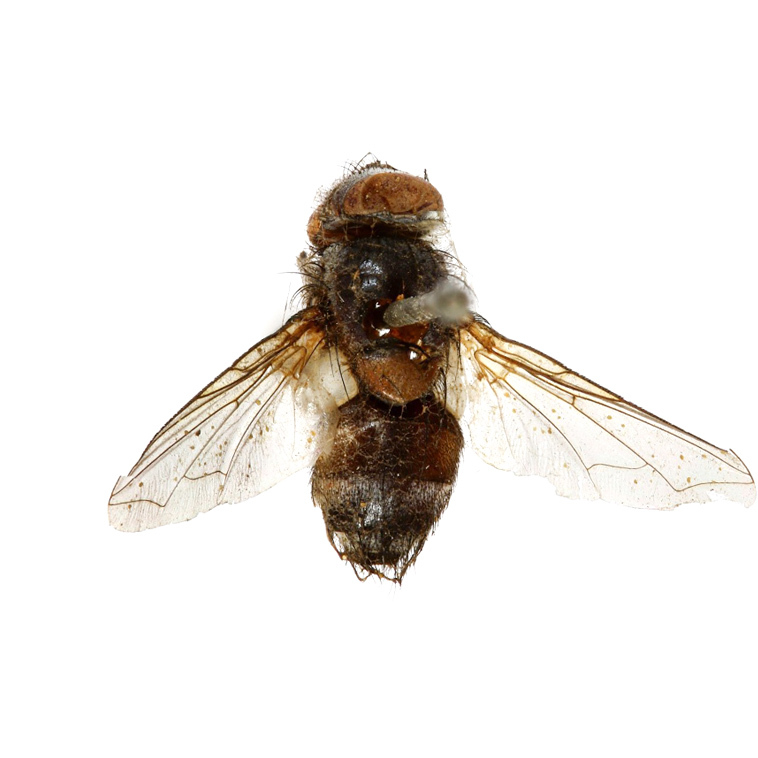
male body in dorsal view

**Figure 14b.**
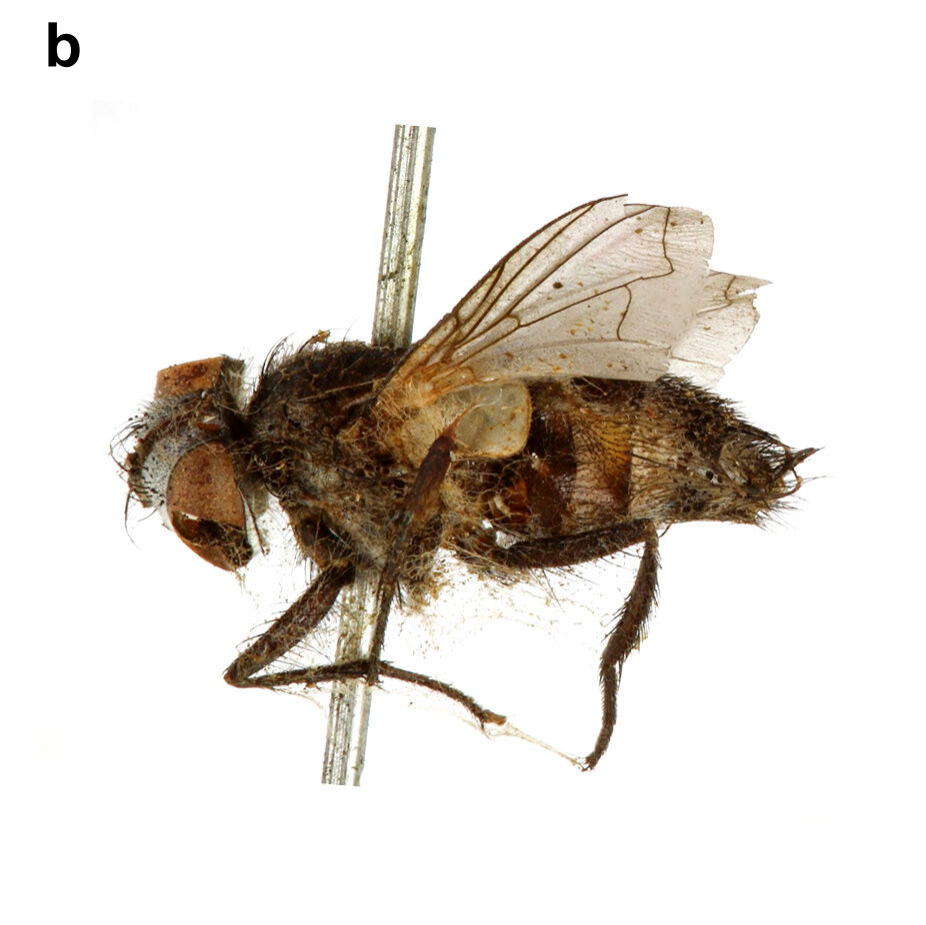
male body in lateral view

**Figure 14c.**
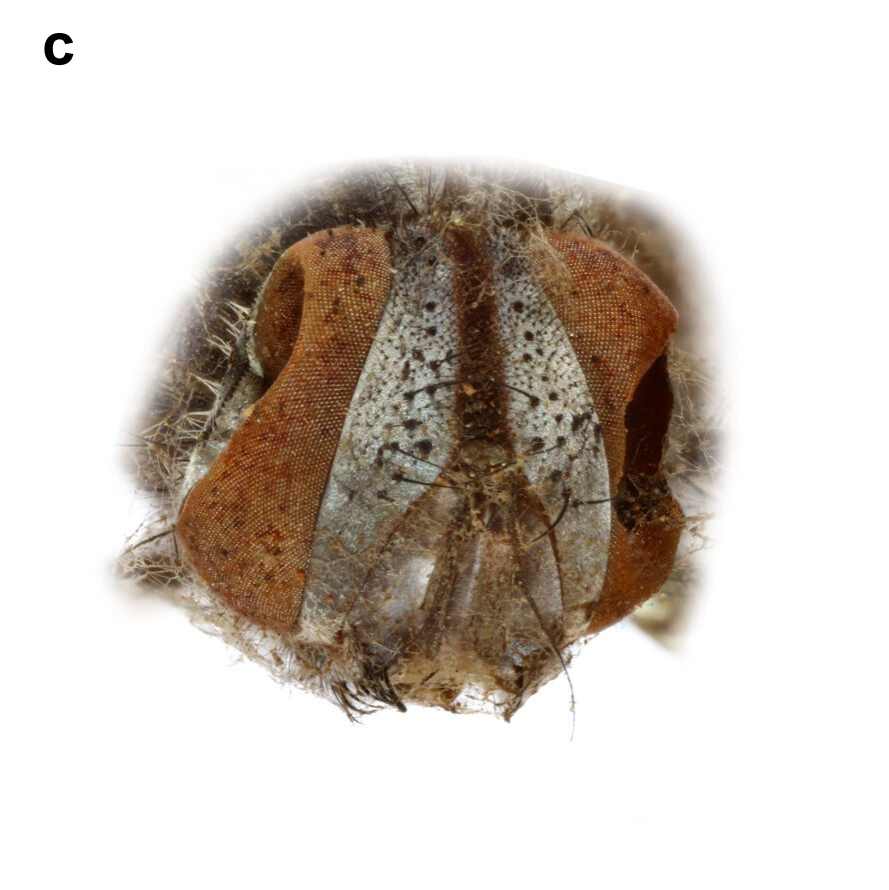
male head in anterior view

**Figure 14d.**
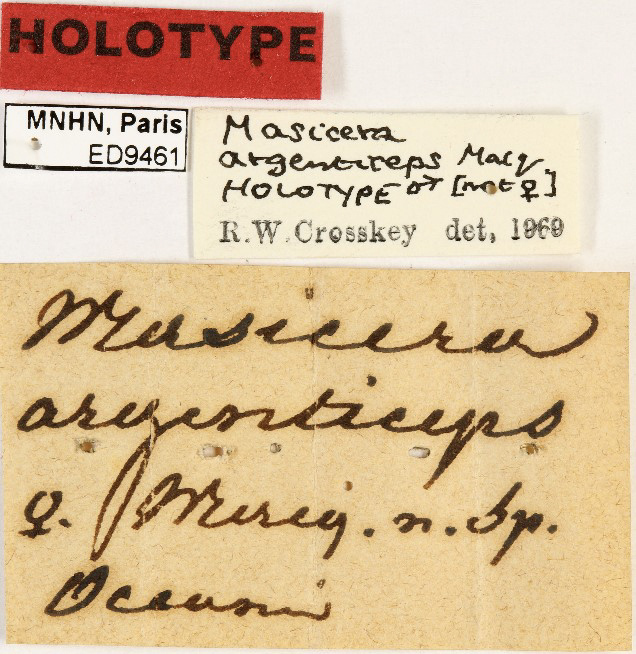
label information of *Masicera argenticeps* Macquart

**Figure 15a.**
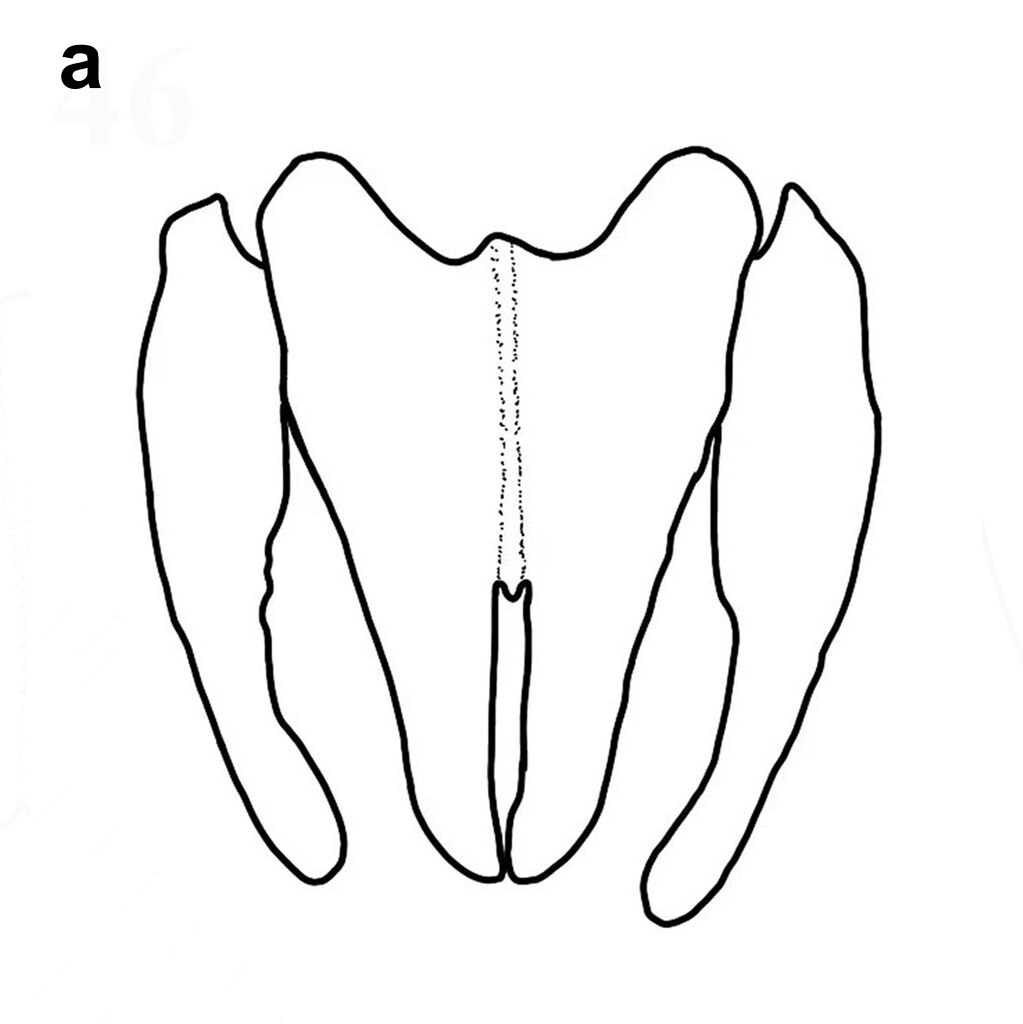
male cerci, surstyli in caudal view.

**Figure 15b.**
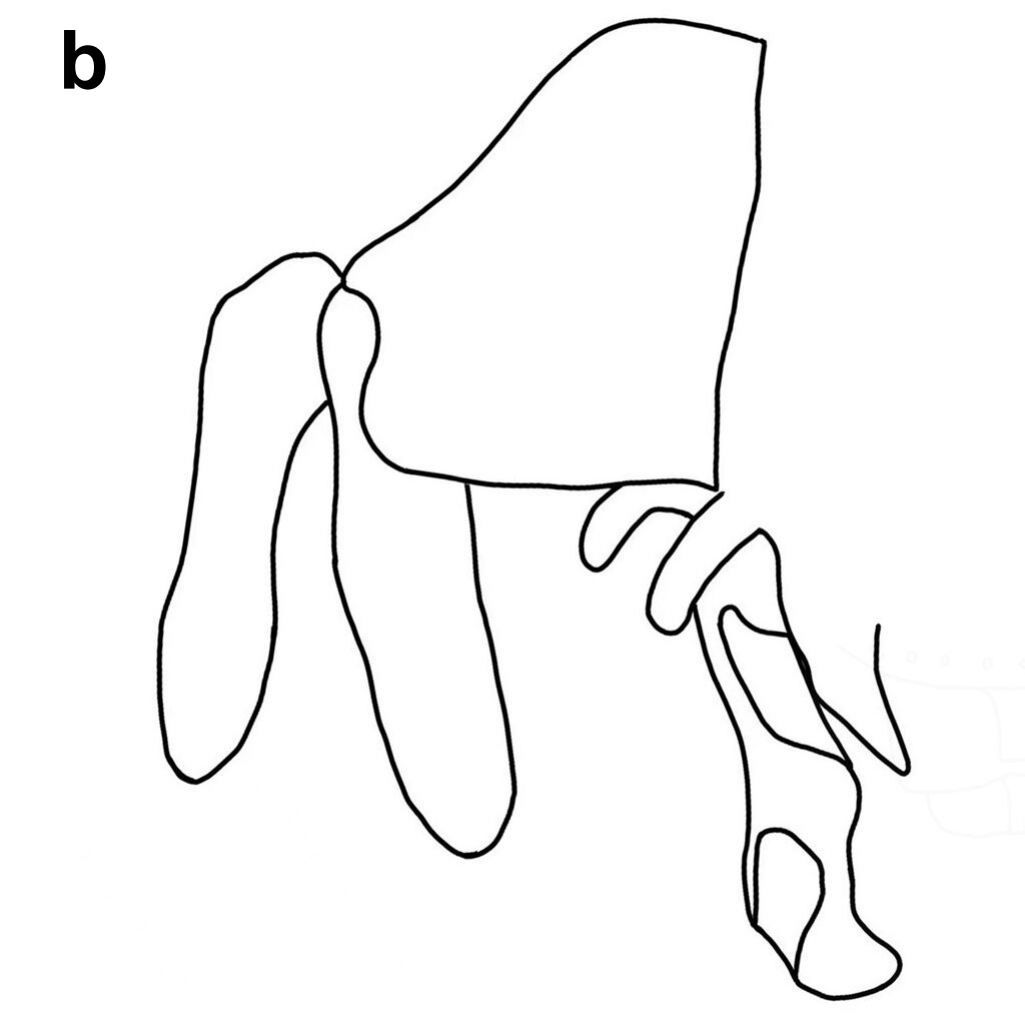
male cercus, surstylus, epandrium and phallus (part) in lateral view.

**Figure 16a.**
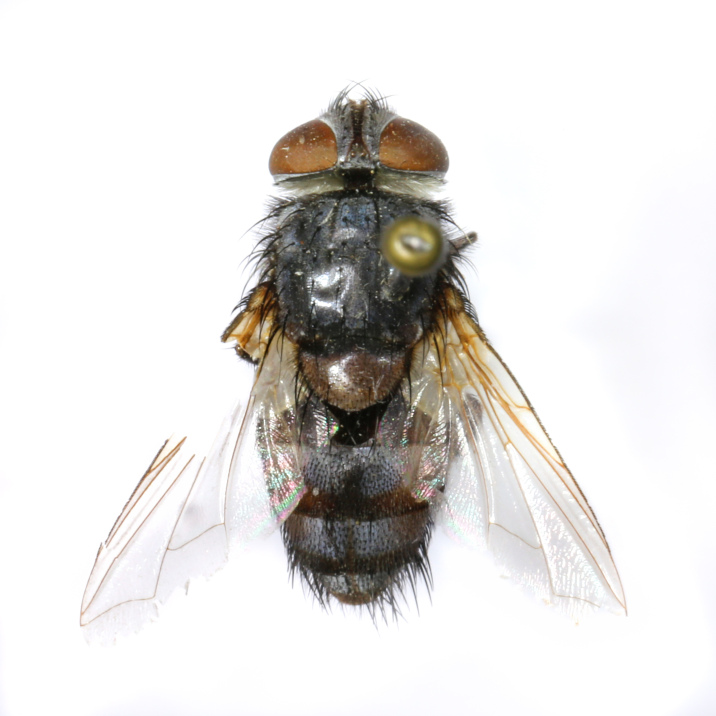
male body in dorsal view

**Figure 16b.**
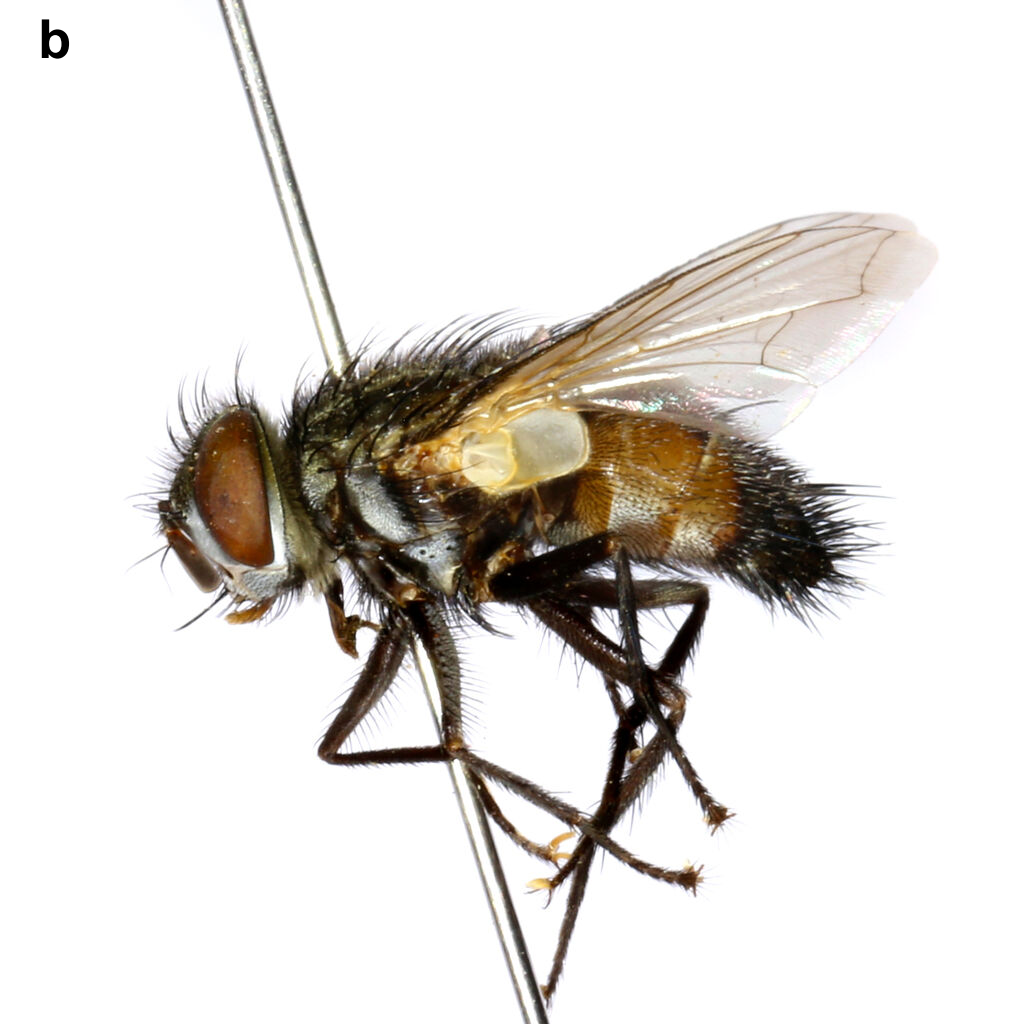
male body in lateral view

**Figure 16c.**
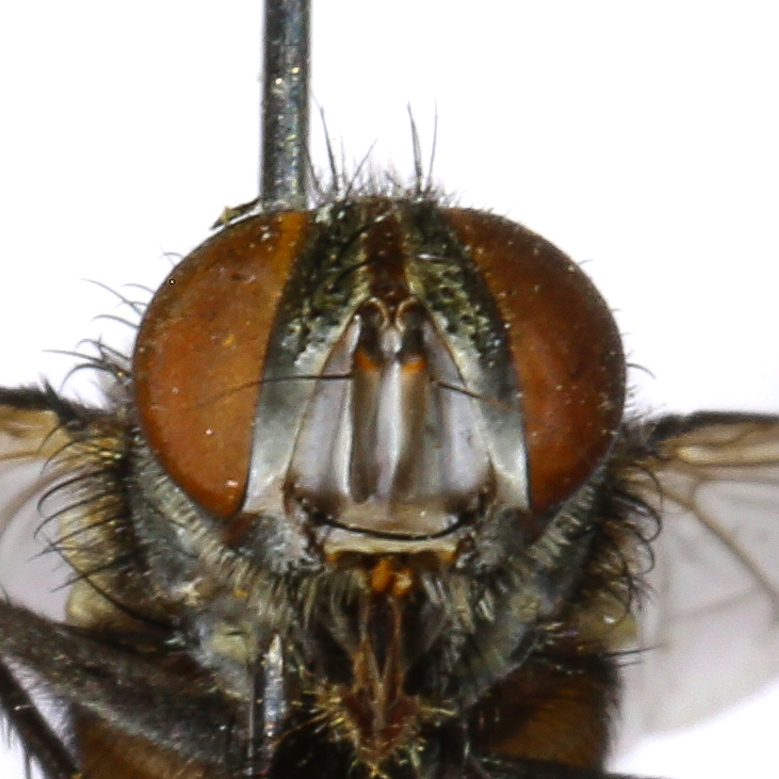
male head in anterior view

**Figure 16d.**
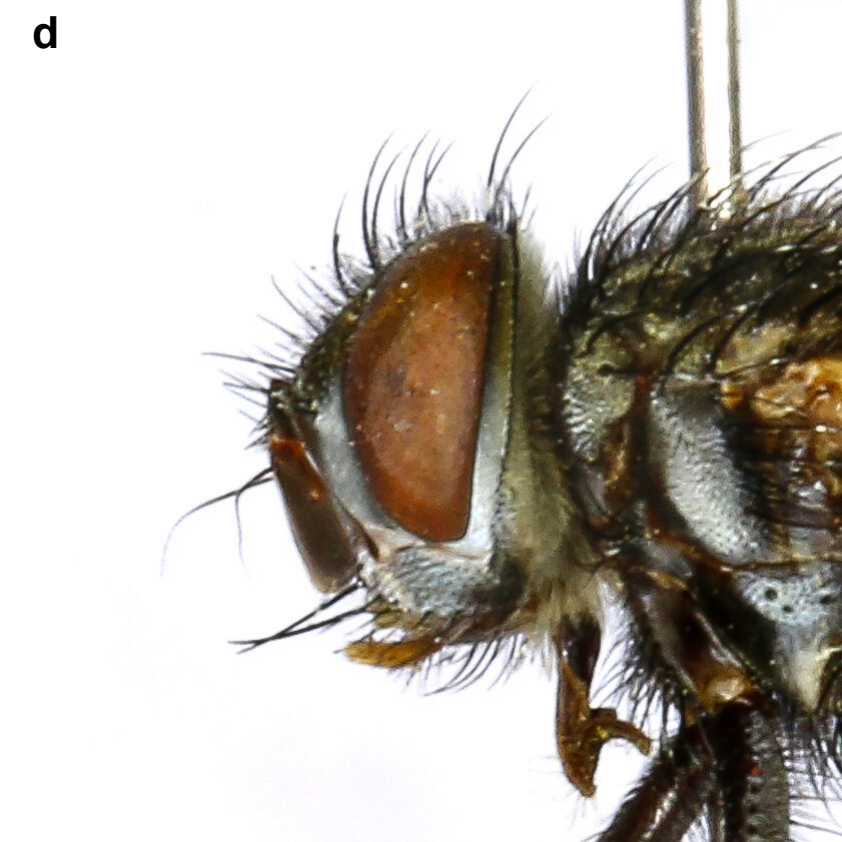
male head in lateral view

**Figure 17a.**
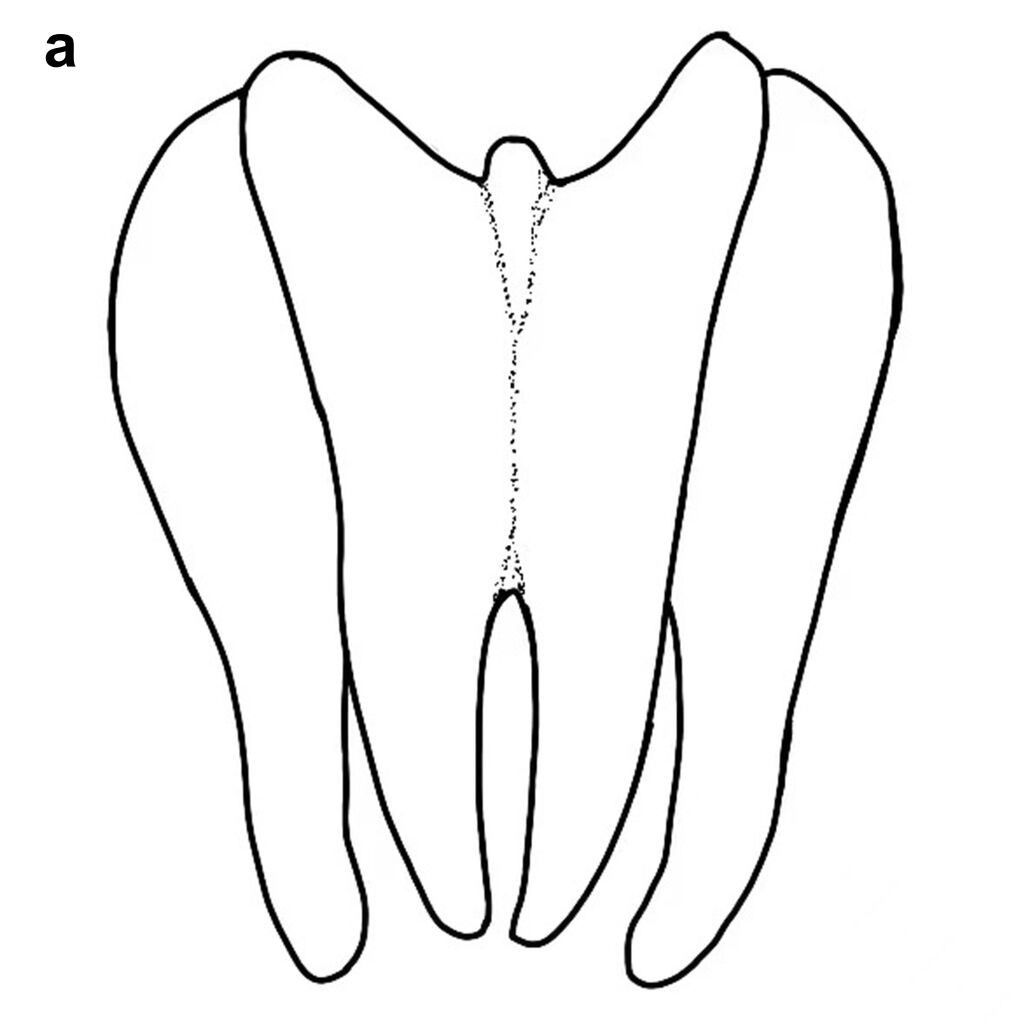
male cerci, surstyli in caudal view.

**Figure 17b.**
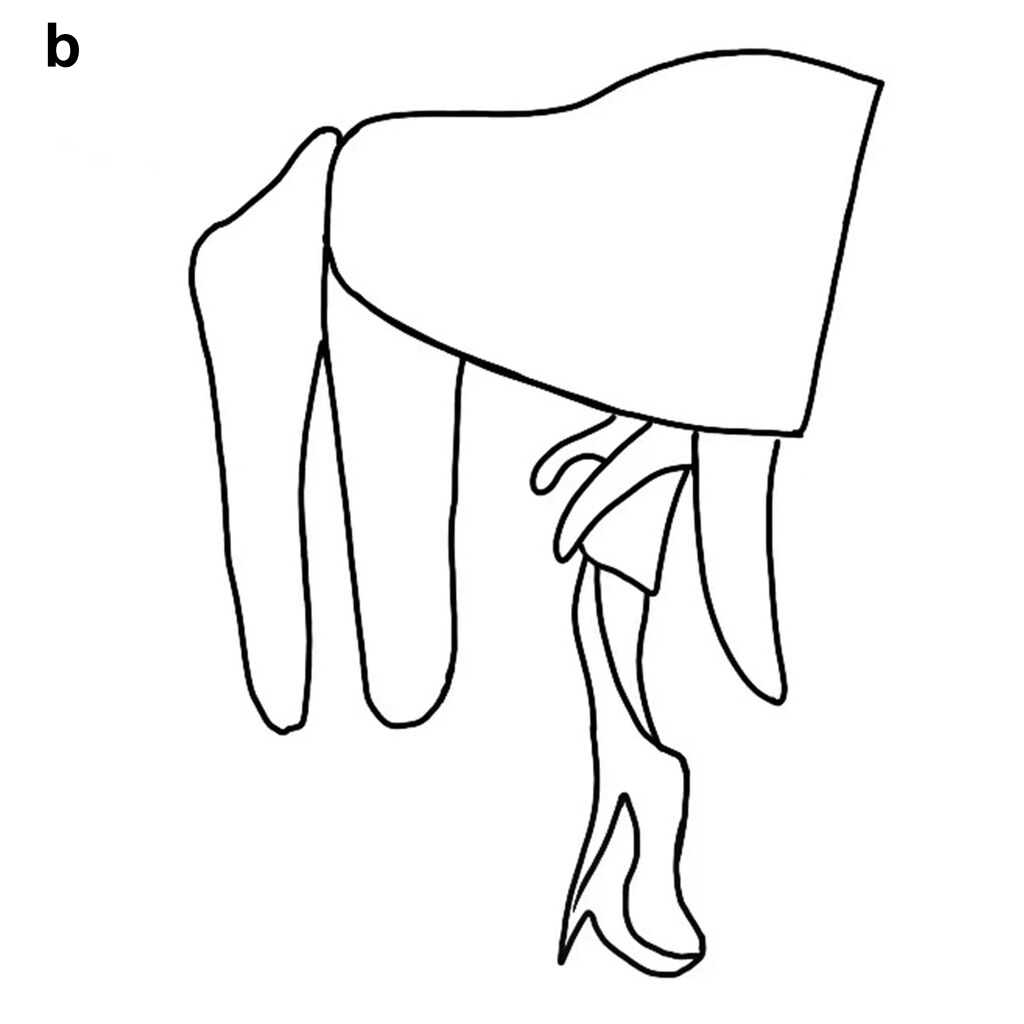
male cercus, surstylus, epandrium and phallus (part) in lateral view.

**Figure 18a.**
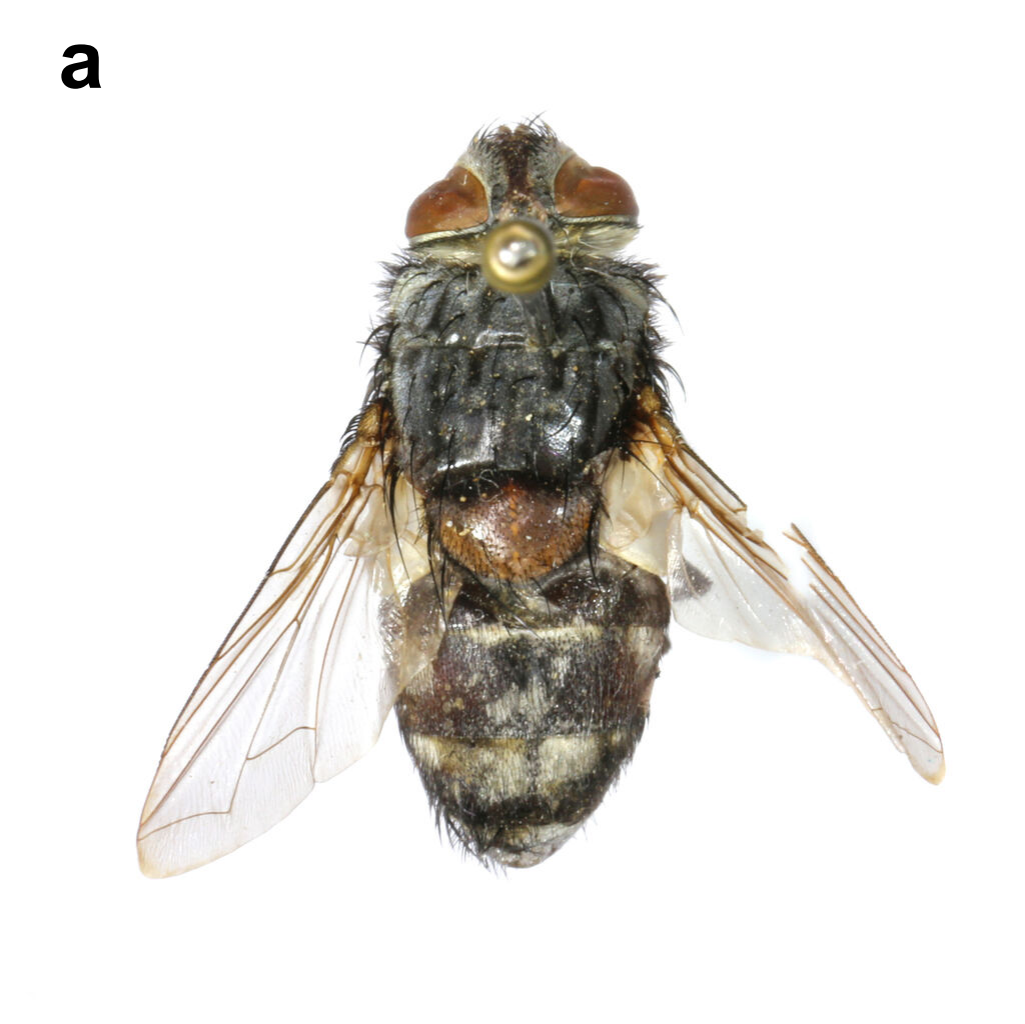
male body in dorsal view

**Figure 18b.**
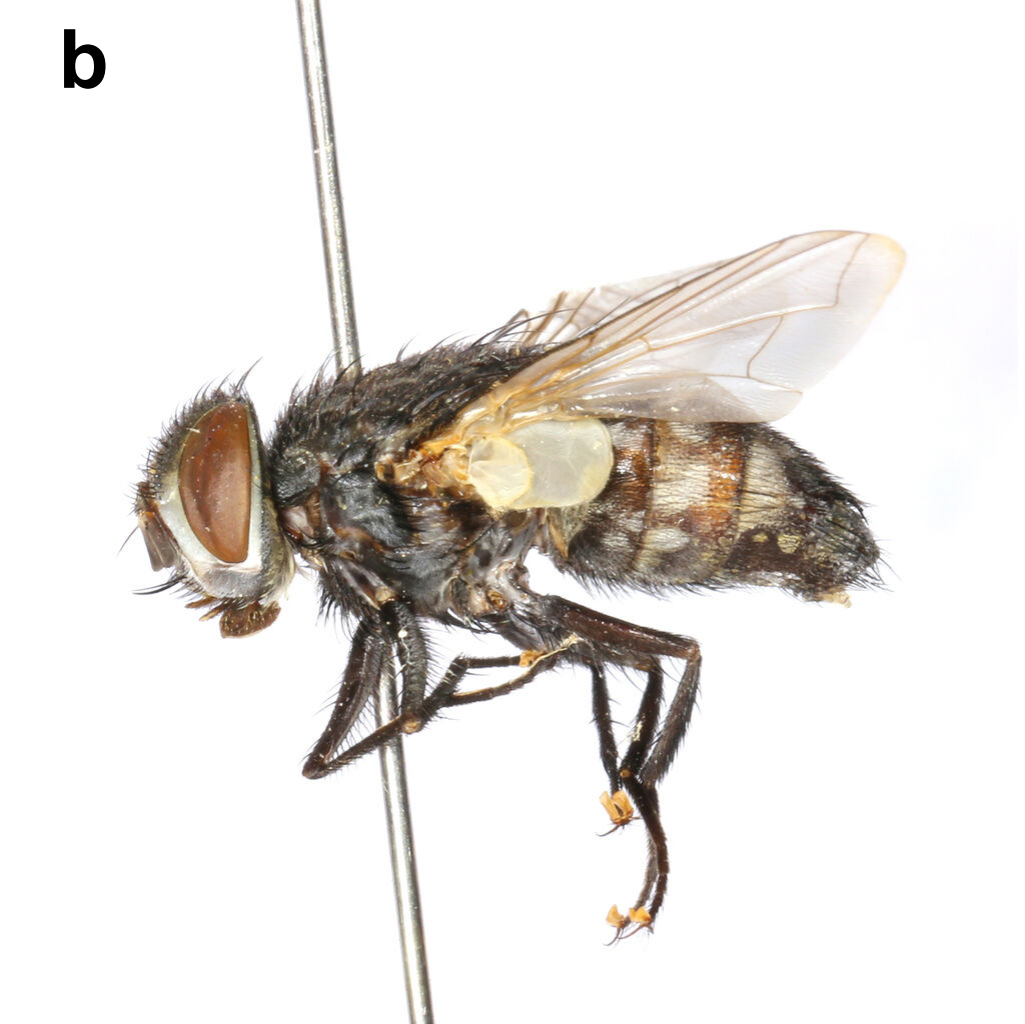
male body in lateral vie

**Figure 18c.**
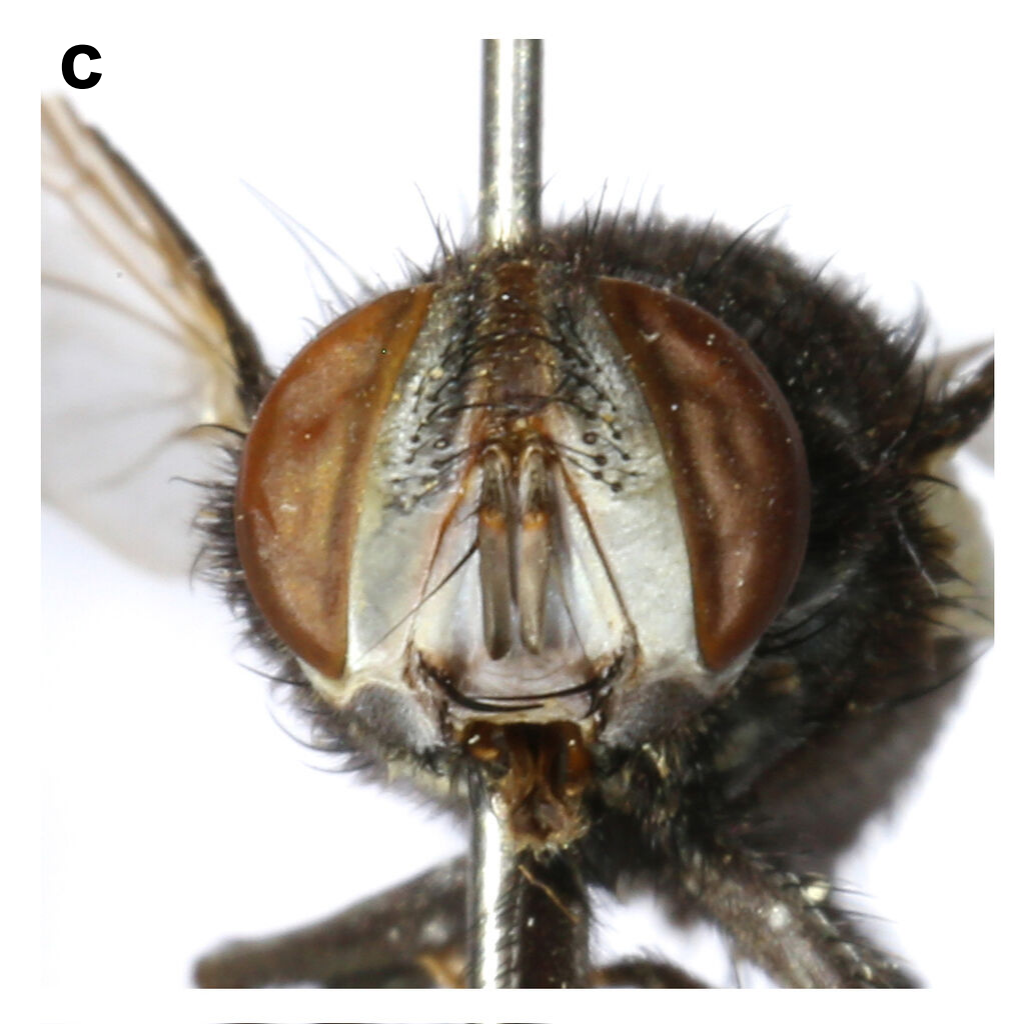
male head in anterior view

**Figure 18d.**
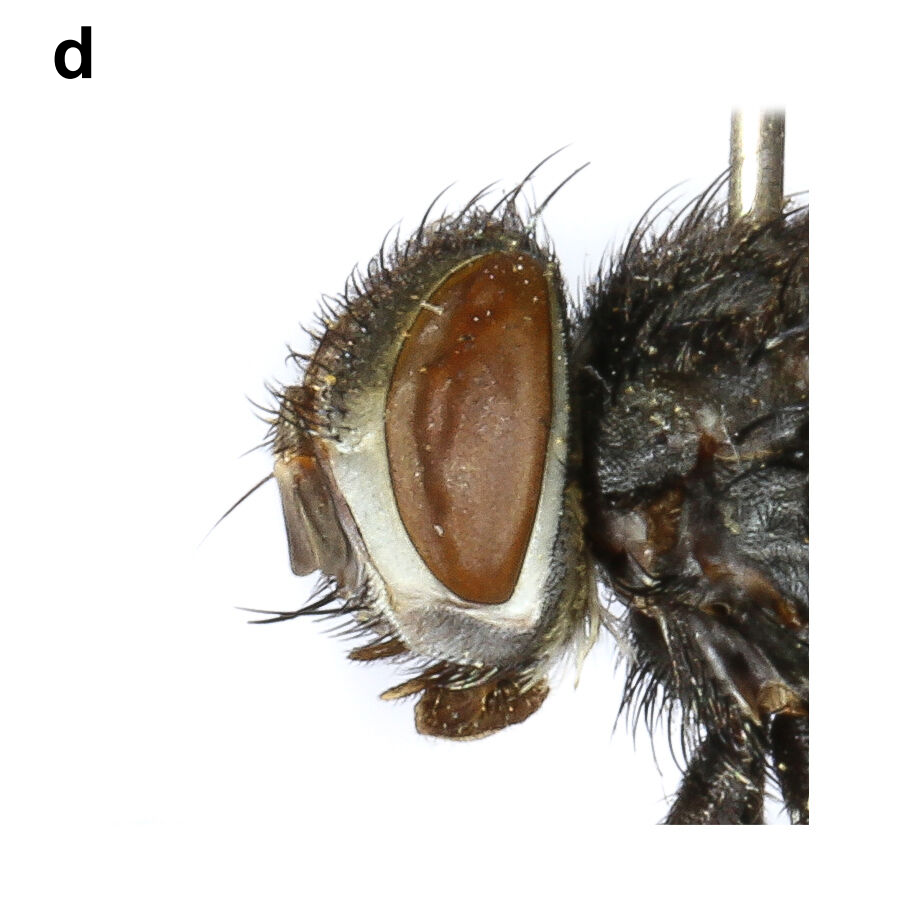
male head in lateral view

**Figure 19a.**
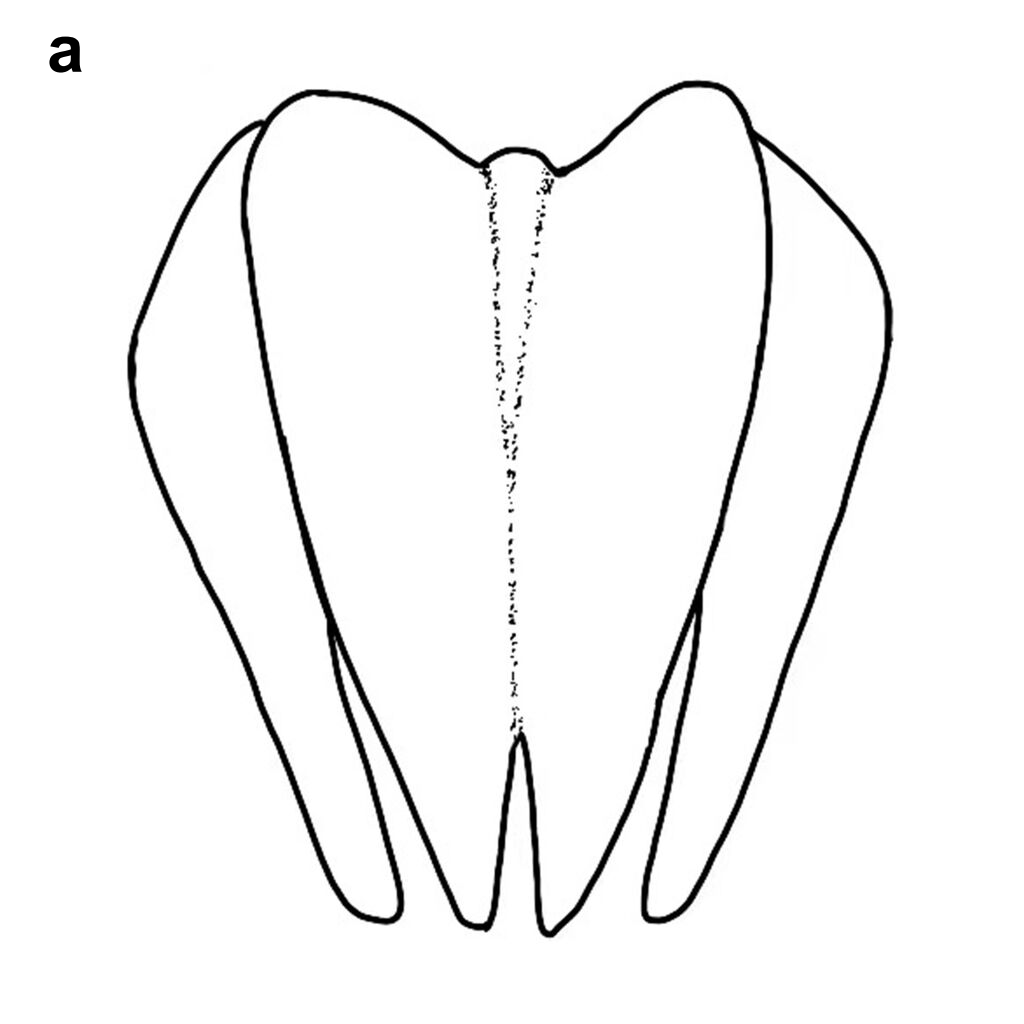
male cerci, surstyli in caudal view.

**Figure 19b.**
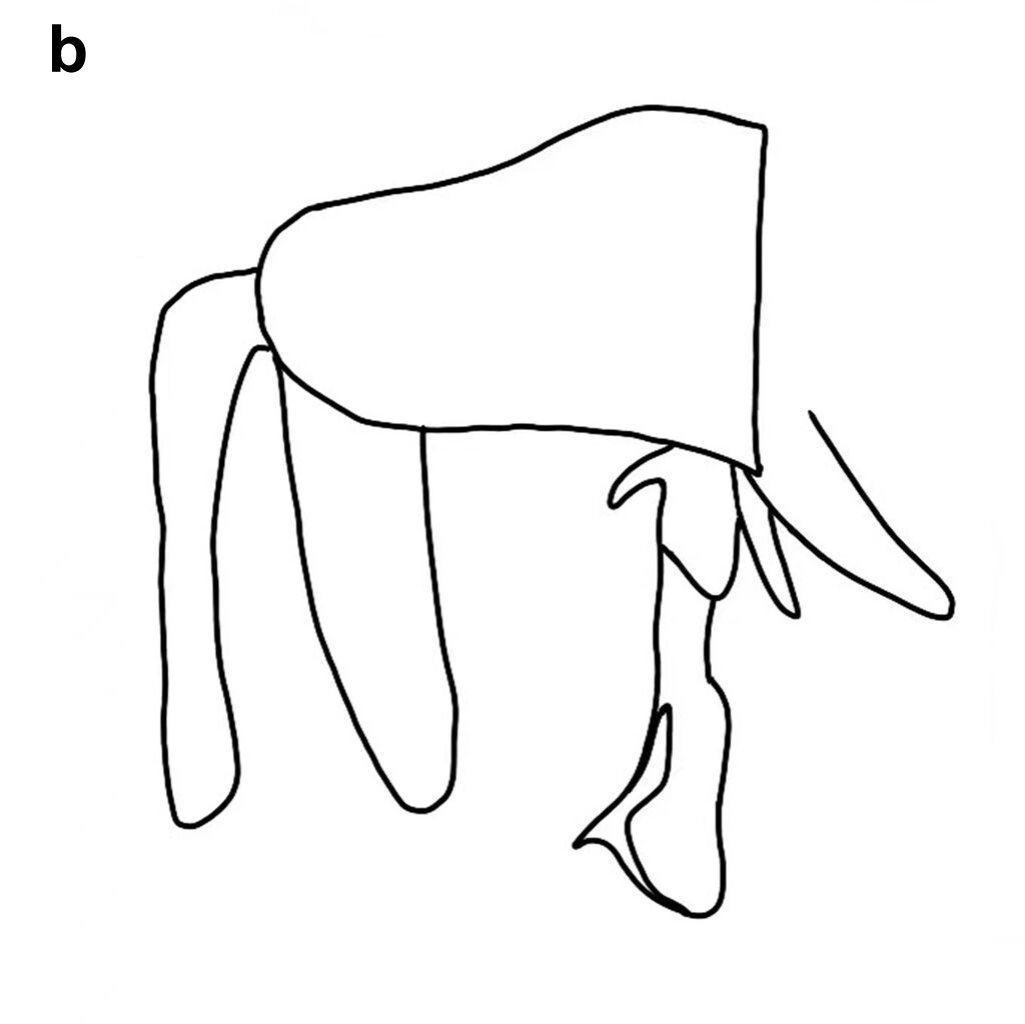
male cercus, surstylus, epandrium and phallus (part) in lateral view.

**Figure 20a.**
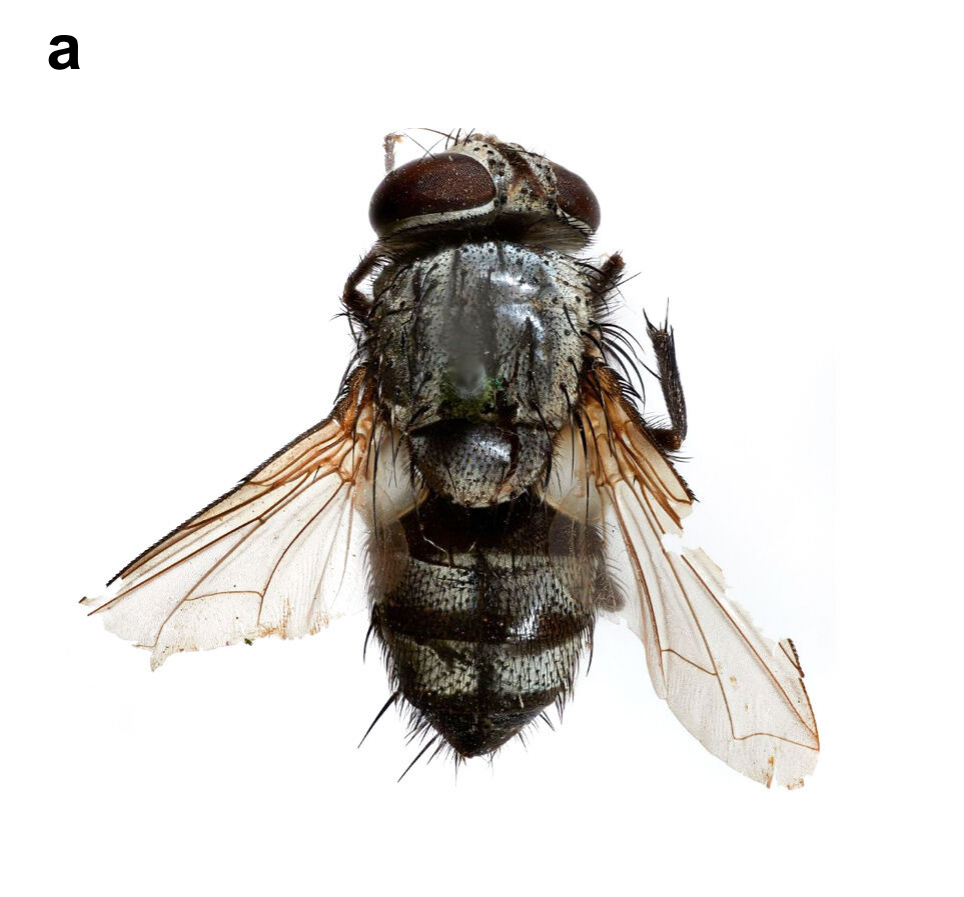
female body in dorsal view.

**Figure 20b.**
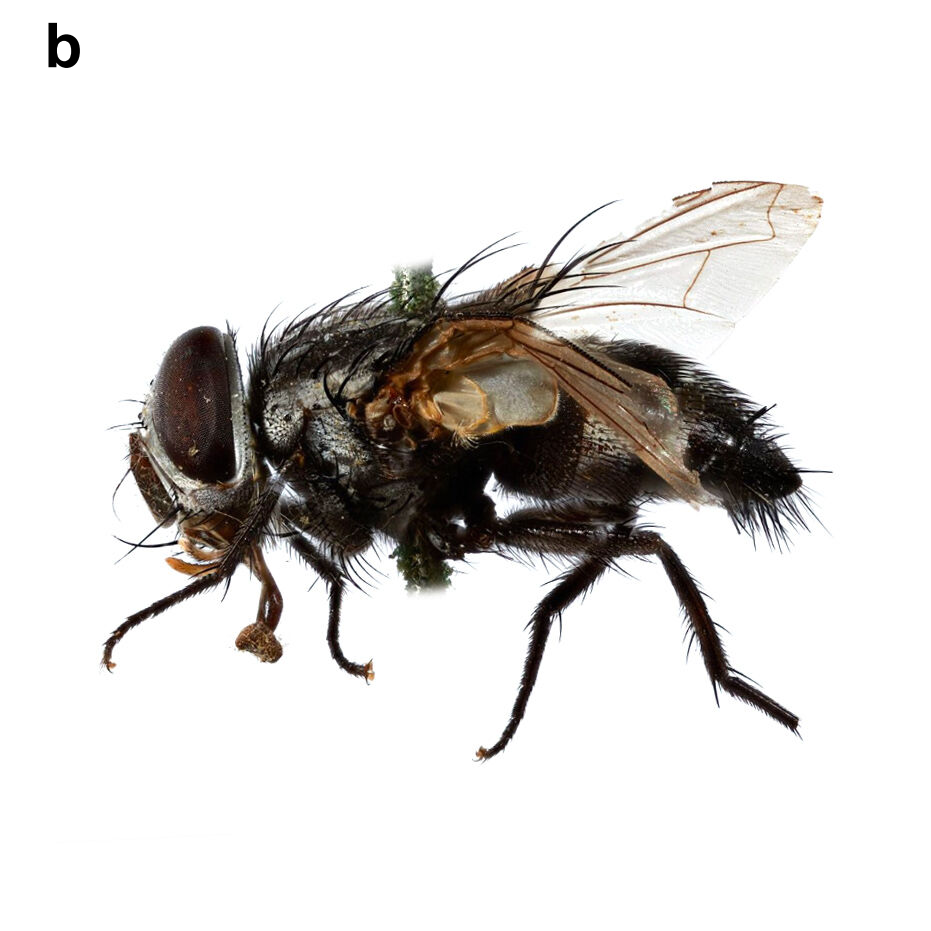
female body in lateral view.

**Figure 20c.**
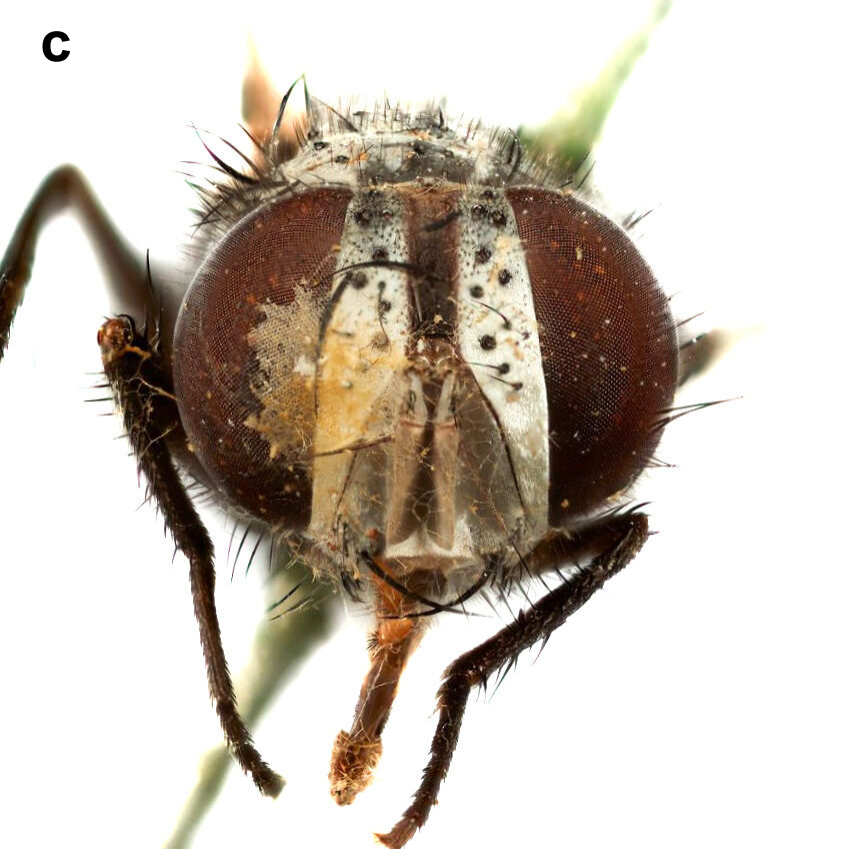
female head in anterior view

**Figure 20d.**
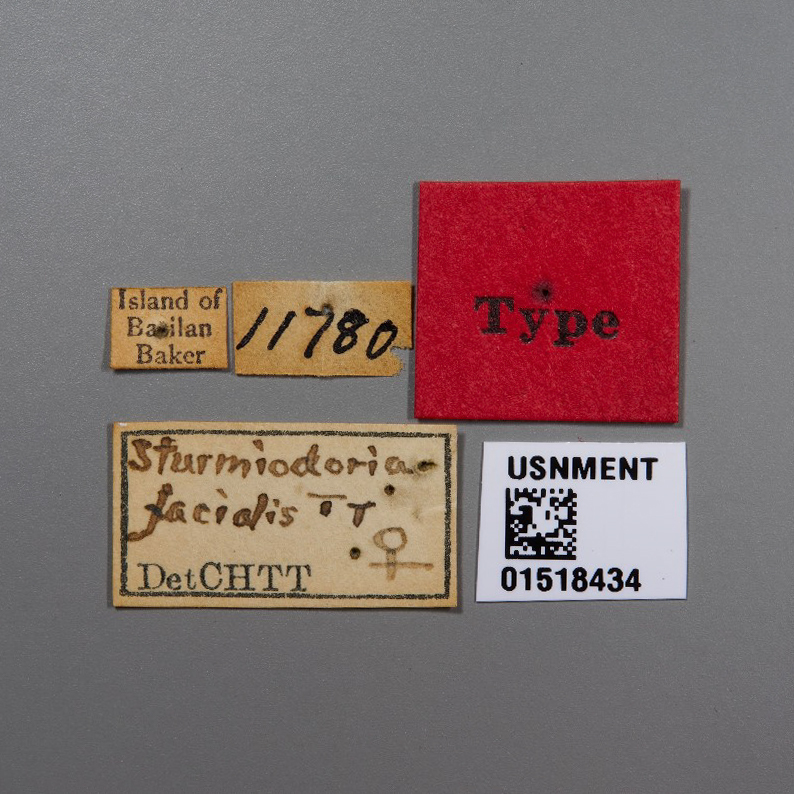
label information of *Sturmiodoria facialis* Townsend

**Figure 21a.**
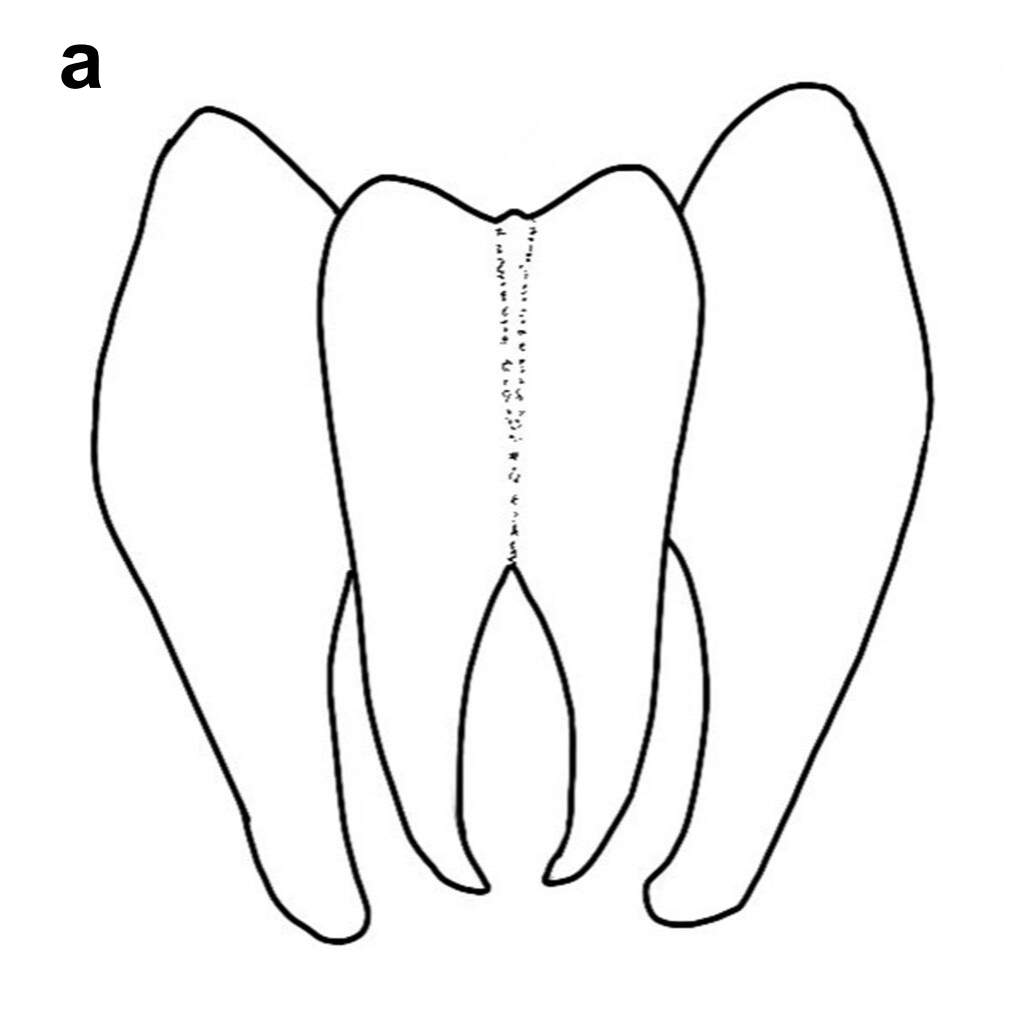
male cerci, surstyli in caudal view.

**Figure 21b.**
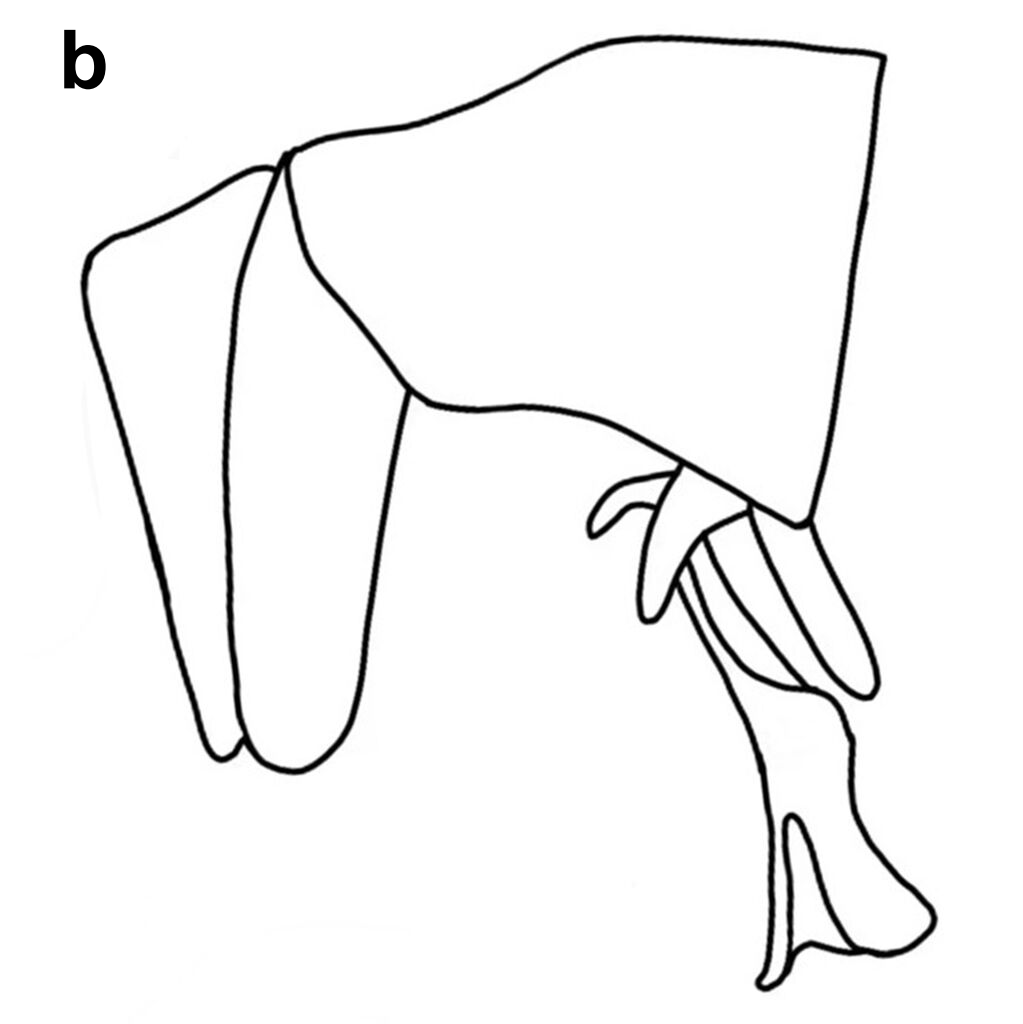
male cercus, surstylus, epandrium and phallus (part) in lateral view.

**Figure 22a.**
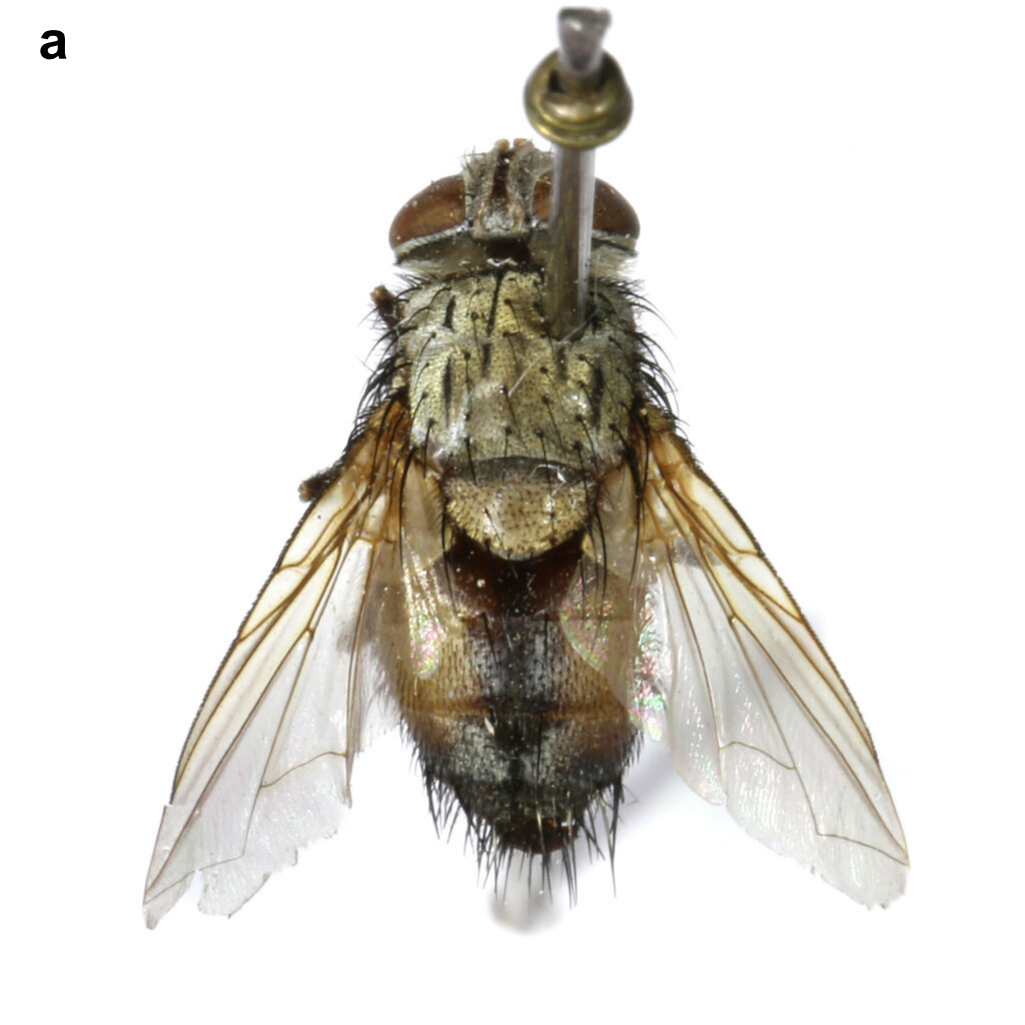
male body in dorsal view

**Figure 22b.**
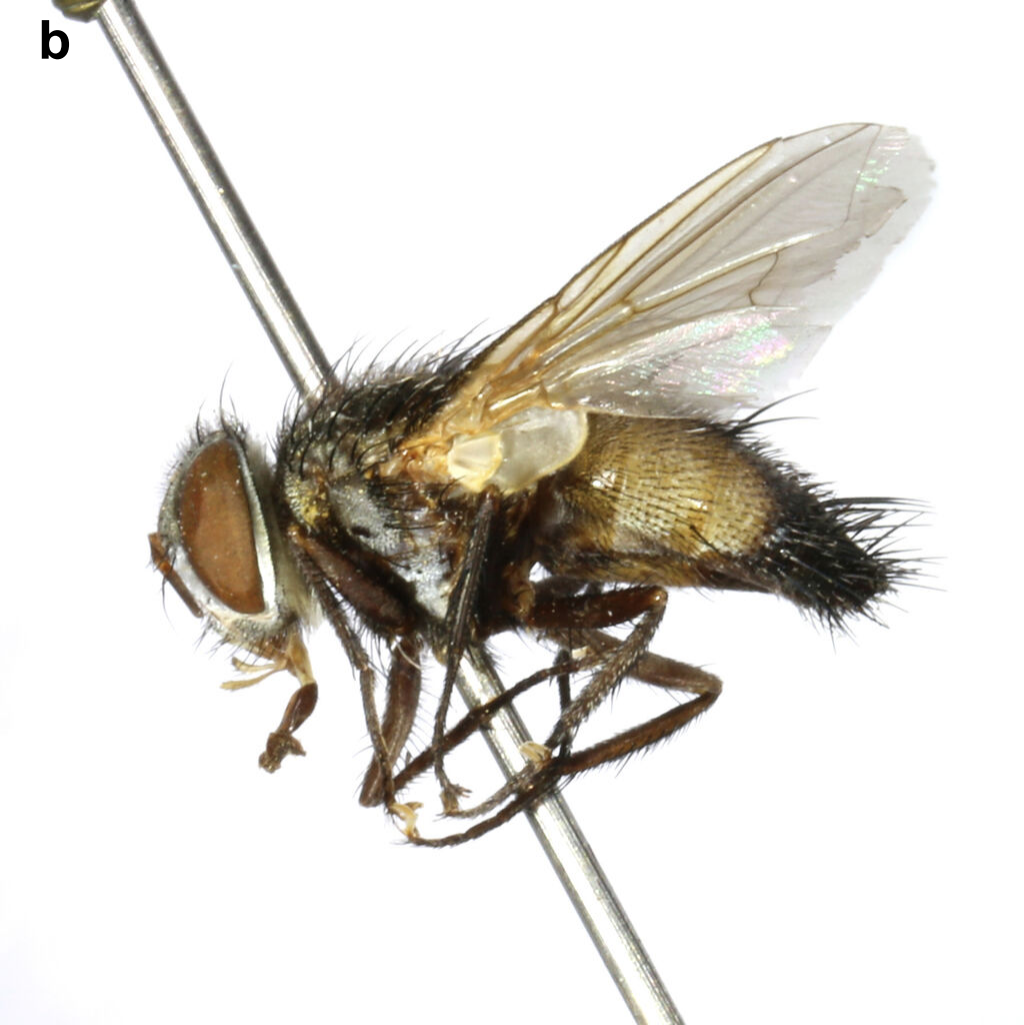
male body in lateral view

**Figure 22c.**
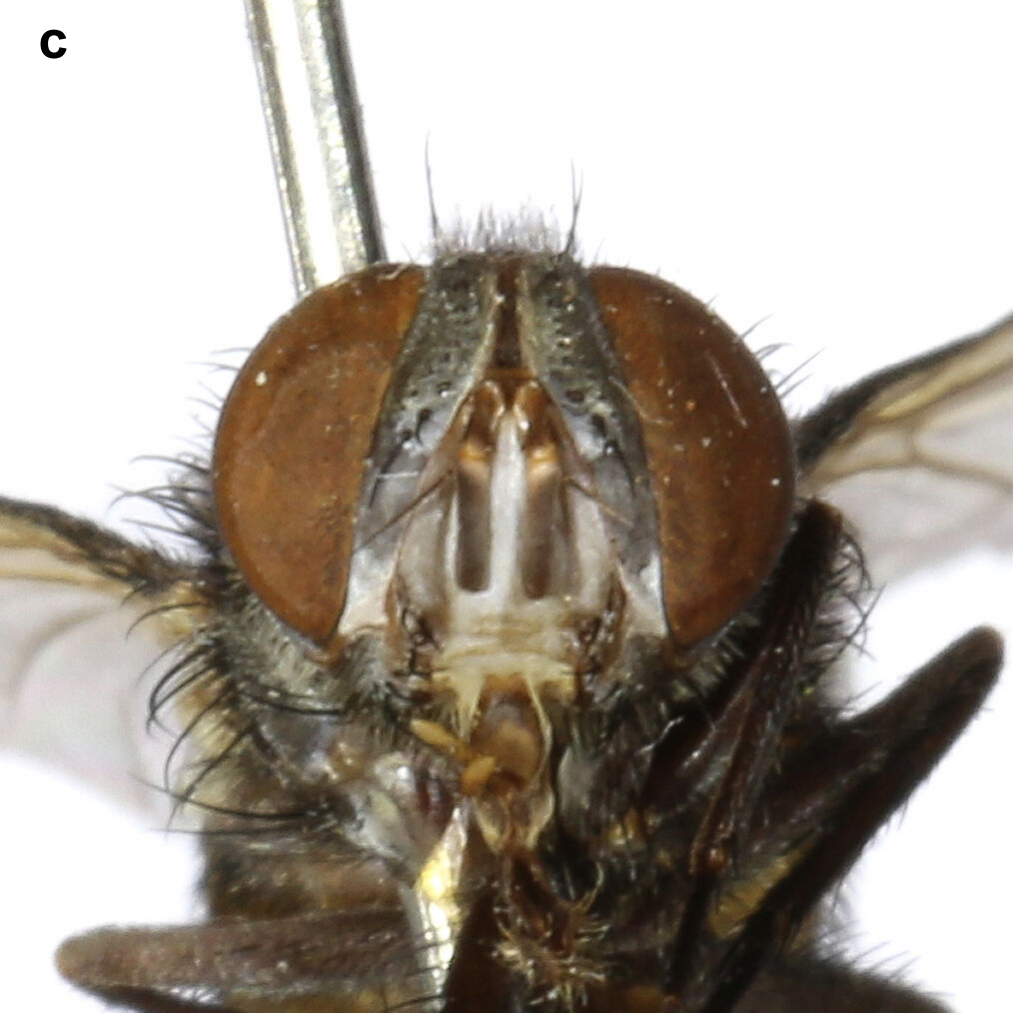
male head in anterior view

**Figure 22d.**
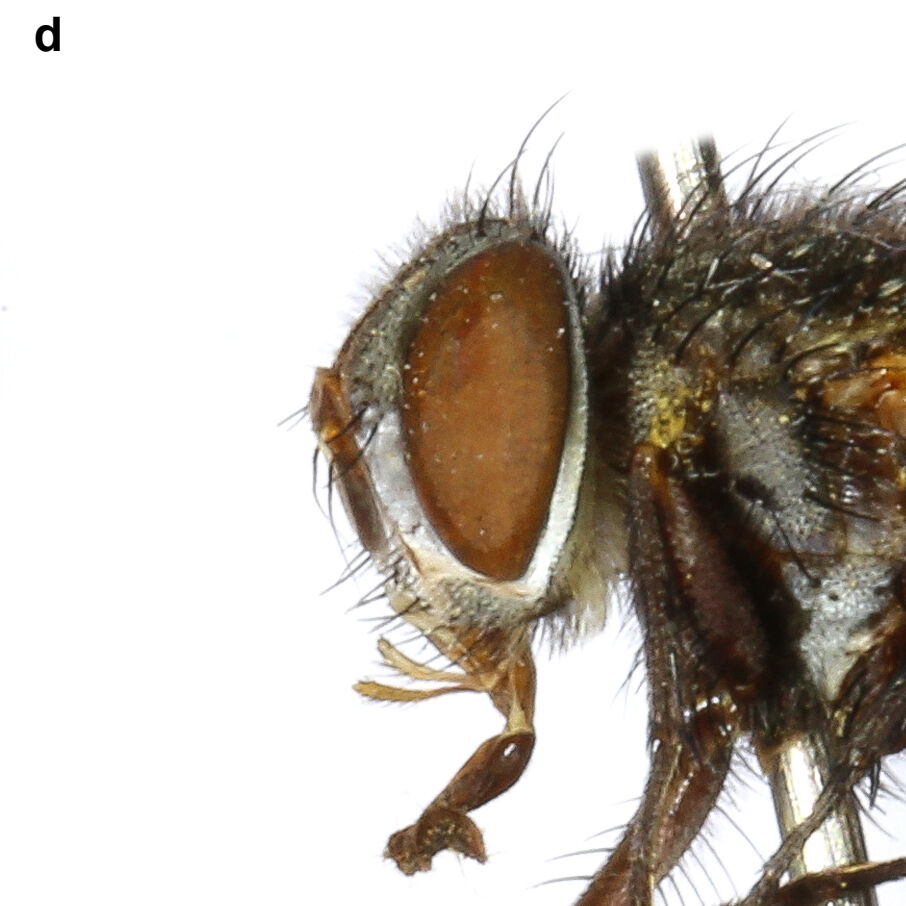
male head in lateral vie

**Figure 23a.**
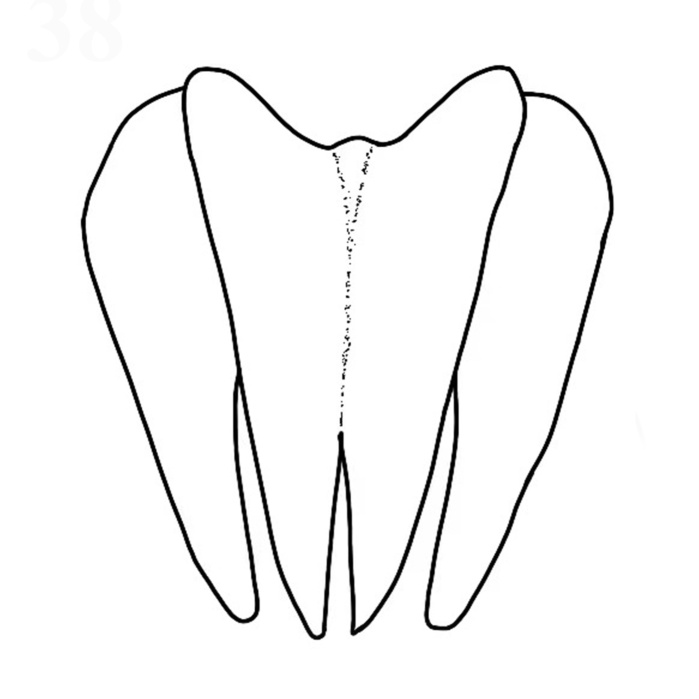
male cerci, surstyli in caudal view.

**Figure 23b.**
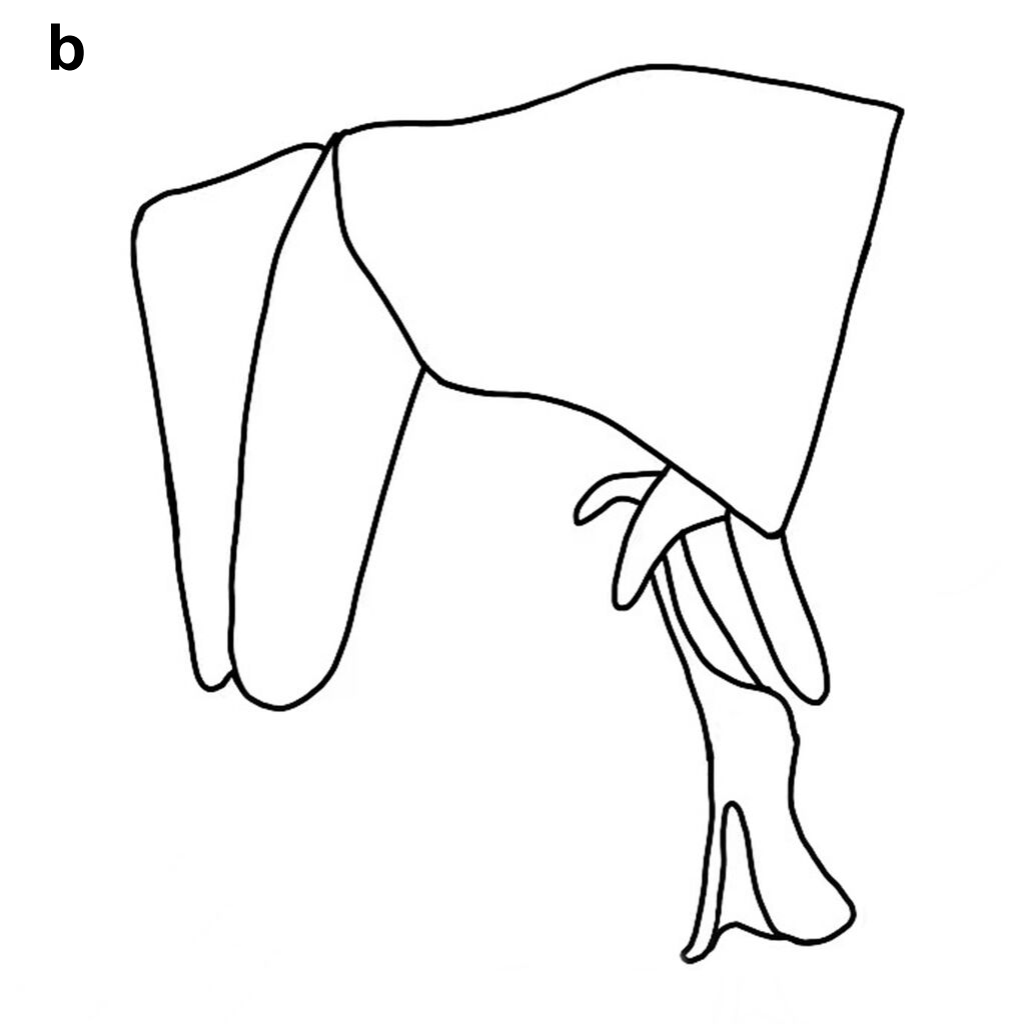
male cercus, surstylus, epandrium and phallus (part) in lateral view.

**Figure 24a.**
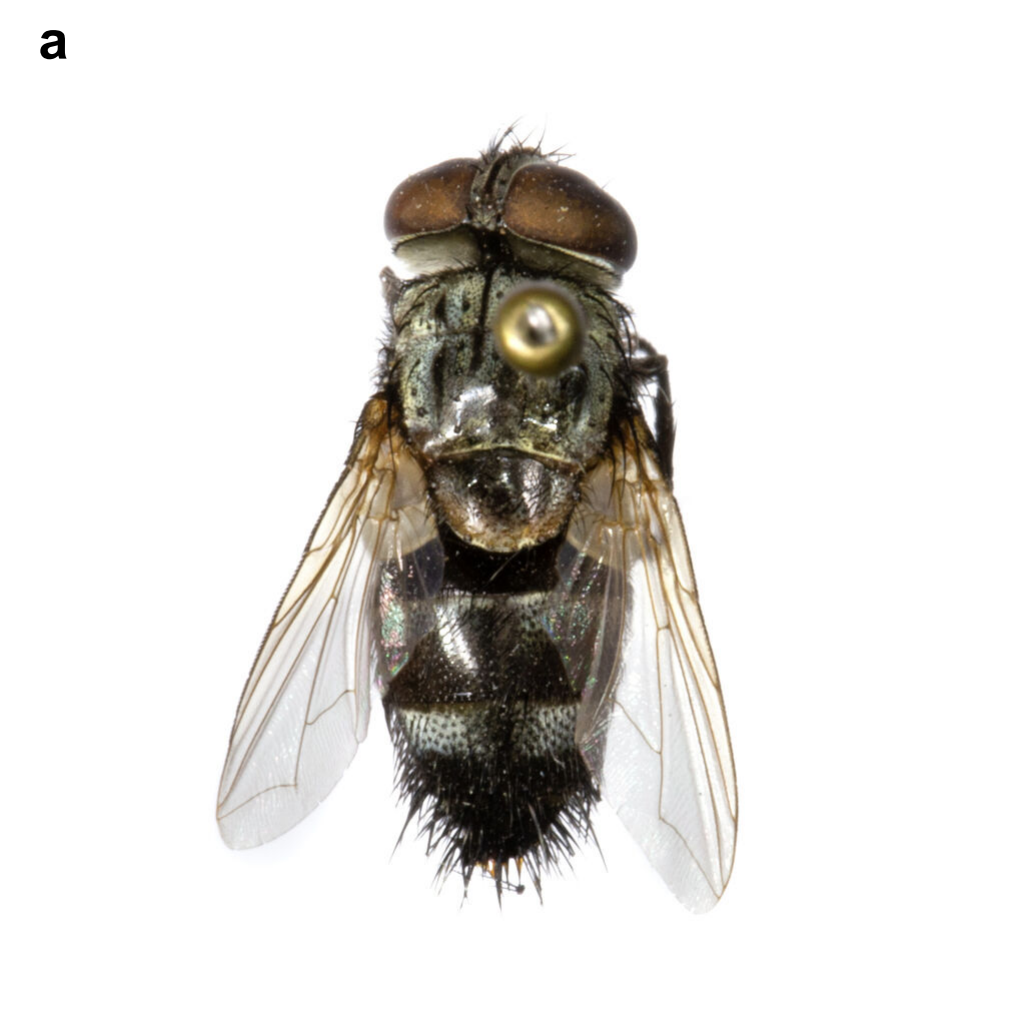
male body in dorsal view

**Figure 24b.**
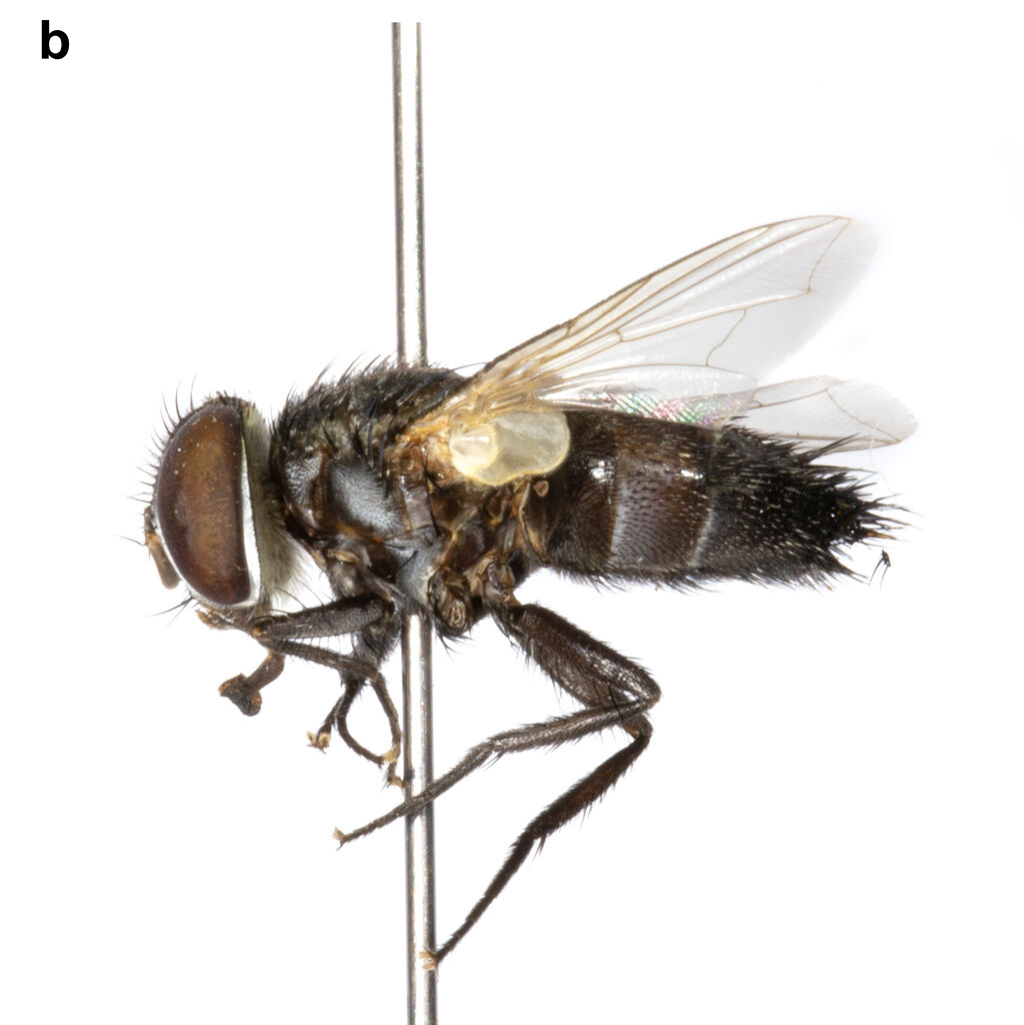
male body in lateral view

**Figure 24c.**
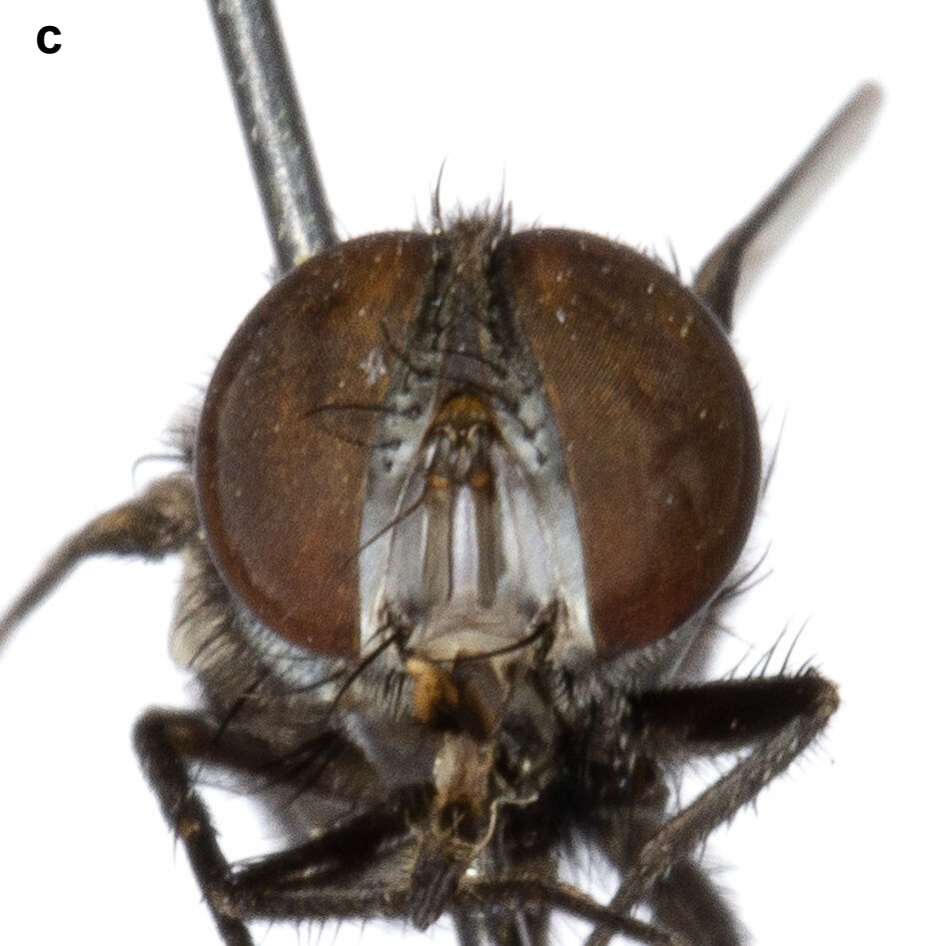
male head in anterior view

**Figure 24d.**
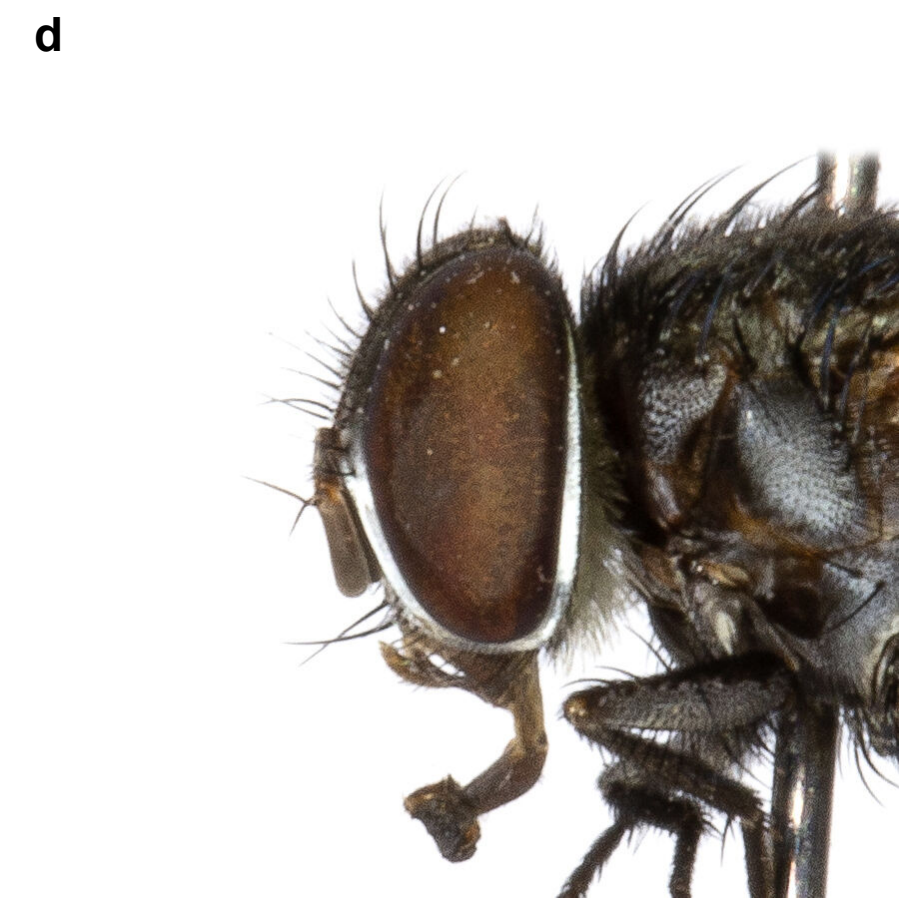
male head in lateral view

**Figure 25a.**
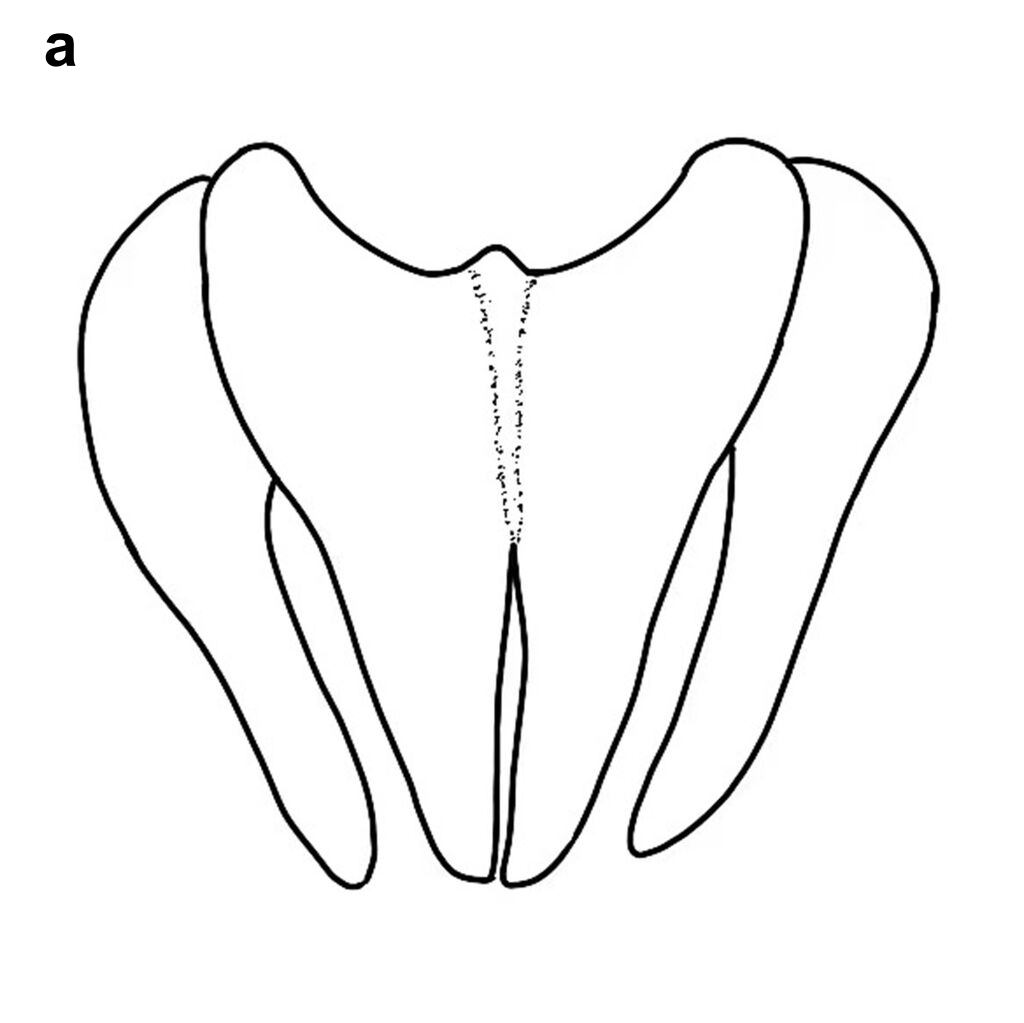
male cerci, surstyli in caudal view.

**Figure 25b.**
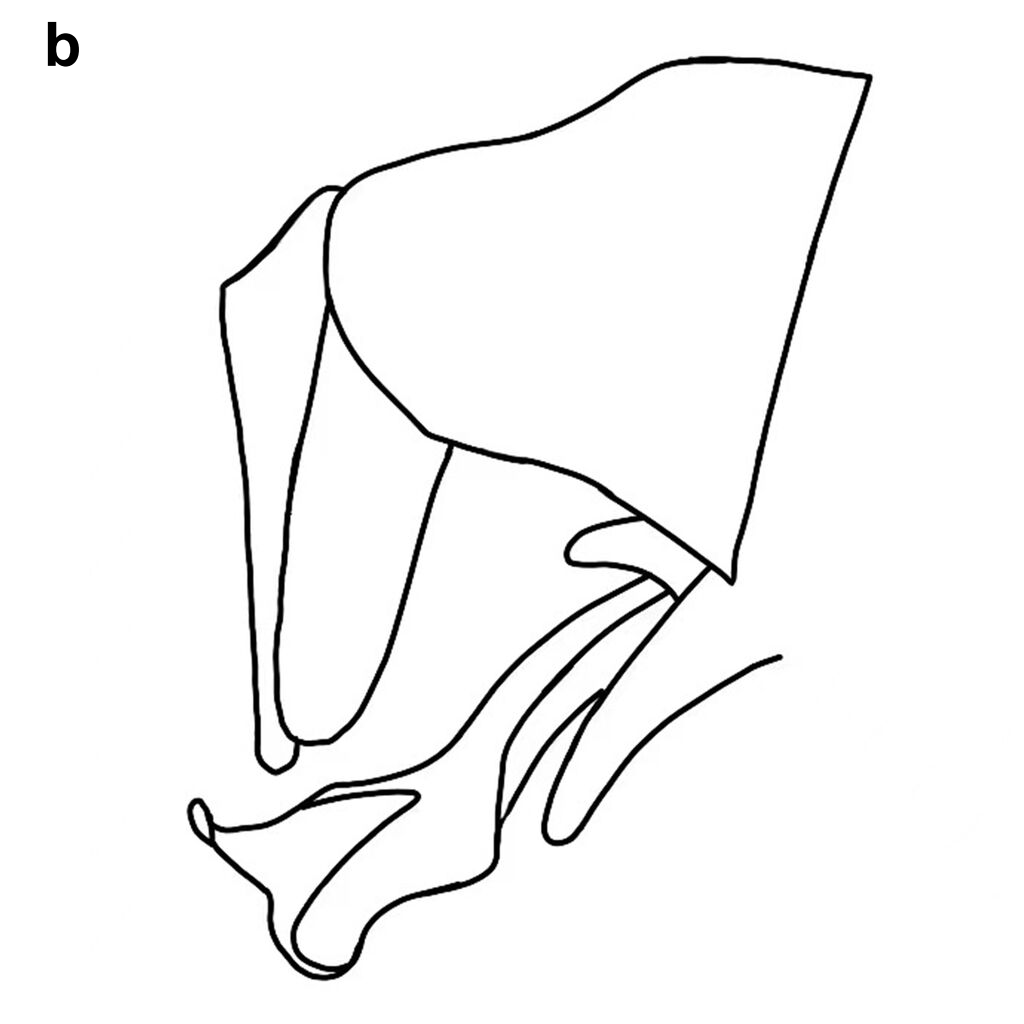
male cercus, surstylus, epandrium and phallus (part) in lateral view.

**Figure 26a.**
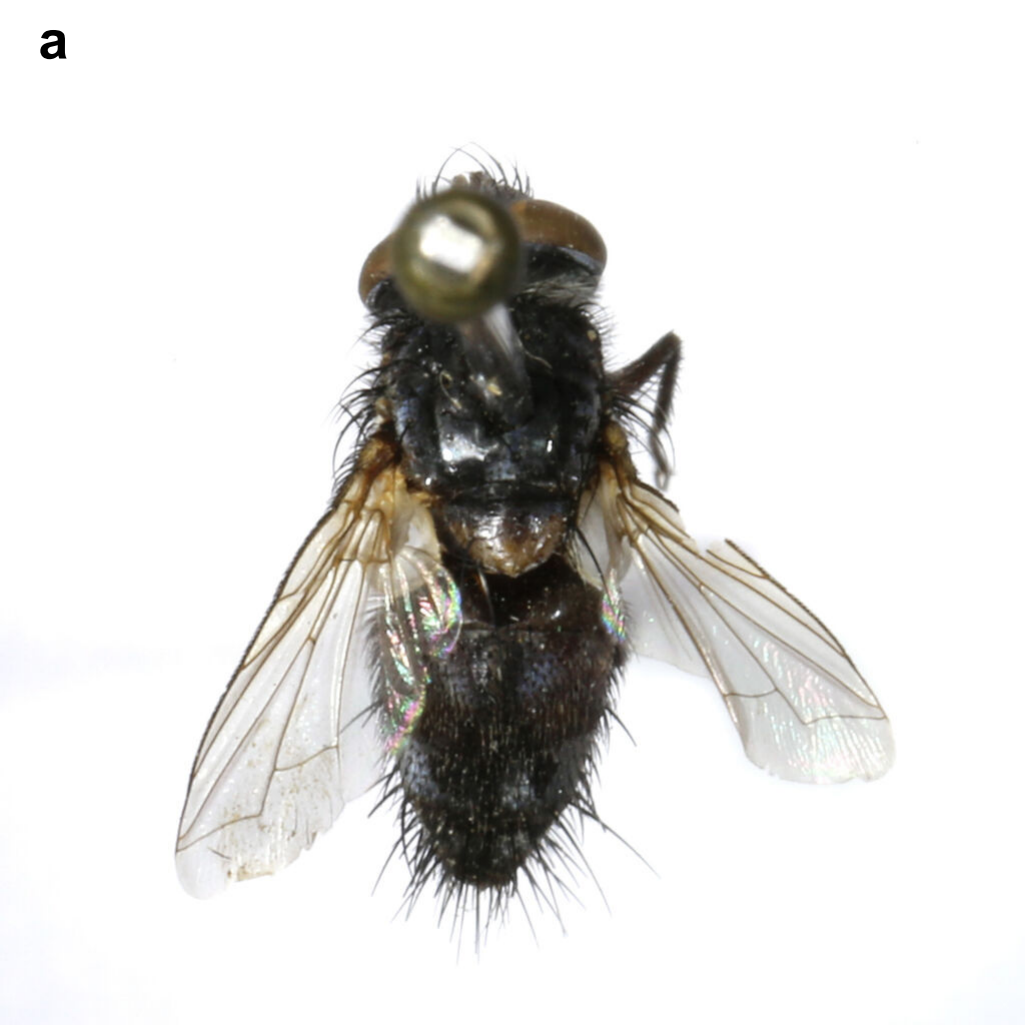
male body in dorsal view

**Figure 26b.**
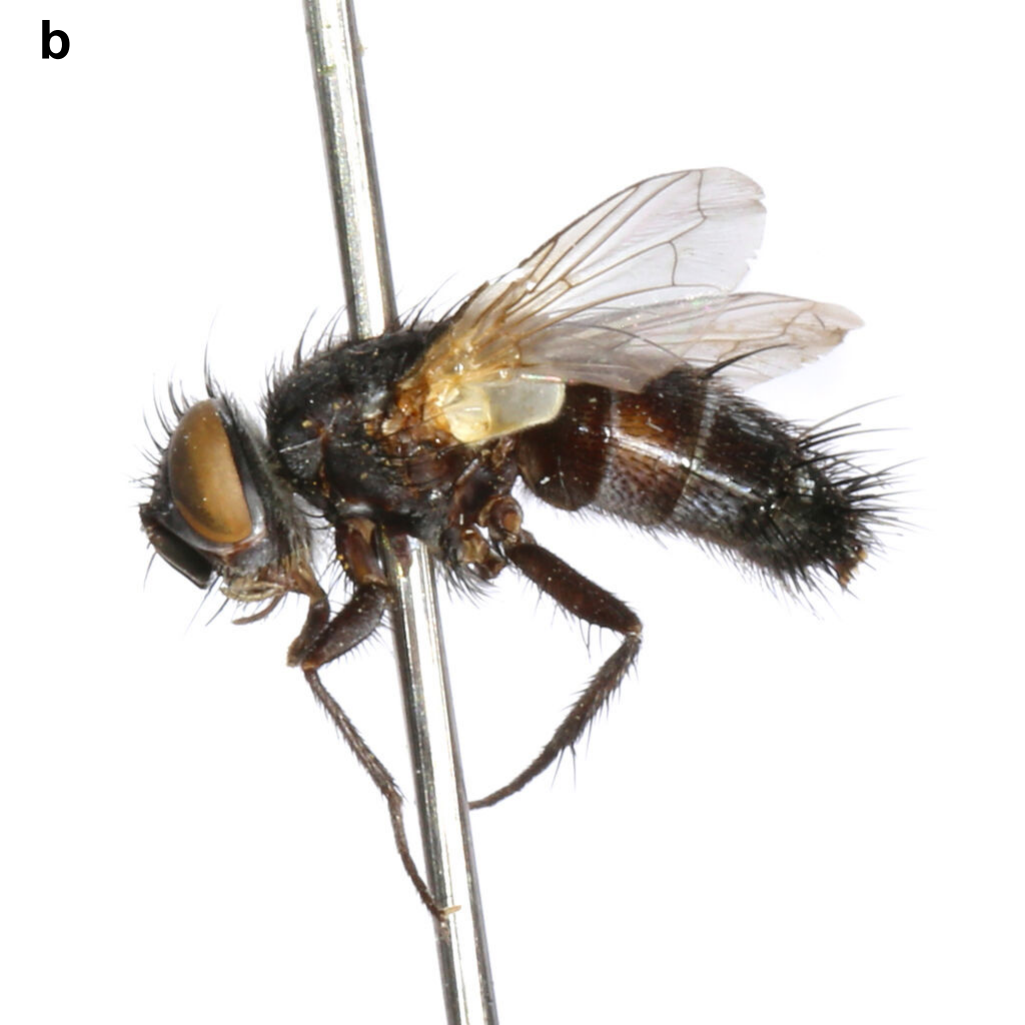
male body in lateral view

**Figure 26c.**
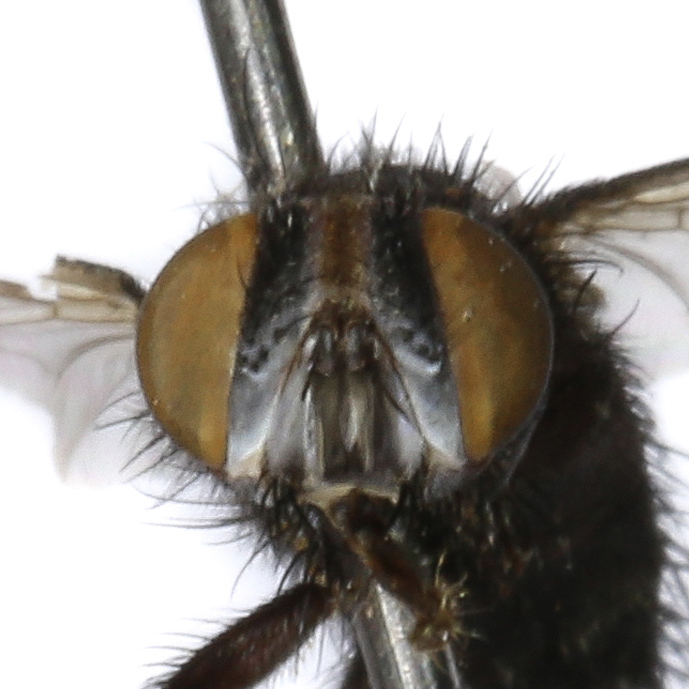
male head in anterior view

**Figure 26d.**
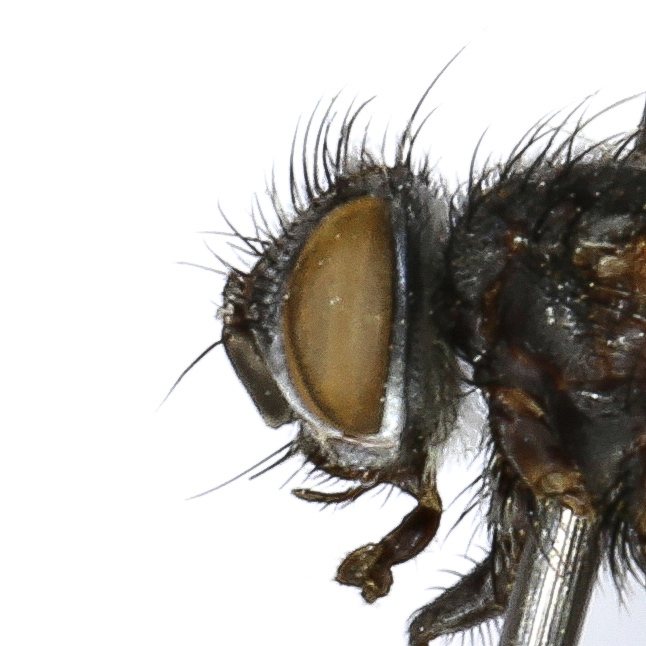
male head in lateral vie

**Figure 27a.**
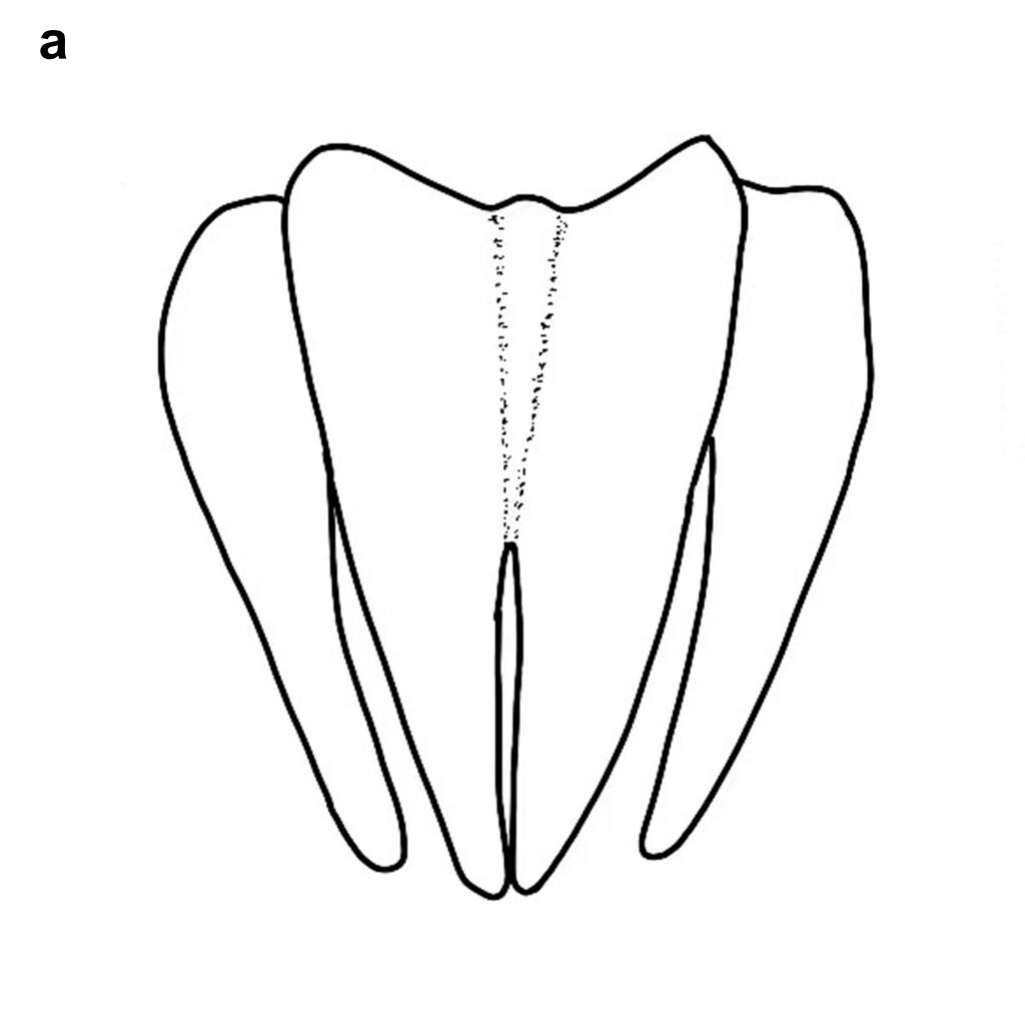
male cerci, surstyli in caudal view.

**Figure 27b.**
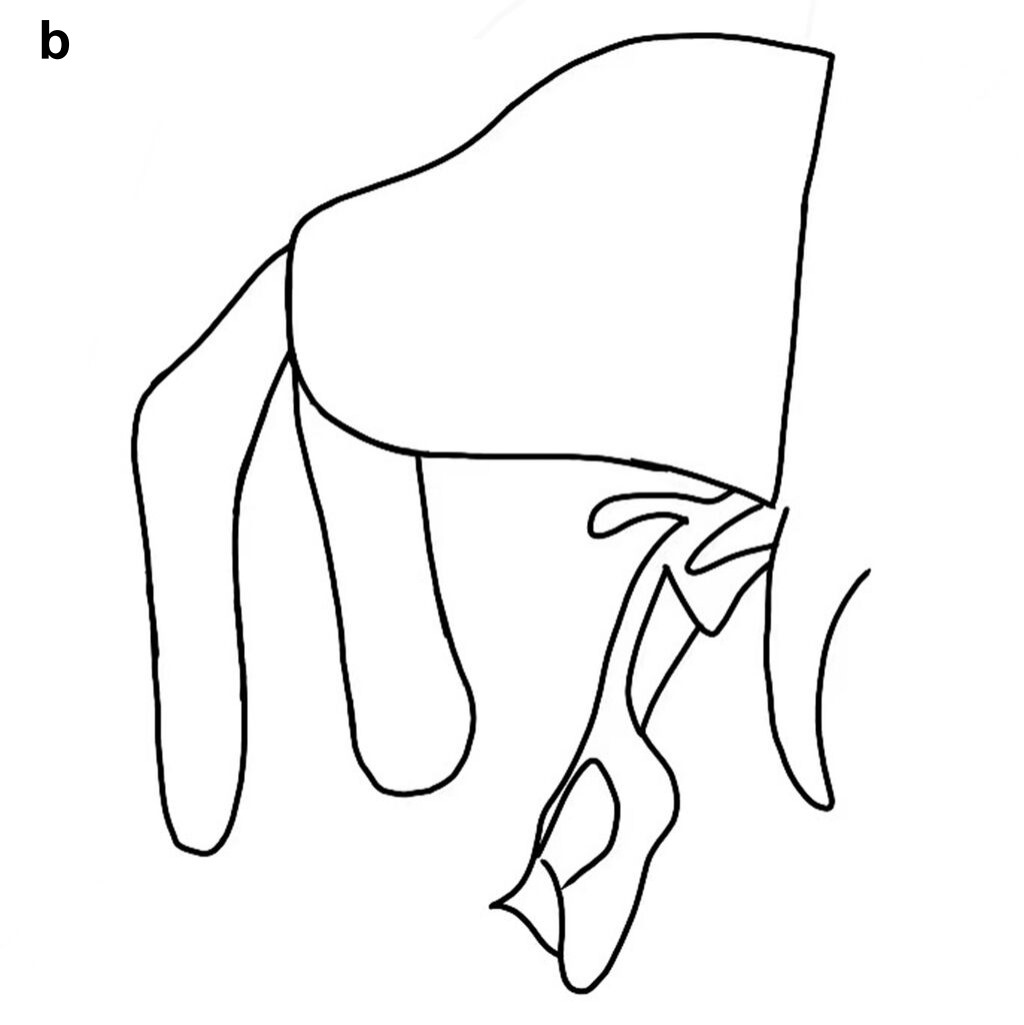
male cercus, surstylus, epandrium and phallus (part) in lateral view.

**Figure 28a.**
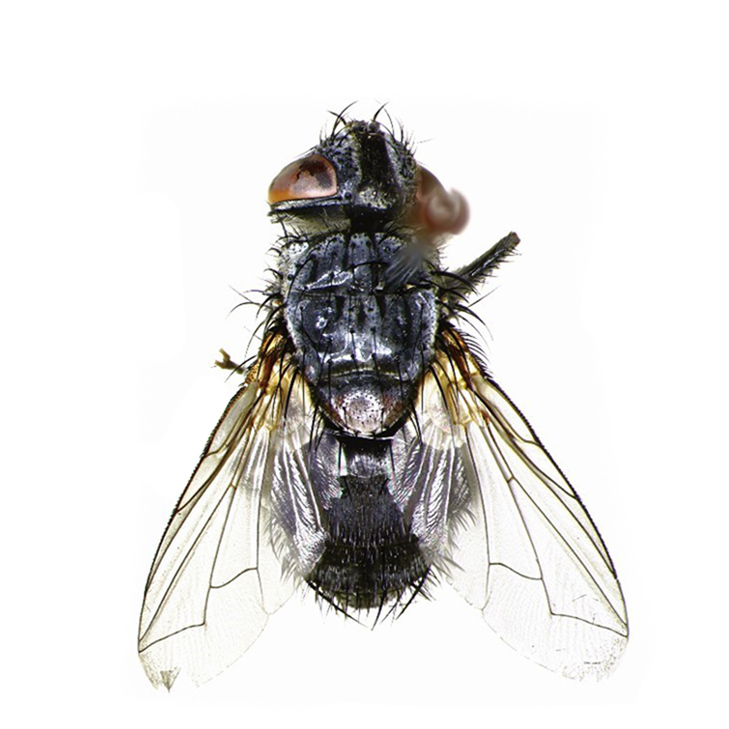
male body in dorsal view

**Figure 28b.**
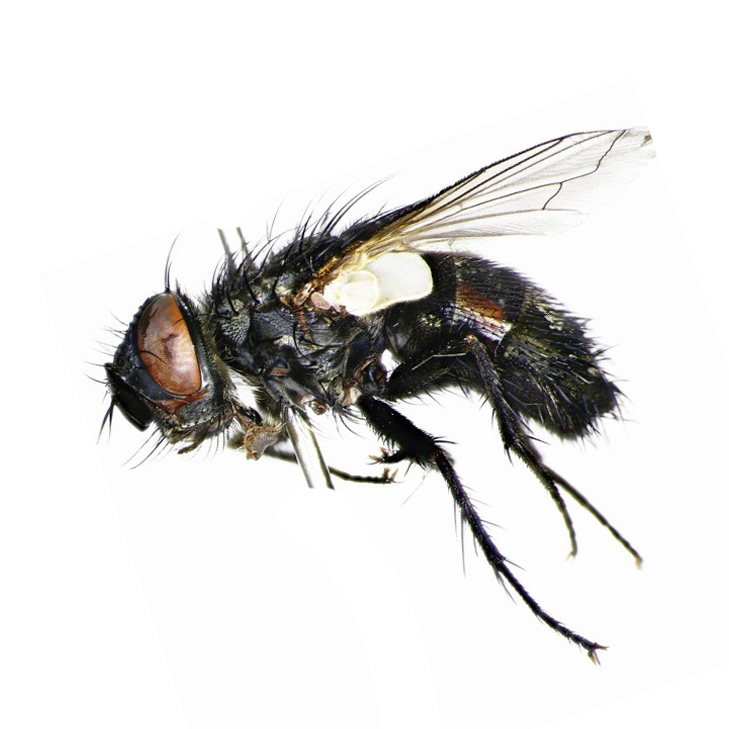
male body in lateral view

**Figure 28c.**
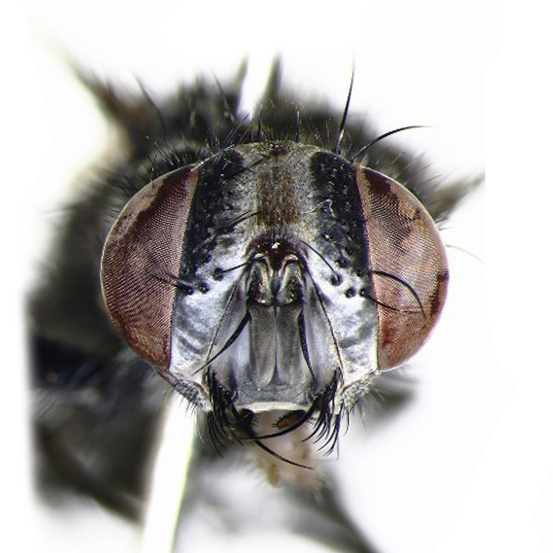
male head in anterior view

**Figure 28d.**
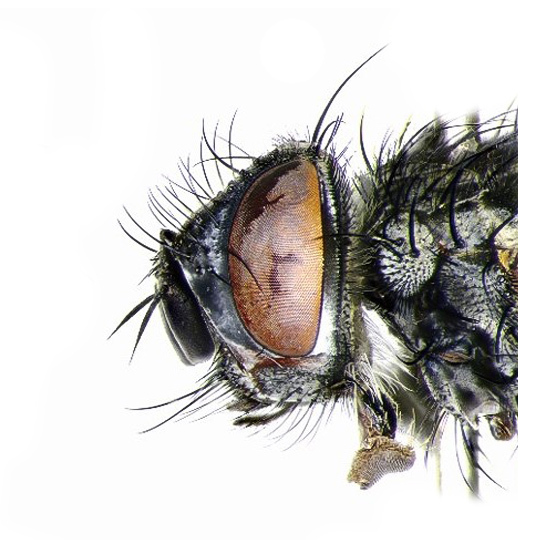
male head in lateral view

**Figure 29a.**
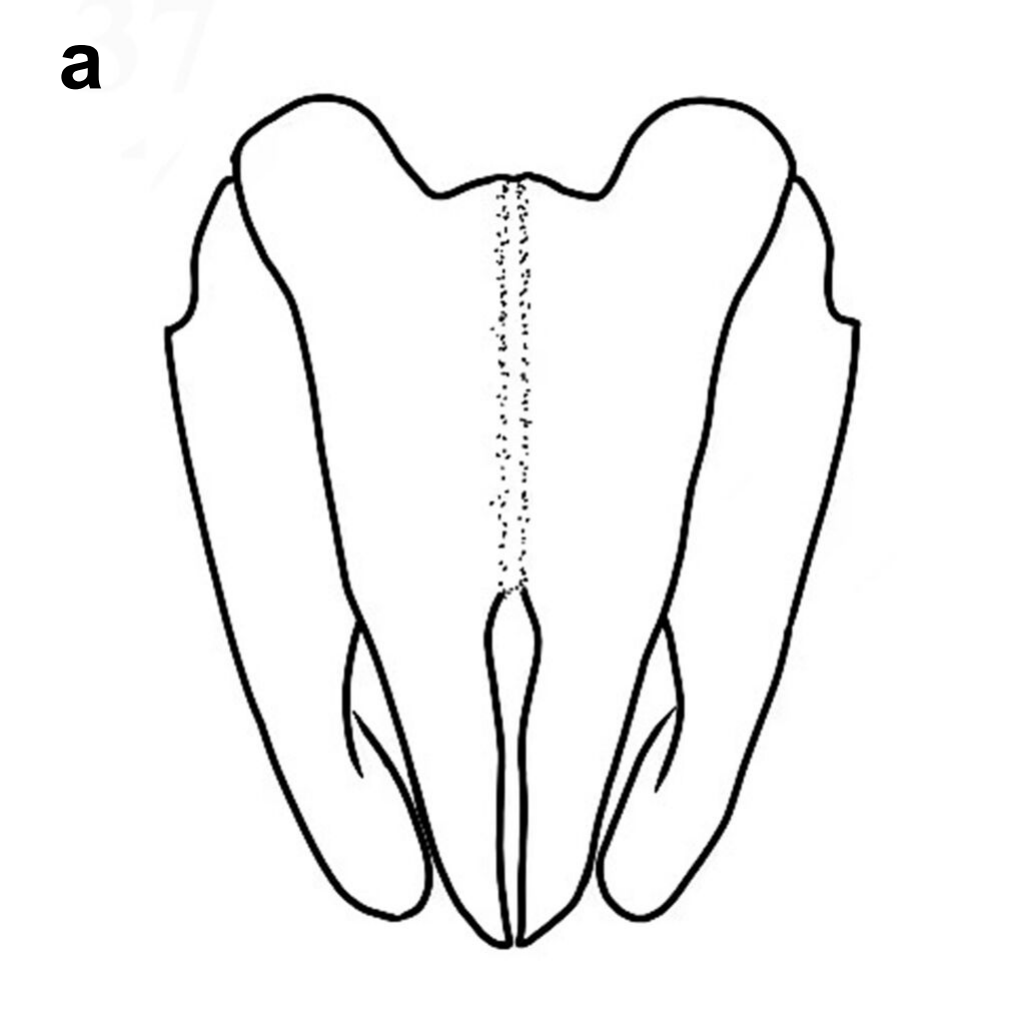
male cerci, surstyli in caudal view.

**Figure 29b.**
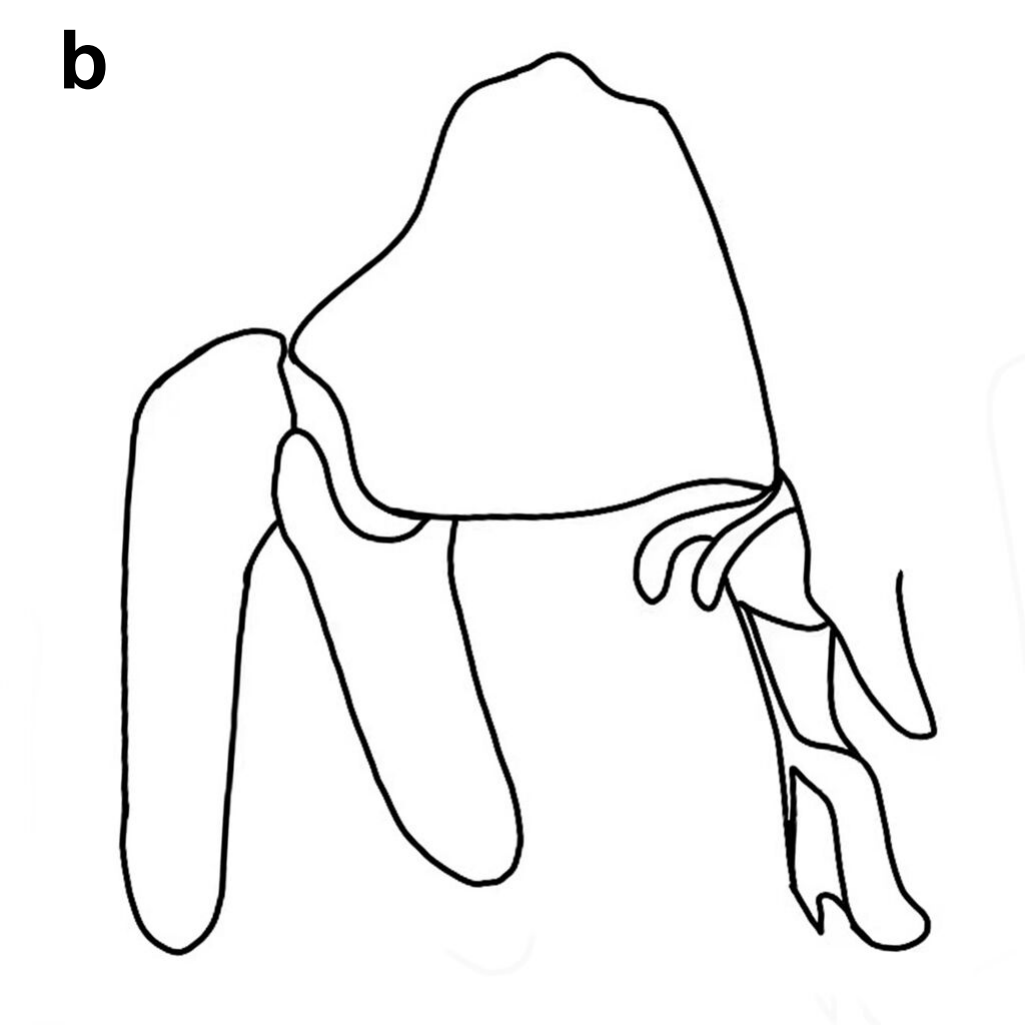
male cercus, surstylus, epandrium and phallus (part) in lateral view.

**Figure 30a.**
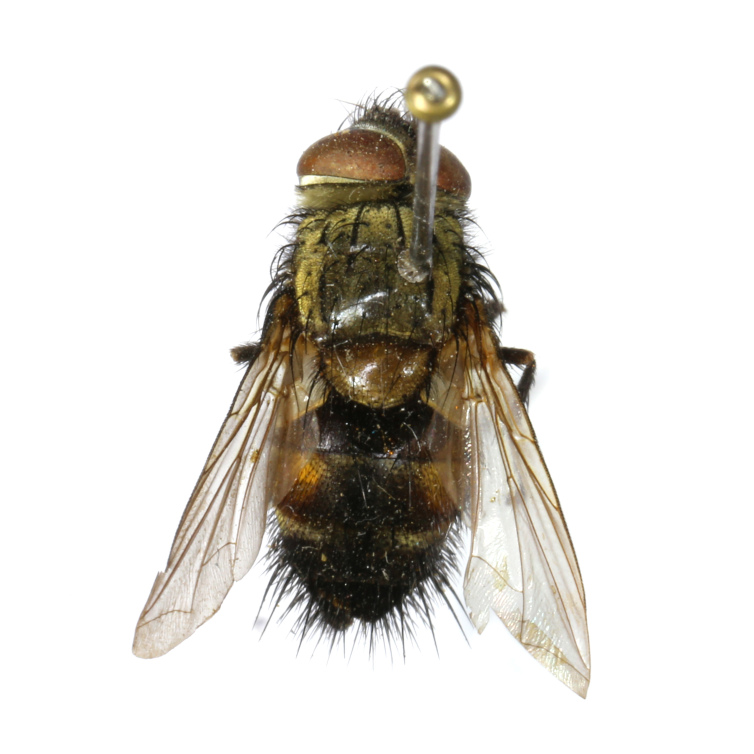
male body in dorsal view

**Figure 30b.**
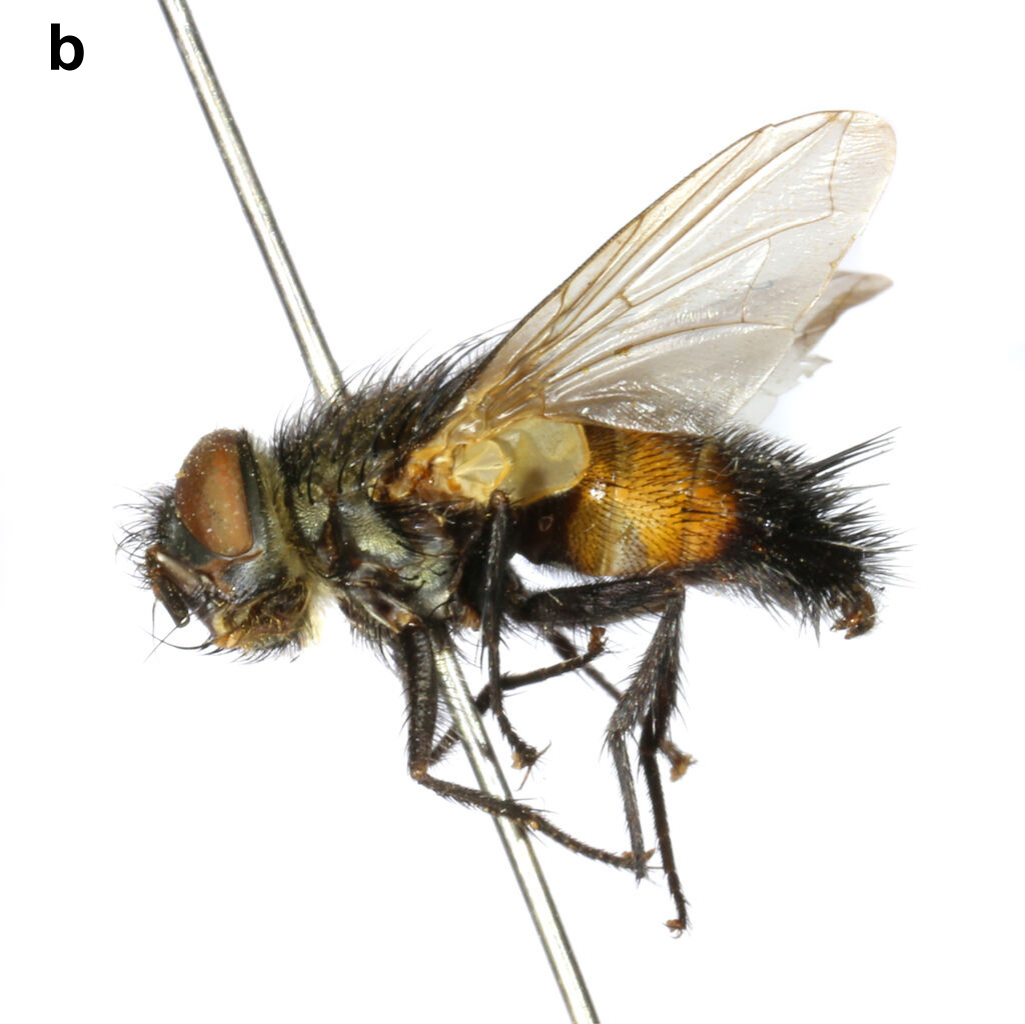
male body in lateral view

**Figure 30c.**
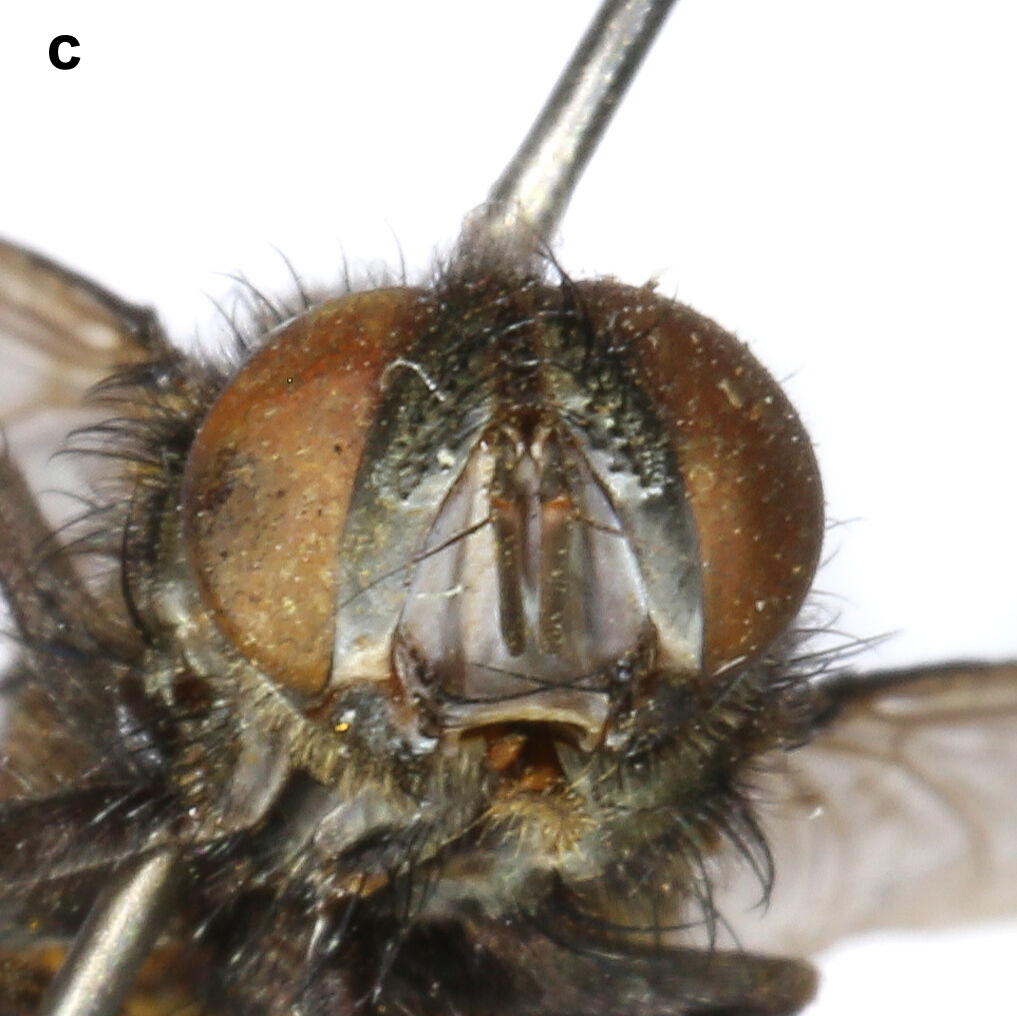
male head in anterior view

**Figure 30d.**
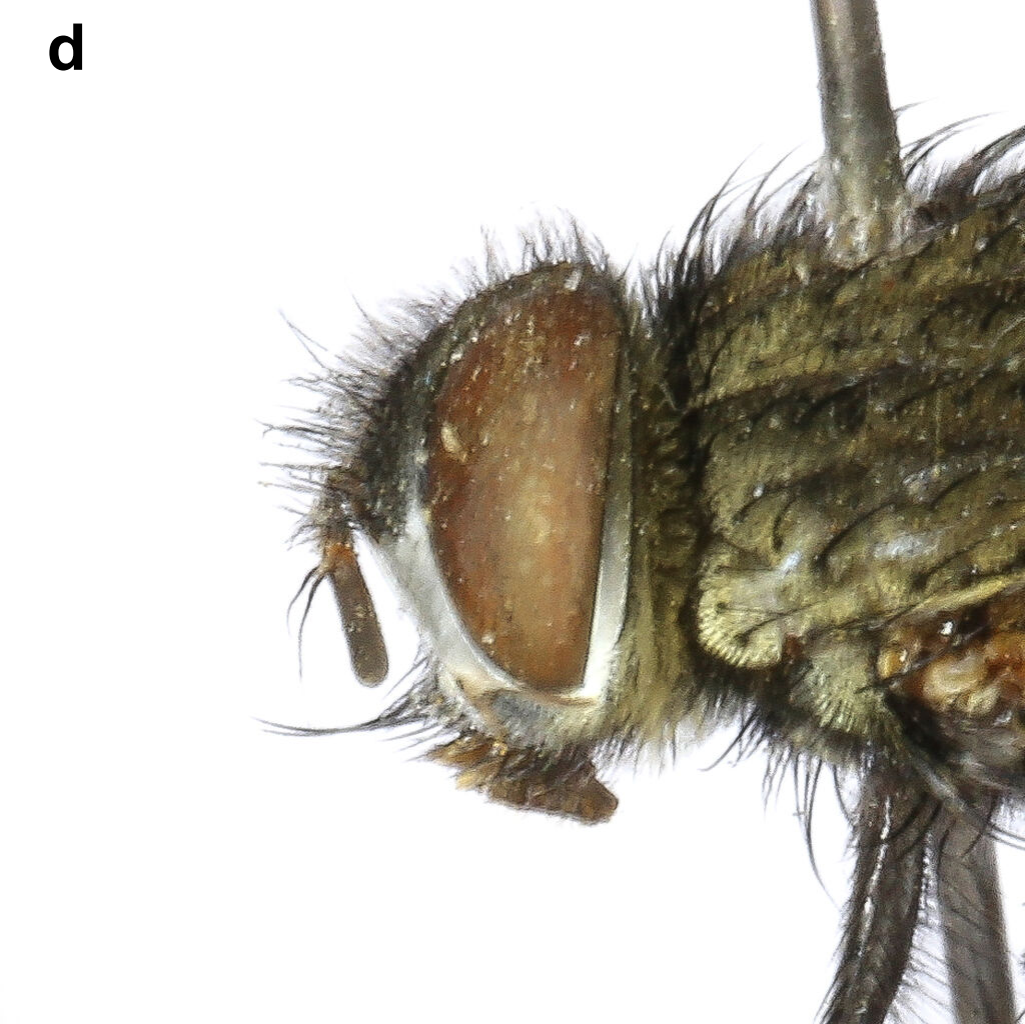
male head in lateral view

**Figure 31a.**
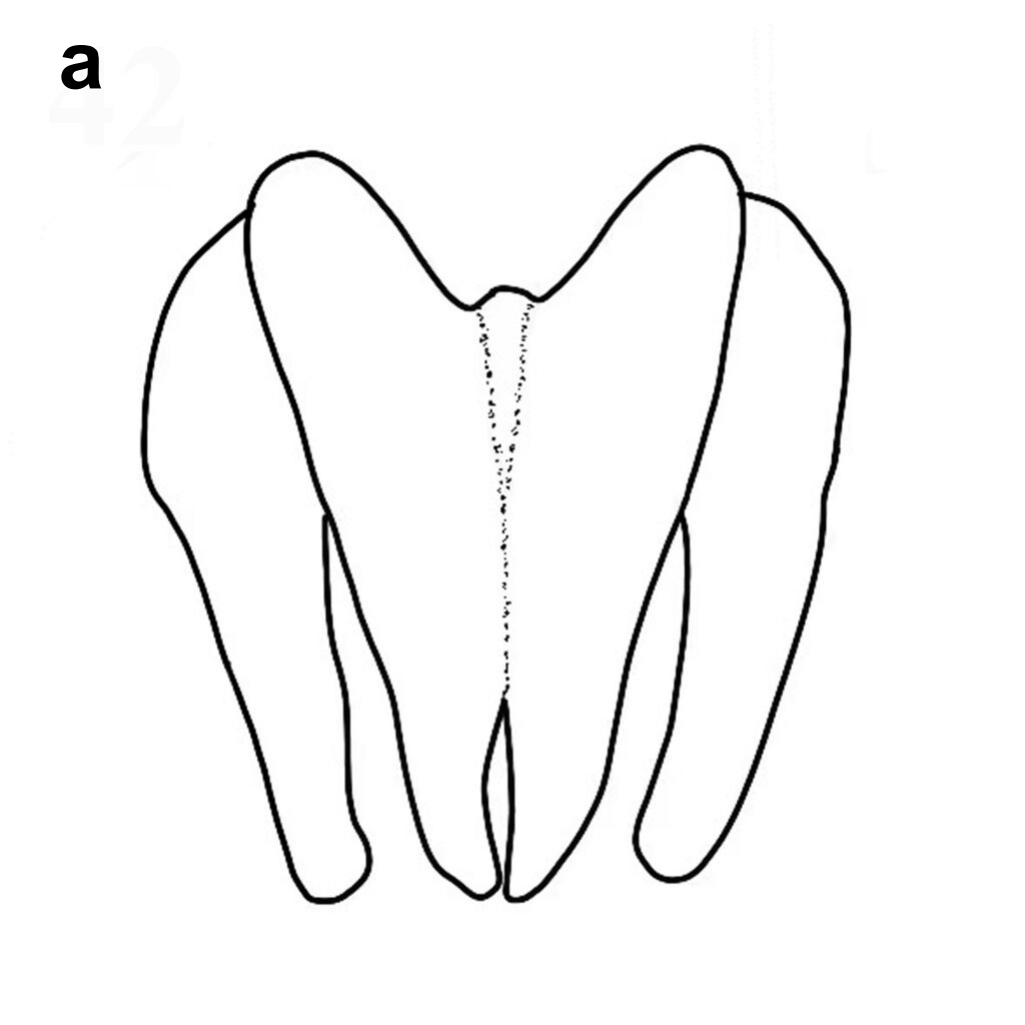
male cerci, surstyli in caudal view.

**Figure 31b.**
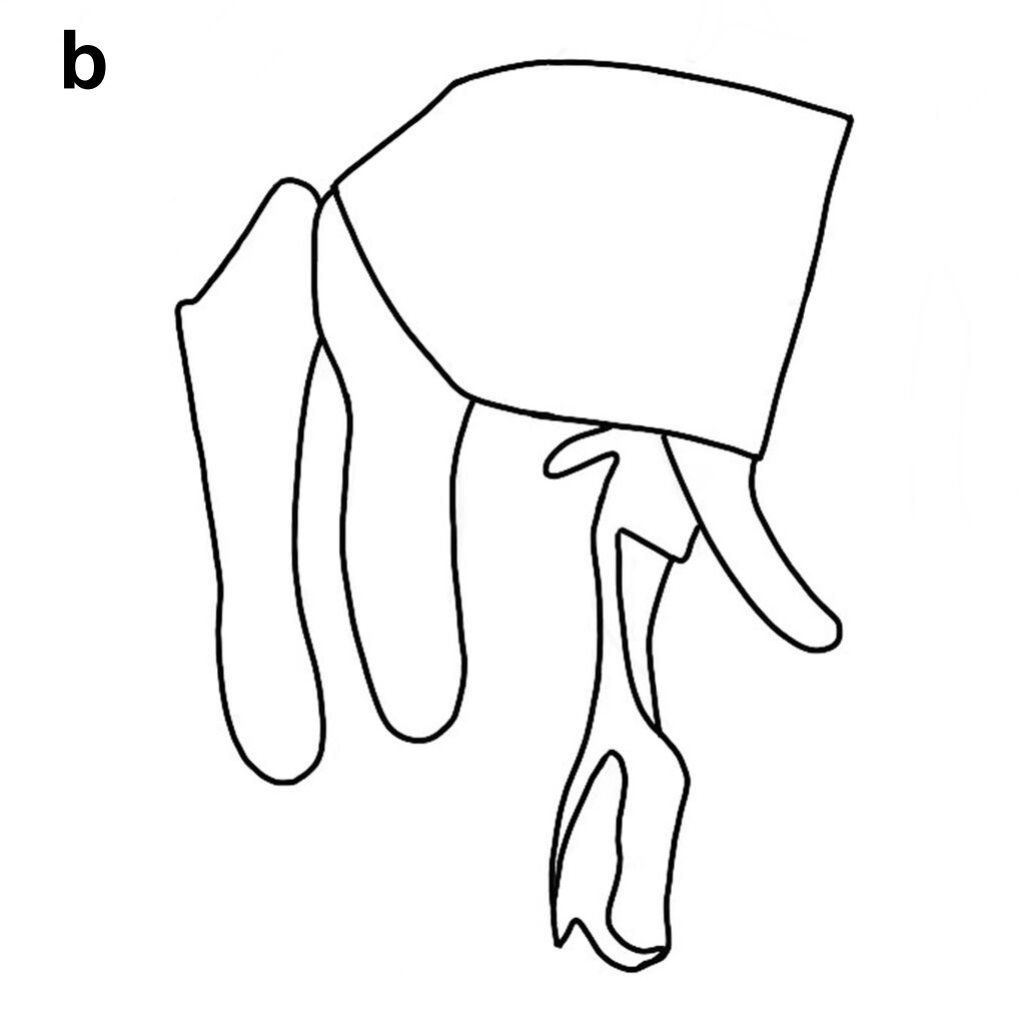
male cercus, surstylus, epandrium and phallus (part) in lateral view.

**Figure 32a.**
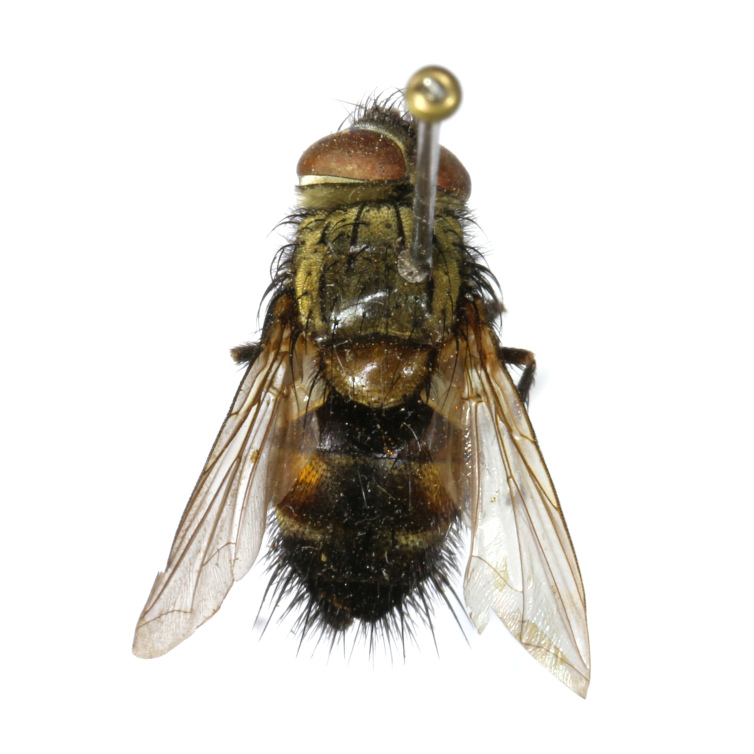
male body in dorsal view

**Figure 32b.**
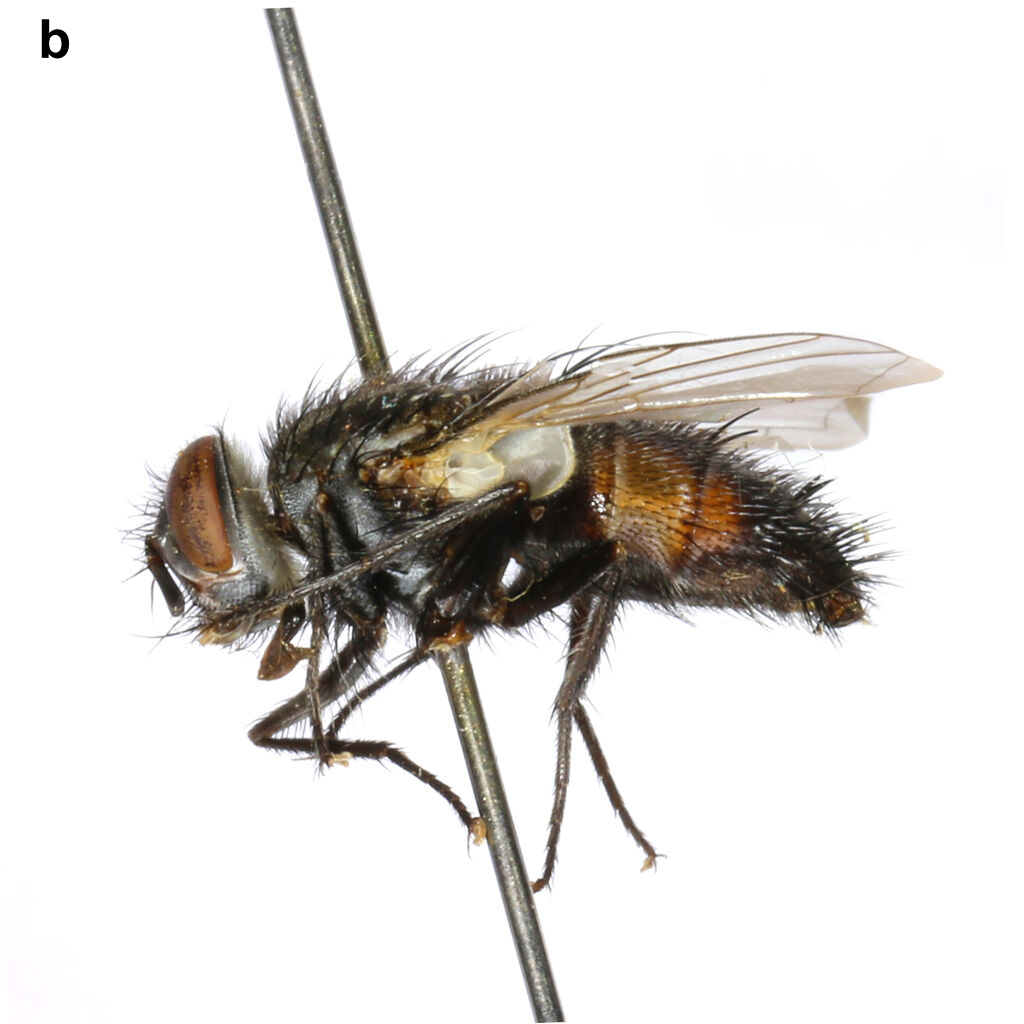
male body in lateral view

**Figure 32c.**
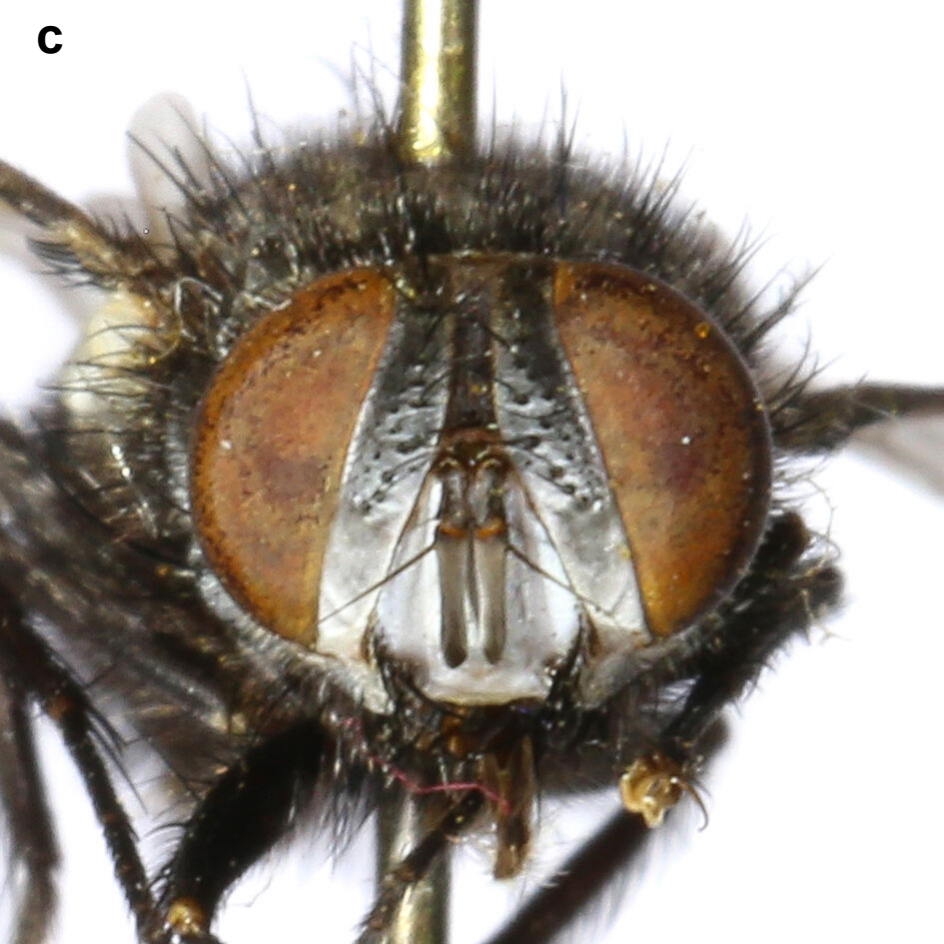
male head in anterior view

**Figure 32d.**
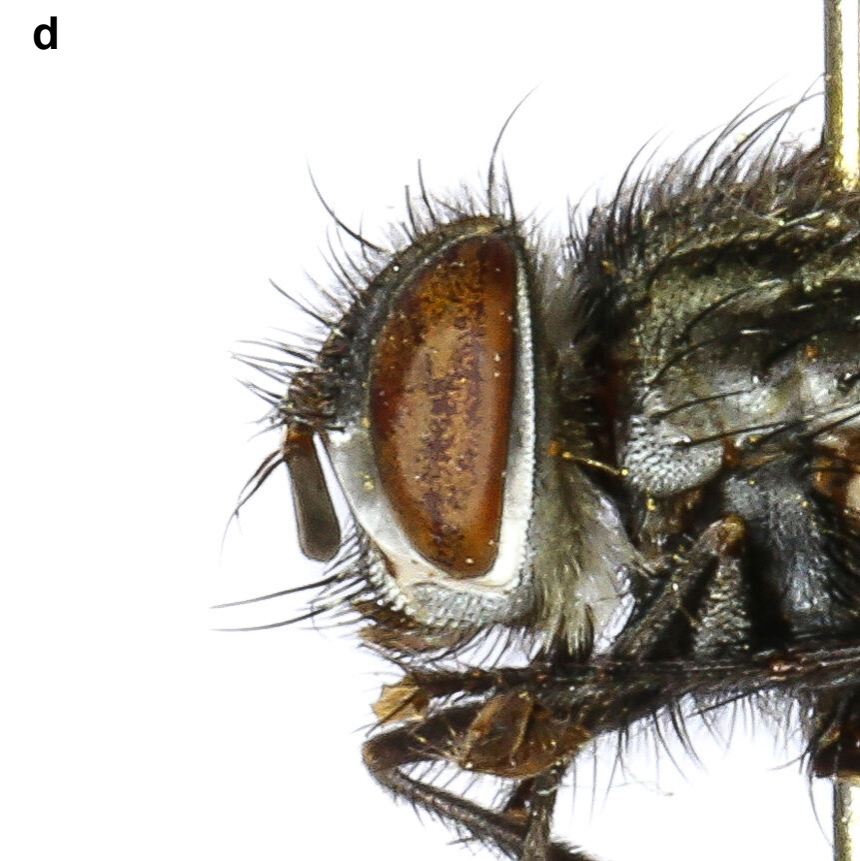
male body in lateral vie

**Figure 33a.**
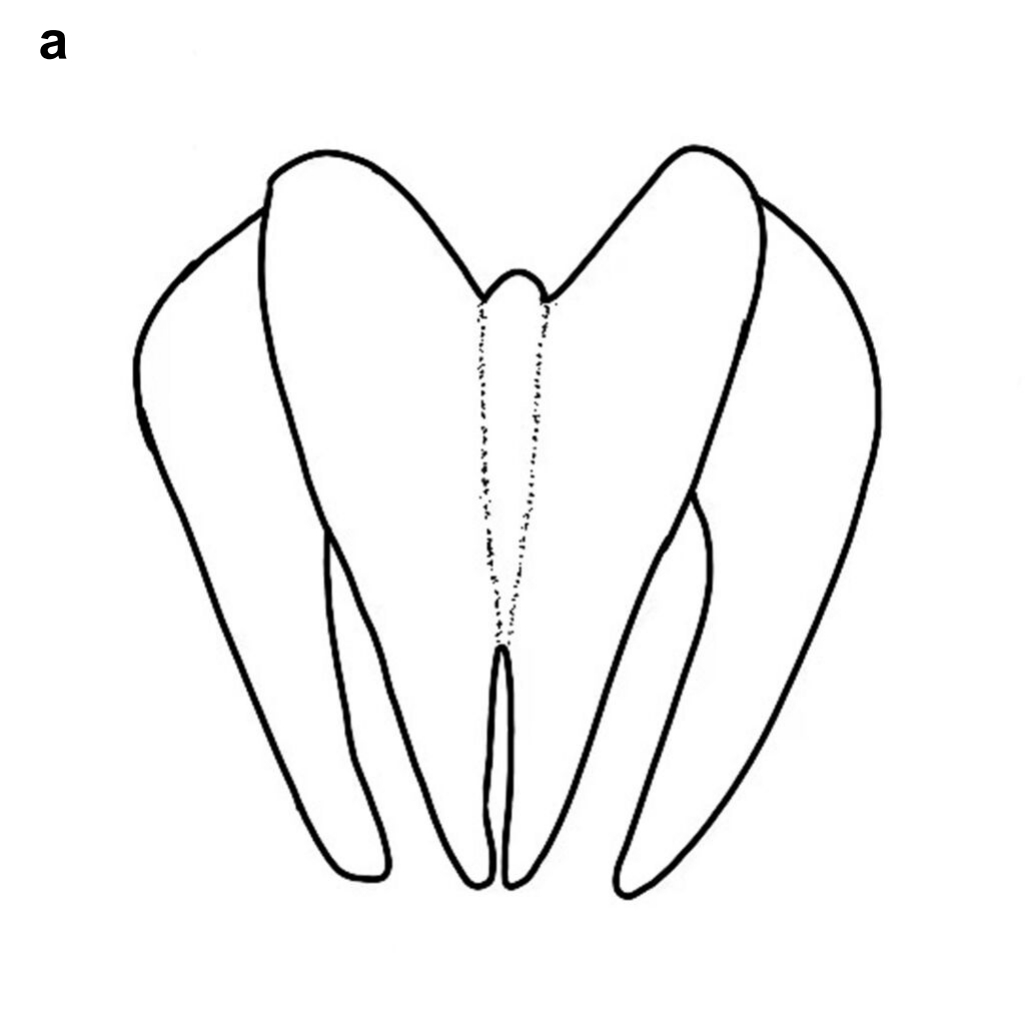
male cerci, surstyli in caudal view.

**Figure 33b.**
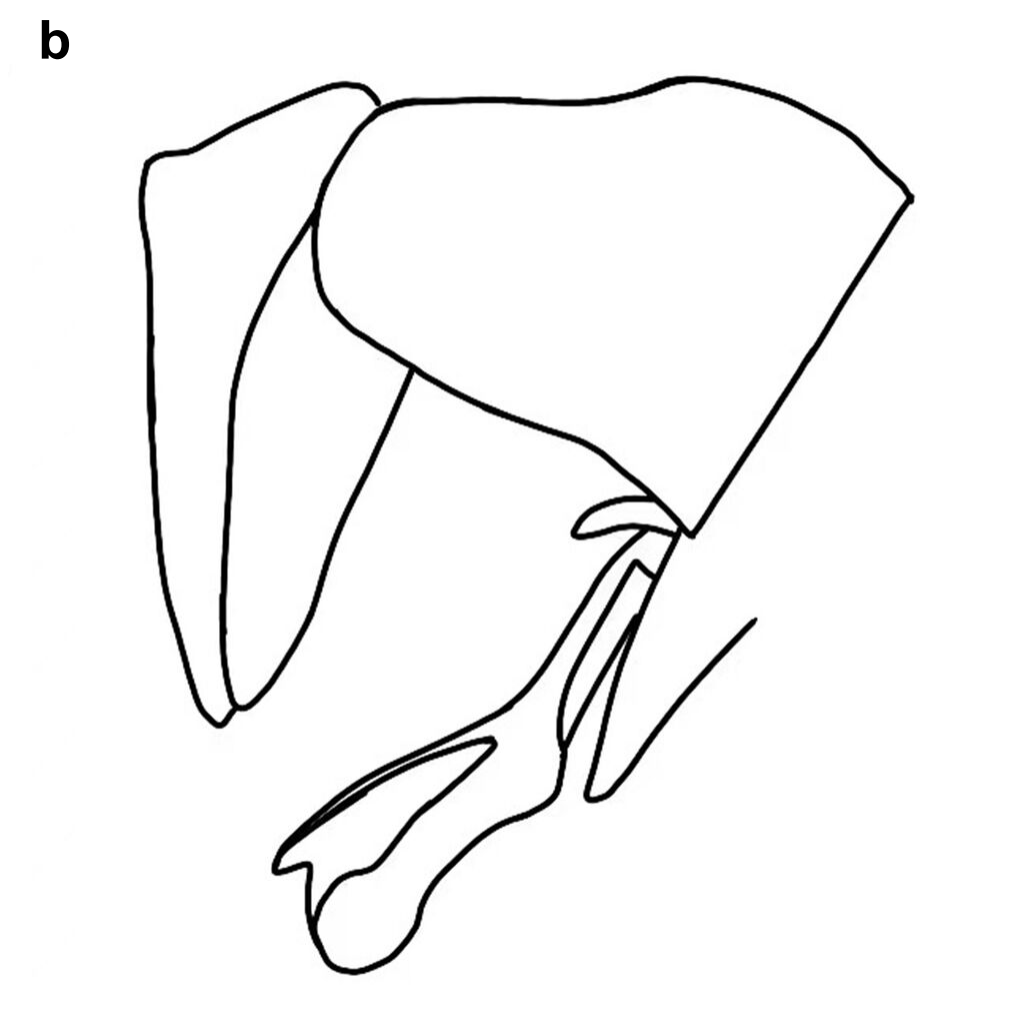
male cercus, surstylus, epandrium and phallus (part) in lateral view.

**Figure 34a.**
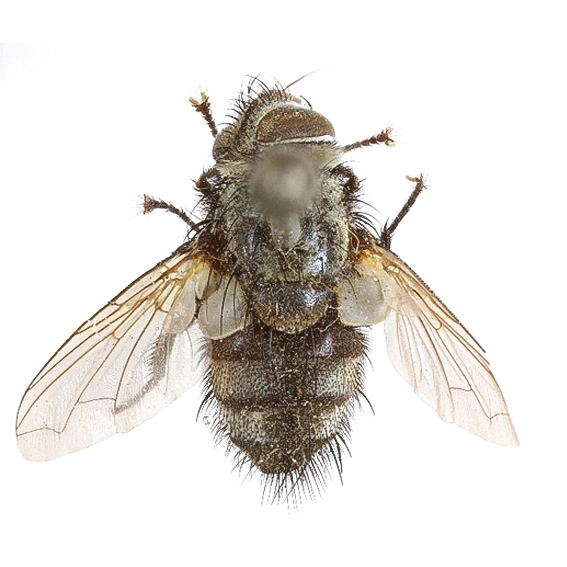
male body in dorsal view.

**Figure 34b.**
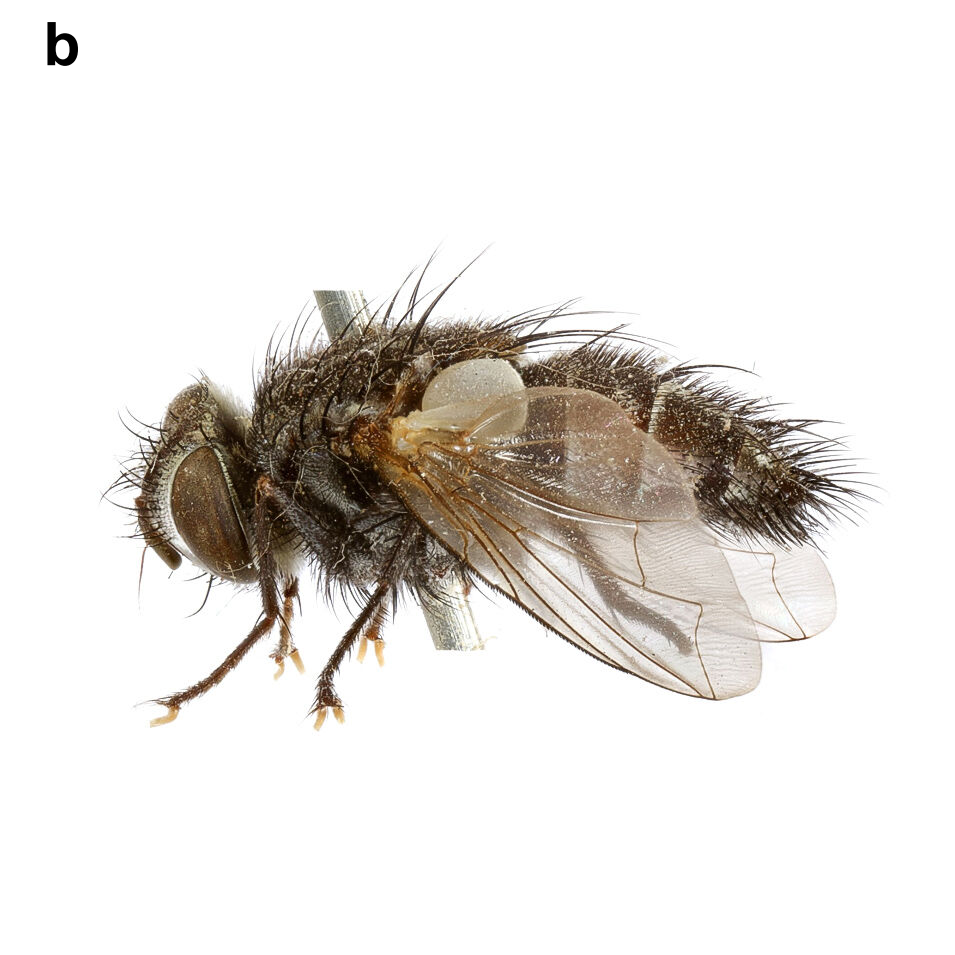
male body in lateral view.

**Figure 34c.**
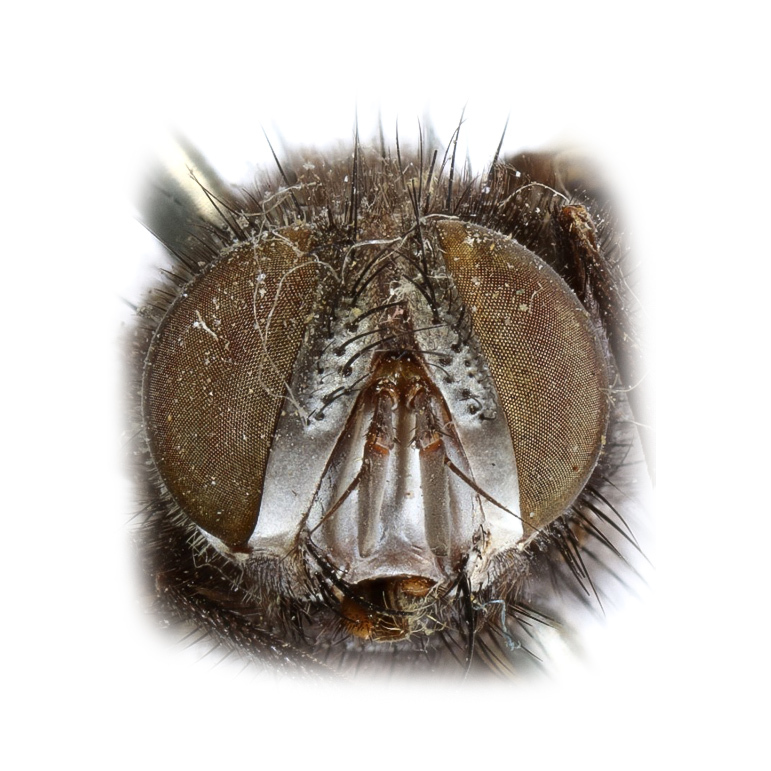
male head in anteriorview.

**Figure 34d.**
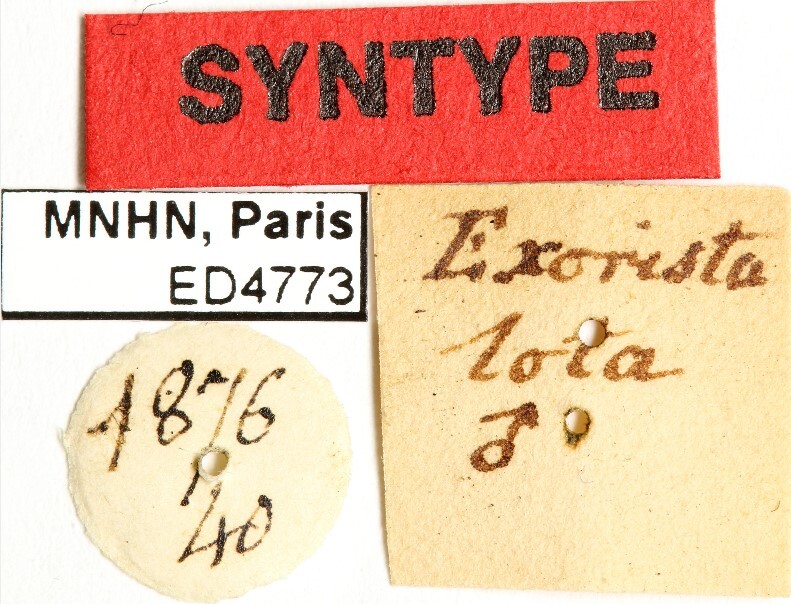
label information of *Exorista rapida* Meigen, 1838

**Figure 35a.**
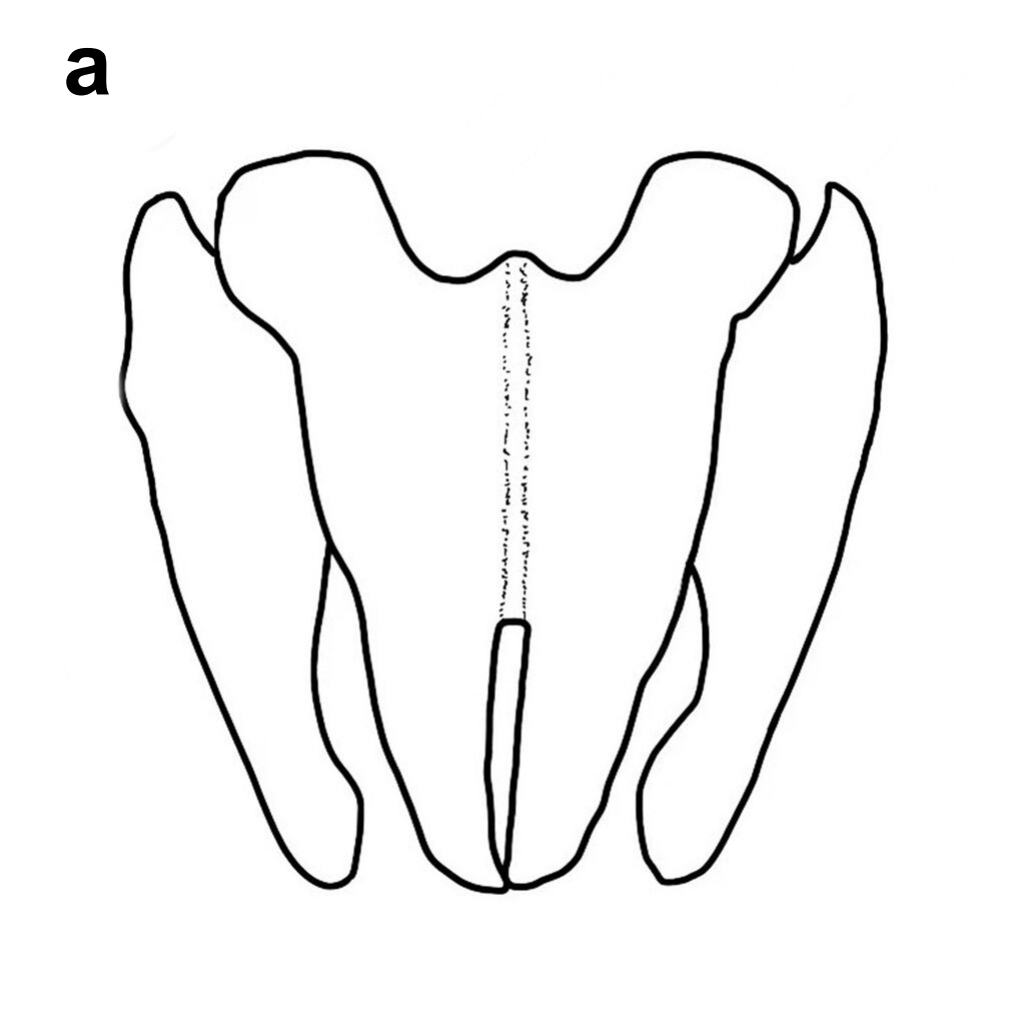
male cerci, surstyli in caudal view.

**Figure 35b.**
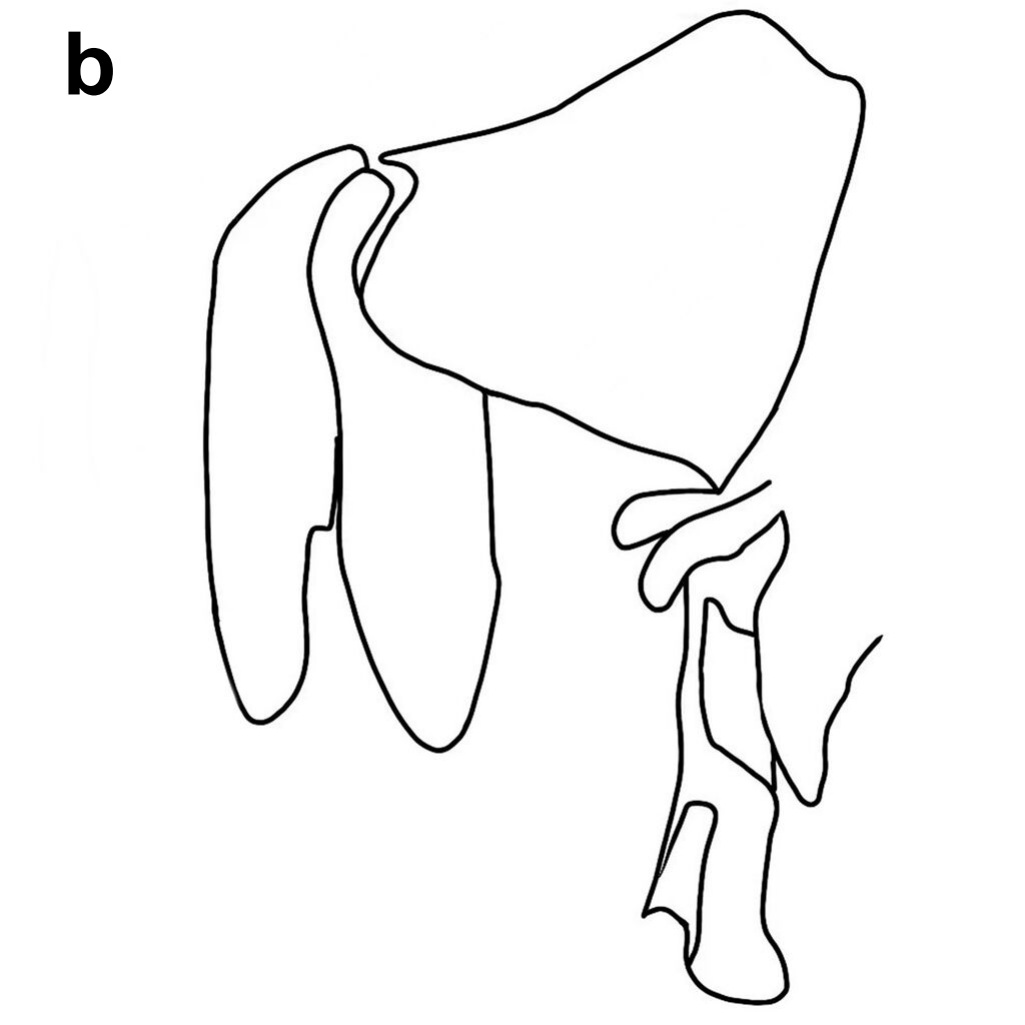
male cercus, surstylus, epandrium and phallus (part) in lateral view.

**Figure 36a.**
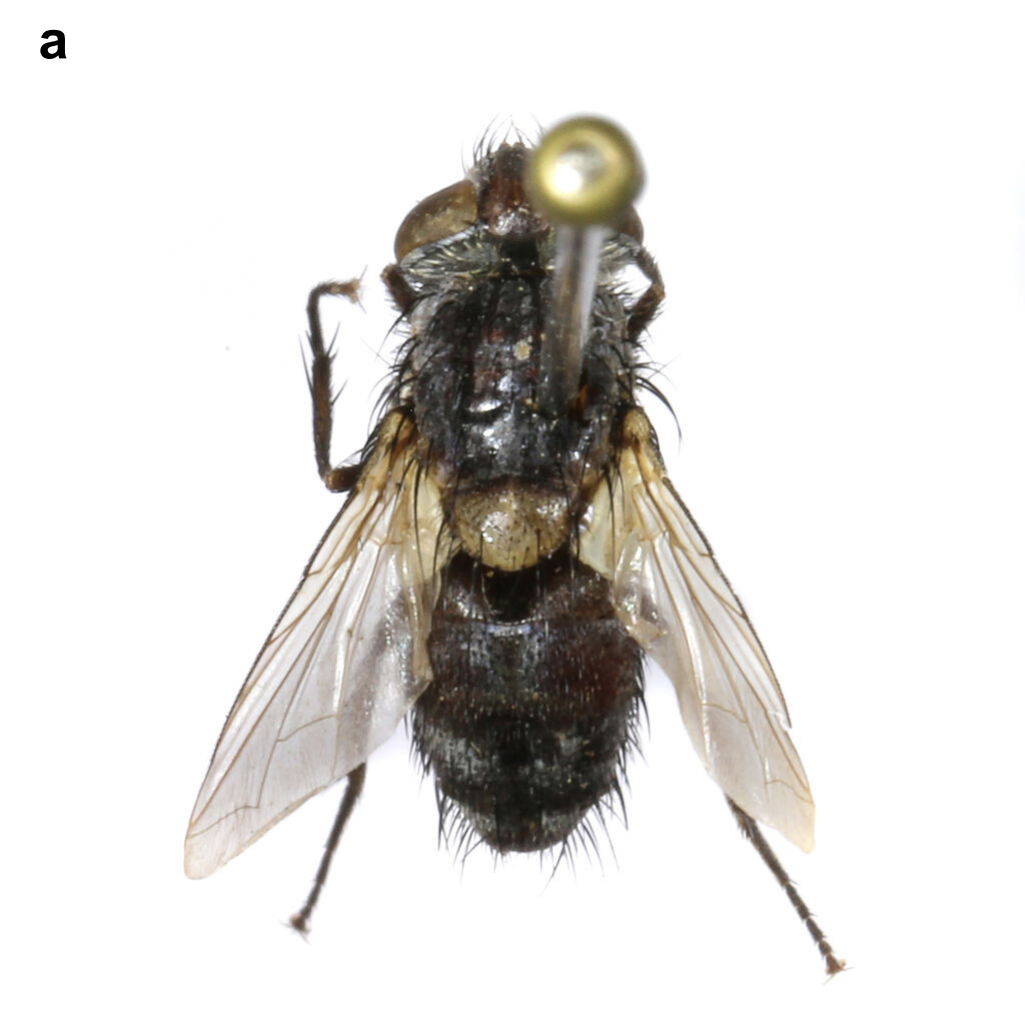
male body in dorsal view

**Figure 36b.**
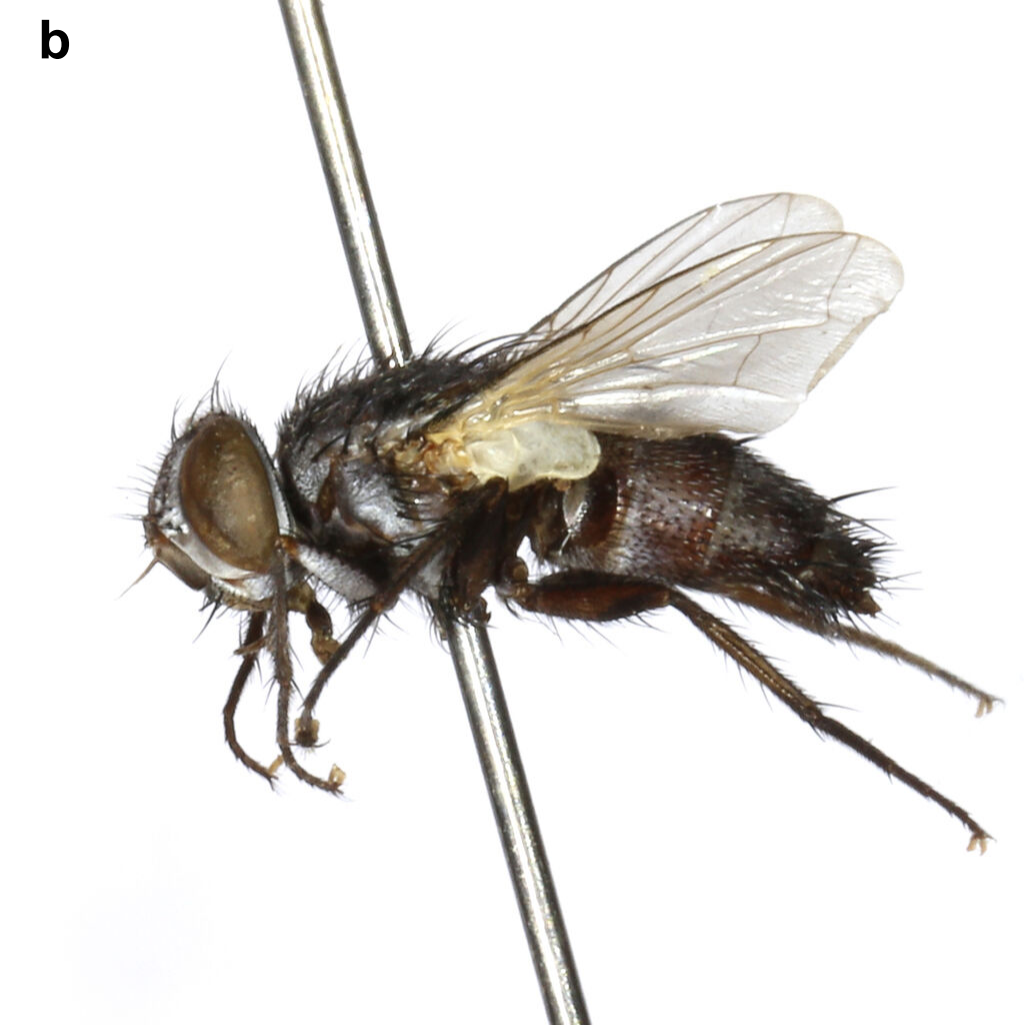
male body in lateral view

**Figure 36c.**
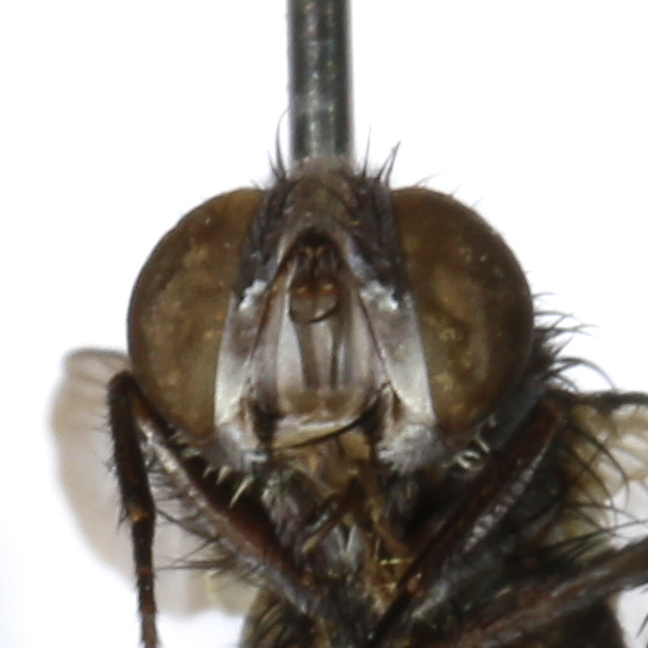
male head in anterior view

**Figure 36d.**
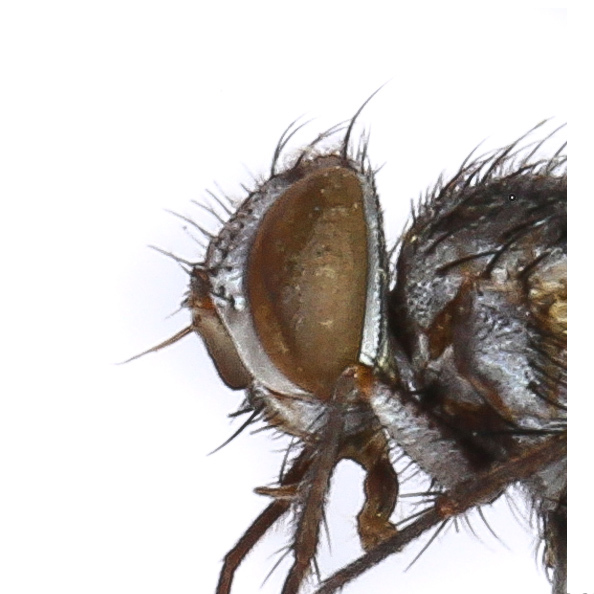
male head in lateral vie

**Figure 37a.**
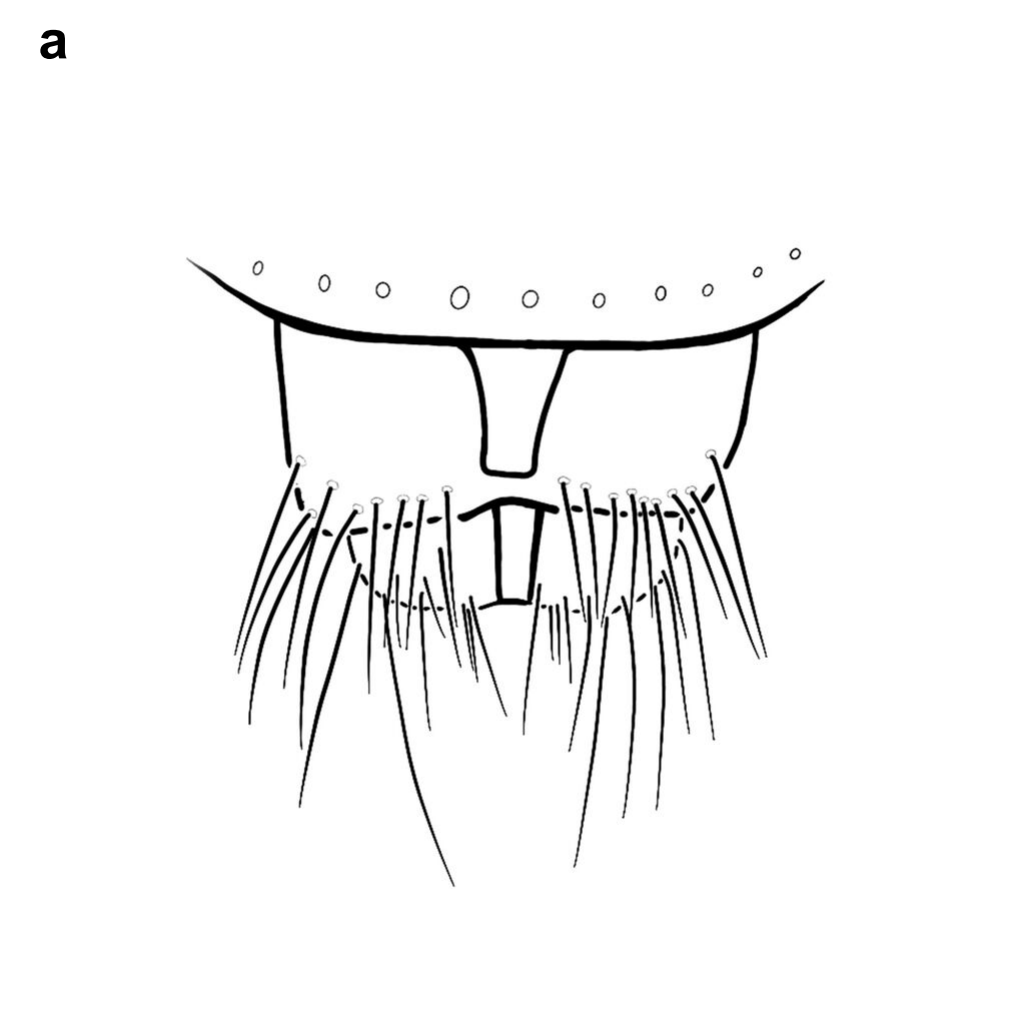
female postabdomen in dorsal view.

**Figure 37b.**
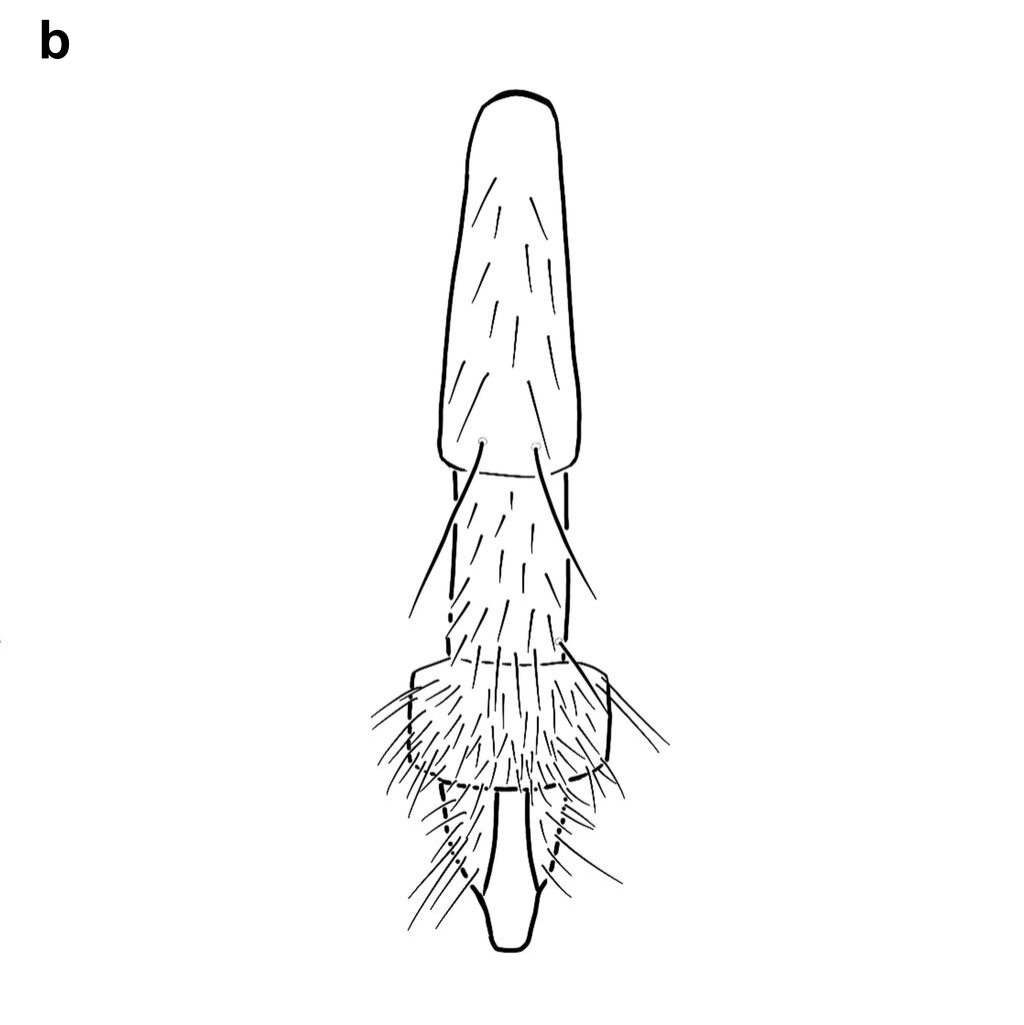
female postabdomen in ventral view.

**Figure 38a.**
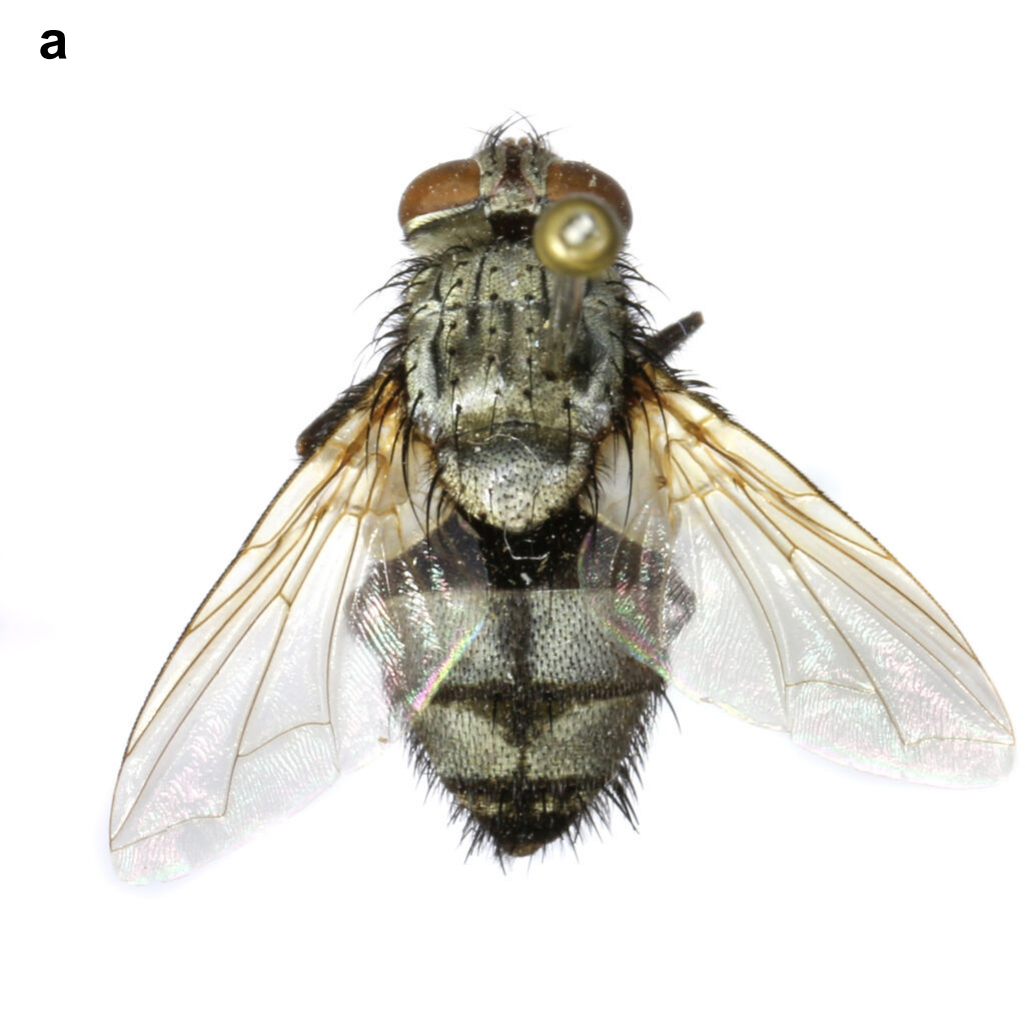
male body in dorsal view

**Figure 38b.**
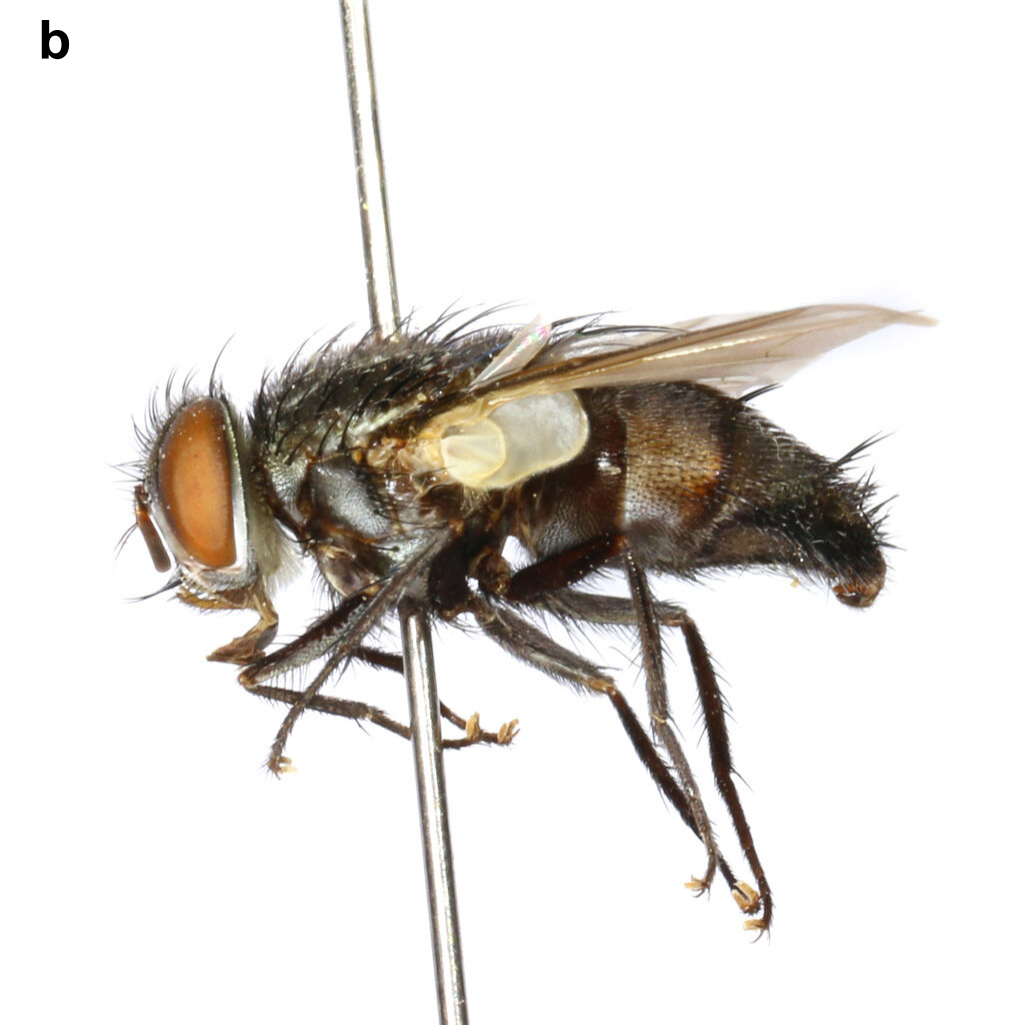
male body in lateral view

**Figure 38c.**
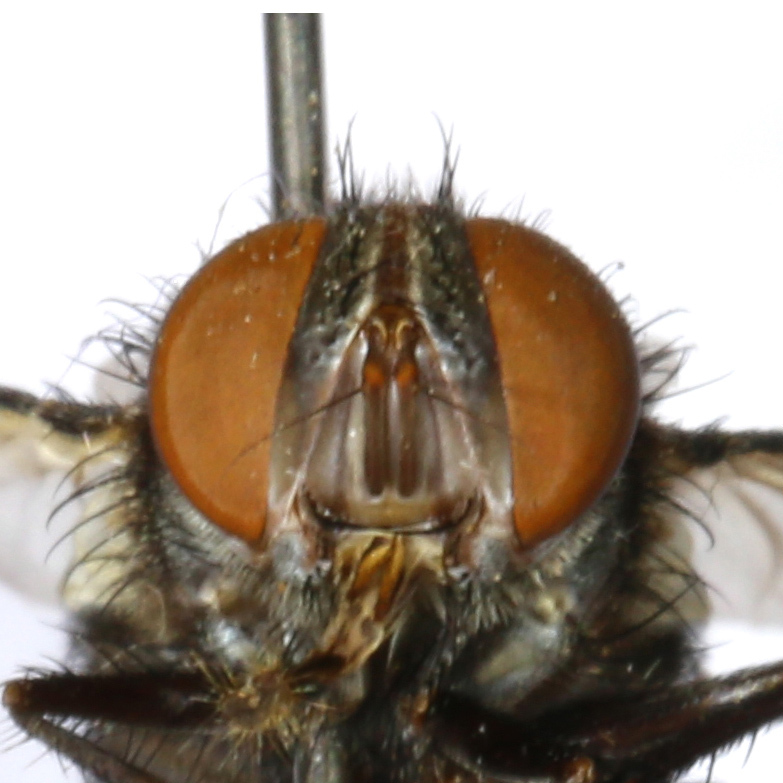
male head in anterior view

**Figure 38d.**
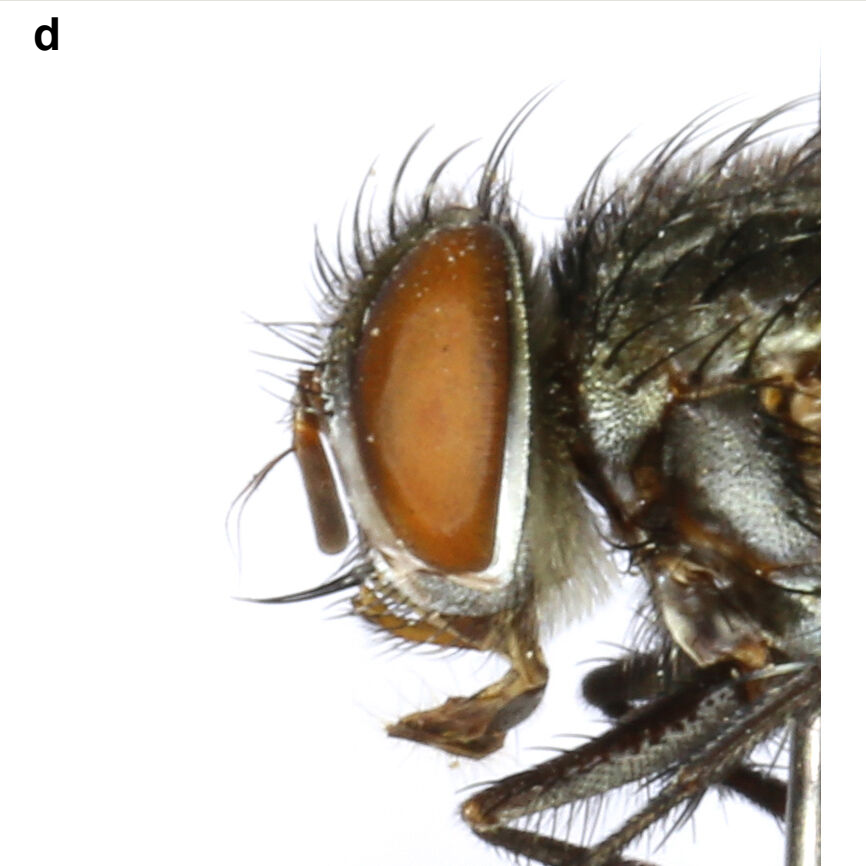
male head in lateral view

**Figure 39a.**
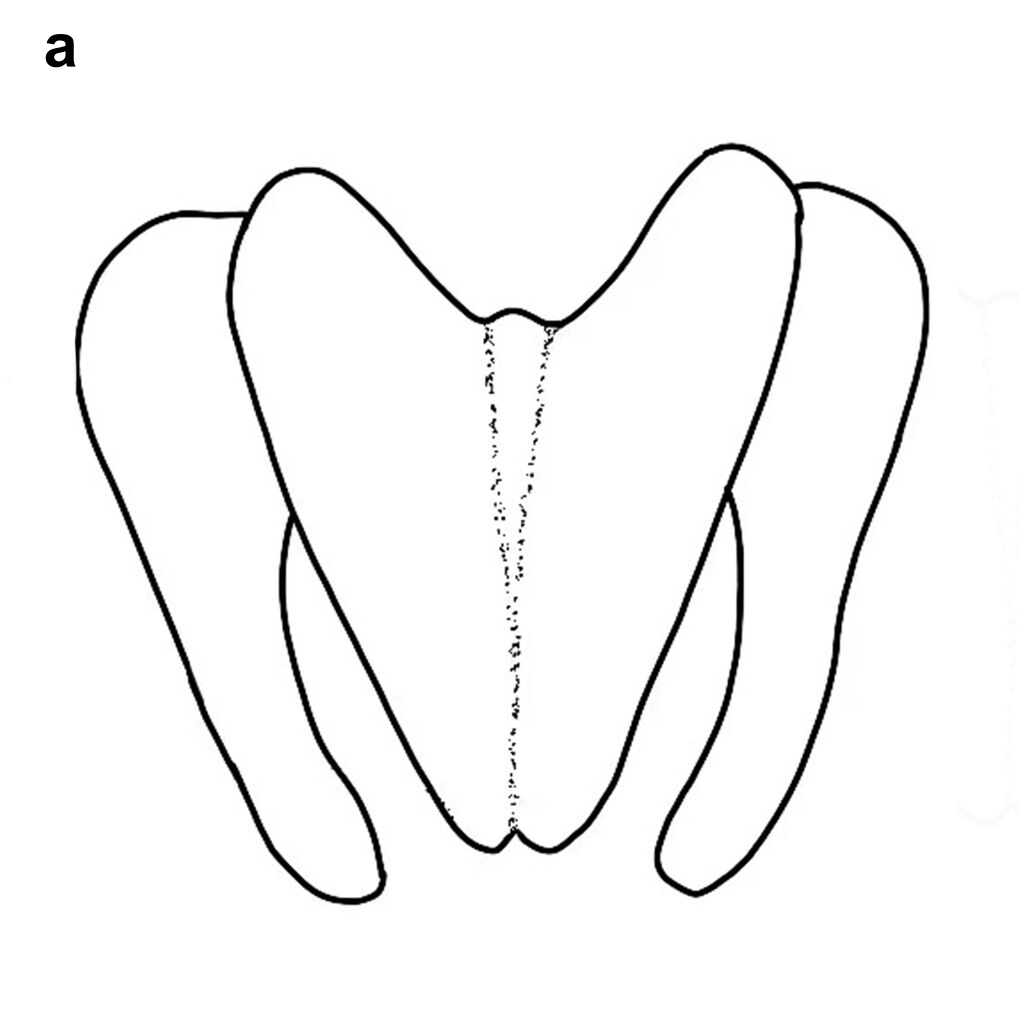
male cerci, surstyli in caudal view.

**Figure 39b.**
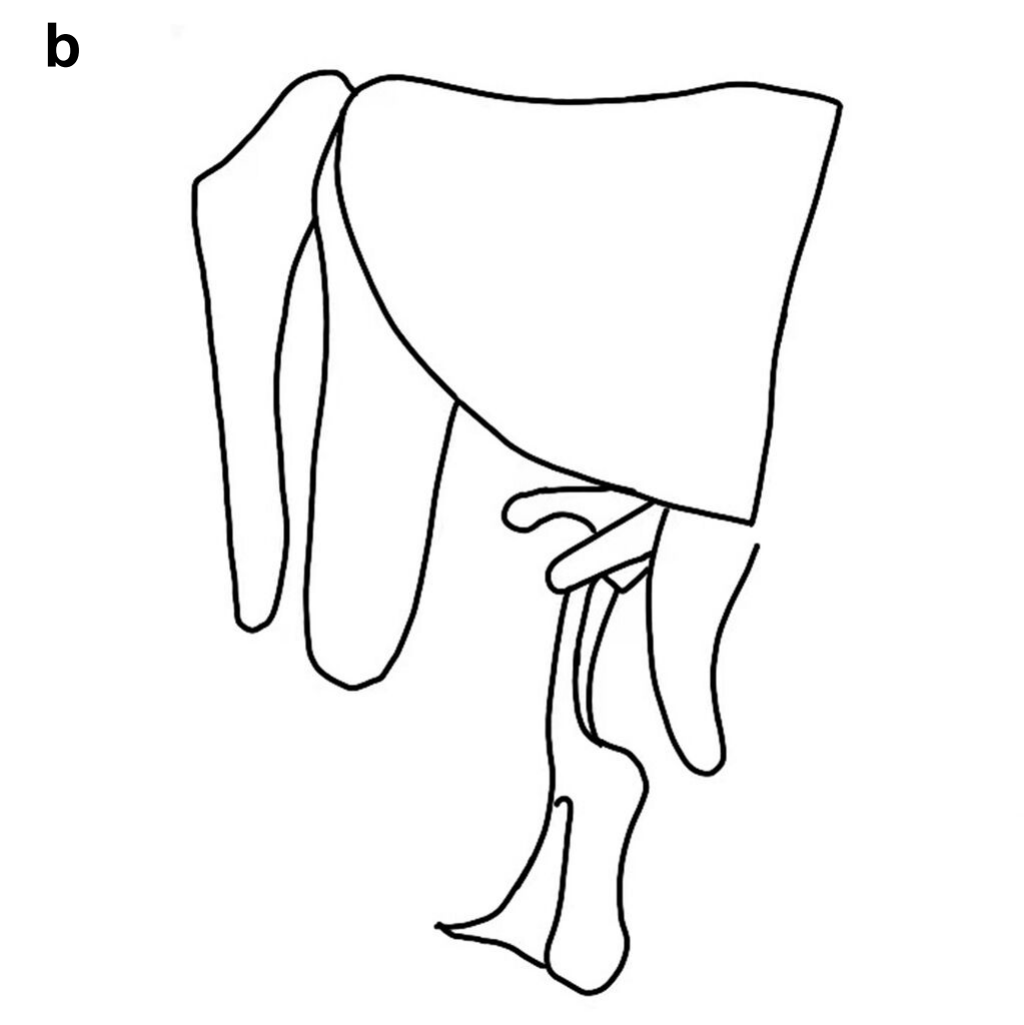
male cercus, surstylus, epandrium and phallus (part) in lateral view.
